# Comprehensive landscape of resistance mechanisms for neoadjuvant therapy in esophageal squamous cell carcinoma by single-cell transcriptomics

**DOI:** 10.1038/s41392-023-01518-0

**Published:** 2023-08-11

**Authors:** Yushang Yang, Yanguo Li, Haopeng Yu, Zhenyu Ding, Longqi Chen, Xiaoxi Zeng, Shunmin He, Qi Liao, Yi Zhao, Yong Yuan

**Affiliations:** 1 Department of Thoracic Surgery, West China Hospital, Sichuan University, Chengdu, Sichuan China; 2 Zhejiang Key Laboratory of Pathophysiology, Health Science Center, Ningbo University, Ningbo, Zhejiang China; 3 West China Biomedical Big Data Center, West China Hospital, Sichuan University, Chengdu, Sichuan China

**Keywords:** Drug development, Predictive medicine, Target identification, Head and neck cancer, Cancer microenvironment

**Dear Editor**,

Preoperative neoadjuvant therapy combined with surgical resection is the standard treatment for locally advanced esophageal squamous cell carcinoma (ESCC). However, more than half of patients having a partial response to neoadjuvant therapy, which is considered as a therapy-resistant phenotype and the mechanism is still unclear. The heterogeneity of the ESCC with surgery alone therapy were characterized by single-cell RNA sequencing (scRNA-seq) previously, but few report was about ESCC patients with neoadjuvant therapy.^1^ It is emergent to illustrate the comprehensive hallmarks of neoadjuvant therapy-resistance in ESCC at single cell level.

We generated scRNA-seq profiles for 7 ESCC patients underwent postoperative neoadjuvant therapy but having a partial pathological response, and 2 patients with surgery alone (Supplementary Fig. 1a, Supplementary Table 1). The cells were then classified into 7 cell lineages according to authoritative markers^2^ (Fig. 1a, Supplementary Fig. 1b), the proportions of which revealed the cellular landscape of ESCC patients undergoing different therapy strategies (Fig. 1b, Supplementary Fig. 1c). We further partitioned epithelial cells into malignant and normal cells according to copy number variations (CNVs, Fig. 1c). Considering the malignant cells that survive after neoadjuvant therapy may acquire therapy resistance ability, only malignant cells were reserved to identify resistant cell lineages to neoadjuvant therapy.

**Fig. 1:**
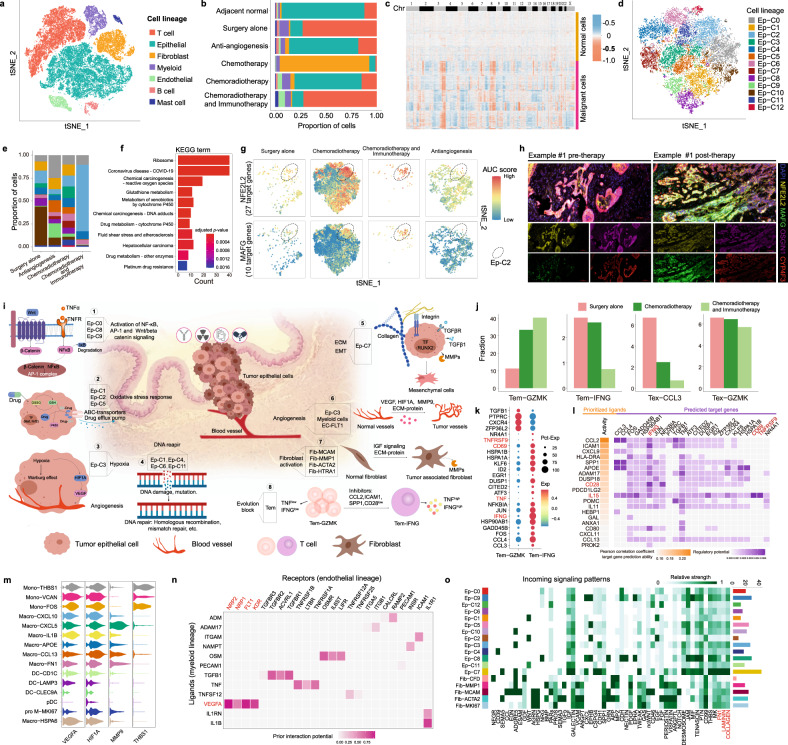
**a** t-SNE plot of high-quality cells across all samples, colored by cell lineage. **b** Proportions of different cell lineages, categorized by neoadjuvant therapy strategy and colored according to cell lineage. **c** Heatmap shows the predicted CNVs by CopyKAT among epithelial cells across all chromosomes. The cells were clustered into two groups based on CNVs, where malignant epithelial cells had more CNVs than normal epithelial cells. **d** t-SNE plot of all malignant epithelial cells (Ep) in our cohort, colored by tumor cell lineages. **e** Proportions of tumor cell lineages in different therapy strategies. **f** Enriched KEGG pathways of Ep-C2 lineage based on marker genes. **g** t-SNE visualization of AUC scores of two regulons (TF and its target genes), split by therapy strategies. **h** Multiplex immunohistochemistry of pre- and post-therapy samples using the antibodies and colors are as follows: DAPI (blue), NFE2L2 (yellow), MAFG (green), OSGIN1 (Magenta), and CYP4F3 (red). **i** Resistance hallmarks of ESCC to neoadjuvant therapy. ECM extracellular matrix, EMT epithelial mesenchymal transition, TF transcription factor, MMPs matrix metalloproteinases, Tem effector memory T cell. **j** The average fractions of four types of T cell lineages under different neoadjuvant therapy. Tem effector memory T cells, Tex exhausted T cells. **k** Dot plot shows differentially expressed genes between Tem-GZMK and Tem-IFNG cells. **l** NicheNet analysis shows the potential ligands expressed by neighboring cells that presumably affected the differentially expressed genes between Tem-GZMK and Tem-IFNG cells. Ligand activity indicates the ability of each ligand to predict the target genes, and better predictive ligands are thus ranked higher. The regulatory potential score indicates the confidence that a particular ligand can regulate the expression of a particular target gene. **m** Violin plot shows average gene expression across all myeloid sub-populations, colored by lineage identity. Mono monocyte, Macro macrophage, DC classical dendritic cell, pDC plasmacytoid dendritic cell, pro M proliferation monocyte/macrophage. **n** Ligand-receptor interaction analysis between the myeloid lineage and endothelial lineage. Prior interaction potential score indicates the confidence that a particular ligand can interact with a particular receptor. **o** Relative interaction strength of each incoming signaling pathway among tumor cell lineages and fibroblasts in samples from chemoradiotherapy patients

After dimension reduction and clustering of malignant cells, we identified 13 robust clusters of tumor cell lineages (Fig. 1d, Supplementary Fig. 1d), which were variously distributed in samples underwent different therapy strategies (Fig. 1e), indicating a potential association between tumor cell lineage and therapy resistance. Among them, the survival of Ep-C2 exhibited the highest proportion in patient who underwent chemoradiotherapy and immunotherapy combination (Fig. 1e). We found the marker genes of Ep-C2 (Supplementary Table 2), such as *OSGIN1* and *CYP4F3*, are related to oxidative stress response and drug metabolism pathways, including glutathione metabolism, cytochrome P450-related pathway and platinum-drug resistance (Fig. 1f, Supplementary Fig. 1e). Transcription factor regulatory network analysis showed that the regulon *MAFG* (10 target genes) and *NFE2L2* (27 target genes), the critical factors for antioxidant response signaling pathway, were activated in Ep-C2 (Fig. 1g, Supplementary Fig. 1f). We conducted a multiplex immunohistochemistry on 8 patients, including pre- and post-therapy (chemotherapy combined with immunotherapy). Representative images of individual samples stained with marker genes of Ep-C2, including *NFE2L2*, *MAFG*, *OSGIN1*, and *CYP4F3* are shown in Fig. 1h. Additionally, the findings demonstrated a significant increase in the expression of these genes in post-therapy samples (Supplementary Fig. 1g). These findings suggest that Ep-C2 with antioxidant characteristic existed before neoadjuvant therapy and transformed into a resistant population after neoadjuvant therapy.

We next imputed the enrichment score of Ep-C2 gene signature (Supplementary Table 3) in another cohort of ESCC without neoadjuvant therapy. Results showed the tumor cells from patient T_865 had the highest Ep-C2 enrichment score and highly expressed the antioxidant response-related genes (Supplementary Fig. 1h, i). Additionally, the Ep-C2 gene signature was also highly enriched in some TCGA-ESCC samples (Supplementary Fig. 1j), indicating the existence of Ep-C2 in ESCC. We then identify potential drugs that could reverse the gene expression and restore therapy sensitivity of Ep-C2 by using L1000 CMAP signatures,^3^ and the top 5 drugs are shown in Supplementary Fig. 1k. As the synthesis of glutathione occurs in an ATP-dependent manner, ouabain and digoxin, which target ATPase, may repress glutathione metabolism of Ep-C2. Overall, this data provided potential targets to combat therapy resistance of Ep-C2.

Meanwhile, according to marker genes expression, pathway and TF activity, the molecular mechanisms and hallmarks of therapy resistance in other heterogeneous tumor cell lineages were also described briefly (Fig. 1i). Gene sets related to DNA repair and cell cycle were activated strongly in the Ep-C1, Ep-C4, Ep-C6, and Ep-C11 lineages (Fig. 1i, Supplementary Fig. 2a, b). The survival of the Ep-C3 lineage with high expressions of *NDRG1*, *SLC2A1*, and *VEGFA*, showed high activity of hypoxia and angiogenesis (Fig. 1i, Supplementary Table 2, Supplementary Fig. 2a). However, Ep-C10 exhibited the highest activation of interferon responses (Supplementary Fig. 2a) and the highest infiltration in patients who underwent general surgery alone (Fig. 1e), indicating Ep-C10 lineage was sensitive to neoadjuvant therapy. Moreover, our study demonstrated that part of tumor sub-populations possesses single therapy-resistance mechanism, whereas some show coexisting of multiple therapy-resistance mechanisms (Fig. 1i). Therefore, exploring comprehensive hallmarks of substantial heterogeneous tumor cells can provide foundation for precision therapy.

T cell heterogeneity and dynamics are important in therapy responses. We found the fraction of *GZMK*^+^ effector memory T cells (Tem-GZMK) was increased in most neoadjuvant therapy patients, especially in the patient who underwent immunotherapy (Fig. 1j, Supplementary Fig. 2c). While the expression levels of cytokines and effector molecules such as *IFNG* and *TNF* in Tem-GZMK cells were lower than in *IFNG*^+^ effector memory T cells (Tem-IFNG) (Fig. 1k), demonstrating the cytotoxic-insufficient status of Tem-GZMK cells. According to the T cells infiltration and evolutionary trajectory (Fig. 1j, Supplementary Fig. 2d), we inferred that the evolution of Tem-GZMK to Tem-IFNG may be inhibited and recover process could be an important means of improving therapeutic effect (Fig. 1i). In ESCC ligand-receptor network, *IL15* exhibited strong regulatory effect to *CD69* and *TNFRSF9* (Fig. 1l), which were important molecules for T cell activation and cytotoxic T cell expansion. However, the insufficiency of *IL15* in the tumor microenvironment (TME, Supplementary Fig. 2e) leading us to hypothesized that combining *IL15* agonists with neoadjuvant therapy would be essential to improve therapy effect.

Myeloid lineages execute therapy resistance through promoting angiogenesis. Typically, we found that most myeloid lineages in tumor tissue expressed well-known angiogenesis inducers such as *VEGFA*, *HIF1A*, and *MMP9*, while only monocyte cells highly expressed the endogenous angiogenesis inhibitor thrombospondin-1 (*THBS1*) (Fig.1m). We assessed cell-cell communication (CCC) between endothelial cells and myeloid cells in the patients treated with the anti-angiogenesis drug apatinib. We found *VEGFA* interact not only with the receptor *VEGFR* (*FLT1*, *KDR*) but also with the alternative receptors *NRP1* and *NRP2* (Fig. 1n), which transmit strong proliferation signals to endothelial cells and lead to the failure of anti-angiogenesis drug. Analysis of endothelial cells also identified tumor-associated endothelial cell population, *i.e*., EC-FLT1 (Supplementary Fig. 2f), which receive angiogenic signals from TME.

We also found activation of tumor-associated fibroblast lineages would enhance therapeutic resistance through CCC (Fig. 1i, Supplementary Fig. 2g). Our results demonstrated stronger CCC exists in chemoradiotherapy and anti-angiogenesis therapy patients than those who underwent general surgery alone, whereas communication strength was suppressed in chemotherapy and immunotherapy patients (Supplementary Fig. 2h). The dynamic changes of communications such as EGF, IGF, CCL, and WNT were activated or up regulated in chemoradiotherapy patients, whereas SEMA5 and SEMA6 were activated in chemoradiotherapy and immunotherapy combination patient (Supplementary Fig. 2i). Further analyses of chemoradiotherapy patients showed that Ep-C7 cells receive the most signals from fibroblasts via the COLLAGEN, LAMININ, and FN1 pathways (Fig. 1o), which is in line with the functions of Ep-C7 cells, such as participating in ECM interactions and EMT (Fig. 1i). These results suggest that deposition of ECM proteins near Ep-C7 cells protects tumor cells from the cytocidal effects of certain therapies.

Collectively, we identified the comprehensive hallmarks of various tumor lineages that resist neoadjuvant therapy, and the TME cells associated with therapy resistance in ESCC. The study of post-neoadjuvant therapy ESCC patients at single cell level uncovered resistance mechanisms and potential targets for therapies.

### Supplementary information

Supplementary Materials

## Data Availability

The raw data used in this study has been deposited to GEO with accession number GSE221561. The cohort of scRNA-seq in ESCC patients with general surgery alone obtained with SRA accession: PRJNA777911. The ESCC bulk transcriptome data was obtained from TCGA data portal.
