# Supplementary material for: Comprehensive landscape of resistance mechanisms for neoadjuvant therapy in esophageal squamous cell carcinoma by single-cell transcriptomics
Source: Signal Transduct Target Ther. 2023 Aug 11;8:298. doi: 10.1038/s41392-023-01518-0 (PMC10415278; doi:10.1038/s41392-023-01518-0)
Supplement: Supplementary file 1 — Supplementary Materials [file 41392_2023_1518_MOESM1_ESM.docx]

Supplementary Materials for

**Comprehensive Landscape of Resistance Mechanisms for Neoadjuvant Therapy in Esophageal Squamous Cell Carcinoma by single-cell Transcriptomics**

Yushang Yang^1, #^, Yanguo Li^2, #^, Haopeng Yu^1, #^, Zhenyu Ding^1^, Longqi Chen^1^, Xiaoxi Zeng^3^, Shunmin He^3^, Qi Liao^2, *^, Yi Zhao^3, *^, Yong Yuan^1, *^

### Affiliations

1. Department of Thoracic Surgery, West China Hospital, Sichuan University, Chengdu, Sichuan, China
2. Zhejiang Key Laboratory of Pathophysiology, Health Science Center, Ningbo University, Ningbo, Zhejiang, China
3. West China Biomedical Big Data Center, West China Hospital, Sichuan University, Chengdu, Sichuan, China

These authors contributed equally: Yushang Yang, Yanguo Li, Haopeng Yu

**Correspondence author:**

**Yong Yuan**

Department of Thoracic Surgery, West China Hospital, Sichuan University, Chengdu, Sichuan, China; E-mail: [yongyuan@scu.edu.cn](mailto:yongyuan@scu.edu.cn)

**Yi Zhao**

West China Biomedical Big Data Center, West China Hospital, Sichuan University, Chengdu, Sichuan, China; E-mail: [biozy@ict.ac.cn](mailto:biozy@ict.ac.cn)

**Qi Liao**

Zhejiang Key Laboratory of Pathophysiology, Health Science Center, Ningbo University, Ningbo, Zhejiang, China; E-mail: [liaoqi@nbu.edu.cn](mailto:liaoqi@nbu.edu.cn)

This PDF file includes:

**Materials and Methods**

**Supplementary Figures 1 and 2**

**Supplementary Table 1-3**

**Materials and methods**

**Patients and ethics statement**

Nine patients who were pathologically diagnosed with ESCC were enrolled in this study. The clinical characteristics of each patient are summarized in Supplementary Table 1. Fresh tumor biopsies were collected post-esophagectomy from the primary tumor site for scRNA-seq analysis. All clinical samples were collected at West China Hospital. Written informed consent was obtained from all participants, and ethical approval was obtained from the Institutional Review Board of West China Hospital of Sichuan University (2019-632).

**Preparation of single-cell suspensions and single cell RNA sequencing**

The samples for scRNA-seq were washed with phosphate-buffered saline (PBS; Thermo Fisher Scientific, Waltham, MA, USA), placed on ice, cut into small pieces (< 2 mm3) and transferred to 5 mL Roswell Park Memorial Institute 1640 (RPMI; Thermo Fisher Scientific) containing collagenase IV (1 μg/mL), collagenase I (2 μg/mL) and DNase (1 μg/mL) (Thermo Fisher Scientific). The samples were incubated for 40 min at 37 °C with manual shaking every 5 min and then filtered through both 70-µm and 40-µm nylon mesh (Thermo Fisher Scientific). Following centrifugation (300 × g, 4 °C, and 5 min), the samples were then resuspended in PBS containing 0.04% bovine serum albumin (BSA; Sigma). Next, the cells were counted and assessed for viability using Trypan blue staining with a hemocytometer. During the dissociation procedure, the cells were kept on ice whenever possible, and the entire procedure was completed in < 90 min (generally ~70 min) to avoid the dissociation-associated artifacts recently described. A positive signal for dissociation signatures that reflects dissociation-associated changes in gene expression was obtained in < 1% of the cells. Droplet-based scRNA-seq, a chromium single-cell 3′-library, was constructed using the Chromium Single-cell 3′-Library, Gel Bead & Multiplex Kit and Chip Kit (10× Genomics, Pleasanton, CA, USA) according to the manufacturer’s instructions. Cell suspensions were loaded onto a chromium single-cell chip along with reverse transcription (RT) master mix and single-cell 3′-gel beads, aiming for 2,000–8,000 single cells per reaction. The samples were processed using 10× Genomics V3 barcoding chemistry kits. Following cell lysis, first-strand cDNA synthesis and amplification were carried out according to the manufacturer’s instructions with cDNA amplification set for 12 cycles. Libraries were sequenced on an Illumina HiSeq X Ten system and mapped to the human reference genome (GRCh38) using CellRanger (v5.0), and 90,094 single cells were ultimately identified.

**Processing and visualization of scRNA-seq data**

scDblFinder^1^ was used to identify doublets across all cells in R software. The “Seurat” (v4.0)^2^ was used for downstream analysis. The complexity of a single cell was calculated based on the number of detected genes (nFeature_RNA) divided by the read count (nCount_RNA) on a log10 scale. These data were used to estimate ambient RNA droplets and filter out low-complexity cells, such as dead cells and granulocytes. Cells with singlet annotation and high quality (counts/cell > 500, genes/cell > 600, < 15% mitochondrial genes, < 0.1% erythrocytic genes, and complexity > 0.80) were then used for further analysis according to default parameters unless otherwise stated. Principal component analysis and the “Harmony” package (v1.0)^3^ were used to remove batch effects (each sample was seen as a batch) and integrate the data after normalization (“SCTransform” function with 1,500 variable features). For unsupervised clustering, we applied the “FindNeighbors” function with a reduction in harmony (k.param=50), and the “FindClusters” function with a resolution array and clustering tree were plotted using the “clustree” package to subjectively evaluate the robustness resolution (0.8). An elbow plot and quantitative approach developed by members of the teaching team at the Harvard Chan Bioinformatics Core (HBC) were used to determine the optimal number of principal components (1~34) for reduction and clustering. The HBC approach calculates where the principal components begin to elbow by taking the larger value of 1**)** the point at which the principal components only contribute 5% of the standard deviation and the principal components cumulatively contribute 80% of the standard deviation, or 2) the point at which the percent change in variation between the consecutive principal components is < 0.1%.

t-SNE was used for dimensional reduction and visualization. Similar pipelines were applied to different lineages for further clustering and sub-population discovery, and very few cells expressing multiple major lineage markers were removed. In particular, to negate the effect of heat shock protein expression in T cells, we removed the heat shock protein and DnaJ heat shock protein families from the variable features. Fibroblasts exhibited extreme differences in cell number between samples, so traditional normalization (“NormalizeData”) and scale (“ScaleData”) functions were used as alternative methods. Finally, the fibroblasts cluster results showed a cell-specific pattern instead of patient-specific pattern.

To identify marker genes within each cluster, we applied the “FindAllmarker” function with the “MAST” test (using a hurdle model tailored to scRNA-seq data) and at least a 0.5-fold difference (log-scale). Only positive markers with adjusted p values < 0.01 were conserved.

**Cell identity assignment**

Acknowledged cell markers and differentially expressed genes were used to infer the identity of major cell types: T cells (*CD3D*), B cells (*CD79A*), myeloid cells (*CD14*, *LYZ*), endothelial cells (*PECAM1*), fibroblasts (*COL1A1*, *DCN*), and epithelial cells (*EPCAM*, *CDH1*). SingleR function and Human Primary Cell Atlas data were used to verify the cell type identification results.

**Function enrichment and gene set variation analysis**

Functional enrichment analysis of interesting gene lists was conducted based on GO terms and KEGG pathway analysis using R package “clusterProfiler”. We used gene set variation analysis (GSVA) to perform reliable gene set enrichment analyses with default parameters based on hallmark gene sets available from the Molecular Signatures Database (V7.4). Drop-out events that lead to zero read counts in scRNA-seq and computational imputing remain controversial, so the average gene expression levels of all cells within each subcluster were input for GSVA analysis. A high GSVA score indicated that the founder genes of a particular gene set were highly activated.

**Trajectory inference**

To describe the dynamic changes and differentiation among sub-populations, trajectory analysis was performed using Monocle 3^4^ according to UMAP visualization.

**Ligand and receptor interaction analysis**

NicheNet analysis (https://github.com/saeyslab/nichenetr) was performed starting from a seurat object to interpret intercellular communication. The "Nichenet_seuratobj_aggregate" function was used to explain differential expression in a receiver cell type under two different conditions involving ligands expressed by sender cells. The "Nichenet_seuratobj_cluster_de" function was used to explain differential expression between two "receiver" cell clusters according to ligands expressed by neighboring cells. We also used CellChat (https://github.com/sqjin/CellChat) to infer intercellular communication networks for different signaling pathways^5^.

**Copy number variations and regulatory network analyses**

CopyKAT^6^ was used to infer copy number variations (CNVs), and epithelial cells with predicted aneuploidy were considered malignant tumor cells, which is common in human cancers. We chose 1 000 random stromal cells (except epithelial cells) and immune cells to represent a reference with near-diploid copy number profiles.

SCENIC was used to build a regulatory network and calculate the activity of regulatory networks in each cell lineage^7^. The AUCell algorithm was used to score regulon activity in each cell (AUC score).

**GSEA of the L1000 CMAP signatures**

CMAP is a resource that uses cellular responses to perturbation to identify relationships between diseases, genes, and therapeutics. L1000 CMAP signatures were constructed based on differentially expressed genes of cell lines under L1000 assay treatment conditions, and we collected the 30,970 down-regulated signatures from Harmonizome, and each of signature was identified according to different treatment conditions (e.g., small molecules and concentrations, cell lines, and time of treatment). We then applied GSEA to determine whether the marker genes of a particular population ranked by average fold-change were enriched in L1000 CMAP signatures. GSEA calculates the NES and Bonferroni-corrected p-value for each signature, and the small molecule with the highest NES is expected to have ability to down-regulate the marker genes. A list of small molecules targeting resistant populations was generated by setting the threshold to Bonferroni-corrected p-values < 0.05.

**Estimation of cell type abundance**

To construct the signatures for enrichment score estimation of cell lineages, we selected cell lineage marker genes exhibiting an average log2 fold-change > 0.5, adjusted p value < 0.01, and minimum difference in the fraction of detection between the two groups (pct. 1 minus pct. 2) > 0.2. The expression profile and clinical matrix of ESCC were downloaded from TCGA, and 97 samples from patients who did not undergoing neoadjuvant therapy were obtained. We then applied ssGSEA to impute the enrichment scores of specific gene signatures based on TCGA. For the scRNA-seq validation expression profile, we first evaluated the CNVs to identify tumor cells, after which the enrichment scores for specific gene signatures were determined using the “AddModuleScore” function.

**Multiplex immunohistochemistry**

Tissue sections from 8 patients with ESCC who underwent chemotherapy and immunotherapy were subjected to multiplex immunohistochemistry (mIHC) at West China Hospital of Sichuan University. Each patient provided both pre-therapy endoscopic biopsies and post-therapy surgical specimens. The samples were incubated with the four antibodies: Anti-CYP4F3 (bioss, bs-14160R), Anti-OSGIN1 (bioss, bs-5723R), Anti-MAFG (bioss, bs-18615R), Anti-NFE2L2 (abcam, ab62352). Single-staining tissue image of each reagent were captured and then merged by ImageJ software. Subsequently, we manually assessed the fluorescence area and identified the total and positive cells in each sample.

**Supplementary figures**


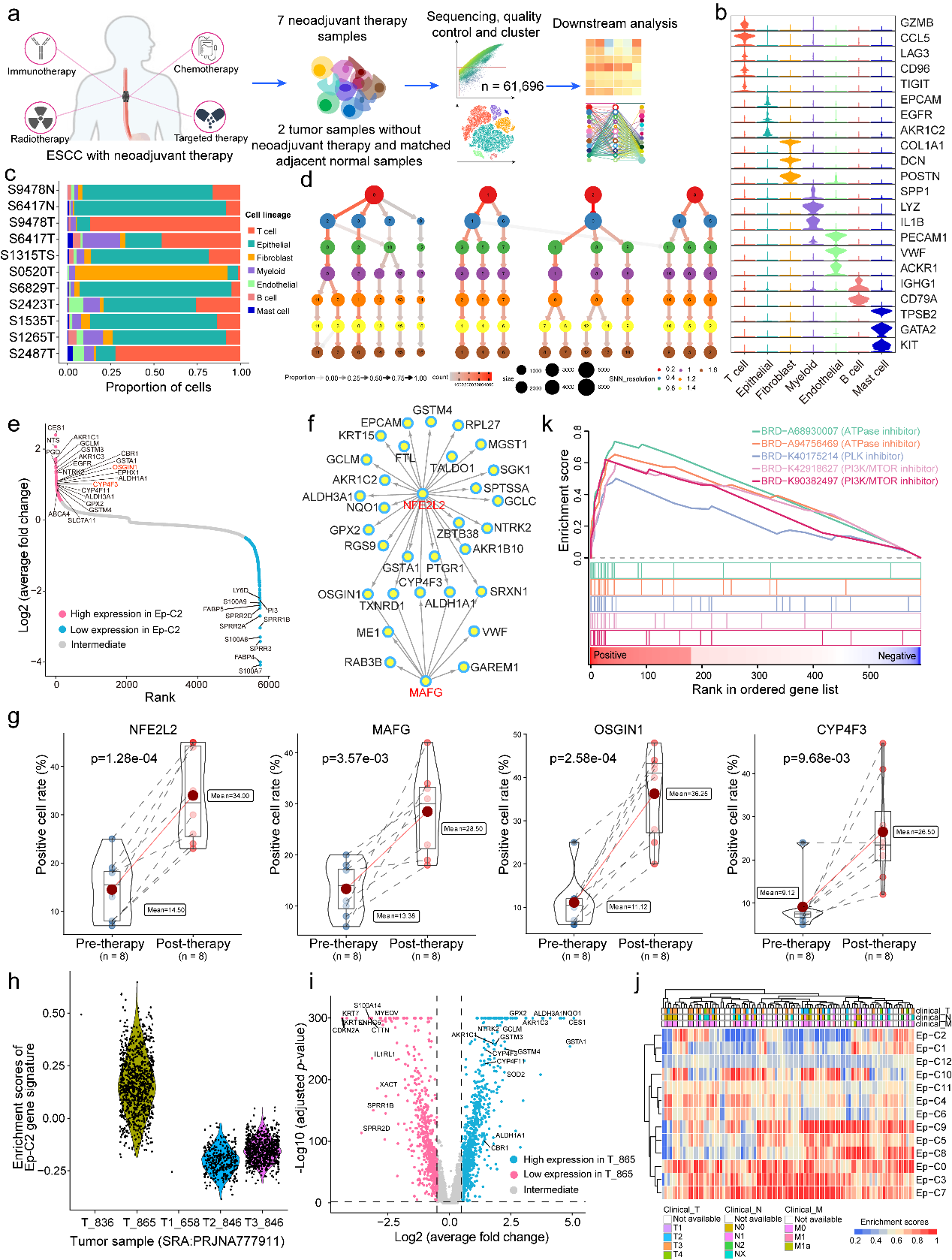


Supplementary Fig. 1. a. Overview of sample collection, experimental design, and analysis. b. Violin plot showing the expression levels of marker genes used to assign cell identity. c. Proportions of cells that contributed to each sample, colored according to cell lineage. d. Cluster tree shows the result of “FindCluster” among malignant epithelial cells, the robustness resolution was selected with sub-clusters without obvious interleaving, that is 0.8. e. Rank order of differentially expressed genes based on average log2 fold-change between Ep-C2 lineage and all other tumor cell lineages. f. Regulatory network of regulon *MAFG* (10 target genes) and *NFE2L2* (27 target genes), estimated using SCENIC. g. A combined box and violin plots showed the positive cell rates for multiplex immunohistochemistry of *NFE2L2*, *MAFG*, *OSGIN1*, and *CYP4F3* on 8 patients with neoadjuvant therapy. h. The enrichment scores of the Ep-C2 gene signatures in ESCC samples from another cohort. i. Volcano plots showed differentially expressed genes of ESCC patient T_865 from Fig. S1h. j. Heatmap shows the tumor cell lineages enrichment score in ESCC bulk transcriptome data from TCGA. k. GSEA plot shows the top 5 drugs with potential inhibition effect on Ep-C2 lineage.


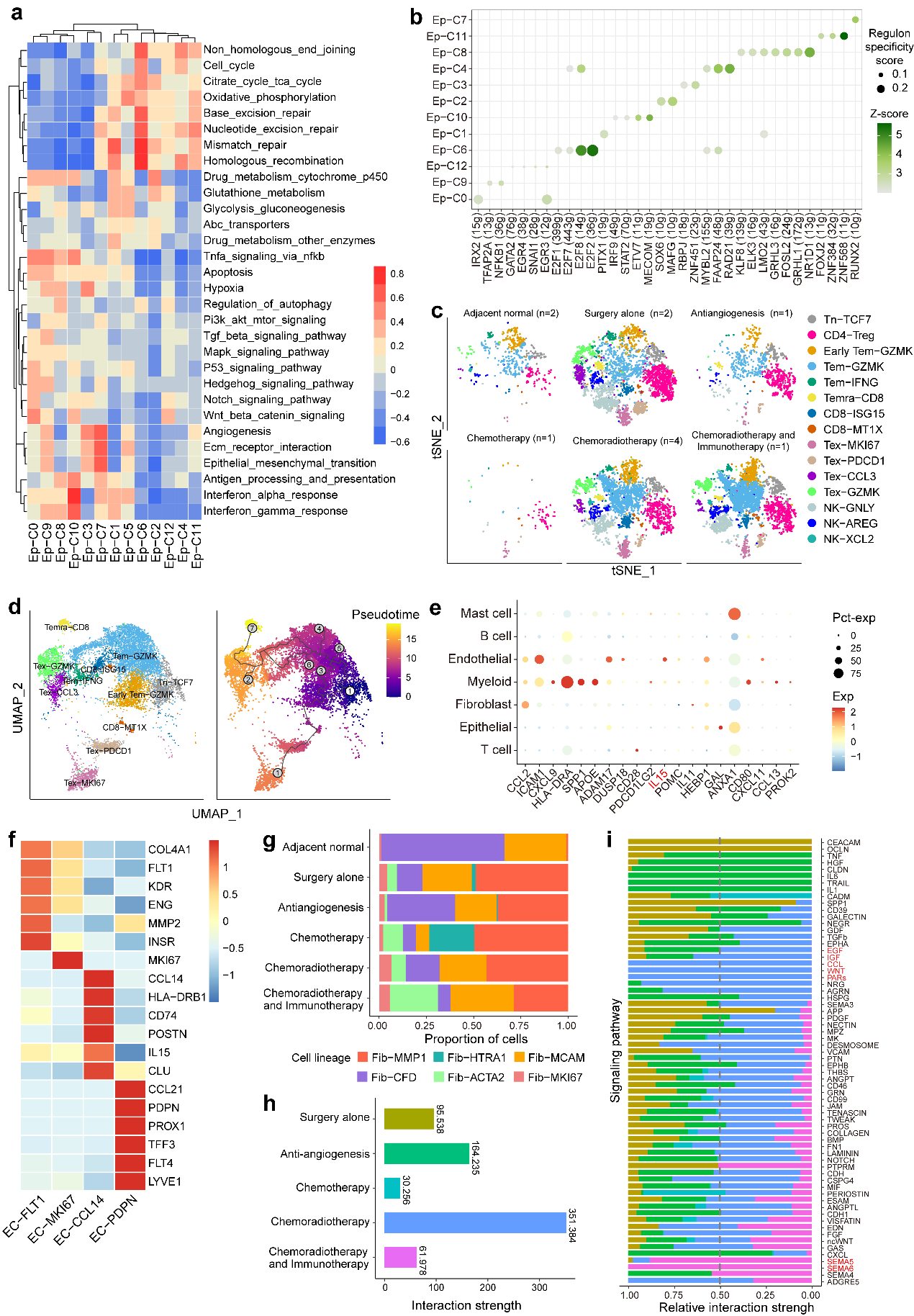


Supplementary Fig. 2. a. Heatmap shows GSVA enrichment scores of tumor cell lineages based on different pathways. b. Heatmap of the regulon specificity score and Z-score in each malignant epithelial sub-population, as estimated using SCENIC tool. c. t-SNE plots show the distribution of T cell lineages under different neoadjuvant therapy. Tn: naïve T cells; Treg: regulatory T cells; Tem: effector memory T cells; Tex: exhausted T cells; Temra: terminally differentiated effector memory or effector; NK: natural killer. d. UMAP plot shows the evolutionary trajectory of *CD8*^+^ T cells in a two-dimensional state space inferred by Monocle 3. e. Dot plot shows the average expression levels (Exp) and expression percentage (Pct-exp) of prioritized ligands (from Fig. 1l) in major cell lineages. f. Marker gene expression in endothelial cell lineages. g. Proportions of fibroblast lineages in different therapy strategies. h. Bar plot shows the total interaction strength of cell-cell communication networks in each therapy strategy. i. Relative interaction strength of each signaling pathway in cell-cell communication networks according to different therapies (the color refers to Supplementary Fig. 2h).

**Reference**

1 Germain, P. L., Lun, A., Garcia Meixide, C., Macnair, W. & Robinson, M. D. Doublet identification in single-cell sequencing data using scDblFinder. *F1000Res* **10**, 979 (2021).

2 Stuart, T. *et al.* Comprehensive Integration of Single-Cell Data. *Cell* **177**, 1888-1902.e21 (2019).

3 Korsunsky, I. *et al.* Fast, sensitive and accurate integration of single-cell data with Harmony. *Nat Methods* **16**, 1289-1296 (2019).

4 Cao, J. *et al.* The single-cell transcriptional landscape of mammalian organogenesis. *Nature* **566**, 496-502 (2019).

5 Jin, S. *et al.* Inference and analysis of cell-cell communication using CellChat. *Nat Commun* **12**, 1088 (2021).

6 Gao, R. *et al.* Delineating copy number and clonal substructure in human tumors from single-cell transcriptomes. *Nat Biotechnol* **39**, 599-608, (2021).

7 Aibar, S. *et al.* SCENIC: single-cell regulatory network inference and clustering. *Nat Methods* **14**, 1083-1086 (2017).

| **Supplementary Table 1 Clinical information of samples** | | | | | | | | | | | | |
| --- | --- | --- | --- | --- | --- | --- | --- | --- | --- | --- | --- | --- |
| Sample ID | Tissue type | Neoadjuvant therapy | Clinical G | Clinical T | Clinical N | Clinical M | Pathological response | Metastatic | Diagnosis metastatic time | Metastatic sites | Therapeutic regimen | Drug |
| S0520T | Tumor | Chemotherapy | G3 | T3 | N2 | M0 | TRS2 | Yes | 2021/2/23 | Bone | Chemotherapy | 5-fluorouracil and oxaliplatin |
| S1265T | Tumor | Chemoradiotherapy | G2 | T3 | N0 | M0 | TRS2 | No | - | - | Chemotherapy, radiotherapy, surgery | Paclitaxel and nedaplatin |
| S1315TS | Tumor | Anti-angiogenesis | G3 | T1b | N1 | M0 | TRS3 | Yes | 2021/1/27 | Hypopharynx | Chemotherapy, surgery | apatinib |
| S1535T | Tumor | Chemoradiotherapy | G3 | T3 | N1 | M0 | TRS2 | Yes | 2021/4/27 | Bone | Chemotherapy, radiotherapy, chemotherapy, surgery | Paclitaxel and carboplatin |
| S2423T | Tumor | Chemoradiotherapy | G3 | T3 | N3 | M0 | TRS2 | Yes | 2020/3/11 | Lymph node | Chemotherapy, radiotherapy, surgery | Paclitaxel and cisplatin |
| S2487T | Tumor | Chemoradiotherapy and Immunotherapy | G1 | T1a | N0 | M0 | TRS1 | Yes | 2020/3/23 | Ribs and liver | Chemoradiotherapy and Immunotherapy, radiotherapy, Chemoradiotherapy and Immunotherapy, surgery | Paclitaxel and Capecitabine and Tislelizumab |
| S6829T | Tumor | Chemoradiotherapy | G3 | T3 | N2 | M0 | TRS2 | Yes | 2020/5/19 | The whole body | Chemotherapy, radiotherapy, chemotherapy, surgery | Paclitaxel and nedaplatin |
| S6417N | Adjacent normal | Adjacent normal | Gx | Tx | Nx | Mx | - | No | - | - | - | - |
| S6417T | Tumor | Surgery alone | G2 | T1b | N2 | M0 | - | No | - | - | Surgery | - |
| S9478N | Adjacent normal | Adjacent normal | Gx | Tx | Nx | Mx | - | No | - | - | - | - |
| S9478T | Tumor | Surgery alone | G2 | T3 | N0 | M0 | - | No | - | - | Surgery | - |

Pathological response grading：

TRS3: non-pathological response, the tumor tissue poorly subsided with large number of residual tumor cells.

TRS2: pathological partial response, the tumor tissue slightly subsided and visible residual tumor cells.

TRS1: pathological basic response, the tumor tissue has basically subsided and few residual tumor cells in microscope.

TRS0: Pathological complete response, the tumor tissue has completely subsided and no residual tumor cells.

| **Supplementary Table 2 Different expression genes of each malignant epithelial lineages** | | | | | | |
| --- | --- | --- | --- | --- | --- | --- |
| Gene | Average log2FC | pct.1 | pct.2 | *p* value | *p* value- adjust | Cluster |
| KRT15 | 1.844 | 0.985 | 0.846 | 0 | 0 | Ep-C0 |
| HES1 | 1.398 | 0.987 | 0.845 | 0 | 0 | Ep-C0 |
| BTG2 | 1.232 | 0.961 | 0.776 | 0 | 0 | Ep-C0 |
| TSC22D1 | 1.173 | 0.944 | 0.764 | 0 | 0 | Ep-C0 |
| FOSB | 1.155 | 0.984 | 0.873 | 0 | 0 | Ep-C0 |
| JUNB | 1.132 | 1 | 0.97 | 0 | 0 | Ep-C0 |
| ZFP36L2 | 1.063 | 0.887 | 0.692 | 0 | 0 | Ep-C0 |
| FOS | 1.053 | 0.997 | 0.944 | 0 | 0 | Ep-C0 |
| NR4A1 | 1.051 | 0.872 | 0.59 | 0 | 0 | Ep-C0 |
| IER2 | 1.039 | 0.998 | 0.944 | 0 | 0 | Ep-C0 |
| SOX4 | 1.034 | 0.963 | 0.82 | 0 | 0 | Ep-C0 |
| JUN | 1.024 | 0.996 | 0.956 | 0 | 0 | Ep-C0 |
| JUND | 1.005 | 1 | 0.994 | 0 | 0 | Ep-C0 |
| MYC | 1.003 | 0.966 | 0.825 | 0 | 0 | Ep-C0 |
| NRARP | 0.994 | 0.986 | 0.915 | 0 | 0 | Ep-C0 |
| ZFP36 | 0.991 | 0.989 | 0.853 | 0 | 0 | Ep-C0 |
| EGR1 | 0.938 | 0.964 | 0.787 | 0 | 0 | Ep-C0 |
| PPP1R15A | 0.905 | 0.993 | 0.922 | 0 | 0 | Ep-C0 |
| SERTAD1 | 0.883 | 0.947 | 0.792 | 0 | 0 | Ep-C0 |
| ZFP36L1 | 0.832 | 0.99 | 0.945 | 0 | 0 | Ep-C0 |
| CCNL1 | 0.757 | 1 | 0.983 | 0 | 0 | Ep-C0 |
| BTG1 | 0.747 | 1 | 0.983 | 0 | 0 | Ep-C0 |
| PNRC1 | 0.713 | 0.994 | 0.912 | 5.43472210425371e-323 | 1.39297362254127e-318 | Ep-C0 |
| CXCL14 | 1.616 | 0.855 | 0.599 | 8.55E-305 | 2.19E-300 | Ep-C0 |
| DUSP1 | 0.903 | 0.986 | 0.887 | 9.79E-305 | 2.51E-300 | Ep-C0 |
| ATF3 | 1.015 | 0.937 | 0.739 | 1.05E-300 | 2.69E-296 | Ep-C0 |
| GEM | 1.027 | 0.41 | 0.164 | 2.59E-293 | 6.63E-289 | Ep-C0 |
| IER3 | 0.897 | 0.998 | 0.965 | 1.06E-291 | 2.71E-287 | Ep-C0 |
| RHOB | 0.938 | 0.939 | 0.768 | 2.18E-290 | 5.59E-286 | Ep-C0 |
| EGR3 | 0.905 | 0.644 | 0.354 | 1.61E-288 | 4.12E-284 | Ep-C0 |
| MIDN | 0.696 | 0.953 | 0.84 | 1.03E-279 | 2.65E-275 | Ep-C0 |
| MARCKSL1 | 0.776 | 0.957 | 0.846 | 2.28E-269 | 5.83E-265 | Ep-C0 |
| SOCS3 | 1.004 | 0.878 | 0.691 | 1.95E-266 | 4.99E-262 | Ep-C0 |
| TIPARP | 0.863 | 0.828 | 0.653 | 9.17E-261 | 2.35E-256 | Ep-C0 |
| IFRD1 | 0.711 | 0.811 | 0.615 | 1.50E-257 | 3.85E-253 | Ep-C0 |
| TGIF1 | 0.714 | 0.899 | 0.71 | 4.40E-257 | 1.13E-252 | Ep-C0 |
| ETS2 | 0.756 | 0.91 | 0.78 | 2.58E-246 | 6.61E-242 | Ep-C0 |
| BRD2 | 0.627 | 0.972 | 0.901 | 1.42E-243 | 3.63E-239 | Ep-C0 |
| TOB1 | 0.698 | 0.887 | 0.72 | 1.92E-242 | 4.93E-238 | Ep-C0 |
| KLF4 | 0.714 | 0.955 | 0.795 | 1.23E-241 | 3.16E-237 | Ep-C0 |
| NCOA7 | 0.853 | 0.95 | 0.791 | 1.39E-239 | 3.57E-235 | Ep-C0 |
| RND3 | 0.821 | 0.943 | 0.787 | 3.42E-237 | 8.75E-233 | Ep-C0 |
| KLF6 | 0.867 | 0.916 | 0.762 | 6.84E-233 | 1.75E-228 | Ep-C0 |
| CEBPD | 0.725 | 0.998 | 0.969 | 3.83E-230 | 9.83E-226 | Ep-C0 |
| UBC | 0.538 | 1 | 1 | 6.82E-230 | 1.75E-225 | Ep-C0 |
| DNAJA1 | 1.011 | 0.986 | 0.94 | 5.13E-228 | 1.31E-223 | Ep-C0 |
| TXNIP | 0.917 | 0.762 | 0.525 | 5.19E-228 | 1.33E-223 | Ep-C0 |
| ID3 | 1 | 0.665 | 0.422 | 1.34E-221 | 3.43E-217 | Ep-C0 |
| HOPX | 1.058 | 0.643 | 0.432 | 7.66E-215 | 1.96E-210 | Ep-C0 |
| YPEL5 | 0.582 | 0.903 | 0.763 | 6.09E-213 | 1.56E-208 | Ep-C0 |
| SNHG8 | 0.632 | 0.968 | 0.912 | 1.87E-211 | 4.79E-207 | Ep-C0 |
| PMAIP1 | 0.592 | 0.95 | 0.796 | 8.41E-200 | 2.16E-195 | Ep-C0 |
| CHMP1B | 0.539 | 0.916 | 0.808 | 1.46E-197 | 3.75E-193 | Ep-C0 |
| ABCC5 | 0.625 | 0.897 | 0.758 | 9.64E-191 | 2.47E-186 | Ep-C0 |
| CDKN1C | 0.88 | 0.561 | 0.365 | 9.43E-182 | 2.42E-177 | Ep-C0 |
| GAS5 | 0.642 | 0.997 | 0.986 | 1.66E-180 | 4.25E-176 | Ep-C0 |
| KDM6B | 0.584 | 0.72 | 0.535 | 1.90E-177 | 4.87E-173 | Ep-C0 |
| SCPEP1 | 0.657 | 0.962 | 0.906 | 1.32E-171 | 3.39E-167 | Ep-C0 |
| MAFF | 0.636 | 0.778 | 0.619 | 1.25E-170 | 3.19E-166 | Ep-C0 |
| PIM3 | 0.58 | 0.933 | 0.836 | 2.02E-170 | 5.17E-166 | Ep-C0 |
| SNRPN | 0.576 | 0.542 | 0.307 | 1.35E-168 | 3.46E-164 | Ep-C0 |
| NTRK2 | 0.564 | 0.969 | 0.821 | 5.01E-166 | 1.29E-161 | Ep-C0 |
| WSB1 | 0.535 | 0.928 | 0.821 | 1.62E-165 | 4.15E-161 | Ep-C0 |
| IRF2BP2 | 0.54 | 0.926 | 0.845 | 3.79E-165 | 9.72E-161 | Ep-C0 |
| MIR22HG | 0.599 | 0.579 | 0.362 | 4.12E-165 | 1.06E-160 | Ep-C0 |
| ALDH3A1 | 0.567 | 0.948 | 0.755 | 8.66E-165 | 2.22E-160 | Ep-C0 |
| REL | 0.511 | 0.912 | 0.808 | 9.08E-163 | 2.33E-158 | Ep-C0 |
| IER5L | 0.756 | 0.786 | 0.633 | 5.28E-159 | 1.35E-154 | Ep-C0 |
| ID2 | 0.858 | 0.505 | 0.311 | 9.60E-159 | 2.46E-154 | Ep-C0 |
| AMD1 | 0.518 | 0.926 | 0.846 | 1.39E-158 | 3.57E-154 | Ep-C0 |
| FAM133B | 0.509 | 0.872 | 0.802 | 7.28E-156 | 1.87E-151 | Ep-C0 |
| MAFB | 0.641 | 0.796 | 0.603 | 3.30E-155 | 8.46E-151 | Ep-C0 |
| GPNMB | 0.617 | 0.957 | 0.826 | 7.11E-155 | 1.82E-150 | Ep-C0 |
| DNAJB1 | 0.701 | 0.991 | 0.945 | 8.85E-154 | 2.27E-149 | Ep-C0 |
| EFNA1 | 0.525 | 0.945 | 0.828 | 1.25E-151 | 3.21E-147 | Ep-C0 |
| ZC3H12A | 0.546 | 0.827 | 0.657 | 2.49E-148 | 6.38E-144 | Ep-C0 |
| CCN1 | 0.715 | 0.428 | 0.237 | 3.09E-148 | 7.91E-144 | Ep-C0 |
| NFKBIA | 0.503 | 0.997 | 0.965 | 1.91E-147 | 4.89E-143 | Ep-C0 |
| ESRG | 0.509 | 0.278 | 0.102 | 1.47E-145 | 3.78E-141 | Ep-C0 |
| SLC25A25 | 0.592 | 0.551 | 0.358 | 1.84E-145 | 4.73E-141 | Ep-C0 |
| TNFAIP3 | 0.601 | 0.871 | 0.682 | 9.42E-144 | 2.41E-139 | Ep-C0 |
| IER5 | 0.509 | 0.928 | 0.775 | 3.52E-143 | 9.03E-139 | Ep-C0 |
| IRS2 | 0.561 | 0.631 | 0.436 | 1.08E-142 | 2.78E-138 | Ep-C0 |
| AATK | 0.538 | 0.444 | 0.288 | 2.12E-142 | 5.44E-138 | Ep-C0 |
| ARID5B | 0.572 | 0.928 | 0.808 | 5.81E-142 | 1.49E-137 | Ep-C0 |
| TUBA1A | 0.767 | 0.578 | 0.406 | 1.81E-141 | 4.63E-137 | Ep-C0 |
| ZFAND5 | 0.51 | 0.701 | 0.683 | 3.34E-141 | 8.57E-137 | Ep-C0 |
| RAP2B | 0.548 | 0.955 | 0.887 | 1.44E-139 | 3.69E-135 | Ep-C0 |
| NFE2L2 | 0.559 | 0.999 | 0.981 | 6.65E-137 | 1.70E-132 | Ep-C0 |
| DST | 0.558 | 0.962 | 0.856 | 8.89E-135 | 2.28E-130 | Ep-C0 |
| PLK2 | 0.558 | 0.594 | 0.425 | 9.13E-134 | 2.34E-129 | Ep-C0 |
| TRIB1 | 0.603 | 0.734 | 0.539 | 3.03E-133 | 7.76E-129 | Ep-C0 |
| ITPKC | 0.547 | 0.692 | 0.542 | 3.56E-127 | 9.12E-123 | Ep-C0 |
| GADD45A | 0.597 | 0.835 | 0.667 | 1.44E-126 | 3.68E-122 | Ep-C0 |
| KLF10 | 0.55 | 0.838 | 0.688 | 1.78E-125 | 4.57E-121 | Ep-C0 |
| TUBB2A | 0.58 | 0.856 | 0.699 | 7.25E-117 | 1.86E-112 | Ep-C0 |
| IRF1 | 0.507 | 0.883 | 0.752 | 6.18E-116 | 1.58E-111 | Ep-C0 |
| FOXQ1 | 0.641 | 0.565 | 0.414 | 4.71E-115 | 1.21E-110 | Ep-C0 |
| THSD4 | 0.537 | 0.361 | 0.201 | 5.90E-115 | 1.51E-110 | Ep-C0 |
| LIME1 | 0.53 | 0.432 | 0.27 | 4.49E-113 | 1.15E-108 | Ep-C0 |
| RPS4X | 0.512 | 1 | 0.999 | 2.04E-107 | 5.22E-103 | Ep-C0 |
| GADD45B | 0.649 | 0.803 | 0.682 | 1.15E-104 | 2.95E-100 | Ep-C0 |
| ZNF131 | 0.654 | 0.828 | 0.708 | 4.15E-101 | 1.06E-96 | Ep-C0 |
| DUSP2 | 0.708 | 0.585 | 0.406 | 1.75E-98 | 4.47E-94 | Ep-C0 |
| ADM | 0.564 | 0.878 | 0.72 | 4.86E-97 | 1.24E-92 | Ep-C0 |
| FAM43A | 0.518 | 0.493 | 0.335 | 6.95E-95 | 1.78E-90 | Ep-C0 |
| MALAT1 | 0.519 | 1 | 1 | 1.81E-94 | 4.65E-90 | Ep-C0 |
| KRTAP3-1 | 0.827 | 0.153 | 0.046 | 3.89E-89 | 9.96E-85 | Ep-C0 |
| NFKBIZ | 0.536 | 0.839 | 0.717 | 2.23E-86 | 5.71E-82 | Ep-C0 |
| RASD1 | 0.537 | 0.442 | 0.294 | 3.77E-82 | 9.67E-78 | Ep-C0 |
| BAG1 | 0.593 | 0.946 | 0.886 | 7.94E-82 | 2.04E-77 | Ep-C0 |
| CXCL2 | 0.785 | 0.376 | 0.24 | 4.20E-81 | 1.08E-76 | Ep-C0 |
| IL1R2 | 0.679 | 0.34 | 0.203 | 1.88E-76 | 4.81E-72 | Ep-C0 |
| TUBB2B | 0.634 | 0.207 | 0.09 | 6.62E-72 | 1.70E-67 | Ep-C0 |
| KCNQ1OT1 | 0.504 | 0.512 | 0.406 | 2.11E-56 | 5.41E-52 | Ep-C0 |
| MRPS30 | 0.525 | 0.829 | 0.737 | 2.76E-44 | 7.06E-40 | Ep-C0 |
| HSPA1A | 0.636 | 0.989 | 0.959 | 1.80E-42 | 4.60E-38 | Ep-C0 |
| HSPA1B | 0.51 | 0.882 | 0.816 | 4.66E-23 | 1.19E-18 | Ep-C0 |
| CCL20 | 2.746 | 0.832 | 0.508 | 0 | 0 | Ep-C1 |
| S100A9 | 2.084 | 0.998 | 0.913 | 0 | 0 | Ep-C1 |
| S100A8 | 1.769 | 0.996 | 0.848 | 0 | 0 | Ep-C1 |
| LCN2 | 1.598 | 0.933 | 0.554 | 0 | 0 | Ep-C1 |
| CXCL8 | 1.443 | 0.549 | 0.333 | 1.15E-170 | 2.94E-166 | Ep-C1 |
| SEC61G | 1.424 | 0.999 | 0.997 | 6.22E-252 | 1.59E-247 | Ep-C1 |
| SPTSSA | 1.343 | 0.923 | 0.859 | 0 | 0 | Ep-C1 |
| TNFAIP2 | 1.257 | 0.878 | 0.667 | 1.08E-304 | 2.77E-300 | Ep-C1 |
| NFKBIA | 1.254 | 0.993 | 0.966 | 4.13E-256 | 1.06E-251 | Ep-C1 |
| DEFB1 | 1.227 | 0.682 | 0.331 | 2.60E-296 | 6.66E-292 | Ep-C1 |
| CHCHD2 | 1.213 | 1 | 1 | 0 | 0 | Ep-C1 |
| PDZK1IP1 | 1.202 | 0.883 | 0.415 | 0 | 0 | Ep-C1 |
| CXCL1 | 1.158 | 0.474 | 0.336 | 3.35E-83 | 8.60E-79 | Ep-C1 |
| CCT6A | 1.132 | 0.992 | 0.982 | 1.89E-286 | 4.84E-282 | Ep-C1 |
| SOD2 | 1.117 | 0.988 | 0.943 | 0 | 0 | Ep-C1 |
| GAL | 1.08 | 0.634 | 0.363 | 5.61E-203 | 1.44E-198 | Ep-C1 |
| NUDT8 | 1.053 | 0.887 | 0.686 | 0 | 0 | Ep-C1 |
| GSTP1 | 1.031 | 1 | 1 | 0 | 0 | Ep-C1 |
| HLA-DRA | 1.03 | 0.509 | 0.237 | 4.92E-180 | 1.26E-175 | Ep-C1 |
| SUMF2 | 1.015 | 0.791 | 0.707 | 1.29E-239 | 3.31E-235 | Ep-C1 |
| NIPSNAP2 | 1.006 | 0.864 | 0.804 | 7.24E-228 | 1.86E-223 | Ep-C1 |
| GSTA1 | 0.995 | 0.483 | 0.226 | 2.01E-139 | 5.15E-135 | Ep-C1 |
| EGFR | 0.993 | 0.885 | 0.861 | 8.47E-163 | 2.17E-158 | Ep-C1 |
| NTS | 0.99 | 0.666 | 0.435 | 1.88E-159 | 4.81E-155 | Ep-C1 |
| GCLM | 0.979 | 0.931 | 0.755 | 3.75E-260 | 9.62E-256 | Ep-C1 |
| GLUL | 0.968 | 0.996 | 0.966 | 0 | 0 | Ep-C1 |
| NAMPT | 0.946 | 0.955 | 0.881 | 8.68E-183 | 2.23E-178 | Ep-C1 |
| PITX1 | 0.906 | 0.992 | 0.943 | 0 | 0 | Ep-C1 |
| S100A7 | 0.903 | 0.764 | 0.554 | 1.02E-186 | 2.62E-182 | Ep-C1 |
| PSMA6 | 0.882 | 0.999 | 0.994 | 1.14E-279 | 2.92E-275 | Ep-C1 |
| LYN | 0.832 | 0.682 | 0.277 | 0 | 0 | Ep-C1 |
| CXCL17 | 0.83 | 0.475 | 0.144 | 4.19E-258 | 1.07E-253 | Ep-C1 |
| LINC01133 | 0.822 | 0.854 | 0.454 | 0 | 0 | Ep-C1 |
| NCOA7 | 0.796 | 0.971 | 0.79 | 1.46E-271 | 3.74E-267 | Ep-C1 |
| SERPINB4 | 0.793 | 0.408 | 0.365 | 2.21E-51 | 5.66E-47 | Ep-C1 |
| CPT1A | 0.782 | 0.57 | 0.3 | 2.11E-188 | 5.40E-184 | Ep-C1 |
| MUC4 | 0.778 | 0.604 | 0.185 | 0 | 0 | Ep-C1 |
| PSPH | 0.774 | 0.485 | 0.267 | 1.55E-180 | 3.98E-176 | Ep-C1 |
| MRPS17 | 0.759 | 0.888 | 0.749 | 9.15E-230 | 2.35E-225 | Ep-C1 |
| NDUFB11 | 0.758 | 0.993 | 0.991 | 7.74E-228 | 1.98E-223 | Ep-C1 |
| EHF | 0.75 | 0.937 | 0.648 | 2.9742751879643e-321 | 7.62336473427131e-317 | Ep-C1 |
| ADH1C | 0.745 | 0.386 | 0.122 | 7.64E-187 | 1.96E-182 | Ep-C1 |
| HILPDA | 0.739 | 0.649 | 0.404 | 9.07E-132 | 2.32E-127 | Ep-C1 |
| NDUFS8 | 0.738 | 0.982 | 0.964 | 1.09E-202 | 2.78E-198 | Ep-C1 |
| PTGR1 | 0.735 | 0.954 | 0.763 | 6.53E-241 | 1.67E-236 | Ep-C1 |
| SAT1 | 0.73 | 0.998 | 0.987 | 1.81E-167 | 4.65E-163 | Ep-C1 |
| TACSTD2 | 0.718 | 0.996 | 0.93 | 4.55E-235 | 1.17E-230 | Ep-C1 |
| AL445524.1 | 0.717 | 0.606 | 0.311 | 9.16E-215 | 2.35E-210 | Ep-C1 |
| CDK2AP2 | 0.715 | 0.831 | 0.732 | 4.96E-190 | 1.27E-185 | Ep-C1 |
| PGD | 0.705 | 0.931 | 0.788 | 1.08E-191 | 2.76E-187 | Ep-C1 |
| GBP1 | 0.702 | 0.611 | 0.393 | 5.26E-119 | 1.35E-114 | Ep-C1 |
| CRYAB | 0.7 | 0.7 | 0.412 | 7.89E-155 | 2.02E-150 | Ep-C1 |
| NFKBIZ | 0.696 | 0.874 | 0.714 | 3.13E-168 | 8.03E-164 | Ep-C1 |
| ASS1 | 0.689 | 0.674 | 0.335 | 3.03E-231 | 7.76E-227 | Ep-C1 |
| MGST1 | 0.677 | 0.955 | 0.795 | 1.74E-212 | 4.47E-208 | Ep-C1 |
| MRPL11 | 0.673 | 0.953 | 0.906 | 3.30E-189 | 8.47E-185 | Ep-C1 |
| RNASEH2C | 0.671 | 0.817 | 0.741 | 1.78E-153 | 4.57E-149 | Ep-C1 |
| EDN2 | 0.666 | 0.249 | 0.121 | 1.12E-54 | 2.87E-50 | Ep-C1 |
| SGK1 | 0.654 | 0.971 | 0.885 | 2.25E-143 | 5.77E-139 | Ep-C1 |
| COTL1 | 0.645 | 0.779 | 0.529 | 2.59E-186 | 6.64E-182 | Ep-C1 |
| STEAP1 | 0.641 | 0.771 | 0.525 | 5.92E-180 | 1.52E-175 | Ep-C1 |
| SAA1 | 0.641 | 0.501 | 0.365 | 3.06E-40 | 7.84E-36 | Ep-C1 |
| PLAT | 0.631 | 0.758 | 0.599 | 1.92E-84 | 4.91E-80 | Ep-C1 |
| RPS4Y1 | 0.63 | 0.643 | 0.687 | 4.97E-90 | 1.27E-85 | Ep-C1 |
| NDUFV1 | 0.627 | 0.96 | 0.929 | 7.15E-180 | 1.83E-175 | Ep-C1 |
| AKR1B10 | 0.613 | 0.881 | 0.672 | 8.42E-141 | 2.16E-136 | Ep-C1 |
| BBC3 | 0.611 | 0.666 | 0.393 | 3.03E-159 | 7.76E-155 | Ep-C1 |
| VOPP1 | 0.609 | 0.637 | 0.488 | 2.67E-158 | 6.84E-154 | Ep-C1 |
| CES1 | 0.606 | 0.48 | 0.232 | 1.06E-123 | 2.71E-119 | Ep-C1 |
| FTL | 0.605 | 1 | 1 | 1.78E-192 | 4.56E-188 | Ep-C1 |
| FIBP | 0.6 | 0.948 | 0.874 | 7.58E-201 | 1.94E-196 | Ep-C1 |
| TMPRSS4 | 0.596 | 0.864 | 0.672 | 1.38E-160 | 3.55E-156 | Ep-C1 |
| EPB41L1 | 0.577 | 0.615 | 0.277 | 7.77E-224 | 1.99E-219 | Ep-C1 |
| RARRES1 | 0.574 | 0.34 | 0.115 | 4.97E-150 | 1.27E-145 | Ep-C1 |
| CEBPD | 0.573 | 0.994 | 0.97 | 9.15E-124 | 2.34E-119 | Ep-C1 |
| CSTA | 0.572 | 0.992 | 0.919 | 5.77E-226 | 1.48E-221 | Ep-C1 |
| TNIP3 | 0.564 | 0.283 | 0.062 | 6.23E-195 | 1.60E-190 | Ep-C1 |
| EPCAM | 0.562 | 0.929 | 0.831 | 7.00E-123 | 1.79E-118 | Ep-C1 |
| SRP54 | 0.558 | 0.781 | 0.674 | 1.83E-132 | 4.70E-128 | Ep-C1 |
| CLDN7 | 0.554 | 0.912 | 0.659 | 2.93E-188 | 7.52E-184 | Ep-C1 |
| CYB5A | 0.548 | 0.917 | 0.786 | 3.22E-153 | 8.26E-149 | Ep-C1 |
| SLC7A5 | 0.547 | 0.83 | 0.68 | 1.58E-122 | 4.05E-118 | Ep-C1 |
| PHGDH | 0.54 | 0.618 | 0.446 | 1.47E-89 | 3.76E-85 | Ep-C1 |
| CALML5 | 0.538 | 0.614 | 0.264 | 1.65E-229 | 4.24E-225 | Ep-C1 |
| KRT31 | 0.538 | 0.322 | 0.169 | 5.45E-74 | 1.40E-69 | Ep-C1 |
| BST2 | 0.533 | 0.572 | 0.402 | 5.62E-83 | 1.44E-78 | Ep-C1 |
| TMPRSS11A | 0.533 | 0.439 | 0.19 | 3.61E-136 | 9.26E-132 | Ep-C1 |
| FAM83A | 0.528 | 0.627 | 0.385 | 9.71E-110 | 2.49E-105 | Ep-C1 |
| NUCB2 | 0.52 | 0.805 | 0.729 | 2.80E-83 | 7.18E-79 | Ep-C1 |
| CCDC85B | 0.517 | 0.929 | 0.889 | 2.69E-103 | 6.89E-99 | Ep-C1 |
| SNX6 | 0.517 | 0.954 | 0.936 | 1.59E-112 | 4.07E-108 | Ep-C1 |
| NEAT1 | 0.514 | 0.999 | 0.999 | 1.32E-100 | 3.39E-96 | Ep-C1 |
| HCAR3 | 0.512 | 0.526 | 0.242 | 1.13E-156 | 2.91E-152 | Ep-C1 |
| MAL2 | 0.511 | 0.962 | 0.9 | 8.78E-144 | 2.25E-139 | Ep-C1 |
| CDKN2A | 0.51 | 0.991 | 0.953 | 3.48E-135 | 8.92E-131 | Ep-C1 |
| BAZ1A | 0.507 | 0.928 | 0.898 | 4.65E-127 | 1.19E-122 | Ep-C1 |
| HLA-DMA | 0.504 | 0.342 | 0.122 | 7.23E-138 | 1.85E-133 | Ep-C1 |
| ICAM1 | 0.504 | 0.422 | 0.25 | 6.38E-75 | 1.63E-70 | Ep-C1 |
| FAM3B | 0.503 | 0.654 | 0.327 | 1.04E-192 | 2.67E-188 | Ep-C1 |
| CFB | 0.502 | 0.422 | 0.178 | 1.93E-138 | 4.93E-134 | Ep-C1 |
| BLVRB | 0.5 | 0.992 | 0.967 | 7.34E-157 | 1.88E-152 | Ep-C1 |
| CES1 | 2.393 | 0.682 | 0.208 | 0 | 0 | Ep-C2 |
| NTS | 2.059 | 0.721 | 0.429 | 0 | 0 | Ep-C2 |
| AKR1C1 | 1.72 | 0.932 | 0.757 | 0 | 0 | Ep-C2 |
| EPCAM | 1.671 | 0.947 | 0.829 | 0 | 0 | Ep-C2 |
| GCLM | 1.606 | 0.917 | 0.757 | 0 | 0 | Ep-C2 |
| RPS4Y1 | 1.507 | 0.837 | 0.662 | 0 | 0 | Ep-C2 |
| PGD | 1.502 | 0.938 | 0.788 | 0 | 0 | Ep-C2 |
| GSTM3 | 1.461 | 0.946 | 0.808 | 0 | 0 | Ep-C2 |
| AKR1C3 | 1.374 | 0.977 | 0.85 | 0 | 0 | Ep-C2 |
| AL033397.1 | 1.345 | 0.65 | 0.189 | 0 | 0 | Ep-C2 |
| EGFR | 1.309 | 0.885 | 0.861 | 0 | 0 | Ep-C2 |
| NTRK2 | 1.228 | 0.969 | 0.823 | 0 | 0 | Ep-C2 |
| CBR1 | 1.086 | 0.984 | 0.934 | 0 | 0 | Ep-C2 |
| OSGIN1 | 1.081 | 0.649 | 0.267 | 0 | 0 | Ep-C2 |
| PART1 | 1.069 | 0.534 | 0.151 | 0 | 0 | Ep-C2 |
| EPHX1 | 1.058 | 0.878 | 0.667 | 0 | 0 | Ep-C2 |
| CYP4F3 | 1.029 | 0.54 | 0.156 | 0 | 0 | Ep-C2 |
| TCF4 | 1.005 | 0.785 | 0.499 | 0 | 0 | Ep-C2 |
| CYP4F11 | 0.987 | 0.599 | 0.238 | 0 | 0 | Ep-C2 |
| RPL23 | 0.974 | 1 | 1 | 0 | 0 | Ep-C2 |
| TKT | 0.891 | 1 | 0.993 | 0 | 0 | Ep-C2 |
| RPS20 | 0.886 | 1 | 0.999 | 0 | 0 | Ep-C2 |
| RPL5 | 0.859 | 1 | 1 | 0 | 0 | Ep-C2 |
| DANCR | 0.855 | 0.96 | 0.844 | 0 | 0 | Ep-C2 |
| PABPC1 | 0.816 | 1 | 1 | 0 | 0 | Ep-C2 |
| RPS13 | 0.816 | 1 | 1 | 0 | 0 | Ep-C2 |
| UXT | 0.795 | 0.978 | 0.928 | 0 | 0 | Ep-C2 |
| EIF3E | 0.781 | 1 | 0.994 | 0 | 0 | Ep-C2 |
| RPL13 | 0.691 | 1 | 1 | 0 | 0 | Ep-C2 |
| RPL7A | 0.689 | 1 | 1 | 0 | 0 | Ep-C2 |
| RPS25 | 0.679 | 1 | 1 | 0 | 0 | Ep-C2 |
| RPS11 | 0.668 | 1 | 1 | 0 | 0 | Ep-C2 |
| RPL18 | 0.655 | 1 | 1 | 0 | 0 | Ep-C2 |
| EEF1G | 0.644 | 1 | 1 | 0 | 0 | Ep-C2 |
| RPL8 | 0.643 | 1 | 1 | 0 | 0 | Ep-C2 |
| RPL24 | 0.642 | 1 | 1 | 0 | 0 | Ep-C2 |
| RPL38 | 0.601 | 1 | 1 | 0 | 0 | Ep-C2 |
| RPL15 | 0.593 | 1 | 1 | 0 | 0 | Ep-C2 |
| RPS19 | 0.565 | 1 | 1 | 0 | 0 | Ep-C2 |
| RPS27A | 0.538 | 1 | 1 | 0 | 0 | Ep-C2 |
| RPS15A | 0.535 | 1 | 1 | 0 | 0 | Ep-C2 |
| FAU | 0.528 | 1 | 1 | 0 | 0 | Ep-C2 |
| GSTM2 | 0.624 | 0.381 | 0.073 | 9.88131291682493e-324 | 2.5326793137114e-319 | Ep-C2 |
| RPL22 | 0.522 | 1 | 1 | 1.48219693752374e-323 | 3.7990189705671e-319 | Ep-C2 |
| RPL36A | 0.67 | 1 | 1 | 1.8626274848215e-321 | 4.77410050634599e-317 | Ep-C2 |
| LMO4 | 0.963 | 0.888 | 0.733 | 3.26083326255223e-321 | 8.35784173524761e-317 | Ep-C2 |
| RPS3A | 0.833 | 1 | 1 | 1.19030295396073e-319 | 3.05086550129675e-315 | Ep-C2 |
| ABCA4 | 0.897 | 0.453 | 0.107 | 2.33821339568509e-316 | 5.99307475448045e-312 | Ep-C2 |
| RPL17 | 0.669 | 1 | 1 | 2.62598629889266e-313 | 6.73066548269179e-309 | Ep-C2 |
| RPL12 | 0.564 | 1 | 1 | 5.93407711036989e-311 | 1.52E-306 | Ep-C2 |
| SEC61G | 1.342 | 0.999 | 0.997 | 1.51881979050782e-310 | 3.89E-306 | Ep-C2 |
| G6PD | 0.86 | 0.837 | 0.642 | 1.33655585911723e-309 | 3.43E-305 | Ep-C2 |
| RPL29 | 0.577 | 1 | 1 | 5.3877917396648e-309 | 1.38E-304 | Ep-C2 |
| RACK1 | 0.609 | 1 | 0.999 | 5.78221880668873e-309 | 1.48E-304 | Ep-C2 |
| ABCC1 | 0.775 | 0.74 | 0.48 | 9.38785296572276e-309 | 2.41E-304 | Ep-C2 |
| NIPSNAP2 | 1.002 | 0.918 | 0.798 | 7.99E-308 | 2.05E-303 | Ep-C2 |
| RPL30 | 0.579 | 1 | 1 | 3.08E-306 | 7.90E-302 | Ep-C2 |
| RPS17 | 0.606 | 1 | 0.999 | 4.77E-306 | 1.22E-301 | Ep-C2 |
| FTL | 0.956 | 1 | 1 | 2.87E-304 | 7.35E-300 | Ep-C2 |
| COA6 | 0.734 | 0.94 | 0.846 | 3.08E-301 | 7.89E-297 | Ep-C2 |
| GPX2 | 0.952 | 0.968 | 0.84 | 1.21E-295 | 3.11E-291 | Ep-C2 |
| NDUFB11 | 0.817 | 0.998 | 0.991 | 2.71E-293 | 6.95E-289 | Ep-C2 |
| SUMF2 | 0.973 | 0.866 | 0.698 | 1.00E-286 | 2.57E-282 | Ep-C2 |
| RPL23A | 0.56 | 1 | 1 | 4.08E-284 | 1.05E-279 | Ep-C2 |
| RPL14 | 0.516 | 1 | 1 | 6.71E-284 | 1.72E-279 | Ep-C2 |
| AP002387.2 | 0.843 | 0.6 | 0.293 | 1.34E-282 | 3.43E-278 | Ep-C2 |
| TRAPPC6A | 0.723 | 0.869 | 0.633 | 3.18E-282 | 8.15E-278 | Ep-C2 |
| RPL37A | 0.543 | 1 | 1 | 2.59E-281 | 6.64E-277 | Ep-C2 |
| RPS29 | 0.588 | 1 | 1 | 1.37E-280 | 3.51E-276 | Ep-C2 |
| NDUFS4 | 0.735 | 0.973 | 0.904 | 1.68E-276 | 4.30E-272 | Ep-C2 |
| SNHG7 | 0.799 | 0.94 | 0.855 | 4.62E-273 | 1.19E-268 | Ep-C2 |
| PTGR1 | 1.027 | 0.916 | 0.769 | 3.43E-272 | 8.80E-268 | Ep-C2 |
| RPL9 | 0.516 | 1 | 1 | 2.83E-270 | 7.24E-266 | Ep-C2 |
| SNHG19 | 0.729 | 0.709 | 0.385 | 2.53E-269 | 6.47E-265 | Ep-C2 |
| UQCRB | 0.513 | 1 | 1 | 2.91E-268 | 7.46E-264 | Ep-C2 |
| RPL27A | 0.555 | 1 | 1 | 5.86E-268 | 1.50E-263 | Ep-C2 |
| RPL3 | 0.612 | 1 | 1 | 1.52E-263 | 3.90E-259 | Ep-C2 |
| RPS5 | 0.563 | 1 | 1 | 1.91E-262 | 4.89E-258 | Ep-C2 |
| GSTM4 | 0.907 | 0.593 | 0.287 | 1.08E-261 | 2.77E-257 | Ep-C2 |
| ALDH1A1 | 1.036 | 0.886 | 0.673 | 1.56E-261 | 4.00E-257 | Ep-C2 |
| SLC7A11 | 0.844 | 0.576 | 0.249 | 1.82E-261 | 4.65E-257 | Ep-C2 |
| MXRA7 | 0.761 | 0.856 | 0.687 | 1.31E-259 | 3.36E-255 | Ep-C2 |
| EEF1D | 0.72 | 1 | 0.999 | 1.72E-258 | 4.40E-254 | Ep-C2 |
| RPS6 | 0.579 | 1 | 1 | 7.63E-253 | 1.95E-248 | Ep-C2 |
| SNX31 | 0.693 | 0.379 | 0.098 | 6.62E-246 | 1.70E-241 | Ep-C2 |
| DUXAP8 | 0.7 | 0.764 | 0.518 | 2.11E-244 | 5.40E-240 | Ep-C2 |
| MRPL11 | 0.737 | 0.966 | 0.904 | 4.26E-242 | 1.09E-237 | Ep-C2 |
| GSTM1 | 0.518 | 0.278 | 0.044 | 1.13E-241 | 2.89E-237 | Ep-C2 |
| NUCB2 | 0.87 | 0.865 | 0.722 | 7.78E-239 | 1.99E-234 | Ep-C2 |
| TALDO1 | 0.636 | 1 | 0.996 | 8.08E-239 | 2.07E-234 | Ep-C2 |
| CCT6A | 0.79 | 0.994 | 0.981 | 1.61E-233 | 4.14E-229 | Ep-C2 |
| NDUFV1 | 0.684 | 0.973 | 0.928 | 5.10E-232 | 1.31E-227 | Ep-C2 |
| STXBP6 | 0.901 | 0.456 | 0.158 | 1.36E-231 | 3.49E-227 | Ep-C2 |
| EIF3H | 0.524 | 0.999 | 0.994 | 2.40E-231 | 6.15E-227 | Ep-C2 |
| RPL13A | 0.646 | 1 | 1 | 3.49E-227 | 8.93E-223 | Ep-C2 |
| CEP68 | 0.723 | 0.681 | 0.416 | 1.54E-223 | 3.94E-219 | Ep-C2 |
| EAPP | 0.677 | 0.885 | 0.763 | 1.65E-221 | 4.22E-217 | Ep-C2 |
| NOP53 | 0.622 | 0.985 | 0.946 | 3.96E-221 | 1.01E-216 | Ep-C2 |
| SNX6 | 0.703 | 0.966 | 0.934 | 7.26E-220 | 1.86E-215 | Ep-C2 |
| LAMTOR4 | 0.541 | 0.996 | 0.98 | 1.38E-218 | 3.53E-214 | Ep-C2 |
| RPS2 | 0.534 | 1 | 1 | 2.50E-217 | 6.41E-213 | Ep-C2 |
| NAMPT | 0.856 | 0.937 | 0.884 | 3.93E-211 | 1.01E-206 | Ep-C2 |
| ADH7 | 0.707 | 0.838 | 0.572 | 1.98E-210 | 5.07E-206 | Ep-C2 |
| DRAM2 | 0.617 | 0.665 | 0.439 | 3.82E-207 | 9.80E-203 | Ep-C2 |
| SNHG29 | 0.516 | 1 | 0.999 | 1.02E-204 | 2.62E-200 | Ep-C2 |
| ALDH3A1 | 0.977 | 0.925 | 0.761 | 7.14E-204 | 1.83E-199 | Ep-C2 |
| APEX1 | 0.55 | 0.972 | 0.889 | 4.20E-203 | 1.08E-198 | Ep-C2 |
| NDUFS8 | 0.661 | 0.99 | 0.963 | 1.07E-202 | 2.74E-198 | Ep-C2 |
| RAB3B | 0.601 | 0.309 | 0.077 | 2.20E-202 | 5.64E-198 | Ep-C2 |
| NFE2L2 | 0.732 | 0.995 | 0.982 | 1.62E-201 | 4.14E-197 | Ep-C2 |
| AKR1B10 | 0.996 | 0.877 | 0.674 | 9.01E-200 | 2.31E-195 | Ep-C2 |
| CPT1A | 0.74 | 0.607 | 0.296 | 1.52E-199 | 3.88E-195 | Ep-C2 |
| EPS8 | 0.738 | 0.652 | 0.346 | 6.14E-198 | 1.57E-193 | Ep-C2 |
| PLAC8 | 0.825 | 0.406 | 0.13 | 3.60E-197 | 9.24E-193 | Ep-C2 |
| RBP7 | 0.665 | 0.597 | 0.3 | 5.06E-197 | 1.30E-192 | Ep-C2 |
| GSTA4 | 0.641 | 0.831 | 0.634 | 1.40E-196 | 3.60E-192 | Ep-C2 |
| CCT4 | 0.595 | 0.99 | 0.977 | 3.70E-194 | 9.49E-190 | Ep-C2 |
| CD99 | 0.738 | 0.955 | 0.939 | 3.44E-192 | 8.82E-188 | Ep-C2 |
| DCXR | 0.607 | 0.976 | 0.931 | 2.95E-188 | 7.57E-184 | Ep-C2 |
| POLD4 | 0.665 | 0.918 | 0.856 | 8.04E-187 | 2.06E-182 | Ep-C2 |
| TESMIN | 0.66 | 0.529 | 0.242 | 2.07E-186 | 5.31E-182 | Ep-C2 |
| RPL7 | 0.575 | 1 | 1 | 1.58E-185 | 4.05E-181 | Ep-C2 |
| BCL11A | 0.656 | 0.742 | 0.523 | 5.69E-185 | 1.46E-180 | Ep-C2 |
| PAK1 | 0.735 | 0.747 | 0.534 | 1.41E-184 | 3.60E-180 | Ep-C2 |
| RBM4 | 0.545 | 0.977 | 0.942 | 1.83E-184 | 4.70E-180 | Ep-C2 |
| RPL10 | 0.65 | 1 | 1 | 6.37E-181 | 1.63E-176 | Ep-C2 |
| MRPS33 | 0.534 | 0.919 | 0.81 | 1.39E-179 | 3.57E-175 | Ep-C2 |
| GSTA1 | 1.082 | 0.51 | 0.224 | 4.31E-177 | 1.10E-172 | Ep-C2 |
| CALML5 | 0.774 | 0.568 | 0.272 | 3.19E-174 | 8.18E-170 | Ep-C2 |
| SOX2 | 0.726 | 0.898 | 0.721 | 2.73E-172 | 7.01E-168 | Ep-C2 |
| CTSF | 0.564 | 0.518 | 0.244 | 4.13E-172 | 1.06E-167 | Ep-C2 |
| CHCHD2 | 0.641 | 1 | 1 | 5.45E-172 | 1.40E-167 | Ep-C2 |
| TMPRSS4 | 0.624 | 0.851 | 0.674 | 6.41E-171 | 1.64E-166 | Ep-C2 |
| SPTSSA | 0.755 | 0.932 | 0.858 | 1.73E-170 | 4.44E-166 | Ep-C2 |
| SRP54 | 0.629 | 0.825 | 0.669 | 2.57E-170 | 6.59E-166 | Ep-C2 |
| CCDC85B | 0.604 | 0.948 | 0.887 | 2.68E-170 | 6.88E-166 | Ep-C2 |
| TSPAN7 | 0.619 | 0.599 | 0.337 | 4.66E-169 | 1.20E-164 | Ep-C2 |
| ETFB | 0.636 | 0.991 | 0.972 | 3.95E-168 | 1.01E-163 | Ep-C2 |
| DNTTIP2 | 0.549 | 0.838 | 0.721 | 7.73E-167 | 1.98E-162 | Ep-C2 |
| GAS5 | 0.561 | 0.999 | 0.986 | 1.32E-166 | 3.38E-162 | Ep-C2 |
| MBIP | 0.602 | 0.655 | 0.448 | 2.29E-166 | 5.87E-162 | Ep-C2 |
| GSTP1 | 0.577 | 1 | 1 | 1.77E-165 | 4.53E-161 | Ep-C2 |
| PVT1 | 0.559 | 0.741 | 0.543 | 7.82E-165 | 2.01E-160 | Ep-C2 |
| LY6K | 0.647 | 0.832 | 0.766 | 2.66E-164 | 6.81E-160 | Ep-C2 |
| ANAPC15 | 0.572 | 0.804 | 0.64 | 1.68E-162 | 4.31E-158 | Ep-C2 |
| AIP | 0.583 | 0.799 | 0.639 | 1.54E-160 | 3.96E-156 | Ep-C2 |
| WNT5A | 0.592 | 0.523 | 0.261 | 2.07E-159 | 5.30E-155 | Ep-C2 |
| SPP1 | 1.165 | 0.439 | 0.235 | 7.72E-159 | 1.98E-154 | Ep-C2 |
| NQO1 | 0.656 | 0.959 | 0.885 | 1.66E-153 | 4.24E-149 | Ep-C2 |
| ALG1L | 0.532 | 0.749 | 0.51 | 9.35E-150 | 2.40E-145 | Ep-C2 |
| CALM2 | 0.693 | 0.997 | 0.997 | 2.08E-146 | 5.33E-142 | Ep-C2 |
| ABHD2 | 0.54 | 0.575 | 0.363 | 3.42E-146 | 8.76E-142 | Ep-C2 |
| NMRK1 | 0.551 | 0.62 | 0.428 | 4.59E-146 | 1.18E-141 | Ep-C2 |
| PSMA6 | 0.578 | 0.997 | 0.994 | 1.28E-145 | 3.29E-141 | Ep-C2 |
| COA4 | 0.513 | 0.944 | 0.891 | 5.98E-145 | 1.53E-140 | Ep-C2 |
| CLUAP1 | 0.544 | 0.673 | 0.436 | 8.92E-144 | 2.29E-139 | Ep-C2 |
| TXNRD1 | 0.601 | 0.886 | 0.736 | 1.98E-143 | 5.07E-139 | Ep-C2 |
| UGDH | 0.578 | 0.709 | 0.507 | 3.19E-143 | 8.18E-139 | Ep-C2 |
| RGMA | 0.507 | 0.486 | 0.24 | 1.26E-141 | 3.22E-137 | Ep-C2 |
| AKR1C2 | 0.555 | 0.953 | 0.826 | 1.54E-141 | 3.95E-137 | Ep-C2 |
| TM7SF2 | 0.535 | 0.595 | 0.33 | 3.52E-141 | 9.02E-137 | Ep-C2 |
| KRT8 | 0.568 | 0.957 | 0.838 | 2.64E-138 | 6.76E-134 | Ep-C2 |
| PAX9 | 0.609 | 0.524 | 0.288 | 7.94E-137 | 2.03E-132 | Ep-C2 |
| ALCAM | 0.593 | 0.798 | 0.629 | 4.61E-136 | 1.18E-131 | Ep-C2 |
| RNASEH2C | 0.558 | 0.847 | 0.738 | 5.68E-132 | 1.46E-127 | Ep-C2 |
| CHL1 | 0.516 | 0.399 | 0.173 | 1.45E-130 | 3.72E-126 | Ep-C2 |
| LTO1 | 0.586 | 0.696 | 0.504 | 2.07E-128 | 5.30E-124 | Ep-C2 |
| CAPNS2 | 0.745 | 0.544 | 0.346 | 5.28E-126 | 1.35E-121 | Ep-C2 |
| CTTN | 0.684 | 0.961 | 0.922 | 3.50E-123 | 8.96E-119 | Ep-C2 |
| CDK5RAP2 | 0.561 | 0.453 | 0.302 | 3.50E-113 | 8.97E-109 | Ep-C2 |
| MYH11 | 1.135 | 0.152 | 0.076 | 4.30E-112 | 1.10E-107 | Ep-C2 |
| KCNE3 | 0.556 | 0.255 | 0.093 | 1.73E-109 | 4.42E-105 | Ep-C2 |
| ME1 | 0.554 | 0.576 | 0.395 | 1.53E-108 | 3.93E-104 | Ep-C2 |
| SRXN1 | 0.513 | 0.676 | 0.495 | 1.74E-107 | 4.47E-103 | Ep-C2 |
| RAB6B | 0.601 | 0.309 | 0.156 | 5.30E-100 | 1.36E-95 | Ep-C2 |
| NUDT8 | 0.523 | 0.832 | 0.694 | 2.93E-99 | 7.51E-95 | Ep-C2 |
| GBP6 | 0.561 | 0.71 | 0.525 | 5.87E-91 | 1.50E-86 | Ep-C2 |
| IGFBP5 | 0.772 | 0.369 | 0.186 | 4.16E-81 | 1.07E-76 | Ep-C2 |
| MAP1B | 0.522 | 0.506 | 0.328 | 1.87E-75 | 4.78E-71 | Ep-C2 |
| SPARCL1 | 0.572 | 0.321 | 0.174 | 2.65E-73 | 6.79E-69 | Ep-C2 |
| GSN | 0.509 | 0.843 | 0.847 | 2.60E-28 | 6.65E-24 | Ep-C2 |
| NDRG1 | 2.156 | 0.996 | 0.861 | 0 | 0 | Ep-C3 |
| SLC2A1 | 2.151 | 0.989 | 0.883 | 0 | 0 | Ep-C3 |
| VEGFA | 1.741 | 0.933 | 0.659 | 0 | 0 | Ep-C3 |
| ERO1A | 1.737 | 0.914 | 0.669 | 0 | 0 | Ep-C3 |
| CCDC91 | 1.659 | 0.776 | 0.647 | 0 | 0 | Ep-C3 |
| PTHLH | 1.654 | 0.889 | 0.584 | 0 | 0 | Ep-C3 |
| SLC6A8 | 1.621 | 0.923 | 0.673 | 0 | 0 | Ep-C3 |
| P4HA1 | 1.54 | 0.813 | 0.399 | 0 | 0 | Ep-C3 |
| EGLN3 | 1.406 | 0.529 | 0.16 | 0 | 0 | Ep-C3 |
| PLOD2 | 1.376 | 0.737 | 0.496 | 0 | 0 | Ep-C3 |
| GJA1 | 1.293 | 0.856 | 0.598 | 0 | 0 | Ep-C3 |
| ENO2 | 1.181 | 0.636 | 0.229 | 0 | 0 | Ep-C3 |
| PGK1 | 1.133 | 0.998 | 0.99 | 0 | 0 | Ep-C3 |
| NOL3 | 1.031 | 0.791 | 0.571 | 0 | 0 | Ep-C3 |
| GAPDH | 0.932 | 1 | 1 | 0 | 0 | Ep-C3 |
| C4orf3 | 0.917 | 0.996 | 0.953 | 0 | 0 | Ep-C3 |
| BNIP3L | 0.874 | 0.926 | 0.732 | 0 | 0 | Ep-C3 |
| MIF | 0.805 | 1 | 1 | 0 | 0 | Ep-C3 |
| ENO1 | 0.804 | 1 | 0.999 | 0 | 0 | Ep-C3 |
| IGFBP2 | 1.66 | 0.987 | 0.914 | 1.5954403408232e-316 | 4.08927313756395e-312 | Ep-C3 |
| SLC16A3 | 0.913 | 0.822 | 0.562 | 3.6723461219152e-316 | 9.41259034508085e-312 | Ep-C3 |
| CA9 | 1.544 | 0.487 | 0.141 | 1.43E-296 | 3.66E-292 | Ep-C3 |
| MME | 1.62 | 0.405 | 0.118 | 1.28E-285 | 3.27E-281 | Ep-C3 |
| TMEM91 | 0.895 | 0.641 | 0.391 | 1.88E-281 | 4.83E-277 | Ep-C3 |
| WASF2 | 0.879 | 0.995 | 0.979 | 1.51E-280 | 3.88E-276 | Ep-C3 |
| NDUFA4L2 | 1.884 | 0.766 | 0.472 | 1.94E-273 | 4.98E-269 | Ep-C3 |
| AF165147.1 | 0.876 | 0.329 | 0.087 | 3.20E-267 | 8.20E-263 | Ep-C3 |
| SLC38A2 | 0.925 | 0.948 | 0.878 | 2.95E-265 | 7.57E-261 | Ep-C3 |
| FAM162A | 0.922 | 0.992 | 0.941 | 3.58E-254 | 9.17E-250 | Ep-C3 |
| CAST | 1.029 | 0.968 | 0.917 | 4.69E-251 | 1.20E-246 | Ep-C3 |
| BNIP3 | 1.302 | 0.788 | 0.509 | 1.74E-250 | 4.46E-246 | Ep-C3 |
| RPL34 | 0.527 | 1 | 1 | 4.92E-239 | 1.26E-234 | Ep-C3 |
| RPL39 | 0.695 | 0.999 | 1 | 2.65E-236 | 6.79E-232 | Ep-C3 |
| PLS3 | 0.871 | 0.915 | 0.733 | 5.95E-235 | 1.52E-230 | Ep-C3 |
| IGKC | 0.793 | 0.977 | 0.938 | 2.51E-234 | 6.43E-230 | Ep-C3 |
| VIM | 1.036 | 0.828 | 0.485 | 7.52E-234 | 1.93E-229 | Ep-C3 |
| COMMD6 | 0.742 | 0.997 | 0.991 | 4.41E-230 | 1.13E-225 | Ep-C3 |
| FSCN1 | 0.759 | 0.992 | 0.946 | 5.47E-228 | 1.40E-223 | Ep-C3 |
| SLCO1B3 | 0.872 | 0.567 | 0.3 | 3.68E-222 | 9.44E-218 | Ep-C3 |
| EIF4A2 | 0.624 | 1 | 0.995 | 1.23E-217 | 3.15E-213 | Ep-C3 |
| AHNAK2 | 0.905 | 0.866 | 0.595 | 2.87E-212 | 7.35E-208 | Ep-C3 |
| PNCK | 0.715 | 0.438 | 0.161 | 3.21E-205 | 8.22E-201 | Ep-C3 |
| LGALS1 | 1.237 | 0.852 | 0.588 | 5.66E-204 | 1.45E-199 | Ep-C3 |
| SUN1 | 0.672 | 0.797 | 0.656 | 3.53E-202 | 9.04E-198 | Ep-C3 |
| ELL2 | 0.661 | 0.565 | 0.408 | 4.26E-202 | 1.09E-197 | Ep-C3 |
| CLEC2B | 1.379 | 0.755 | 0.546 | 7.57E-201 | 1.94E-196 | Ep-C3 |
| MT1X | 1.189 | 0.985 | 0.965 | 9.27E-201 | 2.38E-196 | Ep-C3 |
| DST | 1.051 | 0.958 | 0.86 | 1.45E-200 | 3.71E-196 | Ep-C3 |
| LAMP2 | 0.742 | 0.874 | 0.783 | 3.25E-198 | 8.33E-194 | Ep-C3 |
| TPBG | 0.923 | 0.828 | 0.67 | 3.88E-198 | 9.93E-194 | Ep-C3 |
| PDK1 | 0.563 | 0.458 | 0.272 | 5.56E-198 | 1.43E-193 | Ep-C3 |
| IGF2BP2 | 0.73 | 0.865 | 0.666 | 4.55E-196 | 1.17E-191 | Ep-C3 |
| MIR210HG | 0.61 | 0.481 | 0.233 | 2.01E-190 | 5.15E-186 | Ep-C3 |
| PFKP | 0.605 | 0.63 | 0.487 | 2.47E-186 | 6.32E-182 | Ep-C3 |
| PHLDB2 | 0.772 | 0.741 | 0.553 | 8.26E-183 | 2.12E-178 | Ep-C3 |
| POLR1D | 0.587 | 0.995 | 0.992 | 2.24E-181 | 5.73E-177 | Ep-C3 |
| RPS3 | 0.669 | 1 | 1 | 1.42E-180 | 3.64E-176 | Ep-C3 |
| IGLC2 | 0.668 | 0.776 | 0.628 | 4.37E-177 | 1.12E-172 | Ep-C3 |
| MXI1 | 0.563 | 0.582 | 0.467 | 4.84E-177 | 1.24E-172 | Ep-C3 |
| MKNK2 | 0.77 | 0.844 | 0.727 | 3.76E-176 | 9.65E-172 | Ep-C3 |
| LGALS7 | 1.015 | 0.697 | 0.403 | 1.08E-174 | 2.77E-170 | Ep-C3 |
| GPI | 0.841 | 0.945 | 0.865 | 1.04E-173 | 2.66E-169 | Ep-C3 |
| DDX17 | 0.621 | 0.931 | 0.861 | 1.91E-172 | 4.90E-168 | Ep-C3 |
| IGFL2-AS1 | 1.046 | 0.56 | 0.276 | 4.77E-170 | 1.22E-165 | Ep-C3 |
| MAD1L1 | 0.568 | 0.465 | 0.346 | 1.62E-169 | 4.15E-165 | Ep-C3 |
| KIZ | 0.702 | 0.557 | 0.412 | 1.46E-165 | 3.73E-161 | Ep-C3 |
| CPE | 0.897 | 0.471 | 0.3 | 1.52E-165 | 3.90E-161 | Ep-C3 |
| PGF | 0.792 | 0.523 | 0.319 | 3.62E-164 | 9.28E-160 | Ep-C3 |
| KDM5B | 0.673 | 0.837 | 0.698 | 1.30E-163 | 3.34E-159 | Ep-C3 |
| AC011632.1 | 0.718 | 0.717 | 0.502 | 5.09E-160 | 1.30E-155 | Ep-C3 |
| S100A2 | 0.789 | 0.999 | 0.996 | 2.52E-159 | 6.46E-155 | Ep-C3 |
| C19orf33 | 0.594 | 0.981 | 0.882 | 5.12E-159 | 1.31E-154 | Ep-C3 |
| RNMT | 0.596 | 0.871 | 0.787 | 6.72E-157 | 1.72E-152 | Ep-C3 |
| AHNAK | 0.54 | 0.997 | 0.967 | 8.38E-157 | 2.15E-152 | Ep-C3 |
| CYSTM1 | 0.654 | 0.893 | 0.793 | 2.80E-155 | 7.17E-151 | Ep-C3 |
| P4HA2 | 0.619 | 0.579 | 0.377 | 1.22E-154 | 3.13E-150 | Ep-C3 |
| LPCAT2 | 0.805 | 0.605 | 0.449 | 1.40E-154 | 3.58E-150 | Ep-C3 |
| CLDN1 | 1.102 | 0.824 | 0.694 | 4.69E-154 | 1.20E-149 | Ep-C3 |
| PGAM1 | 0.534 | 0.994 | 0.981 | 1.98E-152 | 5.06E-148 | Ep-C3 |
| S100A10 | 0.588 | 1 | 0.998 | 2.72E-151 | 6.97E-147 | Ep-C3 |
| CD44 | 0.54 | 0.997 | 0.976 | 4.91E-151 | 1.26E-146 | Ep-C3 |
| STK24 | 0.584 | 0.892 | 0.827 | 1.02E-150 | 2.62E-146 | Ep-C3 |
| NRN1 | 0.605 | 0.266 | 0.199 | 1.06E-148 | 2.72E-144 | Ep-C3 |
| COL17A1 | 0.903 | 0.707 | 0.447 | 1.45E-146 | 3.73E-142 | Ep-C3 |
| SNHG6 | 0.53 | 0.997 | 0.992 | 8.89E-146 | 2.28E-141 | Ep-C3 |
| RPS8 | 0.542 | 1 | 1 | 2.67E-142 | 6.85E-138 | Ep-C3 |
| CA2 | 1.241 | 0.571 | 0.377 | 9.63E-139 | 2.47E-134 | Ep-C3 |
| LRRFIP1 | 0.616 | 0.949 | 0.888 | 3.15E-135 | 8.08E-131 | Ep-C3 |
| ITGA5 | 0.745 | 0.397 | 0.219 | 1.02E-131 | 2.62E-127 | Ep-C3 |
| PAM | 0.517 | 0.43 | 0.246 | 9.39E-131 | 2.41E-126 | Ep-C3 |
| LOXL2 | 0.519 | 0.3 | 0.1 | 6.06E-130 | 1.55E-125 | Ep-C3 |
| CRIP2 | 0.635 | 0.59 | 0.334 | 1.41E-128 | 3.62E-124 | Ep-C3 |
| TMEM123 | 0.57 | 0.989 | 0.972 | 4.10E-128 | 1.05E-123 | Ep-C3 |
| P3H2 | 0.741 | 0.513 | 0.252 | 2.97E-126 | 7.62E-122 | Ep-C3 |
| LUM | 0.524 | 0.495 | 0.233 | 4.97E-124 | 1.27E-119 | Ep-C3 |
| IGHG1 | 0.516 | 0.624 | 0.49 | 2.17E-123 | 5.56E-119 | Ep-C3 |
| SEMA4B | 0.546 | 0.812 | 0.673 | 2.04E-122 | 5.23E-118 | Ep-C3 |
| PPFIBP1 | 0.537 | 0.729 | 0.583 | 4.55E-120 | 1.17E-115 | Ep-C3 |
| PLEC | 0.559 | 0.939 | 0.859 | 7.33E-119 | 1.88E-114 | Ep-C3 |
| MAF | 0.659 | 0.498 | 0.299 | 9.48E-119 | 2.43E-114 | Ep-C3 |
| TMEM265 | 0.591 | 0.753 | 0.531 | 1.03E-118 | 2.63E-114 | Ep-C3 |
| CD63 | 0.506 | 0.999 | 0.997 | 1.61E-118 | 4.13E-114 | Ep-C3 |
| UBE2B | 0.507 | 0.981 | 0.946 | 1.03E-117 | 2.64E-113 | Ep-C3 |
| SSR4 | 0.586 | 0.993 | 0.994 | 5.70E-115 | 1.46E-110 | Ep-C3 |
| ANGPTL4 | 0.799 | 0.407 | 0.247 | 8.87E-115 | 2.27E-110 | Ep-C3 |
| CTNND1 | 0.507 | 0.951 | 0.899 | 1.71E-114 | 4.39E-110 | Ep-C3 |
| ARGLU1 | 0.572 | 0.944 | 0.889 | 4.50E-114 | 1.15E-109 | Ep-C3 |
| EMP3 | 0.742 | 0.596 | 0.383 | 2.68E-113 | 6.86E-109 | Ep-C3 |
| FAM3C | 0.569 | 0.878 | 0.794 | 4.19E-113 | 1.07E-108 | Ep-C3 |
| SERPINE1 | 0.913 | 0.44 | 0.255 | 5.80E-113 | 1.49E-108 | Ep-C3 |
| DSG2 | 0.526 | 0.742 | 0.59 | 1.04E-112 | 2.65E-108 | Ep-C3 |
| ARRDC3 | 0.525 | 0.393 | 0.246 | 1.17E-112 | 3.00E-108 | Ep-C3 |
| IVNS1ABP | 0.597 | 0.8 | 0.667 | 9.38E-112 | 2.40E-107 | Ep-C3 |
| PTPRF | 0.572 | 0.855 | 0.783 | 2.39E-108 | 6.13E-104 | Ep-C3 |
| DSC2 | 0.556 | 0.746 | 0.554 | 9.15E-106 | 2.34E-101 | Ep-C3 |
| RPL10 | 0.563 | 0.999 | 1 | 4.20E-105 | 1.08E-100 | Ep-C3 |
| SELENOP | 0.787 | 0.909 | 0.746 | 1.43E-104 | 3.65E-100 | Ep-C3 |
| HERPUD1 | 0.763 | 0.888 | 0.822 | 1.41E-103 | 3.61E-99 | Ep-C3 |
| C1QTNF12 | 0.76 | 0.736 | 0.567 | 2.22E-102 | 5.69E-98 | Ep-C3 |
| LRP1 | 0.513 | 0.536 | 0.402 | 7.47E-102 | 1.92E-97 | Ep-C3 |
| NRIP1 | 0.523 | 0.669 | 0.531 | 3.80E-101 | 9.74E-97 | Ep-C3 |
| S100A4 | 0.864 | 0.735 | 0.595 | 1.42E-99 | 3.65E-95 | Ep-C3 |
| SQSTM1 | 0.533 | 0.988 | 0.974 | 8.33E-99 | 2.13E-94 | Ep-C3 |
| ITGA2 | 0.561 | 0.726 | 0.589 | 3.47E-98 | 8.91E-94 | Ep-C3 |
| SLITRK6 | 0.591 | 0.344 | 0.162 | 1.08E-96 | 2.77E-92 | Ep-C3 |
| LGALS3 | 0.567 | 0.991 | 0.963 | 4.58E-94 | 1.17E-89 | Ep-C3 |
| CRIP1 | 0.506 | 0.922 | 0.745 | 4.10E-93 | 1.05E-88 | Ep-C3 |
| PEPD | 0.627 | 0.568 | 0.598 | 2.99E-92 | 7.66E-88 | Ep-C3 |
| LGALS7B | 0.832 | 0.731 | 0.545 | 4.03E-92 | 1.03E-87 | Ep-C3 |
| DNAJB9 | 0.552 | 0.385 | 0.371 | 5.39E-91 | 1.38E-86 | Ep-C3 |
| PHLDA3 | 0.556 | 0.895 | 0.79 | 2.11E-89 | 5.40E-85 | Ep-C3 |
| EZR | 0.554 | 0.984 | 0.957 | 2.51E-89 | 6.43E-85 | Ep-C3 |
| HLA-B | 0.836 | 0.984 | 0.98 | 2.90E-89 | 7.43E-85 | Ep-C3 |
| ITGB4 | 0.506 | 0.943 | 0.858 | 5.07E-89 | 1.30E-84 | Ep-C3 |
| BIK | 0.508 | 0.527 | 0.421 | 8.47E-88 | 2.17E-83 | Ep-C3 |
| KRT17 | 0.75 | 0.99 | 0.932 | 3.23E-87 | 8.28E-83 | Ep-C3 |
| MIR205HG | 0.587 | 0.994 | 0.99 | 1.10E-86 | 2.83E-82 | Ep-C3 |
| HK2 | 0.574 | 0.416 | 0.279 | 4.98E-84 | 1.28E-79 | Ep-C3 |
| IGFBP6 | 0.511 | 0.772 | 0.552 | 6.24E-84 | 1.60E-79 | Ep-C3 |
| TNFRSF12A | 0.546 | 0.856 | 0.687 | 7.41E-84 | 1.90E-79 | Ep-C3 |
| COL7A1 | 0.526 | 0.672 | 0.526 | 1.17E-83 | 2.99E-79 | Ep-C3 |
| TRIB3 | 0.579 | 0.295 | 0.191 | 3.35E-82 | 8.59E-78 | Ep-C3 |
| TMEM45A | 0.58 | 0.428 | 0.256 | 4.07E-82 | 1.04E-77 | Ep-C3 |
| CALB1 | 0.923 | 0.268 | 0.128 | 7.05E-79 | 1.81E-74 | Ep-C3 |
| TSPAN1 | 0.62 | 0.521 | 0.355 | 7.15E-78 | 1.83E-73 | Ep-C3 |
| LAMB3 | 0.69 | 0.875 | 0.783 | 4.06E-76 | 1.04E-71 | Ep-C3 |
| MUC1 | 0.531 | 0.438 | 0.283 | 8.04E-75 | 2.06E-70 | Ep-C3 |
| DDIT3 | 0.679 | 0.811 | 0.716 | 1.42E-73 | 3.63E-69 | Ep-C3 |
| SCD | 0.534 | 0.823 | 0.674 | 9.40E-72 | 2.41E-67 | Ep-C3 |
| ANKRD37 | 0.67 | 0.514 | 0.438 | 1.37E-68 | 3.51E-64 | Ep-C3 |
| FBXO32 | 0.569 | 0.595 | 0.501 | 1.89E-66 | 4.86E-62 | Ep-C3 |
| GPNMB | 0.648 | 0.923 | 0.834 | 9.87E-66 | 2.53E-61 | Ep-C3 |
| HSPA5 | 0.66 | 0.995 | 0.989 | 1.31E-64 | 3.35E-60 | Ep-C3 |
| IL20RB | 0.563 | 0.564 | 0.459 | 3.91E-64 | 1.00E-59 | Ep-C3 |
| TM4SF1 | 0.603 | 0.974 | 0.944 | 5.21E-62 | 1.34E-57 | Ep-C3 |
| DDIT4 | 0.723 | 0.875 | 0.806 | 5.59E-52 | 1.43E-47 | Ep-C3 |
| AREG | 0.735 | 0.551 | 0.42 | 2.37E-45 | 6.06E-41 | Ep-C3 |
| CTSC | 0.565 | 0.921 | 0.879 | 5.75E-45 | 1.47E-40 | Ep-C3 |
| AC100801.1 | 0.521 | 0.218 | 0.103 | 1.49E-43 | 3.81E-39 | Ep-C3 |
| MMP7 | 0.767 | 0.262 | 0.201 | 4.61E-18 | 1.18E-13 | Ep-C3 |
| UBE2C | 3.256 | 0.927 | 0.291 | 0 | 0 | Ep-C4 |
| CENPF | 3.11 | 0.989 | 0.315 | 0 | 0 | Ep-C4 |
| CCNB1 | 3.05 | 0.984 | 0.266 | 0 | 0 | Ep-C4 |
| TOP2A | 3.003 | 0.978 | 0.308 | 0 | 0 | Ep-C4 |
| CDC20 | 2.781 | 0.962 | 0.212 | 0 | 0 | Ep-C4 |
| CDKN3 | 2.526 | 0.979 | 0.288 | 0 | 0 | Ep-C4 |
| TPX2 | 2.413 | 0.976 | 0.3 | 0 | 0 | Ep-C4 |
| ASPM | 2.367 | 0.914 | 0.175 | 0 | 0 | Ep-C4 |
| KPNA2 | 2.35 | 0.966 | 0.604 | 0 | 0 | Ep-C4 |
| BIRC5 | 2.314 | 0.986 | 0.308 | 0 | 0 | Ep-C4 |
| CCNB2 | 2.31 | 0.95 | 0.208 | 0 | 0 | Ep-C4 |
| ARL6IP1 | 2.297 | 1 | 0.922 | 0 | 0 | Ep-C4 |
| PLK1 | 2.269 | 0.881 | 0.102 | 0 | 0 | Ep-C4 |
| NUSAP1 | 2.225 | 0.942 | 0.269 | 0 | 0 | Ep-C4 |
| HMMR | 2.207 | 0.896 | 0.133 | 0 | 0 | Ep-C4 |
| CKS2 | 2.195 | 0.988 | 0.765 | 0 | 0 | Ep-C4 |
| HMGB2 | 2.163 | 0.994 | 0.768 | 0 | 0 | Ep-C4 |
| MKI67 | 2.131 | 0.914 | 0.221 | 0 | 0 | Ep-C4 |
| CDK1 | 2.117 | 0.815 | 0.298 | 0 | 0 | Ep-C4 |
| PTTG1 | 2.116 | 0.999 | 0.755 | 0 | 0 | Ep-C4 |
| UBE2S | 1.999 | 0.997 | 0.936 | 0 | 0 | Ep-C4 |
| CENPA | 1.975 | 0.863 | 0.106 | 0 | 0 | Ep-C4 |
| DLGAP5 | 1.936 | 0.864 | 0.112 | 0 | 0 | Ep-C4 |
| CDCA3 | 1.924 | 0.843 | 0.137 | 0 | 0 | Ep-C4 |
| NUF2 | 1.877 | 0.887 | 0.169 | 0 | 0 | Ep-C4 |
| CENPE | 1.876 | 0.849 | 0.151 | 0 | 0 | Ep-C4 |
| ECT2 | 1.849 | 0.95 | 0.386 | 0 | 0 | Ep-C4 |
| PRC1 | 1.847 | 0.933 | 0.267 | 0 | 0 | Ep-C4 |
| AURKA | 1.812 | 0.806 | 0.144 | 0 | 0 | Ep-C4 |
| KIF20B | 1.752 | 0.888 | 0.271 | 0 | 0 | Ep-C4 |
| CKS1B | 1.729 | 0.998 | 0.822 | 0 | 0 | Ep-C4 |
| NCAPD2 | 1.716 | 0.855 | 0.253 | 0 | 0 | Ep-C4 |
| GTSE1 | 1.688 | 0.821 | 0.121 | 0 | 0 | Ep-C4 |
| SMC4 | 1.671 | 0.993 | 0.707 | 0 | 0 | Ep-C4 |
| TROAP | 1.646 | 0.87 | 0.174 | 0 | 0 | Ep-C4 |
| NEK2 | 1.633 | 0.781 | 0.087 | 0 | 0 | Ep-C4 |
| CEP55 | 1.631 | 0.863 | 0.173 | 0 | 0 | Ep-C4 |
| CCNA2 | 1.586 | 0.814 | 0.139 | 0 | 0 | Ep-C4 |
| MAD2L1 | 1.557 | 0.953 | 0.363 | 0 | 0 | Ep-C4 |
| AURKB | 1.55 | 0.823 | 0.144 | 0 | 0 | Ep-C4 |
| KIF2C | 1.511 | 0.774 | 0.121 | 0 | 0 | Ep-C4 |
| CKAP2 | 1.509 | 0.855 | 0.327 | 0 | 0 | Ep-C4 |
| KIF23 | 1.483 | 0.786 | 0.142 | 0 | 0 | Ep-C4 |
| TUBA1B | 1.463 | 1 | 0.96 | 0 | 0 | Ep-C4 |
| KNSTRN | 1.432 | 0.828 | 0.207 | 0 | 0 | Ep-C4 |
| CDCA8 | 1.431 | 0.757 | 0.116 | 0 | 0 | Ep-C4 |
| DEPDC1 | 1.392 | 0.74 | 0.09 | 0 | 0 | Ep-C4 |
| ANLN | 1.373 | 0.753 | 0.176 | 0 | 0 | Ep-C4 |
| RAD21 | 1.36 | 0.995 | 0.878 | 0 | 0 | Ep-C4 |
| PBK | 1.358 | 0.847 | 0.191 | 0 | 0 | Ep-C4 |
| SGO2 | 1.346 | 0.744 | 0.119 | 0 | 0 | Ep-C4 |
| CENPW | 1.346 | 0.994 | 0.668 | 0 | 0 | Ep-C4 |
| MZT1 | 1.337 | 0.961 | 0.643 | 0 | 0 | Ep-C4 |
| TUBA1C | 1.335 | 0.999 | 0.986 | 0 | 0 | Ep-C4 |
| H2AFX | 1.321 | 0.958 | 0.633 | 0 | 0 | Ep-C4 |
| UBE2T | 1.311 | 0.938 | 0.427 | 0 | 0 | Ep-C4 |
| TUBB4B | 1.293 | 0.998 | 0.991 | 0 | 0 | Ep-C4 |
| SKA2 | 1.291 | 0.98 | 0.654 | 0 | 0 | Ep-C4 |
| TACC3 | 1.276 | 0.777 | 0.145 | 0 | 0 | Ep-C4 |
| STMN1 | 1.268 | 0.996 | 0.868 | 0 | 0 | Ep-C4 |
| HMGN2 | 1.26 | 1 | 0.965 | 0 | 0 | Ep-C4 |
| FOXM1 | 1.257 | 0.78 | 0.212 | 0 | 0 | Ep-C4 |
| HMGB3 | 1.254 | 0.988 | 0.783 | 0 | 0 | Ep-C4 |
| FAM83D | 1.251 | 0.763 | 0.258 | 0 | 0 | Ep-C4 |
| ANP32E | 1.235 | 0.986 | 0.765 | 0 | 0 | Ep-C4 |
| KIF11 | 1.195 | 0.755 | 0.161 | 0 | 0 | Ep-C4 |
| RACGAP1 | 1.193 | 0.772 | 0.177 | 0 | 0 | Ep-C4 |
| CKAP5 | 1.192 | 0.825 | 0.285 | 0 | 0 | Ep-C4 |
| H2AFZ | 1.184 | 1 | 0.996 | 0 | 0 | Ep-C4 |
| PIMREG | 1.173 | 0.685 | 0.082 | 0 | 0 | Ep-C4 |
| NDC80 | 1.173 | 0.678 | 0.135 | 0 | 0 | Ep-C4 |
| DTYMK | 1.162 | 0.941 | 0.573 | 0 | 0 | Ep-C4 |
| HMGB1 | 1.124 | 1 | 0.999 | 0 | 0 | Ep-C4 |
| KIF14 | 1.122 | 0.629 | 0.074 | 0 | 0 | Ep-C4 |
| CDC25B | 1.12 | 0.87 | 0.407 | 0 | 0 | Ep-C4 |
| BUB1 | 1.118 | 0.708 | 0.083 | 0 | 0 | Ep-C4 |
| KIF4A | 1.114 | 0.704 | 0.096 | 0 | 0 | Ep-C4 |
| TTK | 1.091 | 0.688 | 0.121 | 0 | 0 | Ep-C4 |
| TNFAIP8L1 | 1.082 | 0.647 | 0.097 | 0 | 0 | Ep-C4 |
| MRPL51 | 1.075 | 1 | 0.987 | 0 | 0 | Ep-C4 |
| NUCKS1 | 1.067 | 0.991 | 0.937 | 0 | 0 | Ep-C4 |
| CENPN | 1.058 | 0.857 | 0.327 | 0 | 0 | Ep-C4 |
| NCAPG | 1.049 | 0.696 | 0.125 | 0 | 0 | Ep-C4 |
| ARHGAP11A | 1.045 | 0.683 | 0.118 | 0 | 0 | Ep-C4 |
| JPT1 | 1.044 | 0.998 | 0.998 | 0 | 0 | Ep-C4 |
| GPSM2 | 1.036 | 0.769 | 0.296 | 0 | 0 | Ep-C4 |
| TUBB | 1.032 | 1 | 0.979 | 0 | 0 | Ep-C4 |
| PRR11 | 1.03 | 0.682 | 0.105 | 0 | 0 | Ep-C4 |
| BUB3 | 1.027 | 0.95 | 0.756 | 0 | 0 | Ep-C4 |
| KIFC1 | 1.026 | 0.659 | 0.126 | 0 | 0 | Ep-C4 |
| CKAP2L | 1.025 | 0.643 | 0.108 | 0 | 0 | Ep-C4 |
| LMNB1 | 1.011 | 0.736 | 0.25 | 0 | 0 | Ep-C4 |
| KIF20A | 1.008 | 0.601 | 0.044 | 0 | 0 | Ep-C4 |
| RANGAP1 | 1.008 | 0.84 | 0.426 | 0 | 0 | Ep-C4 |
| CDCA2 | 1.007 | 0.619 | 0.099 | 0 | 0 | Ep-C4 |
| KIF22 | 0.998 | 0.867 | 0.395 | 0 | 0 | Ep-C4 |
| SMC2 | 0.998 | 0.904 | 0.432 | 0 | 0 | Ep-C4 |
| UBALD2 | 0.997 | 0.972 | 0.888 | 0 | 0 | Ep-C4 |
| TMPO | 0.995 | 0.914 | 0.517 | 0 | 0 | Ep-C4 |
| LMNB2 | 0.994 | 0.856 | 0.419 | 0 | 0 | Ep-C4 |
| DBF4 | 0.988 | 0.726 | 0.328 | 0 | 0 | Ep-C4 |
| KNL1 | 0.986 | 0.666 | 0.124 | 0 | 0 | Ep-C4 |
| H2AFV | 0.97 | 0.999 | 0.961 | 0 | 0 | Ep-C4 |
| PSRC1 | 0.965 | 0.608 | 0.063 | 0 | 0 | Ep-C4 |
| CALM2 | 0.963 | 1 | 0.996 | 0 | 0 | Ep-C4 |
| TRIM59 | 0.958 | 0.762 | 0.403 | 0 | 0 | Ep-C4 |
| TK1 | 0.941 | 0.967 | 0.518 | 0 | 0 | Ep-C4 |
| SGO1 | 0.937 | 0.676 | 0.134 | 0 | 0 | Ep-C4 |
| DDX39A | 0.928 | 0.944 | 0.659 | 0 | 0 | Ep-C4 |
| HJURP | 0.922 | 0.566 | 0.086 | 0 | 0 | Ep-C4 |
| CIP2A | 0.922 | 0.688 | 0.173 | 0 | 0 | Ep-C4 |
| SAPCD2 | 0.917 | 0.661 | 0.208 | 0 | 0 | Ep-C4 |
| SPC25 | 0.901 | 0.665 | 0.15 | 0 | 0 | Ep-C4 |
| NCAPH | 0.9 | 0.63 | 0.127 | 0 | 0 | Ep-C4 |
| MIS18BP1 | 0.899 | 0.787 | 0.382 | 0 | 0 | Ep-C4 |
| TEDC1 | 0.898 | 0.743 | 0.273 | 0 | 0 | Ep-C4 |
| ZWINT | 0.887 | 0.876 | 0.36 | 0 | 0 | Ep-C4 |
| KIF18A | 0.872 | 0.588 | 0.087 | 0 | 0 | Ep-C4 |
| PHF19 | 0.859 | 0.765 | 0.29 | 0 | 0 | Ep-C4 |
| SPAG5 | 0.85 | 0.633 | 0.102 | 0 | 0 | Ep-C4 |
| EMC9 | 0.844 | 0.757 | 0.351 | 0 | 0 | Ep-C4 |
| ARHGEF39 | 0.842 | 0.471 | 0.091 | 0 | 0 | Ep-C4 |
| RPL39L | 0.835 | 0.981 | 0.75 | 0 | 0 | Ep-C4 |
| CCNF | 0.832 | 0.538 | 0.086 | 0 | 0 | Ep-C4 |
| PIF1 | 0.825 | 0.451 | 0.031 | 0 | 0 | Ep-C4 |
| DEPDC1B | 0.819 | 0.598 | 0.09 | 0 | 0 | Ep-C4 |
| BORA | 0.819 | 0.523 | 0.081 | 0 | 0 | Ep-C4 |
| GGH | 0.815 | 0.937 | 0.578 | 0 | 0 | Ep-C4 |
| HNRNPA2B1 | 0.797 | 1 | 0.998 | 0 | 0 | Ep-C4 |
| CIT | 0.789 | 0.595 | 0.101 | 0 | 0 | Ep-C4 |
| BCL2L12 | 0.786 | 0.83 | 0.397 | 0 | 0 | Ep-C4 |
| RNF26 | 0.765 | 0.663 | 0.221 | 0 | 0 | Ep-C4 |
| BUB1B | 0.747 | 0.543 | 0.094 | 0 | 0 | Ep-C4 |
| ILF2 | 0.699 | 0.997 | 0.953 | 0 | 0 | Ep-C4 |
| KIF15 | 0.694 | 0.541 | 0.092 | 0 | 0 | Ep-C4 |
| PARPBP | 0.686 | 0.573 | 0.145 | 0 | 0 | Ep-C4 |
| OIP5 | 0.685 | 0.542 | 0.104 | 0 | 0 | Ep-C4 |
| POC1A | 0.651 | 0.598 | 0.157 | 0 | 0 | Ep-C4 |
| GAS2L3 | 0.616 | 0.397 | 0.029 | 0 | 0 | Ep-C4 |
| CDC25C | 0.591 | 0.427 | 0.043 | 0 | 0 | Ep-C4 |
| RRM2 | 0.576 | 0.708 | 0.221 | 0 | 0 | Ep-C4 |
| KIF18B | 0.576 | 0.446 | 0.063 | 0 | 0 | Ep-C4 |
| LBR | 0.822 | 0.841 | 0.496 | 2.51973479379036e-322 | 6.45833224996406e-318 | Ep-C4 |
| MXD3 | 0.601 | 0.441 | 0.076 | 1.24504542751994e-321 | 3.19117593527636e-317 | Ep-C4 |
| RAN | 0.675 | 1 | 0.999 | 1.00048293282852e-320 | 2.56433780513279e-316 | Ep-C4 |
| RAD51AP1 | 0.781 | 0.696 | 0.232 | 1.48714105244167e-317 | 3.81169123151325e-313 | Ep-C4 |
| GLRX5 | 0.743 | 0.987 | 0.915 | 8.63937368002039e-317 | 2.21435786792603e-312 | Ep-C4 |
| NMU | 0.999 | 0.693 | 0.287 | 2.96683374684451e-311 | 7.60E-307 | Ep-C4 |
| NUDT1 | 0.784 | 0.988 | 0.798 | 6.60649237439321e-310 | 1.69E-305 | Ep-C4 |
| SKA3 | 0.608 | 0.555 | 0.138 | 1.20674769813296e-309 | 3.09E-305 | Ep-C4 |
| CCDC18 | 0.572 | 0.476 | 0.1 | 6.86937534029838e-309 | 1.76E-304 | Ep-C4 |
| CDCA5 | 0.627 | 0.61 | 0.176 | 1.81E-306 | 4.64E-302 | Ep-C4 |
| INCENP | 0.589 | 0.449 | 0.094 | 2.30E-305 | 5.90E-301 | Ep-C4 |
| CCDC34 | 0.81 | 0.804 | 0.393 | 9.67E-305 | 2.48E-300 | Ep-C4 |
| PTMS | 0.944 | 0.998 | 0.968 | 2.68E-302 | 6.87E-298 | Ep-C4 |
| TRIP13 | 0.719 | 0.648 | 0.219 | 2.00E-299 | 5.12E-295 | Ep-C4 |
| CNTRL | 0.723 | 0.563 | 0.188 | 3.11E-299 | 7.98E-295 | Ep-C4 |
| SHCBP1 | 0.567 | 0.508 | 0.118 | 1.73E-293 | 4.43E-289 | Ep-C4 |
| DIAPH3 | 0.635 | 0.521 | 0.126 | 8.14E-291 | 2.09E-286 | Ep-C4 |
| SPDL1 | 0.568 | 0.486 | 0.113 | 1.68E-290 | 4.30E-286 | Ep-C4 |
| CEP70 | 0.823 | 0.69 | 0.404 | 1.19E-289 | 3.06E-285 | Ep-C4 |
| G2E3 | 0.68 | 0.591 | 0.219 | 1.98E-288 | 5.08E-284 | Ep-C4 |
| SUN2 | 0.823 | 0.752 | 0.372 | 2.80E-285 | 7.17E-281 | Ep-C4 |
| C16orf95 | 0.764 | 0.559 | 0.211 | 8.86E-283 | 2.27E-278 | Ep-C4 |
| PTMA | 0.642 | 1 | 1 | 1.79E-281 | 4.59E-277 | Ep-C4 |
| HYLS1 | 0.668 | 0.437 | 0.11 | 1.61E-275 | 4.13E-271 | Ep-C4 |
| PCLAF | 0.549 | 0.86 | 0.428 | 1.07E-272 | 2.75E-268 | Ep-C4 |
| MND1 | 0.595 | 0.585 | 0.178 | 3.88E-269 | 9.94E-265 | Ep-C4 |
| CENPM | 0.798 | 0.754 | 0.323 | 2.31E-268 | 5.92E-264 | Ep-C4 |
| PGP | 0.844 | 0.9 | 0.614 | 2.43E-267 | 6.24E-263 | Ep-C4 |
| LSM5 | 0.63 | 0.996 | 0.962 | 3.22E-265 | 8.25E-261 | Ep-C4 |
| GOT1 | 0.705 | 0.792 | 0.463 | 5.11E-265 | 1.31E-260 | Ep-C4 |
| SCLT1 | 0.61 | 0.535 | 0.174 | 3.80E-262 | 9.75E-258 | Ep-C4 |
| MYBL2 | 0.633 | 0.673 | 0.252 | 1.44E-261 | 3.69E-257 | Ep-C4 |
| ORC6 | 0.643 | 0.716 | 0.286 | 1.69E-261 | 4.33E-257 | Ep-C4 |
| NEURL1B | 0.536 | 0.36 | 0.064 | 4.98E-261 | 1.28E-256 | Ep-C4 |
| MTFR2 | 0.518 | 0.432 | 0.092 | 6.16E-260 | 1.58E-255 | Ep-C4 |
| CSE1L | 0.678 | 0.865 | 0.516 | 9.84E-259 | 2.52E-254 | Ep-C4 |
| HP1BP3 | 0.711 | 0.959 | 0.826 | 5.03E-255 | 1.29E-250 | Ep-C4 |
| NUP37 | 0.687 | 0.818 | 0.493 | 7.05E-254 | 1.81E-249 | Ep-C4 |
| CALM3 | 0.695 | 0.96 | 0.785 | 2.19E-251 | 5.61E-247 | Ep-C4 |
| MELK | 0.589 | 0.556 | 0.175 | 3.15E-241 | 8.09E-237 | Ep-C4 |
| CCT5 | 0.611 | 0.999 | 0.984 | 5.49E-238 | 1.41E-233 | Ep-C4 |
| CNIH4 | 0.602 | 0.99 | 0.947 | 3.16E-233 | 8.11E-229 | Ep-C4 |
| NUDCD2 | 0.625 | 0.888 | 0.654 | 3.00E-231 | 7.68E-227 | Ep-C4 |
| REEP4 | 0.709 | 0.833 | 0.516 | 6.07E-228 | 1.56E-223 | Ep-C4 |
| PTGES3 | 0.537 | 1 | 0.998 | 7.08E-228 | 1.81E-223 | Ep-C4 |
| MAGOHB | 0.745 | 0.865 | 0.586 | 6.50E-227 | 1.67E-222 | Ep-C4 |
| RUVBL2 | 0.63 | 0.904 | 0.636 | 7.70E-225 | 1.97E-220 | Ep-C4 |
| CENPK | 0.54 | 0.629 | 0.241 | 5.67E-224 | 1.45E-219 | Ep-C4 |
| ODF2 | 0.544 | 0.546 | 0.2 | 9.38E-224 | 2.40E-219 | Ep-C4 |
| FANCI | 0.536 | 0.607 | 0.224 | 4.80E-222 | 1.23E-217 | Ep-C4 |
| VRK1 | 0.612 | 0.706 | 0.315 | 1.12E-220 | 2.87E-216 | Ep-C4 |
| LSM4 | 0.618 | 0.997 | 0.944 | 2.35E-218 | 6.03E-214 | Ep-C4 |
| PAK4 | 0.666 | 0.639 | 0.323 | 8.64E-217 | 2.21E-212 | Ep-C4 |
| RHNO1 | 0.728 | 0.652 | 0.311 | 7.54E-215 | 1.93E-210 | Ep-C4 |
| TTF2 | 0.603 | 0.655 | 0.299 | 4.16E-212 | 1.07E-207 | Ep-C4 |
| BRD8 | 0.544 | 0.55 | 0.209 | 4.84E-209 | 1.24E-204 | Ep-C4 |
| HSP90AA1 | 0.821 | 1 | 1 | 1.71E-208 | 4.37E-204 | Ep-C4 |
| LYAR | 0.691 | 0.724 | 0.392 | 8.20E-208 | 2.10E-203 | Ep-C4 |
| DCAF7 | 0.608 | 0.853 | 0.586 | 3.18E-207 | 8.14E-203 | Ep-C4 |
| SFPQ | 0.573 | 0.998 | 0.97 | 6.92E-206 | 1.77E-201 | Ep-C4 |
| SAP30 | 0.666 | 0.73 | 0.407 | 1.20E-205 | 3.08E-201 | Ep-C4 |
| CENPH | 0.587 | 0.714 | 0.337 | 1.85E-205 | 4.75E-201 | Ep-C4 |
| TIMM10 | 0.655 | 0.917 | 0.726 | 4.68E-203 | 1.20E-198 | Ep-C4 |
| DYNLL1 | 0.64 | 1 | 0.998 | 2.13E-202 | 5.46E-198 | Ep-C4 |
| MIS18A | 0.609 | 0.653 | 0.3 | 1.04E-201 | 2.65E-197 | Ep-C4 |
| HNRNPA3 | 0.593 | 1 | 0.998 | 2.90E-196 | 7.44E-192 | Ep-C4 |
| RANBP1 | 0.606 | 0.999 | 0.982 | 7.95E-193 | 2.04E-188 | Ep-C4 |
| MLF2 | 0.587 | 0.995 | 0.97 | 8.20E-188 | 2.10E-183 | Ep-C4 |
| CBX3 | 0.5 | 0.992 | 0.958 | 1.15E-187 | 2.95E-183 | Ep-C4 |
| RCCD1 | 0.503 | 0.536 | 0.216 | 3.19E-187 | 8.17E-183 | Ep-C4 |
| STIP1 | 0.64 | 0.898 | 0.697 | 6.03E-185 | 1.55E-180 | Ep-C4 |
| HIST1H2BJ | 0.93 | 0.476 | 0.187 | 1.89E-180 | 4.85E-176 | Ep-C4 |
| HNRNPR | 0.503 | 0.987 | 0.943 | 6.46E-180 | 1.66E-175 | Ep-C4 |
| DLEU2 | 0.501 | 0.523 | 0.201 | 2.25E-179 | 5.78E-175 | Ep-C4 |
| XRCC6 | 0.583 | 0.995 | 0.961 | 4.48E-179 | 1.15E-174 | Ep-C4 |
| ANP32B | 0.511 | 0.999 | 0.994 | 1.15E-178 | 2.94E-174 | Ep-C4 |
| SSNA1 | 0.521 | 0.978 | 0.902 | 1.17E-176 | 3.00E-172 | Ep-C4 |
| ACTL6A | 0.523 | 0.985 | 0.891 | 1.64E-174 | 4.20E-170 | Ep-C4 |
| CCAR1 | 0.605 | 0.907 | 0.729 | 9.61E-174 | 2.46E-169 | Ep-C4 |
| GRK6 | 0.536 | 0.674 | 0.365 | 1.20E-172 | 3.08E-168 | Ep-C4 |
| HIST1H3G | 0.612 | 0.313 | 0.074 | 3.80E-172 | 9.73E-168 | Ep-C4 |
| HNRNPUL1 | 0.764 | 0.908 | 0.737 | 3.56E-167 | 9.13E-163 | Ep-C4 |
| RCC1 | 0.521 | 0.621 | 0.302 | 1.34E-166 | 3.43E-162 | Ep-C4 |
| PRPSAP1 | 0.521 | 0.881 | 0.644 | 9.78E-166 | 2.51E-161 | Ep-C4 |
| HSP90B1 | 0.532 | 1 | 0.991 | 2.31E-165 | 5.92E-161 | Ep-C4 |
| PPIH | 0.566 | 0.834 | 0.581 | 2.09E-164 | 5.36E-160 | Ep-C4 |
| NDE1 | 0.64 | 0.68 | 0.411 | 9.29E-164 | 2.38E-159 | Ep-C4 |
| CCDC88A | 0.724 | 0.611 | 0.319 | 6.82E-159 | 1.75E-154 | Ep-C4 |
| UBB | 0.565 | 0.999 | 0.999 | 4.65E-157 | 1.19E-152 | Ep-C4 |
| FOPNL | 0.552 | 0.782 | 0.516 | 3.76E-155 | 9.65E-151 | Ep-C4 |
| SNRPD1 | 0.526 | 0.996 | 0.978 | 4.65E-154 | 1.19E-149 | Ep-C4 |
| NUDT15 | 0.553 | 0.756 | 0.478 | 8.33E-154 | 2.14E-149 | Ep-C4 |
| NSD2 | 0.51 | 0.598 | 0.289 | 3.03E-152 | 7.78E-148 | Ep-C4 |
| CRNDE | 0.686 | 0.933 | 0.795 | 1.31E-151 | 3.36E-147 | Ep-C4 |
| RBM42 | 0.847 | 0.84 | 0.643 | 6.21E-150 | 1.59E-145 | Ep-C4 |
| SS18 | 0.636 | 0.765 | 0.506 | 3.81E-146 | 9.77E-142 | Ep-C4 |
| DESI2 | 0.515 | 0.846 | 0.62 | 5.01E-146 | 1.28E-141 | Ep-C4 |
| CACYBP | 0.526 | 0.985 | 0.928 | 6.46E-146 | 1.66E-141 | Ep-C4 |
| CDKN1B | 0.607 | 0.632 | 0.367 | 2.84E-141 | 7.29E-137 | Ep-C4 |
| UQCC3 | 0.554 | 0.933 | 0.794 | 3.03E-141 | 7.78E-137 | Ep-C4 |
| SPTBN1 | 0.536 | 0.84 | 0.597 | 1.28E-140 | 3.29E-136 | Ep-C4 |
| WBP11 | 0.689 | 0.785 | 0.568 | 7.84E-140 | 2.01E-135 | Ep-C4 |
| HSPE1 | 0.563 | 0.999 | 0.994 | 7.51E-139 | 1.92E-134 | Ep-C4 |
| CYC1 | 0.507 | 0.994 | 0.978 | 4.20E-137 | 1.08E-132 | Ep-C4 |
| HSPD1 | 0.561 | 0.999 | 0.987 | 6.72E-137 | 1.72E-132 | Ep-C4 |
| DKC1 | 0.554 | 0.912 | 0.767 | 1.94E-136 | 4.97E-132 | Ep-C4 |
| ETV5 | 0.611 | 0.505 | 0.237 | 3.29E-136 | 8.42E-132 | Ep-C4 |
| IKBIP | 0.516 | 0.568 | 0.277 | 2.90E-133 | 7.42E-129 | Ep-C4 |
| FBXO5 | 0.503 | 0.431 | 0.175 | 8.11E-126 | 2.08E-121 | Ep-C4 |
| CBX5 | 0.528 | 0.689 | 0.422 | 3.78E-125 | 9.69E-121 | Ep-C4 |
| HMG20B | 0.503 | 0.895 | 0.705 | 7.32E-125 | 1.88E-120 | Ep-C4 |
| TOMM40 | 0.527 | 0.941 | 0.824 | 3.53E-124 | 9.05E-120 | Ep-C4 |
| MRPL12 | 0.549 | 0.982 | 0.929 | 4.28E-120 | 1.10E-115 | Ep-C4 |
| HSPH1 | 0.636 | 0.932 | 0.849 | 1.80E-119 | 4.61E-115 | Ep-C4 |
| AIMP2 | 0.517 | 0.688 | 0.45 | 1.70E-117 | 4.37E-113 | Ep-C4 |
| COPS7A | 0.607 | 0.747 | 0.544 | 9.38E-113 | 2.40E-108 | Ep-C4 |
| USP5 | 0.6 | 0.64 | 0.407 | 1.45E-112 | 3.71E-108 | Ep-C4 |
| H2AFJ | 0.787 | 0.963 | 0.939 | 3.78E-111 | 9.69E-107 | Ep-C4 |
| FKBP4 | 0.909 | 0.838 | 0.789 | 1.68E-105 | 4.32E-101 | Ep-C4 |
| EMG1 | 0.619 | 0.872 | 0.734 | 4.04E-105 | 1.04E-100 | Ep-C4 |
| AHSA1 | 0.523 | 0.89 | 0.763 | 6.17E-105 | 1.58E-100 | Ep-C4 |
| SLC12A2 | 0.57 | 0.5 | 0.281 | 9.89E-97 | 2.53E-92 | Ep-C4 |
| SAP25 | 0.693 | 0.316 | 0.13 | 1.10E-96 | 2.81E-92 | Ep-C4 |
| LSM14A | 0.544 | 0.86 | 0.727 | 7.34E-94 | 1.88E-89 | Ep-C4 |
| MRPS35 | 0.514 | 0.854 | 0.725 | 6.93E-93 | 1.78E-88 | Ep-C4 |
| PHB2 | 0.576 | 0.983 | 0.959 | 1.28E-92 | 3.29E-88 | Ep-C4 |
| LDHB | 0.559 | 0.997 | 0.978 | 1.90E-91 | 4.88E-87 | Ep-C4 |
| CCNA1 | 0.551 | 0.314 | 0.121 | 2.08E-91 | 5.32E-87 | Ep-C4 |
| KRT18 | 0.741 | 0.989 | 0.978 | 3.00E-90 | 7.68E-86 | Ep-C4 |
| HIST1H4C | 0.74 | 0.801 | 0.712 | 5.13E-90 | 1.32E-85 | Ep-C4 |
| DNM1L | 0.533 | 0.783 | 0.626 | 7.17E-87 | 1.84E-82 | Ep-C4 |
| FGF19 | 1.584 | 0.308 | 0.126 | 5.28E-86 | 1.35E-81 | Ep-C4 |
| CTNNAL1 | 0.535 | 0.545 | 0.314 | 2.40E-83 | 6.14E-79 | Ep-C4 |
| TIMM50 | 0.58 | 0.75 | 0.593 | 3.39E-83 | 8.70E-79 | Ep-C4 |
| HSPA8 | 0.537 | 0.99 | 0.972 | 7.73E-83 | 1.98E-78 | Ep-C4 |
| ANO1 | 0.767 | 0.699 | 0.613 | 1.70E-82 | 4.35E-78 | Ep-C4 |
| COX8C | 0.708 | 0.274 | 0.101 | 1.52E-79 | 3.90E-75 | Ep-C4 |
| NDUFA9 | 0.519 | 0.878 | 0.784 | 4.70E-79 | 1.21E-74 | Ep-C4 |
| KRT8 | 0.656 | 0.859 | 0.851 | 1.20E-75 | 3.08E-71 | Ep-C4 |
| TBCB | 0.571 | 0.924 | 0.863 | 4.61E-75 | 1.18E-70 | Ep-C4 |
| MEST | 0.557 | 0.536 | 0.335 | 5.52E-74 | 1.41E-69 | Ep-C4 |
| SHKBP1 | 0.624 | 0.642 | 0.484 | 1.07E-73 | 2.74E-69 | Ep-C4 |
| ITPRID2 | 0.588 | 0.814 | 0.675 | 3.40E-73 | 8.71E-69 | Ep-C4 |
| STRAP | 0.514 | 0.962 | 0.924 | 5.46E-72 | 1.40E-67 | Ep-C4 |
| HIST3H2A | 0.781 | 0.618 | 0.563 | 5.51E-72 | 1.41E-67 | Ep-C4 |
| SERPINH1 | 0.574 | 0.754 | 0.579 | 2.13E-71 | 5.45E-67 | Ep-C4 |
| UBA2 | 0.553 | 0.878 | 0.805 | 1.27E-69 | 3.26E-65 | Ep-C4 |
| PLEKHA5 | 0.533 | 0.681 | 0.537 | 1.99E-67 | 5.11E-63 | Ep-C4 |
| COX6B1 | 0.529 | 1 | 0.999 | 1.36E-66 | 3.48E-62 | Ep-C4 |
| LTBR | 0.502 | 0.872 | 0.798 | 4.69E-66 | 1.20E-61 | Ep-C4 |
| CAVIN3 | 0.593 | 0.586 | 0.414 | 7.90E-66 | 2.02E-61 | Ep-C4 |
| FST | 0.547 | 0.722 | 0.55 | 4.10E-63 | 1.05E-58 | Ep-C4 |
| IGFBP4 | 0.509 | 0.821 | 0.692 | 5.31E-62 | 1.36E-57 | Ep-C4 |
| LGALS1 | 0.742 | 0.722 | 0.604 | 1.90E-61 | 4.87E-57 | Ep-C4 |
| DCTN3 | 0.565 | 0.961 | 0.915 | 8.01E-57 | 2.05E-52 | Ep-C4 |
| MRPL21 | 0.789 | 0.93 | 0.923 | 2.35E-51 | 6.04E-47 | Ep-C4 |
| CLDN10 | 0.656 | 0.382 | 0.242 | 4.93E-51 | 1.26E-46 | Ep-C4 |
| MT1E | 0.522 | 0.93 | 0.838 | 3.89E-50 | 9.98E-46 | Ep-C4 |
| SFRP2 | 0.515 | 0.182 | 0.066 | 7.33E-50 | 1.88E-45 | Ep-C4 |
| VIM | 0.674 | 0.621 | 0.51 | 4.52E-47 | 1.16E-42 | Ep-C4 |
| POLR2I | 0.562 | 0.935 | 0.911 | 3.86E-46 | 9.89E-42 | Ep-C4 |
| HSPA6 | 0.575 | 0.365 | 0.217 | 5.40E-40 | 1.38E-35 | Ep-C4 |
| DKK1 | 0.673 | 0.352 | 0.221 | 9.00E-35 | 2.31E-30 | Ep-C4 |
| CCND1 | 0.729 | 0.913 | 0.928 | 1.86E-28 | 4.77E-24 | Ep-C4 |
| LY6D | 2.178 | 0.985 | 0.819 | 0 | 0 | Ep-C5 |
| KRT14 | 1.75 | 0.991 | 0.822 | 0 | 0 | Ep-C5 |
| FABP5 | 1.534 | 1 | 0.997 | 0 | 0 | Ep-C5 |
| KRT5 | 0.99 | 1 | 0.999 | 0 | 0 | Ep-C5 |
| SPON2 | 1.471 | 0.744 | 0.31 | 3.96E-296 | 1.02E-291 | Ep-C5 |
| SELENOP | 1.247 | 0.97 | 0.745 | 1.28E-271 | 3.27E-267 | Ep-C5 |
| S100A16 | 0.791 | 1 | 0.996 | 1.48E-271 | 3.79E-267 | Ep-C5 |
| CA2 | 1.32 | 0.777 | 0.365 | 4.68E-268 | 1.20E-263 | Ep-C5 |
| HSPB1 | 0.725 | 1 | 1 | 2.13E-257 | 5.46E-253 | Ep-C5 |
| PHLDA3 | 0.887 | 0.967 | 0.786 | 4.23E-257 | 1.08E-252 | Ep-C5 |
| LGALS7 | 1.488 | 0.765 | 0.405 | 4.55E-242 | 1.17E-237 | Ep-C5 |
| ATP1B3 | 0.774 | 1 | 0.998 | 7.61E-237 | 1.95E-232 | Ep-C5 |
| DSC3 | 0.733 | 0.985 | 0.881 | 9.35E-215 | 2.40E-210 | Ep-C5 |
| LUM | 1.686 | 0.554 | 0.234 | 1.99E-214 | 5.10E-210 | Ep-C5 |
| COPZ2 | 0.799 | 0.913 | 0.637 | 1.14E-211 | 2.93E-207 | Ep-C5 |
| HSPB3 | 0.917 | 0.468 | 0.133 | 5.53E-209 | 1.42E-204 | Ep-C5 |
| CRABP2 | 1.134 | 0.857 | 0.565 | 2.25E-208 | 5.78E-204 | Ep-C5 |
| S100A14 | 0.893 | 0.993 | 0.946 | 3.14E-207 | 8.04E-203 | Ep-C5 |
| IL20RB | 0.832 | 0.793 | 0.442 | 8.44E-185 | 2.16E-180 | Ep-C5 |
| MT1X | 0.905 | 0.999 | 0.964 | 6.60E-184 | 1.69E-179 | Ep-C5 |
| TSPO | 0.528 | 0.999 | 0.993 | 1.00E-183 | 2.56E-179 | Ep-C5 |
| ATP5IF1 | 0.691 | 0.997 | 0.977 | 2.80E-183 | 7.18E-179 | Ep-C5 |
| SERPINB3 | 0.506 | 0.905 | 0.587 | 2.79E-181 | 7.15E-177 | Ep-C5 |
| DSG3 | 0.651 | 0.976 | 0.807 | 3.20E-177 | 8.20E-173 | Ep-C5 |
| LGALS3 | 0.724 | 0.998 | 0.963 | 1.46E-175 | 3.74E-171 | Ep-C5 |
| S100A4 | 0.938 | 0.855 | 0.588 | 7.99E-164 | 2.05E-159 | Ep-C5 |
| TYMP | 0.776 | 0.995 | 0.948 | 8.96E-163 | 2.30E-158 | Ep-C5 |
| DAPL1 | 0.885 | 0.936 | 0.754 | 1.27E-157 | 3.26E-153 | Ep-C5 |
| SLC25A3 | 0.502 | 0.999 | 0.999 | 1.85E-157 | 4.74E-153 | Ep-C5 |
| TMEM54 | 0.606 | 0.969 | 0.902 | 5.03E-153 | 1.29E-148 | Ep-C5 |
| PRDX1 | 0.558 | 1 | 1 | 8.99E-152 | 2.30E-147 | Ep-C5 |
| IGFBP6 | 1.172 | 0.813 | 0.554 | 2.33E-151 | 5.97E-147 | Ep-C5 |
| PPP2R5A | 0.667 | 0.719 | 0.507 | 3.80E-151 | 9.73E-147 | Ep-C5 |
| LGALS7B | 0.691 | 0.857 | 0.539 | 6.68E-151 | 1.71E-146 | Ep-C5 |
| LAD1 | 0.604 | 0.959 | 0.855 | 2.80E-148 | 7.19E-144 | Ep-C5 |
| EIF3I | 0.543 | 0.993 | 0.975 | 2.59E-145 | 6.63E-141 | Ep-C5 |
| CTSB | 0.69 | 0.993 | 0.959 | 3.63E-144 | 9.30E-140 | Ep-C5 |
| AC100801.1 | 0.909 | 0.349 | 0.095 | 1.03E-142 | 2.63E-138 | Ep-C5 |
| PLS3 | 0.615 | 0.935 | 0.736 | 2.74E-142 | 7.03E-138 | Ep-C5 |
| S100A2 | 0.622 | 1 | 0.996 | 1.18E-140 | 3.03E-136 | Ep-C5 |
| TMPRSS11E | 0.801 | 0.507 | 0.203 | 6.60E-140 | 1.69E-135 | Ep-C5 |
| RBM3 | 0.553 | 0.978 | 0.926 | 2.43E-137 | 6.24E-133 | Ep-C5 |
| GPNMB | 0.639 | 0.972 | 0.832 | 1.58E-135 | 4.04E-131 | Ep-C5 |
| SERPINB13 | 0.546 | 0.73 | 0.402 | 9.48E-135 | 2.43E-130 | Ep-C5 |
| RPS3 | 0.574 | 1 | 1 | 2.59E-134 | 6.63E-130 | Ep-C5 |
| EDIL3 | 0.676 | 0.41 | 0.138 | 3.87E-134 | 9.93E-130 | Ep-C5 |
| CDA | 0.632 | 0.448 | 0.162 | 1.84E-133 | 4.71E-129 | Ep-C5 |
| AL161431.1 | 0.625 | 0.837 | 0.549 | 3.35E-133 | 8.58E-129 | Ep-C5 |
| AC011632.1 | 0.747 | 0.679 | 0.511 | 1.06E-130 | 2.70E-126 | Ep-C5 |
| SLC25A6 | 0.582 | 0.996 | 0.977 | 1.83E-125 | 4.69E-121 | Ep-C5 |
| UPK3BL1 | 0.84 | 0.748 | 0.539 | 2.16E-125 | 5.52E-121 | Ep-C5 |
| CRISPLD1 | 0.782 | 0.491 | 0.24 | 8.14E-125 | 2.09E-120 | Ep-C5 |
| UPK1B | 0.827 | 0.298 | 0.076 | 7.42E-124 | 1.90E-119 | Ep-C5 |
| DSC2 | 0.641 | 0.823 | 0.552 | 9.47E-124 | 2.43E-119 | Ep-C5 |
| NDUFS6 | 0.517 | 0.999 | 0.997 | 1.03E-121 | 2.64E-117 | Ep-C5 |
| IGFL2-AS1 | 0.628 | 0.585 | 0.281 | 1.34E-120 | 3.43E-116 | Ep-C5 |
| MIR99AHG | 0.562 | 0.506 | 0.217 | 6.65E-119 | 1.71E-114 | Ep-C5 |
| DHCR24 | 0.547 | 0.876 | 0.729 | 4.44E-117 | 1.14E-112 | Ep-C5 |
| MT1E | 0.773 | 0.95 | 0.838 | 1.26E-114 | 3.24E-110 | Ep-C5 |
| MT-CO1 | 0.543 | 1 | 1 | 5.36E-113 | 1.37E-108 | Ep-C5 |
| KRT6A | 0.571 | 1 | 0.999 | 5.84E-108 | 1.50E-103 | Ep-C5 |
| SERPINB1 | 0.608 | 0.939 | 0.8 | 1.23E-105 | 3.14E-101 | Ep-C5 |
| DSP | 0.521 | 0.997 | 0.975 | 9.11E-104 | 2.33E-99 | Ep-C5 |
| TPPP3 | 0.608 | 0.522 | 0.255 | 1.10E-102 | 2.83E-98 | Ep-C5 |
| SERPINB9 | 0.581 | 0.516 | 0.261 | 1.41E-102 | 3.62E-98 | Ep-C5 |
| GJA1 | 0.709 | 0.827 | 0.607 | 4.19E-99 | 1.08E-94 | Ep-C5 |
| TMEM265 | 0.561 | 0.785 | 0.534 | 2.14E-97 | 5.49E-93 | Ep-C5 |
| SESN3 | 0.564 | 0.836 | 0.652 | 1.41E-96 | 3.63E-92 | Ep-C5 |
| HDAC1 | 0.519 | 0.941 | 0.894 | 2.47E-96 | 6.32E-92 | Ep-C5 |
| WNT4 | 0.51 | 0.498 | 0.241 | 1.46E-94 | 3.75E-90 | Ep-C5 |
| DMKN | 0.56 | 0.86 | 0.692 | 2.71E-93 | 6.94E-89 | Ep-C5 |
| SULF1 | 0.629 | 0.322 | 0.122 | 6.44E-93 | 1.65E-88 | Ep-C5 |
| CES2 | 0.607 | 0.64 | 0.461 | 4.89E-92 | 1.25E-87 | Ep-C5 |
| PALMD | 0.513 | 0.508 | 0.255 | 9.91E-92 | 2.54E-87 | Ep-C5 |
| DCN | 0.731 | 0.336 | 0.134 | 7.77E-84 | 1.99E-79 | Ep-C5 |
| CSRP2 | 0.538 | 0.875 | 0.759 | 3.43E-82 | 8.80E-78 | Ep-C5 |
| TSKU | 0.541 | 0.682 | 0.526 | 1.30E-81 | 3.34E-77 | Ep-C5 |
| FABP4 | 1.022 | 0.444 | 0.219 | 1.02E-79 | 2.62E-75 | Ep-C5 |
| MAF | 0.585 | 0.519 | 0.302 | 3.60E-78 | 9.22E-74 | Ep-C5 |
| KRT16 | 0.783 | 0.935 | 0.844 | 3.73E-76 | 9.55E-72 | Ep-C5 |
| RBP1 | 0.61 | 0.897 | 0.741 | 5.47E-68 | 1.40E-63 | Ep-C5 |
| THBD | 0.566 | 0.477 | 0.291 | 6.63E-54 | 1.70E-49 | Ep-C5 |
| MME | 0.603 | 0.296 | 0.135 | 4.11E-52 | 1.05E-47 | Ep-C5 |
| BAALC | 0.516 | 0.289 | 0.171 | 2.82E-44 | 7.23E-40 | Ep-C5 |
| HIST1H4C | 2.433 | 0.98 | 0.7 | 0 | 0 | Ep-C6 |
| PCLAF | 2.045 | 0.996 | 0.426 | 0 | 0 | Ep-C6 |
| RRM2 | 2.004 | 0.954 | 0.213 | 0 | 0 | Ep-C6 |
| TYMS | 1.924 | 0.98 | 0.388 | 0 | 0 | Ep-C6 |
| CDK1 | 1.822 | 0.97 | 0.296 | 0 | 0 | Ep-C6 |
| ZWINT | 1.769 | 0.971 | 0.363 | 0 | 0 | Ep-C6 |
| TK1 | 1.726 | 0.997 | 0.525 | 0 | 0 | Ep-C6 |
| CENPM | 1.614 | 0.943 | 0.317 | 0 | 0 | Ep-C6 |
| UBE2T | 1.584 | 0.986 | 0.434 | 0 | 0 | Ep-C6 |
| RAD51AP1 | 1.575 | 0.875 | 0.228 | 0 | 0 | Ep-C6 |
| ATAD2 | 1.563 | 0.945 | 0.361 | 0 | 0 | Ep-C6 |
| TUBA1B | 1.553 | 1 | 0.961 | 0 | 0 | Ep-C6 |
| MYBL2 | 1.533 | 0.931 | 0.241 | 0 | 0 | Ep-C6 |
| HMGB2 | 1.492 | 0.997 | 0.772 | 0 | 0 | Ep-C6 |
| DUT | 1.467 | 0.999 | 0.876 | 0 | 0 | Ep-C6 |
| PCNA | 1.393 | 0.991 | 0.763 | 0 | 0 | Ep-C6 |
| DHFR | 1.346 | 0.897 | 0.275 | 0 | 0 | Ep-C6 |
| RFC4 | 1.315 | 0.964 | 0.511 | 0 | 0 | Ep-C6 |
| TUBB | 1.283 | 1 | 0.98 | 0 | 0 | Ep-C6 |
| STMN1 | 1.282 | 1 | 0.871 | 0 | 0 | Ep-C6 |
| MCM7 | 1.265 | 0.976 | 0.573 | 0 | 0 | Ep-C6 |
| CKS1B | 1.262 | 1 | 0.825 | 0 | 0 | Ep-C6 |
| SPC25 | 1.261 | 0.826 | 0.148 | 0 | 0 | Ep-C6 |
| MAD2L1 | 1.255 | 0.953 | 0.375 | 0 | 0 | Ep-C6 |
| SMC2 | 1.252 | 0.95 | 0.438 | 0 | 0 | Ep-C6 |
| CDC45 | 1.248 | 0.817 | 0.176 | 0 | 0 | Ep-C6 |
| FEN1 | 1.247 | 0.867 | 0.271 | 0 | 0 | Ep-C6 |
| H2AFZ | 1.246 | 1 | 0.996 | 0 | 0 | Ep-C6 |
| CENPX | 1.212 | 0.998 | 0.913 | 0 | 0 | Ep-C6 |
| GINS2 | 1.204 | 0.876 | 0.303 | 0 | 0 | Ep-C6 |
| RANBP1 | 1.201 | 1 | 0.982 | 0 | 0 | Ep-C6 |
| ORC6 | 1.199 | 0.897 | 0.281 | 0 | 0 | Ep-C6 |
| TMEM106C | 1.193 | 0.959 | 0.697 | 0 | 0 | Ep-C6 |
| HIST1H1A | 1.191 | 0.55 | 0.08 | 0 | 0 | Ep-C6 |
| CDCA5 | 1.184 | 0.829 | 0.168 | 0 | 0 | Ep-C6 |
| PBK | 1.178 | 0.855 | 0.203 | 0 | 0 | Ep-C6 |
| CLSPN | 1.176 | 0.779 | 0.194 | 0 | 0 | Ep-C6 |
| TOP2A | 1.157 | 0.944 | 0.324 | 0 | 0 | Ep-C6 |
| PKMYT1 | 1.156 | 0.842 | 0.175 | 0 | 0 | Ep-C6 |
| SMC4 | 1.154 | 0.995 | 0.713 | 0 | 0 | Ep-C6 |
| DNMT1 | 1.146 | 0.955 | 0.554 | 0 | 0 | Ep-C6 |
| CDC6 | 1.139 | 0.805 | 0.187 | 0 | 0 | Ep-C6 |
| ASF1B | 1.137 | 0.815 | 0.174 | 0 | 0 | Ep-C6 |
| MCM3 | 1.136 | 0.912 | 0.439 | 0 | 0 | Ep-C6 |
| CDT1 | 1.133 | 0.852 | 0.26 | 0 | 0 | Ep-C6 |
| RPL39L | 1.132 | 0.996 | 0.753 | 0 | 0 | Ep-C6 |
| NUSAP1 | 1.13 | 0.888 | 0.286 | 0 | 0 | Ep-C6 |
| MKI67 | 1.106 | 0.852 | 0.24 | 0 | 0 | Ep-C6 |
| UBE2C | 1.104 | 0.965 | 0.301 | 0 | 0 | Ep-C6 |
| HELLS | 1.095 | 0.867 | 0.314 | 0 | 0 | Ep-C6 |
| H2AFX | 1.089 | 0.969 | 0.639 | 0 | 0 | Ep-C6 |
| BIRC5 | 1.084 | 0.943 | 0.326 | 0 | 0 | Ep-C6 |
| ESCO2 | 1.067 | 0.766 | 0.121 | 0 | 0 | Ep-C6 |
| FOXM1 | 1.066 | 0.749 | 0.226 | 0 | 0 | Ep-C6 |
| MCM4 | 1.055 | 0.907 | 0.466 | 0 | 0 | Ep-C6 |
| RFC2 | 1.051 | 0.902 | 0.498 | 0 | 0 | Ep-C6 |
| MCM5 | 1.038 | 0.847 | 0.371 | 0 | 0 | Ep-C6 |
| GMNN | 1.035 | 0.871 | 0.425 | 0 | 0 | Ep-C6 |
| CENPK | 1.026 | 0.817 | 0.235 | 0 | 0 | Ep-C6 |
| RPA3 | 1.023 | 0.999 | 0.905 | 0 | 0 | Ep-C6 |
| AURKB | 1.012 | 0.765 | 0.163 | 0 | 0 | Ep-C6 |
| NUDT1 | 0.995 | 0.996 | 0.801 | 0 | 0 | Ep-C6 |
| CENPU | 0.992 | 0.742 | 0.201 | 0 | 0 | Ep-C6 |
| RRM1 | 0.989 | 0.942 | 0.597 | 0 | 0 | Ep-C6 |
| BRCA1 | 0.984 | 0.753 | 0.193 | 0 | 0 | Ep-C6 |
| DTYMK | 0.976 | 0.953 | 0.58 | 0 | 0 | Ep-C6 |
| HMGB1 | 0.953 | 1 | 0.999 | 0 | 0 | Ep-C6 |
| CENPH | 0.946 | 0.829 | 0.336 | 0 | 0 | Ep-C6 |
| TMPO | 0.94 | 0.925 | 0.525 | 0 | 0 | Ep-C6 |
| FANCI | 0.939 | 0.776 | 0.219 | 0 | 0 | Ep-C6 |
| CENPN | 0.931 | 0.835 | 0.34 | 0 | 0 | Ep-C6 |
| DNAJC9 | 0.93 | 0.928 | 0.543 | 0 | 0 | Ep-C6 |
| FBXO5 | 0.93 | 0.696 | 0.16 | 0 | 0 | Ep-C6 |
| CDK4 | 0.915 | 0.984 | 0.792 | 0 | 0 | Ep-C6 |
| MELK | 0.913 | 0.72 | 0.17 | 0 | 0 | Ep-C6 |
| FAM111B | 0.913 | 0.601 | 0.124 | 0 | 0 | Ep-C6 |
| VRK1 | 0.908 | 0.806 | 0.315 | 0 | 0 | Ep-C6 |
| TPX2 | 0.907 | 0.904 | 0.319 | 0 | 0 | Ep-C6 |
| NASP | 0.905 | 0.989 | 0.867 | 0 | 0 | Ep-C6 |
| MND1 | 0.899 | 0.727 | 0.175 | 0 | 0 | Ep-C6 |
| GGH | 0.888 | 0.962 | 0.583 | 0 | 0 | Ep-C6 |
| TUBG1 | 0.883 | 0.896 | 0.473 | 0 | 0 | Ep-C6 |
| LSM4 | 0.88 | 0.997 | 0.945 | 0 | 0 | Ep-C6 |
| NDC80 | 0.865 | 0.703 | 0.144 | 0 | 0 | Ep-C6 |
| ATAD5 | 0.858 | 0.712 | 0.192 | 0 | 0 | Ep-C6 |
| DIAPH3 | 0.843 | 0.612 | 0.127 | 0 | 0 | Ep-C6 |
| PRC1 | 0.84 | 0.847 | 0.287 | 0 | 0 | Ep-C6 |
| ALYREF | 0.84 | 0.984 | 0.829 | 0 | 0 | Ep-C6 |
| CENPF | 0.827 | 0.914 | 0.335 | 0 | 0 | Ep-C6 |
| CDCA3 | 0.822 | 0.687 | 0.163 | 0 | 0 | Ep-C6 |
| TCF19 | 0.816 | 0.656 | 0.155 | 0 | 0 | Ep-C6 |
| DSCC1 | 0.804 | 0.662 | 0.155 | 0 | 0 | Ep-C6 |
| NCAPG | 0.794 | 0.64 | 0.141 | 0 | 0 | Ep-C6 |
| HIST1H1B | 0.782 | 0.456 | 0.054 | 0 | 0 | Ep-C6 |
| NUF2 | 0.759 | 0.782 | 0.192 | 0 | 0 | Ep-C6 |
| CCNA2 | 0.747 | 0.705 | 0.161 | 0 | 0 | Ep-C6 |
| RAD51 | 0.723 | 0.608 | 0.124 | 0 | 0 | Ep-C6 |
| SGO1 | 0.711 | 0.658 | 0.146 | 0 | 0 | Ep-C6 |
| KIF23 | 0.71 | 0.678 | 0.163 | 0 | 0 | Ep-C6 |
| ASPM | 0.625 | 0.752 | 0.203 | 0 | 0 | Ep-C6 |
| RFC5 | 0.79 | 0.755 | 0.274 | 1.72922976044436e-322 | 4.43218879899495e-318 | Ep-C6 |
| NCAPH | 0.751 | 0.619 | 0.138 | 2.37151510003798e-322 | 6.07843035290736e-318 | Ep-C6 |
| ANLN | 0.739 | 0.697 | 0.192 | 1.40205948976829e-319 | 3.5936186782251e-315 | Ep-C6 |
| TEX30 | 0.807 | 0.77 | 0.302 | 5.28094911264208e-318 | 1.35356006706129e-313 | Ep-C6 |
| KIF11 | 0.745 | 0.677 | 0.179 | 1.81620387121808e-315 | 4.65511214231906e-311 | Ep-C6 |
| PSMC3IP | 0.756 | 0.61 | 0.151 | 7.36360076850077e-315 | 1.88736451297443e-310 | Ep-C6 |
| CENPW | 0.97 | 0.987 | 0.675 | 7.50196756996853e-314 | 1.92282930785863e-309 | Ep-C6 |
| LIG1 | 0.762 | 0.705 | 0.226 | 1.00277277423309e-313 | 2.57020689763683e-309 | Ep-C6 |
| SKA3 | 0.695 | 0.608 | 0.142 | 2.00284705148314e-310 | 5.13E-306 | Ep-C6 |
| BRCA2 | 0.719 | 0.64 | 0.161 | 2.64666606740327e-310 | 6.78E-306 | Ep-C6 |
| PRKDC | 0.901 | 0.99 | 0.909 | 8.54282239149206e-310 | 2.19E-305 | Ep-C6 |
| HJURP | 0.607 | 0.533 | 0.099 | 4.07439757854378e-309 | 1.04E-304 | Ep-C6 |
| FANCA | 0.714 | 0.668 | 0.19 | 5.88E-307 | 1.51E-302 | Ep-C6 |
| HIST1H1D | 0.985 | 0.524 | 0.111 | 8.20E-307 | 2.10E-302 | Ep-C6 |
| CHAF1A | 0.776 | 0.742 | 0.252 | 4.34E-305 | 1.11E-300 | Ep-C6 |
| EXO1 | 0.571 | 0.446 | 0.061 | 1.40E-303 | 3.58E-299 | Ep-C6 |
| WDR34 | 0.913 | 0.868 | 0.476 | 1.91E-300 | 4.89E-296 | Ep-C6 |
| KIFC1 | 0.723 | 0.604 | 0.142 | 2.64E-297 | 6.77E-293 | Ep-C6 |
| TFDP1 | 0.776 | 0.964 | 0.703 | 6.25E-297 | 1.60E-292 | Ep-C6 |
| MNS1 | 0.655 | 0.503 | 0.096 | 2.35E-296 | 6.03E-292 | Ep-C6 |
| POLD3 | 0.96 | 0.772 | 0.36 | 2.76E-294 | 7.08E-290 | Ep-C6 |
| DEK | 0.839 | 1 | 0.964 | 8.98E-294 | 2.30E-289 | Ep-C6 |
| MCM10 | 0.631 | 0.499 | 0.09 | 2.76E-292 | 7.08E-288 | Ep-C6 |
| GTSE1 | 0.616 | 0.603 | 0.152 | 3.83E-291 | 9.81E-287 | Ep-C6 |
| FANCB | 0.64 | 0.562 | 0.125 | 6.94E-291 | 1.78E-286 | Ep-C6 |
| TEDC2 | 0.672 | 0.552 | 0.119 | 4.39E-289 | 1.12E-284 | Ep-C6 |
| NCAPD3 | 0.755 | 0.697 | 0.236 | 4.98E-288 | 1.28E-283 | Ep-C6 |
| USP1 | 0.796 | 0.878 | 0.529 | 2.43E-279 | 6.24E-275 | Ep-C6 |
| CDKN3 | 0.615 | 0.807 | 0.315 | 4.82E-279 | 1.24E-274 | Ep-C6 |
| GGCT | 0.771 | 0.993 | 0.928 | 4.23E-278 | 1.08E-273 | Ep-C6 |
| RFC3 | 0.655 | 0.613 | 0.167 | 3.30E-275 | 8.46E-271 | Ep-C6 |
| HIST2H2AC | 0.75 | 0.562 | 0.152 | 6.62E-273 | 1.70E-268 | Ep-C6 |
| SAE1 | 0.765 | 0.901 | 0.538 | 1.14E-272 | 2.92E-268 | Ep-C6 |
| DTL | 0.621 | 0.508 | 0.105 | 3.86E-268 | 9.89E-264 | Ep-C6 |
| SLBP | 0.81 | 0.936 | 0.711 | 2.31E-266 | 5.93E-262 | Ep-C6 |
| CEP55 | 0.523 | 0.648 | 0.203 | 1.47E-265 | 3.78E-261 | Ep-C6 |
| CKLF | 0.852 | 0.859 | 0.498 | 5.55E-265 | 1.42E-260 | Ep-C6 |
| CIP2A | 0.648 | 0.642 | 0.187 | 1.77E-264 | 4.53E-260 | Ep-C6 |
| KIF15 | 0.577 | 0.508 | 0.104 | 1.07E-263 | 2.73E-259 | Ep-C6 |
| MCM2 | 0.772 | 0.759 | 0.298 | 8.94E-263 | 2.29E-258 | Ep-C6 |
| FAM111A | 0.807 | 0.707 | 0.285 | 1.78E-262 | 4.56E-258 | Ep-C6 |
| TRIP13 | 0.694 | 0.687 | 0.225 | 9.51E-262 | 2.44E-257 | Ep-C6 |
| PTMA | 0.674 | 1 | 1 | 1.30E-261 | 3.32E-257 | Ep-C6 |
| KIF2C | 0.615 | 0.577 | 0.149 | 2.02E-260 | 5.17E-256 | Ep-C6 |
| DSN1 | 0.715 | 0.698 | 0.255 | 3.30E-259 | 8.46E-255 | Ep-C6 |
| ECT2 | 0.723 | 0.867 | 0.403 | 2.35E-258 | 6.02E-254 | Ep-C6 |
| TACC3 | 0.613 | 0.608 | 0.17 | 2.04E-255 | 5.23E-251 | Ep-C6 |
| KNL1 | 0.571 | 0.562 | 0.142 | 5.35E-253 | 1.37E-248 | Ep-C6 |
| CDCA4 | 1.262 | 0.933 | 0.588 | 6.62E-253 | 1.70E-248 | Ep-C6 |
| SUPT16H | 0.818 | 0.922 | 0.659 | 1.15E-251 | 2.95E-247 | Ep-C6 |
| PRIM1 | 0.697 | 0.661 | 0.237 | 1.60E-250 | 4.11E-246 | Ep-C6 |
| TEDC1 | 0.753 | 0.736 | 0.283 | 3.86E-250 | 9.90E-246 | Ep-C6 |
| CACYBP | 0.725 | 0.996 | 0.929 | 4.02E-250 | 1.03E-245 | Ep-C6 |
| SNRNP25 | 0.743 | 0.928 | 0.629 | 4.07E-249 | 1.04E-244 | Ep-C6 |
| RECQL4 | 0.601 | 0.539 | 0.133 | 1.55E-247 | 3.96E-243 | Ep-C6 |
| CCDC34 | 0.769 | 0.82 | 0.4 | 2.57E-245 | 6.60E-241 | Ep-C6 |
| LRRCC1 | 0.81 | 0.738 | 0.312 | 1.09E-244 | 2.80E-240 | Ep-C6 |
| LDHB | 1.064 | 1 | 0.978 | 1.24E-244 | 3.17E-240 | Ep-C6 |
| MTFR2 | 0.523 | 0.474 | 0.096 | 7.74E-244 | 1.98E-239 | Ep-C6 |
| PXMP2 | 0.803 | 0.832 | 0.502 | 1.67E-242 | 4.28E-238 | Ep-C6 |
| ITGB3BP | 0.695 | 0.737 | 0.315 | 4.69E-240 | 1.20E-235 | Ep-C6 |
| KIF20B | 0.615 | 0.751 | 0.294 | 1.18E-239 | 3.04E-235 | Ep-C6 |
| CDCA8 | 0.511 | 0.545 | 0.146 | 1.54E-239 | 3.94E-235 | Ep-C6 |
| MTHFD1 | 0.747 | 0.72 | 0.313 | 1.33E-238 | 3.40E-234 | Ep-C6 |
| PSMC3 | 0.655 | 0.991 | 0.911 | 5.37E-238 | 1.38E-233 | Ep-C6 |
| HSP90AA1 | 0.956 | 1 | 1 | 7.39E-238 | 1.89E-233 | Ep-C6 |
| HMGN2 | 0.796 | 1 | 0.966 | 9.69E-238 | 2.48E-233 | Ep-C6 |
| H2AFV | 0.724 | 0.997 | 0.962 | 8.54E-237 | 2.19E-232 | Ep-C6 |
| POLA2 | 0.602 | 0.562 | 0.16 | 7.70E-235 | 1.97E-230 | Ep-C6 |
| RMI2 | 0.699 | 0.652 | 0.233 | 1.01E-234 | 2.60E-230 | Ep-C6 |
| LMNB1 | 0.701 | 0.699 | 0.262 | 5.34E-231 | 1.37E-226 | Ep-C6 |
| TTK | 0.525 | 0.541 | 0.144 | 1.65E-230 | 4.22E-226 | Ep-C6 |
| LRR1 | 0.644 | 0.637 | 0.22 | 2.25E-230 | 5.76E-226 | Ep-C6 |
| MASTL | 0.599 | 0.522 | 0.139 | 3.03E-228 | 7.76E-224 | Ep-C6 |
| MIS18BP1 | 0.774 | 0.788 | 0.39 | 7.67E-228 | 1.97E-223 | Ep-C6 |
| PSIP1 | 0.745 | 0.833 | 0.475 | 1.84E-227 | 4.71E-223 | Ep-C6 |
| LSM3 | 0.619 | 0.996 | 0.935 | 1.63E-225 | 4.18E-221 | Ep-C6 |
| PA2G4 | 0.641 | 0.999 | 0.969 | 1.52E-224 | 3.88E-220 | Ep-C6 |
| E2F8 | 0.519 | 0.413 | 0.077 | 1.73E-223 | 4.43E-219 | Ep-C6 |
| C21orf58 | 0.525 | 0.47 | 0.107 | 2.04E-223 | 5.24E-219 | Ep-C6 |
| GMPS | 0.674 | 0.979 | 0.887 | 2.05E-223 | 5.26E-219 | Ep-C6 |
| E2F1 | 0.652 | 0.559 | 0.161 | 2.37E-223 | 6.07E-219 | Ep-C6 |
| SPC24 | 0.527 | 0.456 | 0.1 | 4.51E-223 | 1.16E-218 | Ep-C6 |
| CKAP2L | 0.55 | 0.509 | 0.129 | 6.24E-222 | 1.60E-217 | Ep-C6 |
| CCNE2 | 0.768 | 0.497 | 0.134 | 8.73E-222 | 2.24E-217 | Ep-C6 |
| RBBP7 | 0.684 | 0.931 | 0.669 | 5.63E-221 | 1.44E-216 | Ep-C6 |
| HPRT1 | 0.681 | 0.957 | 0.772 | 1.74E-220 | 4.45E-216 | Ep-C6 |
| RACGAP1 | 0.551 | 0.616 | 0.201 | 6.32E-219 | 1.62E-214 | Ep-C6 |
| TIMELESS | 0.586 | 0.585 | 0.184 | 8.38E-219 | 2.15E-214 | Ep-C6 |
| NCAPG2 | 0.558 | 0.518 | 0.142 | 5.36E-218 | 1.37E-213 | Ep-C6 |
| SCCPDH | 0.722 | 0.828 | 0.464 | 1.44E-217 | 3.69E-213 | Ep-C6 |
| E2F7 | 0.535 | 0.468 | 0.108 | 5.03E-217 | 1.29E-212 | Ep-C6 |
| RAD51C | 0.665 | 0.779 | 0.394 | 1.32E-216 | 3.38E-212 | Ep-C6 |
| CSE1L | 0.692 | 0.866 | 0.523 | 2.18E-216 | 5.58E-212 | Ep-C6 |
| UBR7 | 0.637 | 0.69 | 0.296 | 3.21E-216 | 8.23E-212 | Ep-C6 |
| IGFLR1 | 0.817 | 0.527 | 0.161 | 1.07E-214 | 2.74E-210 | Ep-C6 |
| SAC3D1 | 0.722 | 0.806 | 0.445 | 1.64E-214 | 4.20E-210 | Ep-C6 |
| KIF22 | 0.686 | 0.816 | 0.408 | 2.10E-214 | 5.37E-210 | Ep-C6 |
| POLD1 | 0.569 | 0.568 | 0.177 | 1.24E-213 | 3.18E-209 | Ep-C6 |
| SHCBP1 | 0.526 | 0.499 | 0.126 | 9.38E-213 | 2.40E-208 | Ep-C6 |
| DDX11 | 0.614 | 0.565 | 0.177 | 5.96E-212 | 1.53E-207 | Ep-C6 |
| SNRPG | 0.679 | 1 | 0.991 | 6.56E-212 | 1.68E-207 | Ep-C6 |
| GINS1 | 0.516 | 0.466 | 0.115 | 1.84E-211 | 4.71E-207 | Ep-C6 |
| MIS18A | 0.637 | 0.717 | 0.302 | 3.04E-209 | 7.79E-205 | Ep-C6 |
| POLR3K | 0.747 | 0.829 | 0.495 | 5.51E-209 | 1.41E-204 | Ep-C6 |
| HAT1 | 0.69 | 0.921 | 0.696 | 1.58E-206 | 4.04E-202 | Ep-C6 |
| COMMD4 | 0.682 | 0.93 | 0.733 | 4.49E-204 | 1.15E-199 | Ep-C6 |
| POC1A | 0.543 | 0.555 | 0.169 | 2.04E-203 | 5.24E-199 | Ep-C6 |
| BCL2L12 | 0.633 | 0.811 | 0.408 | 2.39E-202 | 6.13E-198 | Ep-C6 |
| CMC2 | 0.709 | 0.892 | 0.66 | 3.28E-202 | 8.41E-198 | Ep-C6 |
| CKS2 | 0.694 | 0.981 | 0.77 | 2.89E-201 | 7.41E-197 | Ep-C6 |
| WDR76 | 0.548 | 0.507 | 0.144 | 2.96E-201 | 7.59E-197 | Ep-C6 |
| PRIM2 | 0.648 | 0.655 | 0.275 | 4.12E-201 | 1.06E-196 | Ep-C6 |
| SNRPD1 | 0.646 | 0.999 | 0.978 | 4.43E-201 | 1.14E-196 | Ep-C6 |
| SASS6 | 0.572 | 0.604 | 0.214 | 5.79E-201 | 1.48E-196 | Ep-C6 |
| SMC1A | 0.721 | 0.814 | 0.459 | 3.55E-200 | 9.10E-196 | Ep-C6 |
| UHRF1 | 0.562 | 0.523 | 0.151 | 4.04E-200 | 1.04E-195 | Ep-C6 |
| ANP32B | 0.597 | 1 | 0.994 | 5.24E-200 | 1.34E-195 | Ep-C6 |
| NDUFAF8 | 0.628 | 0.997 | 0.941 | 2.66E-199 | 6.81E-195 | Ep-C6 |
| ERH | 0.61 | 1 | 0.981 | 6.63E-199 | 1.70E-194 | Ep-C6 |
| RECQL | 0.777 | 0.768 | 0.416 | 1.35E-198 | 3.46E-194 | Ep-C6 |
| PAXX | 0.621 | 0.964 | 0.778 | 1.12E-197 | 2.87E-193 | Ep-C6 |
| CHEK1 | 0.628 | 0.631 | 0.241 | 3.82E-196 | 9.80E-192 | Ep-C6 |
| HNRNPAB | 0.612 | 0.993 | 0.917 | 7.93E-196 | 2.03E-191 | Ep-C6 |
| SMC3 | 0.63 | 0.959 | 0.786 | 3.50E-195 | 8.98E-191 | Ep-C6 |
| MAGOHB | 0.757 | 0.868 | 0.591 | 4.93E-192 | 1.26E-187 | Ep-C6 |
| MCM8 | 0.516 | 0.506 | 0.149 | 4.96E-191 | 1.27E-186 | Ep-C6 |
| ATP5MC1 | 0.65 | 0.999 | 0.987 | 1.08E-190 | 2.77E-186 | Ep-C6 |
| HIRIP3 | 0.634 | 0.625 | 0.256 | 2.01E-190 | 5.14E-186 | Ep-C6 |
| PARP1 | 0.682 | 0.956 | 0.786 | 2.68E-188 | 6.87E-184 | Ep-C6 |
| PAICS | 0.624 | 0.939 | 0.703 | 3.76E-188 | 9.64E-184 | Ep-C6 |
| TYMSOS | 0.592 | 0.587 | 0.206 | 2.22E-187 | 5.69E-183 | Ep-C6 |
| RHNO1 | 0.76 | 0.67 | 0.317 | 4.53E-187 | 1.16E-182 | Ep-C6 |
| PTN | 0.831 | 0.905 | 0.634 | 1.16E-186 | 2.97E-182 | Ep-C6 |
| TMEM97 | 0.693 | 0.731 | 0.364 | 1.18E-186 | 3.01E-182 | Ep-C6 |
| CD320 | 0.701 | 0.842 | 0.536 | 1.31E-185 | 3.36E-181 | Ep-C6 |
| WDHD1 | 0.514 | 0.517 | 0.158 | 2.90E-185 | 7.45E-181 | Ep-C6 |
| CHTF18 | 0.507 | 0.51 | 0.152 | 3.75E-185 | 9.62E-181 | Ep-C6 |
| PGP | 0.713 | 0.909 | 0.619 | 2.47E-184 | 6.32E-180 | Ep-C6 |
| NCAPD2 | 0.747 | 0.673 | 0.279 | 4.10E-184 | 1.05E-179 | Ep-C6 |
| SKP2 | 0.568 | 0.63 | 0.26 | 8.67E-184 | 2.22E-179 | Ep-C6 |
| SSRP1 | 0.596 | 0.944 | 0.736 | 4.10E-182 | 1.05E-177 | Ep-C6 |
| GPN3 | 0.605 | 0.839 | 0.507 | 9.00E-182 | 2.31E-177 | Ep-C6 |
| TONSL | 0.505 | 0.477 | 0.134 | 2.04E-181 | 5.23E-177 | Ep-C6 |
| HSPB11 | 0.625 | 0.913 | 0.724 | 2.38E-180 | 6.11E-176 | Ep-C6 |
| CDCA7L | 0.588 | 0.677 | 0.298 | 1.51E-179 | 3.88E-175 | Ep-C6 |
| ZWILCH | 0.505 | 0.555 | 0.191 | 6.00E-178 | 1.54E-173 | Ep-C6 |
| PTGES3 | 0.507 | 1 | 0.998 | 1.01E-177 | 2.60E-173 | Ep-C6 |
| C19orf48 | 0.641 | 0.924 | 0.637 | 6.98E-177 | 1.79E-172 | Ep-C6 |
| CYC1 | 0.624 | 0.998 | 0.978 | 7.61E-177 | 1.95E-172 | Ep-C6 |
| HSPE1 | 0.68 | 1 | 0.994 | 3.54E-176 | 9.08E-172 | Ep-C6 |
| CENPS | 0.611 | 0.677 | 0.32 | 3.47E-175 | 8.90E-171 | Ep-C6 |
| TOMM40 | 0.669 | 0.965 | 0.824 | 1.55E-174 | 3.97E-170 | Ep-C6 |
| NAP1L1 | 0.581 | 1 | 0.98 | 1.75E-173 | 4.50E-169 | Ep-C6 |
| RNASEH2B | 0.615 | 0.806 | 0.494 | 3.40E-173 | 8.71E-169 | Ep-C6 |
| CEP78 | 0.54 | 0.565 | 0.208 | 3.55E-173 | 9.10E-169 | Ep-C6 |
| TTF2 | 0.591 | 0.674 | 0.304 | 3.61E-173 | 9.25E-169 | Ep-C6 |
| TOPBP1 | 0.636 | 0.782 | 0.446 | 9.71E-173 | 2.49E-168 | Ep-C6 |
| DDX39A | 0.641 | 0.923 | 0.667 | 4.44E-171 | 1.14E-166 | Ep-C6 |
| AP2S1 | 0.538 | 0.999 | 0.991 | 5.89E-171 | 1.51E-166 | Ep-C6 |
| PAFAH1B3 | 0.603 | 0.954 | 0.786 | 7.10E-170 | 1.82E-165 | Ep-C6 |
| BARD1 | 0.584 | 0.648 | 0.282 | 1.25E-169 | 3.20E-165 | Ep-C6 |
| HADH | 0.571 | 0.823 | 0.491 | 1.63E-169 | 4.17E-165 | Ep-C6 |
| LRRC45 | 0.523 | 0.553 | 0.206 | 1.73E-169 | 4.45E-165 | Ep-C6 |
| ANP32E | 0.645 | 0.958 | 0.772 | 8.84E-169 | 2.26E-164 | Ep-C6 |
| RAN | 0.521 | 1 | 0.999 | 1.69E-167 | 4.32E-163 | Ep-C6 |
| MRPL51 | 0.756 | 0.998 | 0.987 | 2.17E-167 | 5.55E-163 | Ep-C6 |
| RPA2 | 0.641 | 0.753 | 0.445 | 1.32E-166 | 3.38E-162 | Ep-C6 |
| CBX5 | 0.631 | 0.77 | 0.422 | 5.13E-166 | 1.31E-161 | Ep-C6 |
| PHGDH | 0.777 | 0.776 | 0.443 | 5.31E-166 | 1.36E-161 | Ep-C6 |
| HSPD1 | 0.641 | 0.999 | 0.987 | 6.00E-166 | 1.54E-161 | Ep-C6 |
| HIST1H1E | 0.54 | 0.444 | 0.136 | 8.41E-166 | 2.16E-161 | Ep-C6 |
| SHMT1 | 0.543 | 0.66 | 0.295 | 1.32E-165 | 3.40E-161 | Ep-C6 |
| CENPQ | 0.53 | 0.504 | 0.168 | 4.15E-165 | 1.06E-160 | Ep-C6 |
| EZH2 | 0.643 | 0.696 | 0.362 | 2.95E-163 | 7.56E-159 | Ep-C6 |
| ACTL6A | 0.572 | 0.982 | 0.893 | 7.07E-162 | 1.81E-157 | Ep-C6 |
| UNG | 0.722 | 0.658 | 0.355 | 4.41E-161 | 1.13E-156 | Ep-C6 |
| KPNA2 | 0.504 | 0.917 | 0.615 | 4.99E-160 | 1.28E-155 | Ep-C6 |
| CMSS1 | 0.625 | 0.927 | 0.698 | 5.36E-160 | 1.37E-155 | Ep-C6 |
| COPS3 | 0.562 | 0.922 | 0.723 | 7.07E-160 | 1.81E-155 | Ep-C6 |
| ACAT2 | 0.668 | 0.915 | 0.693 | 9.51E-160 | 2.44E-155 | Ep-C6 |
| NUDC | 0.565 | 0.984 | 0.924 | 4.49E-159 | 1.15E-154 | Ep-C6 |
| HAUS1 | 0.551 | 0.655 | 0.316 | 3.83E-158 | 9.80E-154 | Ep-C6 |
| FANCG | 0.542 | 0.511 | 0.182 | 5.18E-158 | 1.33E-153 | Ep-C6 |
| LMNB2 | 0.609 | 0.786 | 0.433 | 8.85E-158 | 2.27E-153 | Ep-C6 |
| CDK2 | 0.506 | 0.548 | 0.207 | 9.85E-158 | 2.52E-153 | Ep-C6 |
| YEATS4 | 0.585 | 0.773 | 0.44 | 2.15E-157 | 5.50E-153 | Ep-C6 |
| HIST3H2A | 1.247 | 0.701 | 0.558 | 2.88E-156 | 7.38E-152 | Ep-C6 |
| ANAPC11 | 0.578 | 0.998 | 0.976 | 1.04E-155 | 2.66E-151 | Ep-C6 |
| HNRNPD | 0.508 | 0.996 | 0.971 | 6.02E-155 | 1.54E-150 | Ep-C6 |
| SRSF10 | 0.529 | 0.974 | 0.864 | 2.51E-153 | 6.44E-149 | Ep-C6 |
| BRI3BP | 0.578 | 0.737 | 0.396 | 2.55E-151 | 6.54E-147 | Ep-C6 |
| SLC25A11 | 0.552 | 0.93 | 0.764 | 4.57E-151 | 1.17E-146 | Ep-C6 |
| XRCC5 | 0.524 | 0.99 | 0.948 | 5.86E-151 | 1.50E-146 | Ep-C6 |
| OXCT1 | 0.562 | 0.7 | 0.37 | 3.48E-150 | 8.93E-146 | Ep-C6 |
| HAUS5 | 0.595 | 0.511 | 0.202 | 2.62E-147 | 6.72E-143 | Ep-C6 |
| AKR7A2 | 0.569 | 0.936 | 0.773 | 7.89E-147 | 2.02E-142 | Ep-C6 |
| UBA2 | 0.767 | 0.93 | 0.802 | 1.25E-146 | 3.20E-142 | Ep-C6 |
| NCAPH2 | 0.553 | 0.649 | 0.318 | 1.78E-146 | 4.56E-142 | Ep-C6 |
| PAQR4 | 0.599 | 0.586 | 0.251 | 2.02E-146 | 5.17E-142 | Ep-C6 |
| RFWD3 | 0.501 | 0.552 | 0.228 | 6.18E-146 | 1.58E-141 | Ep-C6 |
| DCTPP1 | 0.614 | 0.938 | 0.794 | 9.25E-146 | 2.37E-141 | Ep-C6 |
| ILF2 | 0.506 | 0.993 | 0.954 | 8.06E-144 | 2.07E-139 | Ep-C6 |
| RNPS1 | 0.547 | 0.988 | 0.924 | 1.77E-143 | 4.54E-139 | Ep-C6 |
| GCSH | 0.648 | 0.924 | 0.82 | 2.70E-143 | 6.91E-139 | Ep-C6 |
| POLD2 | 0.51 | 0.965 | 0.836 | 4.71E-143 | 1.21E-138 | Ep-C6 |
| MRPL37 | 0.516 | 0.908 | 0.674 | 7.83E-143 | 2.01E-138 | Ep-C6 |
| NDUFA6 | 0.509 | 0.992 | 0.947 | 2.57E-142 | 6.60E-138 | Ep-C6 |
| HNRNPA3 | 0.549 | 1 | 0.998 | 8.93E-142 | 2.29E-137 | Ep-C6 |
| TCOF1 | 0.55 | 0.692 | 0.354 | 1.46E-141 | 3.75E-137 | Ep-C6 |
| HIST1H3G | 0.511 | 0.334 | 0.077 | 3.18E-141 | 8.14E-137 | Ep-C6 |
| TUBB4B | 0.578 | 0.999 | 0.991 | 4.00E-141 | 1.03E-136 | Ep-C6 |
| EXOSC8 | 0.546 | 0.827 | 0.535 | 4.65E-141 | 1.19E-136 | Ep-C6 |
| MCM6 | 0.578 | 0.626 | 0.294 | 8.43E-141 | 2.16E-136 | Ep-C6 |
| ACYP1 | 0.6 | 0.781 | 0.469 | 1.90E-140 | 4.87E-136 | Ep-C6 |
| NUDT15 | 0.573 | 0.779 | 0.482 | 4.52E-139 | 1.16E-134 | Ep-C6 |
| TRAP1 | 0.581 | 0.88 | 0.652 | 9.57E-139 | 2.45E-134 | Ep-C6 |
| POLE3 | 0.547 | 0.9 | 0.671 | 9.89E-138 | 2.54E-133 | Ep-C6 |
| MRPL17 | 0.553 | 0.919 | 0.753 | 2.42E-136 | 6.20E-132 | Ep-C6 |
| GPAA1 | 0.573 | 0.924 | 0.768 | 3.17E-136 | 8.14E-132 | Ep-C6 |
| C5orf34 | 0.553 | 0.359 | 0.102 | 1.60E-135 | 4.09E-131 | Ep-C6 |
| SAP30 | 0.546 | 0.743 | 0.413 | 2.96E-135 | 7.59E-131 | Ep-C6 |
| FN3KRP | 0.531 | 0.723 | 0.408 | 5.44E-135 | 1.40E-130 | Ep-C6 |
| TOMM5 | 0.563 | 0.995 | 0.966 | 1.34E-134 | 3.44E-130 | Ep-C6 |
| NSD2 | 0.504 | 0.624 | 0.293 | 1.36E-133 | 3.49E-129 | Ep-C6 |
| DBF4 | 0.51 | 0.679 | 0.339 | 1.01E-132 | 2.60E-128 | Ep-C6 |
| TMEM107 | 0.513 | 0.694 | 0.365 | 3.49E-132 | 8.95E-128 | Ep-C6 |
| XRCC6 | 0.553 | 0.994 | 0.961 | 1.96E-131 | 5.02E-127 | Ep-C6 |
| HNRNPUL1 | 0.754 | 0.896 | 0.742 | 6.57E-131 | 1.68E-126 | Ep-C6 |
| CISD1 | 0.582 | 0.936 | 0.78 | 8.74E-131 | 2.24E-126 | Ep-C6 |
| SHMT2 | 0.532 | 0.894 | 0.659 | 1.29E-130 | 3.30E-126 | Ep-C6 |
| NETO2 | 0.534 | 0.61 | 0.294 | 4.21E-130 | 1.08E-125 | Ep-C6 |
| RAD21 | 0.539 | 0.981 | 0.882 | 2.14E-129 | 5.47E-125 | Ep-C6 |
| MRPL12 | 0.596 | 0.984 | 0.93 | 3.26E-128 | 8.37E-124 | Ep-C6 |
| TPI1 | 0.605 | 1 | 1 | 5.97E-127 | 1.53E-122 | Ep-C6 |
| HIST1H2BJ | 0.934 | 0.439 | 0.195 | 1.05E-125 | 2.69E-121 | Ep-C6 |
| CENPV | 0.502 | 0.584 | 0.276 | 1.74E-122 | 4.46E-118 | Ep-C6 |
| EMG1 | 0.771 | 0.866 | 0.737 | 3.07E-121 | 7.88E-117 | Ep-C6 |
| PRPS2 | 0.524 | 0.746 | 0.445 | 5.19E-121 | 1.33E-116 | Ep-C6 |
| RRP7A | 0.669 | 0.814 | 0.604 | 3.11E-120 | 7.98E-116 | Ep-C6 |
| RBBP8 | 0.566 | 0.875 | 0.61 | 7.10E-120 | 1.82E-115 | Ep-C6 |
| SNRPA | 0.528 | 0.869 | 0.627 | 9.12E-120 | 2.34E-115 | Ep-C6 |
| POLR2I | 0.847 | 0.955 | 0.91 | 1.97E-118 | 5.04E-114 | Ep-C6 |
| PEG10 | 0.665 | 0.401 | 0.136 | 9.31E-117 | 2.39E-112 | Ep-C6 |
| NMRAL1 | 0.515 | 0.843 | 0.649 | 3.56E-116 | 9.12E-112 | Ep-C6 |
| MSH2 | 0.556 | 0.579 | 0.298 | 5.12E-116 | 1.31E-111 | Ep-C6 |
| TOR3A | 0.502 | 0.729 | 0.437 | 1.82E-114 | 4.66E-110 | Ep-C6 |
| CTNNAL1 | 0.662 | 0.621 | 0.313 | 2.69E-114 | 6.90E-110 | Ep-C6 |
| TMEM38B | 0.504 | 0.649 | 0.354 | 2.60E-112 | 6.66E-108 | Ep-C6 |
| FDPS | 0.514 | 0.989 | 0.924 | 5.25E-112 | 1.35E-107 | Ep-C6 |
| ARL6IP6 | 0.562 | 0.77 | 0.498 | 6.29E-112 | 1.61E-107 | Ep-C6 |
| TIMM10 | 0.515 | 0.909 | 0.731 | 8.81E-111 | 2.26E-106 | Ep-C6 |
| PNKP | 0.515 | 0.728 | 0.461 | 1.81E-109 | 4.63E-105 | Ep-C6 |
| C1orf35 | 0.522 | 0.857 | 0.668 | 1.66E-108 | 4.25E-104 | Ep-C6 |
| FBL | 0.537 | 0.98 | 0.925 | 3.18E-106 | 8.16E-102 | Ep-C6 |
| SIVA1 | 0.972 | 1 | 0.96 | 8.35E-105 | 2.14E-100 | Ep-C6 |
| MTHFD2 | 0.523 | 0.808 | 0.547 | 8.37E-104 | 2.15E-99 | Ep-C6 |
| POLR2H | 0.509 | 0.995 | 0.957 | 8.88E-104 | 2.28E-99 | Ep-C6 |
| TIMM50 | 0.7 | 0.802 | 0.592 | 2.39E-103 | 6.14E-99 | Ep-C6 |
| PAK1 | 0.671 | 0.763 | 0.542 | 4.86E-103 | 1.25E-98 | Ep-C6 |
| FH | 0.509 | 0.818 | 0.592 | 5.58E-102 | 1.43E-97 | Ep-C6 |
| KRT18 | 0.855 | 0.986 | 0.978 | 1.85E-101 | 4.73E-97 | Ep-C6 |
| NDUFA9 | 0.666 | 0.898 | 0.785 | 2.90E-99 | 7.42E-95 | Ep-C6 |
| PDCD5 | 0.592 | 0.988 | 0.972 | 1.10E-97 | 2.82E-93 | Ep-C6 |
| PSAT1 | 0.52 | 0.696 | 0.417 | 5.04E-95 | 1.29E-90 | Ep-C6 |
| STIP1 | 0.505 | 0.862 | 0.704 | 5.12E-95 | 1.31E-90 | Ep-C6 |
| RNASEH2C | 0.67 | 0.894 | 0.739 | 1.15E-94 | 2.96E-90 | Ep-C6 |
| SVIP | 0.584 | 0.528 | 0.326 | 4.42E-92 | 1.13E-87 | Ep-C6 |
| HLTF | 0.516 | 0.854 | 0.671 | 2.08E-91 | 5.34E-87 | Ep-C6 |
| GNG4 | 0.562 | 0.331 | 0.121 | 3.93E-90 | 1.01E-85 | Ep-C6 |
| KRT8 | 0.718 | 0.886 | 0.849 | 1.73E-88 | 4.44E-84 | Ep-C6 |
| LY6E | 0.51 | 0.999 | 0.986 | 2.27E-88 | 5.82E-84 | Ep-C6 |
| NCL | 0.502 | 0.996 | 0.978 | 3.19E-88 | 8.17E-84 | Ep-C6 |
| ERGIC2 | 0.526 | 0.89 | 0.787 | 9.83E-88 | 2.52E-83 | Ep-C6 |
| FKBP4 | 0.916 | 0.847 | 0.789 | 1.79E-87 | 4.59E-83 | Ep-C6 |
| MRPS12 | 0.583 | 0.965 | 0.932 | 2.70E-86 | 6.91E-82 | Ep-C6 |
| WBP11 | 0.597 | 0.778 | 0.573 | 1.62E-85 | 4.16E-81 | Ep-C6 |
| AHSA1 | 0.52 | 0.899 | 0.765 | 3.54E-84 | 9.09E-80 | Ep-C6 |
| USP5 | 0.531 | 0.652 | 0.411 | 1.02E-82 | 2.61E-78 | Ep-C6 |
| MAGEA1 | 0.5 | 0.494 | 0.271 | 5.12E-82 | 1.31E-77 | Ep-C6 |
| TCTEX1D2 | 0.506 | 0.847 | 0.715 | 2.82E-81 | 7.23E-77 | Ep-C6 |
| EXOSC5 | 0.504 | 0.8 | 0.598 | 2.93E-81 | 7.51E-77 | Ep-C6 |
| STXBP6 | 0.57 | 0.411 | 0.175 | 2.95E-81 | 7.57E-77 | Ep-C6 |
| MRPL21 | 0.988 | 0.959 | 0.921 | 3.49E-81 | 8.95E-77 | Ep-C6 |
| MEST | 0.592 | 0.573 | 0.336 | 1.79E-79 | 4.59E-75 | Ep-C6 |
| H1FX | 0.596 | 0.998 | 0.991 | 7.49E-78 | 1.92E-73 | Ep-C6 |
| PHB2 | 0.624 | 0.985 | 0.959 | 2.22E-76 | 5.70E-72 | Ep-C6 |
| EPS8 | 0.524 | 0.613 | 0.363 | 8.70E-76 | 2.23E-71 | Ep-C6 |
| MAGEB2 | 0.542 | 0.275 | 0.094 | 3.33E-73 | 8.54E-69 | Ep-C6 |
| NFKBID | 0.56 | 0.459 | 0.237 | 5.99E-73 | 1.54E-68 | Ep-C6 |
| DHCR7 | 0.728 | 0.759 | 0.6 | 1.05E-72 | 2.70E-68 | Ep-C6 |
| H2AFJ | 0.747 | 0.957 | 0.94 | 6.25E-69 | 1.60E-64 | Ep-C6 |
| HEBP1 | 0.516 | 0.749 | 0.587 | 1.22E-68 | 3.12E-64 | Ep-C6 |
| COX8C | 0.663 | 0.287 | 0.104 | 1.30E-68 | 3.33E-64 | Ep-C6 |
| GTSF1 | 0.521 | 0.272 | 0.098 | 3.21E-68 | 8.24E-64 | Ep-C6 |
| UCHL1 | 0.652 | 0.518 | 0.288 | 8.61E-65 | 2.21E-60 | Ep-C6 |
| DNM1L | 0.521 | 0.787 | 0.629 | 1.37E-64 | 3.52E-60 | Ep-C6 |
| COX6B1 | 0.584 | 0.999 | 0.999 | 1.82E-64 | 4.67E-60 | Ep-C6 |
| FHL1 | 0.504 | 0.248 | 0.087 | 6.92E-64 | 1.77E-59 | Ep-C6 |
| FGF19 | 1.155 | 0.316 | 0.129 | 6.26E-63 | 1.60E-58 | Ep-C6 |
| SFRP2 | 0.608 | 0.214 | 0.066 | 4.15E-61 | 1.06E-56 | Ep-C6 |
| SIGMAR1 | 0.521 | 0.768 | 0.561 | 5.56E-61 | 1.42E-56 | Ep-C6 |
| HEY1 | 0.592 | 0.592 | 0.369 | 8.59E-61 | 2.20E-56 | Ep-C6 |
| TBCB | 0.535 | 0.899 | 0.866 | 1.59E-60 | 4.07E-56 | Ep-C6 |
| M6PR | 0.523 | 0.816 | 0.715 | 3.63E-58 | 9.31E-54 | Ep-C6 |
| SAP25 | 0.533 | 0.309 | 0.134 | 1.15E-57 | 2.95E-53 | Ep-C6 |
| IGHMBP2 | 0.52 | 0.505 | 0.315 | 2.36E-57 | 6.05E-53 | Ep-C6 |
| MT-ND6 | 0.596 | 0.702 | 0.575 | 1.74E-56 | 4.47E-52 | Ep-C6 |
| SHKBP1 | 0.592 | 0.631 | 0.488 | 2.95E-55 | 7.56E-51 | Ep-C6 |
| RBM42 | 0.575 | 0.761 | 0.653 | 5.89E-54 | 1.51E-49 | Ep-C6 |
| NUDT8 | 0.61 | 0.834 | 0.7 | 2.26E-53 | 5.80E-49 | Ep-C6 |
| HIST1H1C | 0.57 | 0.824 | 0.662 | 4.72E-51 | 1.21E-46 | Ep-C6 |
| HSPA6 | 0.651 | 0.394 | 0.217 | 7.09E-44 | 1.82E-39 | Ep-C6 |
| EPCAM | 0.577 | 0.924 | 0.837 | 4.03E-42 | 1.03E-37 | Ep-C6 |
| H1F0 | 0.542 | 0.774 | 0.743 | 1.56E-41 | 4.00E-37 | Ep-C6 |
| ITPRID2 | 0.502 | 0.768 | 0.681 | 5.44E-38 | 1.40E-33 | Ep-C6 |
| CRABP1 | 0.52 | 0.214 | 0.167 | 9.44E-38 | 2.42E-33 | Ep-C6 |
| NTS | 0.651 | 0.475 | 0.462 | 2.28E-17 | 5.83E-13 | Ep-C6 |
| LAMC2 | 3.323 | 0.993 | 0.477 | 0 | 0 | Ep-C7 |
| TGFBI | 3.14 | 0.901 | 0.287 | 0 | 0 | Ep-C7 |
| P3H2 | 2.895 | 0.808 | 0.242 | 0 | 0 | Ep-C7 |
| MT2A | 2.877 | 0.983 | 0.908 | 0 | 0 | Ep-C7 |
| CAV1 | 2.467 | 0.991 | 0.61 | 0 | 0 | Ep-C7 |
| LGALS1 | 2.384 | 0.975 | 0.59 | 0 | 0 | Ep-C7 |
| COL17A1 | 2.339 | 0.953 | 0.441 | 0 | 0 | Ep-C7 |
| INHBA | 2.257 | 0.76 | 0.178 | 0 | 0 | Ep-C7 |
| LAMB3 | 2.242 | 0.987 | 0.779 | 0 | 0 | Ep-C7 |
| PMEPA1 | 2.229 | 0.966 | 0.471 | 0 | 0 | Ep-C7 |
| PDPN | 2.186 | 0.779 | 0.269 | 0 | 0 | Ep-C7 |
| LAMA3 | 2.138 | 0.925 | 0.38 | 0 | 0 | Ep-C7 |
| VIM | 2.09 | 0.915 | 0.493 | 0 | 0 | Ep-C7 |
| COL5A2 | 2.057 | 0.658 | 0.131 | 0 | 0 | Ep-C7 |
| IGFBP6 | 2.01 | 0.954 | 0.549 | 0 | 0 | Ep-C7 |
| MMP2 | 1.992 | 0.861 | 0.226 | 0 | 0 | Ep-C7 |
| EMP3 | 1.846 | 0.935 | 0.369 | 0 | 0 | Ep-C7 |
| CTSV | 1.784 | 0.842 | 0.367 | 0 | 0 | Ep-C7 |
| FLNA | 1.77 | 0.994 | 0.762 | 0 | 0 | Ep-C7 |
| TNFRSF12A | 1.77 | 0.978 | 0.686 | 0 | 0 | Ep-C7 |
| ANO1 | 1.765 | 0.96 | 0.598 | 0 | 0 | Ep-C7 |
| SEMA3C | 1.735 | 0.774 | 0.267 | 0 | 0 | Ep-C7 |
| SERPINE2 | 1.717 | 0.942 | 0.557 | 0 | 0 | Ep-C7 |
| SERINC2 | 1.706 | 0.931 | 0.642 | 0 | 0 | Ep-C7 |
| SERPINH1 | 1.667 | 0.935 | 0.571 | 0 | 0 | Ep-C7 |
| PRSS23 | 1.658 | 0.76 | 0.215 | 0 | 0 | Ep-C7 |
| COL12A1 | 1.602 | 0.847 | 0.241 | 0 | 0 | Ep-C7 |
| CAVIN3 | 1.547 | 0.934 | 0.395 | 0 | 0 | Ep-C7 |
| COL4A2 | 1.486 | 0.849 | 0.327 | 0 | 0 | Ep-C7 |
| PTHLH | 1.473 | 0.965 | 0.591 | 0 | 0 | Ep-C7 |
| ANXA5 | 1.449 | 0.996 | 0.903 | 0 | 0 | Ep-C7 |
| MFAP5 | 1.416 | 0.425 | 0.04 | 0 | 0 | Ep-C7 |
| ITGA6 | 1.409 | 0.99 | 0.827 | 0 | 0 | Ep-C7 |
| ITGB4 | 1.389 | 0.998 | 0.858 | 0 | 0 | Ep-C7 |
| AMIGO2 | 1.352 | 0.653 | 0.127 | 0 | 0 | Ep-C7 |
| COL4A1 | 1.349 | 0.755 | 0.263 | 0 | 0 | Ep-C7 |
| LPCAT2 | 1.349 | 0.826 | 0.44 | 0 | 0 | Ep-C7 |
| MMP28 | 1.349 | 0.795 | 0.263 | 0 | 0 | Ep-C7 |
| DAAM1 | 1.338 | 0.861 | 0.537 | 0 | 0 | Ep-C7 |
| CRIP2 | 1.308 | 0.844 | 0.328 | 0 | 0 | Ep-C7 |
| ACTN1 | 1.304 | 0.978 | 0.689 | 0 | 0 | Ep-C7 |
| PLEK2 | 1.302 | 0.818 | 0.384 | 0 | 0 | Ep-C7 |
| CALR | 1.293 | 1 | 0.998 | 0 | 0 | Ep-C7 |
| ITGB6 | 1.277 | 0.735 | 0.255 | 0 | 0 | Ep-C7 |
| CAVIN1 | 1.272 | 0.968 | 0.643 | 0 | 0 | Ep-C7 |
| FLRT2 | 1.253 | 0.619 | 0.129 | 0 | 0 | Ep-C7 |
| ITGA3 | 1.251 | 0.939 | 0.561 | 0 | 0 | Ep-C7 |
| FSTL3 | 1.243 | 0.682 | 0.145 | 0 | 0 | Ep-C7 |
| S100A10 | 1.241 | 1 | 0.998 | 0 | 0 | Ep-C7 |
| CD59 | 1.239 | 0.997 | 0.924 | 0 | 0 | Ep-C7 |
| COL7A1 | 1.234 | 0.919 | 0.515 | 0 | 0 | Ep-C7 |
| ARPC1B | 1.233 | 1 | 0.958 | 0 | 0 | Ep-C7 |
| PLS3 | 1.225 | 0.983 | 0.736 | 0 | 0 | Ep-C7 |
| CD151 | 1.209 | 0.998 | 0.918 | 0 | 0 | Ep-C7 |
| GALNT2 | 1.193 | 0.871 | 0.449 | 0 | 0 | Ep-C7 |
| PLEC | 1.182 | 0.987 | 0.859 | 0 | 0 | Ep-C7 |
| ACTB | 1.167 | 1 | 1 | 0 | 0 | Ep-C7 |
| MYH9 | 1.101 | 0.994 | 0.897 | 0 | 0 | Ep-C7 |
| PKM | 1.094 | 1 | 0.999 | 0 | 0 | Ep-C7 |
| P4HA2 | 1.083 | 0.814 | 0.369 | 0 | 0 | Ep-C7 |
| MSC | 1.064 | 0.567 | 0.103 | 0 | 0 | Ep-C7 |
| PDIA3 | 1.044 | 0.998 | 0.986 | 0 | 0 | Ep-C7 |
| ITGB1 | 1.03 | 0.976 | 0.835 | 0 | 0 | Ep-C7 |
| CDK6 | 0.993 | 0.931 | 0.653 | 0 | 0 | Ep-C7 |
| FSCN1 | 0.968 | 1 | 0.947 | 0 | 0 | Ep-C7 |
| CSPG4 | 0.946 | 0.533 | 0.083 | 0 | 0 | Ep-C7 |
| WDR66 | 0.943 | 0.946 | 0.67 | 0 | 0 | Ep-C7 |
| ANXA2 | 0.912 | 1 | 1 | 0 | 0 | Ep-C7 |
| TUBB6 | 1.117 | 0.916 | 0.679 | 6.91691904177745e-323 | 1.77287551959798e-318 | Ep-C7 |
| SPCS2 | 0.901 | 0.998 | 0.99 | 1.28457067918724e-322 | 3.29248310782482e-318 | Ep-C7 |
| NDFIP2 | 1.076 | 0.925 | 0.722 | 5.23709584591721e-321 | 1.34232003626704e-316 | Ep-C7 |
| TMSB10 | 0.843 | 1 | 1 | 2.87941458396278e-320 | 7.38022752015501e-316 | Ep-C7 |
| FAP | 0.635 | 0.339 | 0.018 | 4.6013321728487e-319 | 1.17936744922285e-314 | Ep-C7 |
| P4HB | 0.926 | 1 | 0.999 | 5.11925130807116e-318 | 1.31211530277172e-313 | Ep-C7 |
| FXYD5 | 1.196 | 0.937 | 0.584 | 1.35514183077572e-314 | 3.47336402646125e-310 | Ep-C7 |
| SERPINE1 | 1.735 | 0.72 | 0.244 | 2.01170113072695e-313 | 5.15619116816625e-309 | Ep-C7 |
| ITGAV | 0.998 | 0.851 | 0.489 | 9.96904429995701e-311 | 2.56E-306 | Ep-C7 |
| S100A2 | 1.31 | 1 | 0.996 | 9.87529614117531e-309 | 2.53E-304 | Ep-C7 |
| MYL9 | 1.256 | 0.749 | 0.319 | 1.55228381700771e-308 | 3.98E-304 | Ep-C7 |
| KDELR2 | 0.89 | 0.998 | 0.98 | 2.12E-307 | 5.43E-303 | Ep-C7 |
| PRNP | 1.01 | 0.987 | 0.874 | 4.23E-303 | 1.08E-298 | Ep-C7 |
| ITGA5 | 1.207 | 0.682 | 0.207 | 7.81E-301 | 2.00E-296 | Ep-C7 |
| OCIAD2 | 0.902 | 0.993 | 0.954 | 4.37E-300 | 1.12E-295 | Ep-C7 |
| SLITRK6 | 1.14 | 0.626 | 0.151 | 3.19E-299 | 8.18E-295 | Ep-C7 |
| TGM2 | 1.334 | 0.337 | 0.029 | 4.50E-296 | 1.15E-291 | Ep-C7 |
| KTN1 | 0.88 | 1 | 0.982 | 2.18E-295 | 5.60E-291 | Ep-C7 |
| SPCS3 | 0.854 | 0.959 | 0.801 | 7.57E-295 | 1.94E-290 | Ep-C7 |
| MT1E | 1.523 | 0.969 | 0.838 | 6.89E-294 | 1.77E-289 | Ep-C7 |
| CAP1 | 0.875 | 0.981 | 0.896 | 1.48E-293 | 3.79E-289 | Ep-C7 |
| CDH3 | 1.086 | 0.892 | 0.578 | 1.84E-293 | 4.71E-289 | Ep-C7 |
| LAMC1 | 0.936 | 0.778 | 0.382 | 2.15E-292 | 5.52E-288 | Ep-C7 |
| PDGFA | 1.058 | 0.852 | 0.468 | 4.34E-291 | 1.11E-286 | Ep-C7 |
| MMP13 | 1.614 | 0.462 | 0.066 | 3.69E-285 | 9.45E-281 | Ep-C7 |
| PPIB | 0.887 | 0.999 | 0.992 | 1.93E-284 | 4.96E-280 | Ep-C7 |
| GALNT1 | 1.075 | 0.942 | 0.689 | 1.26E-279 | 3.23E-275 | Ep-C7 |
| CYB5R3 | 0.825 | 0.969 | 0.805 | 6.77E-276 | 1.73E-271 | Ep-C7 |
| USB1 | 0.9 | 0.765 | 0.495 | 9.55E-272 | 2.45E-267 | Ep-C7 |
| KDELR3 | 0.883 | 0.663 | 0.292 | 1.55E-270 | 3.97E-266 | Ep-C7 |
| CAV2 | 0.994 | 0.97 | 0.72 | 1.21E-269 | 3.10E-265 | Ep-C7 |
| APP | 0.913 | 0.994 | 0.924 | 2.13E-268 | 5.47E-264 | Ep-C7 |
| HSP90B1 | 0.914 | 1 | 0.992 | 3.79E-264 | 9.70E-260 | Ep-C7 |
| AREG | 1.757 | 0.805 | 0.408 | 1.65E-263 | 4.24E-259 | Ep-C7 |
| TPM3 | 0.741 | 0.999 | 0.988 | 3.82E-263 | 9.78E-259 | Ep-C7 |
| LOXL2 | 0.888 | 0.498 | 0.094 | 8.39E-263 | 2.15E-258 | Ep-C7 |
| LGALS7 | 1.651 | 0.756 | 0.411 | 1.64E-262 | 4.22E-258 | Ep-C7 |
| IL7R | 0.915 | 0.328 | 0.029 | 1.76E-262 | 4.52E-258 | Ep-C7 |
| VEGFC | 0.628 | 0.318 | 0.027 | 1.75E-255 | 4.49E-251 | Ep-C7 |
| GUK1 | 0.796 | 1 | 0.981 | 1.00E-253 | 2.57E-249 | Ep-C7 |
| COL4A6 | 0.793 | 0.609 | 0.175 | 1.31E-253 | 3.36E-249 | Ep-C7 |
| TENM2 | 1.011 | 0.791 | 0.379 | 8.56E-253 | 2.19E-248 | Ep-C7 |
| CD63 | 0.874 | 1 | 0.997 | 3.04E-252 | 7.79E-248 | Ep-C7 |
| TMED9 | 0.778 | 0.988 | 0.934 | 8.07E-252 | 2.07E-247 | Ep-C7 |
| SLC38A5 | 0.829 | 0.48 | 0.117 | 1.37E-250 | 3.52E-246 | Ep-C7 |
| RRAS | 0.864 | 0.937 | 0.66 | 1.12E-249 | 2.88E-245 | Ep-C7 |
| TINAGL1 | 1.124 | 0.884 | 0.606 | 1.58E-249 | 4.06E-245 | Ep-C7 |
| COPZ2 | 1.01 | 0.881 | 0.644 | 1.36E-247 | 3.49E-243 | Ep-C7 |
| UPP1 | 1.262 | 0.898 | 0.675 | 1.94E-247 | 4.97E-243 | Ep-C7 |
| ITGB5 | 0.875 | 0.756 | 0.406 | 7.18E-247 | 1.84E-242 | Ep-C7 |
| CRELD2 | 0.914 | 0.768 | 0.497 | 3.12E-246 | 7.99E-242 | Ep-C7 |
| TGFBR2 | 0.709 | 0.513 | 0.121 | 3.40E-246 | 8.70E-242 | Ep-C7 |
| PFN1 | 0.712 | 1 | 0.999 | 1.81E-245 | 4.63E-241 | Ep-C7 |
| FHL2 | 0.918 | 0.89 | 0.549 | 8.01E-244 | 2.05E-239 | Ep-C7 |
| FERMT1 | 1.166 | 0.851 | 0.61 | 1.48E-243 | 3.79E-239 | Ep-C7 |
| MYDGF | 0.73 | 0.992 | 0.956 | 4.08E-243 | 1.05E-238 | Ep-C7 |
| PDIA6 | 0.797 | 0.999 | 0.995 | 8.94E-243 | 2.29E-238 | Ep-C7 |
| HSPA5 | 0.97 | 1 | 0.989 | 2.30E-241 | 5.90E-237 | Ep-C7 |
| GPC1 | 0.861 | 0.972 | 0.88 | 1.68E-239 | 4.30E-235 | Ep-C7 |
| KIFC3 | 0.779 | 0.566 | 0.174 | 3.27E-239 | 8.38E-235 | Ep-C7 |
| APLP2 | 0.829 | 0.983 | 0.894 | 1.20E-232 | 3.07E-228 | Ep-C7 |
| PDGFC | 0.735 | 0.488 | 0.109 | 7.37E-232 | 1.89E-227 | Ep-C7 |
| WNT10A | 0.848 | 0.605 | 0.188 | 2.98E-229 | 7.63E-225 | Ep-C7 |
| CD44 | 0.815 | 0.999 | 0.977 | 1.98E-228 | 5.08E-224 | Ep-C7 |
| LUM | 1.814 | 0.653 | 0.233 | 3.44E-228 | 8.83E-224 | Ep-C7 |
| BCAP31 | 0.718 | 0.997 | 0.982 | 9.55E-227 | 2.45E-222 | Ep-C7 |
| C19orf33 | 0.907 | 0.994 | 0.885 | 2.42E-225 | 6.20E-221 | Ep-C7 |
| IKBIP | 0.803 | 0.682 | 0.277 | 1.54E-224 | 3.95E-220 | Ep-C7 |
| TRAM1 | 0.77 | 0.927 | 0.779 | 5.48E-224 | 1.41E-219 | Ep-C7 |
| ATP1B1 | 0.902 | 0.999 | 0.974 | 4.03E-223 | 1.03E-218 | Ep-C7 |
| SPON2 | 1.448 | 0.713 | 0.319 | 4.24E-223 | 1.09E-218 | Ep-C7 |
| SSR3 | 0.752 | 0.997 | 0.981 | 6.94E-223 | 1.78E-218 | Ep-C7 |
| YBX1 | 0.814 | 1 | 0.999 | 7.06E-221 | 1.81E-216 | Ep-C7 |
| MFSD10 | 0.801 | 0.907 | 0.669 | 1.39E-220 | 3.56E-216 | Ep-C7 |
| IVNS1ABP | 1.03 | 0.844 | 0.669 | 6.74E-220 | 1.73E-215 | Ep-C7 |
| MSN | 0.812 | 0.969 | 0.75 | 1.63E-219 | 4.19E-215 | Ep-C7 |
| NELL2 | 0.879 | 0.6 | 0.233 | 3.15E-218 | 8.07E-214 | Ep-C7 |
| CD109 | 0.845 | 0.805 | 0.435 | 7.51E-217 | 1.93E-212 | Ep-C7 |
| BMP1 | 0.707 | 0.619 | 0.235 | 3.72E-215 | 9.52E-211 | Ep-C7 |
| NEFL | 1.202 | 0.514 | 0.138 | 6.75E-215 | 1.73E-210 | Ep-C7 |
| TMSB4X | 0.963 | 1 | 0.999 | 2.81E-213 | 7.19E-209 | Ep-C7 |
| HCFC1R1 | 0.866 | 0.85 | 0.661 | 5.38E-212 | 1.38E-207 | Ep-C7 |
| MYO1B | 0.827 | 0.83 | 0.469 | 1.40E-211 | 3.60E-207 | Ep-C7 |
| GJA1 | 1.12 | 0.919 | 0.604 | 4.93E-211 | 1.26E-206 | Ep-C7 |
| BASP1 | 1.04 | 0.711 | 0.292 | 1.09E-210 | 2.78E-206 | Ep-C7 |
| MYL12B | 0.556 | 1 | 1 | 6.95E-210 | 1.78E-205 | Ep-C7 |
| ARNTL2 | 0.852 | 0.687 | 0.328 | 1.30E-209 | 3.33E-205 | Ep-C7 |
| ETS1 | 0.608 | 0.374 | 0.061 | 6.84E-209 | 1.75E-204 | Ep-C7 |
| TMEM208 | 0.667 | 0.988 | 0.922 | 1.30E-207 | 3.32E-203 | Ep-C7 |
| CTSZ | 0.742 | 0.939 | 0.762 | 2.65E-204 | 6.79E-200 | Ep-C7 |
| EIF3I | 0.732 | 0.993 | 0.975 | 5.63E-204 | 1.44E-199 | Ep-C7 |
| MSC-AS1 | 0.65 | 0.459 | 0.104 | 1.33E-202 | 3.41E-198 | Ep-C7 |
| CANX | 0.767 | 0.978 | 0.937 | 1.32E-200 | 3.37E-196 | Ep-C7 |
| HLA-B | 1.281 | 0.996 | 0.98 | 2.83E-199 | 7.25E-195 | Ep-C7 |
| PYGL | 0.852 | 0.722 | 0.374 | 3.73E-199 | 9.57E-195 | Ep-C7 |
| ARL6IP5 | 0.765 | 0.881 | 0.649 | 1.89E-197 | 4.84E-193 | Ep-C7 |
| PDLIM7 | 0.777 | 0.673 | 0.324 | 2.97E-197 | 7.60E-193 | Ep-C7 |
| TAGLN | 1.147 | 0.404 | 0.102 | 9.40E-195 | 2.41E-190 | Ep-C7 |
| EHD2 | 0.721 | 0.738 | 0.36 | 1.31E-194 | 3.37E-190 | Ep-C7 |
| CTNNAL1 | 0.974 | 0.692 | 0.311 | 3.88E-194 | 9.95E-190 | Ep-C7 |
| LRPAP1 | 0.687 | 0.945 | 0.781 | 4.23E-193 | 1.08E-188 | Ep-C7 |
| SH3BGRL3 | 0.69 | 1 | 0.995 | 1.23E-190 | 3.16E-186 | Ep-C7 |
| GPR87 | 0.851 | 0.85 | 0.536 | 2.95E-190 | 7.56E-186 | Ep-C7 |
| NRG1 | 0.921 | 0.578 | 0.23 | 5.10E-190 | 1.31E-185 | Ep-C7 |
| KRT6A | 0.856 | 1 | 0.999 | 4.92E-189 | 1.26E-184 | Ep-C7 |
| PLOD3 | 0.686 | 0.624 | 0.26 | 7.49E-188 | 1.92E-183 | Ep-C7 |
| MAGEA10 | 0.683 | 0.523 | 0.148 | 1.03E-186 | 2.65E-182 | Ep-C7 |
| LOX | 0.986 | 0.421 | 0.119 | 4.36E-186 | 1.12E-181 | Ep-C7 |
| KC877982.1 | 0.851 | 0.372 | 0.07 | 4.53E-186 | 1.16E-181 | Ep-C7 |
| SSR4 | 0.72 | 1 | 0.993 | 2.55E-185 | 6.54E-181 | Ep-C7 |
| HSP90AB1 | 0.672 | 1 | 1 | 1.81E-184 | 4.64E-180 | Ep-C7 |
| SDC4 | 0.882 | 0.968 | 0.832 | 3.74E-184 | 9.60E-180 | Ep-C7 |
| AC011632.1 | 0.918 | 0.782 | 0.507 | 3.58E-183 | 9.17E-179 | Ep-C7 |
| UFD1 | 0.653 | 0.955 | 0.814 | 1.72E-181 | 4.40E-177 | Ep-C7 |
| CALD1 | 0.924 | 0.83 | 0.478 | 4.95E-181 | 1.27E-176 | Ep-C7 |
| SELENOW | 0.645 | 1 | 0.995 | 5.65E-181 | 1.45E-176 | Ep-C7 |
| CD47 | 0.74 | 0.982 | 0.918 | 2.95E-180 | 7.55E-176 | Ep-C7 |
| MDFI | 0.768 | 0.769 | 0.48 | 3.47E-180 | 8.88E-176 | Ep-C7 |
| SDF2L1 | 0.781 | 0.93 | 0.767 | 9.82E-178 | 2.52E-173 | Ep-C7 |
| TSKU | 0.845 | 0.79 | 0.521 | 1.25E-177 | 3.20E-173 | Ep-C7 |
| SYT8 | 0.8 | 0.507 | 0.158 | 2.09E-177 | 5.35E-173 | Ep-C7 |
| TNFRSF6B | 0.999 | 0.566 | 0.193 | 4.24E-177 | 1.09E-172 | Ep-C7 |
| TAGLN2 | 0.628 | 1 | 0.997 | 8.06E-177 | 2.07E-172 | Ep-C7 |
| HMGA2 | 0.768 | 0.508 | 0.177 | 1.30E-176 | 3.34E-172 | Ep-C7 |
| ANXA8 | 0.715 | 0.708 | 0.347 | 1.99E-176 | 5.10E-172 | Ep-C7 |
| IL20RB | 1.019 | 0.734 | 0.452 | 3.82E-173 | 9.79E-169 | Ep-C7 |
| MANF | 0.73 | 0.914 | 0.758 | 3.72E-172 | 9.53E-168 | Ep-C7 |
| AC100801.1 | 0.915 | 0.416 | 0.095 | 1.19E-170 | 3.05E-166 | Ep-C7 |
| NT5E | 0.649 | 0.399 | 0.116 | 2.30E-170 | 5.89E-166 | Ep-C7 |
| TGFB1I1 | 0.586 | 0.514 | 0.169 | 1.46E-169 | 3.74E-165 | Ep-C7 |
| YWHAZ | 0.519 | 1 | 1 | 8.69E-169 | 2.23E-164 | Ep-C7 |
| PLAU | 1.191 | 0.883 | 0.673 | 1.85E-168 | 4.74E-164 | Ep-C7 |
| FKBP11 | 0.707 | 0.764 | 0.449 | 9.18E-168 | 2.35E-163 | Ep-C7 |
| CRIP1 | 0.912 | 0.944 | 0.751 | 1.13E-167 | 2.90E-163 | Ep-C7 |
| CRISPLD1 | 0.85 | 0.598 | 0.237 | 2.78E-167 | 7.13E-163 | Ep-C7 |
| GLG1 | 0.652 | 0.909 | 0.692 | 5.01E-167 | 1.28E-162 | Ep-C7 |
| MAP7D1 | 0.742 | 0.867 | 0.599 | 8.83E-167 | 2.26E-162 | Ep-C7 |
| TYMP | 0.919 | 0.995 | 0.949 | 9.89E-167 | 2.54E-162 | Ep-C7 |
| IRAK1 | 0.689 | 0.835 | 0.569 | 1.75E-165 | 4.49E-161 | Ep-C7 |
| EDIL3 | 0.72 | 0.485 | 0.138 | 2.98E-165 | 7.63E-161 | Ep-C7 |
| KRT17 | 0.705 | 1 | 0.934 | 4.29E-164 | 1.10E-159 | Ep-C7 |
| RPN2 | 0.616 | 0.995 | 0.968 | 5.56E-164 | 1.42E-159 | Ep-C7 |
| COL27A1 | 0.674 | 0.621 | 0.274 | 2.12E-163 | 5.43E-159 | Ep-C7 |
| HSPB3 | 0.834 | 0.476 | 0.138 | 2.82E-163 | 7.23E-159 | Ep-C7 |
| AGTRAP | 0.676 | 0.939 | 0.797 | 5.79E-162 | 1.48E-157 | Ep-C7 |
| NDUFS6 | 0.642 | 0.999 | 0.997 | 1.83E-161 | 4.69E-157 | Ep-C7 |
| GLS | 0.781 | 0.774 | 0.508 | 2.66E-161 | 6.82E-157 | Ep-C7 |
| MAP4K4 | 0.796 | 0.83 | 0.584 | 2.90E-161 | 7.43E-157 | Ep-C7 |
| PPT1 | 0.714 | 0.856 | 0.63 | 4.84E-161 | 1.24E-156 | Ep-C7 |
| BSG | 0.57 | 0.998 | 0.989 | 5.41E-161 | 1.39E-156 | Ep-C7 |
| LMNA | 0.635 | 0.999 | 0.996 | 7.79E-161 | 2.00E-156 | Ep-C7 |
| RPN1 | 0.706 | 0.91 | 0.787 | 1.00E-160 | 2.56E-156 | Ep-C7 |
| INO80C | 0.764 | 0.714 | 0.421 | 3.98E-160 | 1.02E-155 | Ep-C7 |
| PPFIBP1 | 0.74 | 0.855 | 0.581 | 7.09E-160 | 1.82E-155 | Ep-C7 |
| CDH13 | 0.69 | 0.664 | 0.32 | 8.95E-160 | 2.29E-155 | Ep-C7 |
| LAMP2 | 0.763 | 0.925 | 0.783 | 1.74E-159 | 4.46E-155 | Ep-C7 |
| GJB3 | 0.709 | 0.881 | 0.598 | 2.80E-159 | 7.18E-155 | Ep-C7 |
| PLP2 | 0.602 | 0.999 | 0.987 | 4.35E-159 | 1.12E-154 | Ep-C7 |
| DKK3 | 0.763 | 0.647 | 0.322 | 8.17E-159 | 2.09E-154 | Ep-C7 |
| EREG | 0.997 | 0.38 | 0.084 | 2.60E-158 | 6.66E-154 | Ep-C7 |
| DNAJC8 | 0.64 | 0.962 | 0.879 | 1.95E-157 | 5.00E-153 | Ep-C7 |
| CAST | 0.78 | 0.995 | 0.918 | 2.33E-157 | 5.96E-153 | Ep-C7 |
| TNC | 1.066 | 0.735 | 0.375 | 2.80E-157 | 7.17E-153 | Ep-C7 |
| DSG2 | 0.72 | 0.861 | 0.588 | 4.10E-156 | 1.05E-151 | Ep-C7 |
| TM9SF2 | 0.642 | 0.876 | 0.651 | 7.15E-156 | 1.83E-151 | Ep-C7 |
| PTMS | 0.736 | 0.998 | 0.969 | 1.80E-154 | 4.61E-150 | Ep-C7 |
| SRPX | 0.646 | 0.331 | 0.067 | 9.97E-154 | 2.55E-149 | Ep-C7 |
| MAF | 0.813 | 0.647 | 0.297 | 5.16E-153 | 1.32E-148 | Ep-C7 |
| HOMER3 | 0.713 | 0.84 | 0.57 | 8.40E-153 | 2.15E-148 | Ep-C7 |
| MARVELD1 | 0.613 | 0.594 | 0.247 | 7.22E-152 | 1.85E-147 | Ep-C7 |
| SULF2 | 0.755 | 0.797 | 0.485 | 9.19E-152 | 2.36E-147 | Ep-C7 |
| POPDC3 | 0.571 | 0.488 | 0.154 | 1.36E-151 | 3.49E-147 | Ep-C7 |
| NPC2 | 0.641 | 0.992 | 0.931 | 7.80E-151 | 2.00E-146 | Ep-C7 |
| TUBA4A | 0.747 | 0.936 | 0.789 | 1.14E-150 | 2.92E-146 | Ep-C7 |
| GPX8 | 0.563 | 0.512 | 0.183 | 1.48E-150 | 3.80E-146 | Ep-C7 |
| MAP4 | 0.634 | 0.834 | 0.591 | 3.44E-150 | 8.81E-146 | Ep-C7 |
| TGFB1 | 0.818 | 0.863 | 0.612 | 5.63E-150 | 1.44E-145 | Ep-C7 |
| RHOD | 0.733 | 0.895 | 0.714 | 6.04E-150 | 1.55E-145 | Ep-C7 |
| ELOVL1 | 0.637 | 0.852 | 0.595 | 8.81E-150 | 2.26E-145 | Ep-C7 |
| HTRA1 | 0.689 | 0.592 | 0.273 | 1.08E-149 | 2.77E-145 | Ep-C7 |
| P3H1 | 0.518 | 0.451 | 0.152 | 1.38E-149 | 3.55E-145 | Ep-C7 |
| TMED10 | 0.633 | 0.994 | 0.97 | 5.44E-149 | 1.40E-144 | Ep-C7 |
| ARHGDIA | 0.552 | 0.988 | 0.928 | 5.89E-149 | 1.51E-144 | Ep-C7 |
| CLPTM1L | 0.586 | 0.925 | 0.782 | 3.58E-148 | 9.19E-144 | Ep-C7 |
| CALM3 | 0.644 | 0.933 | 0.792 | 8.55E-148 | 2.19E-143 | Ep-C7 |
| ECM1 | 0.713 | 0.585 | 0.233 | 1.08E-147 | 2.77E-143 | Ep-C7 |
| SPATS2L | 0.624 | 0.888 | 0.652 | 2.85E-147 | 7.31E-143 | Ep-C7 |
| EFEMP1 | 0.646 | 0.389 | 0.11 | 9.64E-147 | 2.47E-142 | Ep-C7 |
| LIMA1 | 0.729 | 0.941 | 0.75 | 1.09E-146 | 2.81E-142 | Ep-C7 |
| ORMDL2 | 0.599 | 0.945 | 0.778 | 1.41E-146 | 3.62E-142 | Ep-C7 |
| ATP6V1D | 0.6 | 0.886 | 0.745 | 6.26E-146 | 1.60E-141 | Ep-C7 |
| KRT14 | 1.34 | 0.935 | 0.829 | 7.37E-146 | 1.89E-141 | Ep-C7 |
| FRMD6 | 0.69 | 0.789 | 0.51 | 1.40E-145 | 3.59E-141 | Ep-C7 |
| PABPC4 | 0.679 | 0.881 | 0.772 | 1.53E-145 | 3.93E-141 | Ep-C7 |
| TAX1BP3 | 0.62 | 0.963 | 0.819 | 2.30E-145 | 5.90E-141 | Ep-C7 |
| ARF4 | 0.621 | 0.978 | 0.917 | 6.11E-144 | 1.57E-139 | Ep-C7 |
| DNAJC3 | 0.645 | 0.869 | 0.693 | 7.75E-144 | 1.99E-139 | Ep-C7 |
| PGAM1 | 0.578 | 1 | 0.981 | 3.03E-143 | 7.78E-139 | Ep-C7 |
| MMP9 | 1.05 | 0.202 | 0.032 | 6.06E-142 | 1.55E-137 | Ep-C7 |
| DNAJB11 | 0.614 | 0.978 | 0.936 | 4.60E-141 | 1.18E-136 | Ep-C7 |
| PORCN | 0.622 | 0.441 | 0.158 | 1.00E-140 | 2.58E-136 | Ep-C7 |
| RBM3 | 0.598 | 0.993 | 0.926 | 1.01E-140 | 2.58E-136 | Ep-C7 |
| DST | 0.818 | 0.985 | 0.862 | 3.17E-139 | 8.12E-135 | Ep-C7 |
| EFNB2 | 0.732 | 0.817 | 0.512 | 1.82E-138 | 4.66E-134 | Ep-C7 |
| MAGEA3 | 0.655 | 0.73 | 0.377 | 5.63E-138 | 1.44E-133 | Ep-C7 |
| CD70 | 0.629 | 0.327 | 0.069 | 3.25E-137 | 8.33E-133 | Ep-C7 |
| UBE2Q2 | 0.682 | 0.655 | 0.41 | 4.61E-137 | 1.18E-132 | Ep-C7 |
| OS9 | 0.605 | 0.928 | 0.769 | 2.31E-136 | 5.91E-132 | Ep-C7 |
| PDLIM4 | 0.611 | 0.947 | 0.777 | 7.90E-136 | 2.03E-131 | Ep-C7 |
| ZBED2 | 0.57 | 0.371 | 0.102 | 1.41E-135 | 3.61E-131 | Ep-C7 |
| IRS1 | 0.593 | 0.488 | 0.186 | 1.78E-134 | 4.57E-130 | Ep-C7 |
| PLXNB2 | 0.648 | 0.831 | 0.577 | 9.70E-134 | 2.49E-129 | Ep-C7 |
| FNDC3B | 0.655 | 0.826 | 0.558 | 4.58E-133 | 1.17E-128 | Ep-C7 |
| REEP3 | 0.606 | 0.818 | 0.557 | 4.60E-133 | 1.18E-128 | Ep-C7 |
| NFE2L1 | 0.652 | 0.905 | 0.729 | 4.63E-133 | 1.19E-128 | Ep-C7 |
| PPP1R14C | 0.63 | 0.892 | 0.7 | 1.98E-132 | 5.08E-128 | Ep-C7 |
| NAA10 | 0.563 | 0.958 | 0.859 | 8.10E-132 | 2.08E-127 | Ep-C7 |
| PRR16 | 0.518 | 0.386 | 0.103 | 8.99E-132 | 2.30E-127 | Ep-C7 |
| MYO1C | 0.596 | 0.821 | 0.581 | 9.92E-132 | 2.54E-127 | Ep-C7 |
| TPM1 | 0.792 | 0.819 | 0.593 | 2.13E-131 | 5.46E-127 | Ep-C7 |
| TNFRSF11B | 1.357 | 0.172 | 0.029 | 3.20E-131 | 8.20E-127 | Ep-C7 |
| PLXNA1 | 0.607 | 0.634 | 0.35 | 1.32E-130 | 3.37E-126 | Ep-C7 |
| TMX2 | 0.616 | 0.91 | 0.761 | 1.38E-130 | 3.54E-126 | Ep-C7 |
| DCBLD1 | 0.595 | 0.582 | 0.257 | 1.48E-130 | 3.79E-126 | Ep-C7 |
| MYL12A | 0.532 | 1 | 0.996 | 2.22E-130 | 5.68E-126 | Ep-C7 |
| ATP5IF1 | 0.634 | 0.998 | 0.978 | 9.75E-130 | 2.50E-125 | Ep-C7 |
| RAB32 | 0.698 | 0.777 | 0.466 | 1.34E-129 | 3.43E-125 | Ep-C7 |
| SEC13 | 0.553 | 0.706 | 0.423 | 4.70E-129 | 1.20E-124 | Ep-C7 |
| NUDT21 | 0.656 | 0.927 | 0.796 | 6.27E-129 | 1.61E-124 | Ep-C7 |
| HLA-C | 0.608 | 0.999 | 0.992 | 1.05E-128 | 2.70E-124 | Ep-C7 |
| PXN | 0.548 | 0.614 | 0.296 | 1.24E-128 | 3.19E-124 | Ep-C7 |
| PSMB2 | 0.539 | 0.998 | 0.987 | 1.23E-127 | 3.16E-123 | Ep-C7 |
| THAP12 | 0.617 | 0.723 | 0.443 | 4.18E-127 | 1.07E-122 | Ep-C7 |
| IGF2BP2 | 0.644 | 0.894 | 0.672 | 8.56E-127 | 2.20E-122 | Ep-C7 |
| CD276 | 0.578 | 0.604 | 0.31 | 3.33E-126 | 8.55E-122 | Ep-C7 |
| ARPC4 | 0.51 | 0.952 | 0.848 | 5.52E-126 | 1.41E-121 | Ep-C7 |
| CDA | 0.804 | 0.452 | 0.167 | 7.01E-126 | 1.80E-121 | Ep-C7 |
| HDAC1 | 0.638 | 0.955 | 0.893 | 2.83E-125 | 7.27E-121 | Ep-C7 |
| CTSB | 0.661 | 0.998 | 0.96 | 3.00E-125 | 7.70E-121 | Ep-C7 |
| ARSJ | 0.556 | 0.557 | 0.23 | 9.19E-125 | 2.36E-120 | Ep-C7 |
| DKK1 | 1.35 | 0.513 | 0.213 | 7.33E-124 | 1.88E-119 | Ep-C7 |
| COL1A1 | 0.833 | 0.521 | 0.254 | 8.13E-124 | 2.08E-119 | Ep-C7 |
| WDR1 | 0.546 | 0.909 | 0.735 | 8.70E-124 | 2.23E-119 | Ep-C7 |
| LOXL4 | 0.631 | 0.359 | 0.099 | 1.37E-123 | 3.51E-119 | Ep-C7 |
| C7orf50 | 0.604 | 0.91 | 0.758 | 3.49E-123 | 8.94E-119 | Ep-C7 |
| MAGEA11 | 0.517 | 0.558 | 0.233 | 2.09E-122 | 5.35E-118 | Ep-C7 |
| LINC01807 | 0.565 | 0.525 | 0.21 | 3.62E-122 | 9.29E-118 | Ep-C7 |
| C1orf122 | 0.549 | 0.943 | 0.866 | 1.01E-121 | 2.60E-117 | Ep-C7 |
| CA2 | 0.678 | 0.719 | 0.375 | 7.74E-121 | 1.98E-116 | Ep-C7 |
| TPBG | 0.667 | 0.916 | 0.67 | 1.16E-120 | 2.97E-116 | Ep-C7 |
| PDXK | 0.559 | 0.95 | 0.824 | 2.12E-120 | 5.44E-116 | Ep-C7 |
| LSR | 0.553 | 0.962 | 0.816 | 3.82E-120 | 9.80E-116 | Ep-C7 |
| NT5DC2 | 0.591 | 0.725 | 0.477 | 5.00E-120 | 1.28E-115 | Ep-C7 |
| SLC2A1 | 0.838 | 0.944 | 0.891 | 5.24E-120 | 1.34E-115 | Ep-C7 |
| EIF3B | 0.524 | 0.978 | 0.902 | 1.17E-119 | 2.99E-115 | Ep-C7 |
| TRNAU1AP | 0.578 | 0.751 | 0.472 | 1.55E-119 | 3.97E-115 | Ep-C7 |
| KCNK1 | 0.652 | 0.876 | 0.624 | 2.42E-119 | 6.21E-115 | Ep-C7 |
| FEZ1 | 0.599 | 0.485 | 0.206 | 2.75E-119 | 7.04E-115 | Ep-C7 |
| PKP3 | 0.518 | 0.986 | 0.89 | 4.89E-119 | 1.25E-114 | Ep-C7 |
| B2M | 0.671 | 1 | 1 | 6.74E-119 | 1.73E-114 | Ep-C7 |
| XRCC6 | 0.532 | 0.989 | 0.962 | 2.04E-118 | 5.23E-114 | Ep-C7 |
| FHL3 | 0.51 | 0.536 | 0.238 | 4.06E-118 | 1.04E-113 | Ep-C7 |
| SLC2A4RG | 0.57 | 0.933 | 0.799 | 4.32E-118 | 1.11E-113 | Ep-C7 |
| SERPINB5 | 0.589 | 0.976 | 0.864 | 4.94E-118 | 1.27E-113 | Ep-C7 |
| AP3S1 | 0.547 | 0.896 | 0.764 | 1.18E-117 | 3.02E-113 | Ep-C7 |
| IL1RAP | 0.626 | 0.724 | 0.457 | 2.41E-117 | 6.19E-113 | Ep-C7 |
| LMF2 | 0.587 | 0.698 | 0.462 | 2.64E-117 | 6.76E-113 | Ep-C7 |
| PTK7 | 0.614 | 0.736 | 0.509 | 2.84E-117 | 7.27E-113 | Ep-C7 |
| CAPN2 | 0.599 | 0.932 | 0.792 | 3.74E-117 | 9.57E-113 | Ep-C7 |
| NCLN | 0.545 | 0.702 | 0.408 | 1.49E-116 | 3.81E-112 | Ep-C7 |
| INTS1 | 0.56 | 0.735 | 0.468 | 2.11E-116 | 5.42E-112 | Ep-C7 |
| ERO1A | 0.638 | 0.912 | 0.679 | 5.47E-116 | 1.40E-111 | Ep-C7 |
| MAD1L1 | 0.639 | 0.625 | 0.34 | 5.73E-116 | 1.47E-111 | Ep-C7 |
| AC004556.3 | 0.584 | 0.597 | 0.283 | 1.00E-115 | 2.57E-111 | Ep-C7 |
| MFSD12 | 0.537 | 0.688 | 0.378 | 3.18E-115 | 8.15E-111 | Ep-C7 |
| MACF1 | 0.639 | 0.849 | 0.666 | 1.00E-114 | 2.56E-110 | Ep-C7 |
| MESD | 0.522 | 0.912 | 0.774 | 8.72E-114 | 2.24E-109 | Ep-C7 |
| TTYH3 | 0.503 | 0.546 | 0.244 | 1.09E-113 | 2.78E-109 | Ep-C7 |
| KLF7 | 0.572 | 0.634 | 0.325 | 1.20E-113 | 3.06E-109 | Ep-C7 |
| MED15 | 0.544 | 0.716 | 0.448 | 1.23E-113 | 3.17E-109 | Ep-C7 |
| C12orf75 | 0.971 | 0.92 | 0.763 | 1.47E-113 | 3.78E-109 | Ep-C7 |
| PLOD2 | 0.813 | 0.762 | 0.504 | 1.90E-113 | 4.88E-109 | Ep-C7 |
| SLC25A1 | 0.571 | 0.892 | 0.702 | 2.00E-113 | 5.13E-109 | Ep-C7 |
| SPHK1 | 0.693 | 0.549 | 0.327 | 3.41E-113 | 8.73E-109 | Ep-C7 |
| THBS1 | 0.655 | 0.339 | 0.123 | 8.19E-113 | 2.10E-108 | Ep-C7 |
| ACOT9 | 0.546 | 0.619 | 0.34 | 1.55E-112 | 3.97E-108 | Ep-C7 |
| CNN2 | 0.531 | 0.92 | 0.765 | 4.32E-112 | 1.11E-107 | Ep-C7 |
| MCTS1 | 0.526 | 0.938 | 0.84 | 4.35E-112 | 1.12E-107 | Ep-C7 |
| AIM2 | 0.684 | 0.52 | 0.22 | 5.86E-112 | 1.50E-107 | Ep-C7 |
| AC245041.2 | 0.609 | 0.682 | 0.39 | 6.18E-112 | 1.58E-107 | Ep-C7 |
| CYTOR | 0.611 | 0.573 | 0.325 | 1.61E-111 | 4.12E-107 | Ep-C7 |
| BCAM | 0.647 | 0.762 | 0.464 | 1.23E-110 | 3.15E-106 | Ep-C7 |
| TMX1 | 0.535 | 0.799 | 0.612 | 1.63E-110 | 4.19E-106 | Ep-C7 |
| GNA12 | 0.502 | 0.589 | 0.319 | 3.42E-110 | 8.76E-106 | Ep-C7 |
| LAMP1 | 0.545 | 0.993 | 0.972 | 4.61E-110 | 1.18E-105 | Ep-C7 |
| GLIPR1 | 0.728 | 0.527 | 0.235 | 6.44E-110 | 1.65E-105 | Ep-C7 |
| SLC7A8 | 0.619 | 0.607 | 0.316 | 8.83E-110 | 2.26E-105 | Ep-C7 |
| FAM50A | 0.541 | 0.822 | 0.615 | 6.61E-109 | 1.70E-104 | Ep-C7 |
| UQCRC1 | 0.502 | 0.977 | 0.921 | 1.03E-108 | 2.64E-104 | Ep-C7 |
| KRT5 | 0.549 | 1 | 0.999 | 2.01E-108 | 5.16E-104 | Ep-C7 |
| IFITM3 | 0.583 | 1 | 0.98 | 7.02E-108 | 1.80E-103 | Ep-C7 |
| CFH | 0.612 | 0.509 | 0.216 | 2.36E-107 | 6.05E-103 | Ep-C7 |
| CCT5 | 0.516 | 0.997 | 0.984 | 5.74E-107 | 1.47E-102 | Ep-C7 |
| TUSC3 | 0.52 | 0.872 | 0.631 | 8.32E-107 | 2.13E-102 | Ep-C7 |
| SULF1 | 0.784 | 0.352 | 0.124 | 1.08E-106 | 2.78E-102 | Ep-C7 |
| SORL1 | 0.551 | 0.533 | 0.255 | 1.15E-106 | 2.94E-102 | Ep-C7 |
| DGCR6L | 0.524 | 0.896 | 0.768 | 1.53E-106 | 3.93E-102 | Ep-C7 |
| FN1 | 1.001 | 0.355 | 0.112 | 1.68E-106 | 4.29E-102 | Ep-C7 |
| EZR | 0.551 | 0.993 | 0.958 | 3.13E-105 | 8.02E-101 | Ep-C7 |
| FADD | 0.606 | 0.893 | 0.666 | 3.28E-105 | 8.41E-101 | Ep-C7 |
| MFAP2 | 0.527 | 0.56 | 0.268 | 3.39E-105 | 8.69E-101 | Ep-C7 |
| NPTN | 0.54 | 0.824 | 0.617 | 1.21E-104 | 3.11E-100 | Ep-C7 |
| EVA1B | 0.5 | 0.548 | 0.253 | 2.72E-104 | 6.98E-100 | Ep-C7 |
| SNX8 | 0.523 | 0.619 | 0.354 | 3.53E-103 | 9.04E-99 | Ep-C7 |
| NRIP1 | 0.599 | 0.796 | 0.528 | 6.90E-103 | 1.77E-98 | Ep-C7 |
| AGRN | 0.557 | 0.641 | 0.384 | 9.65E-103 | 2.47E-98 | Ep-C7 |
| DSE | 0.544 | 0.721 | 0.419 | 2.32E-102 | 5.96E-98 | Ep-C7 |
| ATP6V0B | 0.543 | 0.981 | 0.979 | 6.01E-102 | 1.54E-97 | Ep-C7 |
| AC103702.2 | 0.568 | 0.658 | 0.362 | 1.73E-101 | 4.43E-97 | Ep-C7 |
| ANKLE2 | 0.553 | 0.827 | 0.621 | 2.07E-101 | 5.31E-97 | Ep-C7 |
| STON2 | 0.53 | 0.562 | 0.304 | 3.68E-101 | 9.42E-97 | Ep-C7 |
| LGALS3BP | 0.559 | 0.964 | 0.962 | 2.15E-100 | 5.52E-96 | Ep-C7 |
| N4BP1 | 0.587 | 0.887 | 0.748 | 2.62E-99 | 6.72E-95 | Ep-C7 |
| ERAP2 | 0.505 | 0.525 | 0.246 | 4.46E-99 | 1.14E-94 | Ep-C7 |
| PDLIM2 | 0.568 | 0.706 | 0.478 | 1.14E-97 | 2.92E-93 | Ep-C7 |
| ATP6AP1 | 0.519 | 0.855 | 0.668 | 9.48E-97 | 2.43E-92 | Ep-C7 |
| COL4A5 | 0.504 | 0.5 | 0.223 | 1.22E-96 | 3.12E-92 | Ep-C7 |
| AP003555.1 | 0.507 | 0.332 | 0.1 | 2.98E-96 | 7.64E-92 | Ep-C7 |
| TAPBP | 0.547 | 0.93 | 0.845 | 3.89E-96 | 9.98E-92 | Ep-C7 |
| IGHG3 | 0.521 | 0.675 | 0.386 | 6.30E-96 | 1.62E-91 | Ep-C7 |
| IFI6 | 1.131 | 0.862 | 0.725 | 4.74E-94 | 1.21E-89 | Ep-C7 |
| BCAP29 | 0.505 | 0.835 | 0.629 | 8.39E-94 | 2.15E-89 | Ep-C7 |
| IRF6 | 0.501 | 0.926 | 0.754 | 2.09E-93 | 5.35E-89 | Ep-C7 |
| ADGRG1 | 0.547 | 0.812 | 0.573 | 3.82E-93 | 9.79E-89 | Ep-C7 |
| GOLIM4 | 0.539 | 0.853 | 0.639 | 3.76E-92 | 9.64E-88 | Ep-C7 |
| RECQL | 0.635 | 0.648 | 0.427 | 7.09E-92 | 1.82E-87 | Ep-C7 |
| SUN1 | 0.509 | 0.847 | 0.658 | 1.11E-90 | 2.85E-86 | Ep-C7 |
| SEC61A1 | 0.528 | 0.844 | 0.689 | 2.10E-90 | 5.38E-86 | Ep-C7 |
| PTGFRN | 0.528 | 0.736 | 0.535 | 2.41E-90 | 6.19E-86 | Ep-C7 |
| MYLK | 0.783 | 0.371 | 0.147 | 4.84E-90 | 1.24E-85 | Ep-C7 |
| MAGEA6 | 0.51 | 0.687 | 0.397 | 5.68E-90 | 1.46E-85 | Ep-C7 |
| INAVA | 0.505 | 0.638 | 0.373 | 5.78E-90 | 1.48E-85 | Ep-C7 |
| DSC3 | 0.561 | 0.97 | 0.884 | 2.02E-89 | 5.18E-85 | Ep-C7 |
| NUDT1 | 0.585 | 0.914 | 0.808 | 2.17E-89 | 5.56E-85 | Ep-C7 |
| C1GALT1 | 0.51 | 0.643 | 0.385 | 4.96E-89 | 1.27E-84 | Ep-C7 |
| PDE7A | 0.506 | 0.583 | 0.324 | 9.88E-89 | 2.53E-84 | Ep-C7 |
| GJB5 | 0.545 | 0.692 | 0.454 | 1.38E-88 | 3.53E-84 | Ep-C7 |
| SKA2 | 0.521 | 0.866 | 0.671 | 1.51E-88 | 3.87E-84 | Ep-C7 |
| SLC39A6 | 0.512 | 0.722 | 0.509 | 4.66E-88 | 1.19E-83 | Ep-C7 |
| AHNAK2 | 0.514 | 0.866 | 0.606 | 5.27E-88 | 1.35E-83 | Ep-C7 |
| CCN3 | 0.93 | 0.27 | 0.183 | 3.07E-87 | 7.88E-83 | Ep-C7 |
| RANBP1 | 0.542 | 0.999 | 0.983 | 4.72E-87 | 1.21E-82 | Ep-C7 |
| PHLDA3 | 0.548 | 0.932 | 0.791 | 6.29E-84 | 1.61E-79 | Ep-C7 |
| FRMD4B | 0.519 | 0.633 | 0.37 | 1.24E-83 | 3.19E-79 | Ep-C7 |
| EFNB1 | 0.558 | 0.808 | 0.609 | 1.26E-83 | 3.24E-79 | Ep-C7 |
| GGH | 0.572 | 0.792 | 0.597 | 1.99E-83 | 5.10E-79 | Ep-C7 |
| ELL2 | 0.532 | 0.66 | 0.408 | 7.95E-83 | 2.04E-78 | Ep-C7 |
| TIMP3 | 0.58 | 0.308 | 0.115 | 3.37E-82 | 8.65E-78 | Ep-C7 |
| MT1M | 0.516 | 0.423 | 0.183 | 2.19E-81 | 5.60E-77 | Ep-C7 |
| SUN2 | 0.536 | 0.633 | 0.391 | 7.15E-81 | 1.83E-76 | Ep-C7 |
| CTHRC1 | 0.57 | 0.587 | 0.314 | 1.90E-80 | 4.88E-76 | Ep-C7 |
| CLDN1 | 0.887 | 0.818 | 0.7 | 1.98E-80 | 5.07E-76 | Ep-C7 |
| IGFL2 | 0.726 | 0.206 | 0.051 | 5.85E-80 | 1.50E-75 | Ep-C7 |
| HACD2 | 0.525 | 0.684 | 0.506 | 8.59E-80 | 2.20E-75 | Ep-C7 |
| TK1 | 0.7 | 0.757 | 0.544 | 1.62E-79 | 4.16E-75 | Ep-C7 |
| CDCP1 | 0.527 | 0.692 | 0.45 | 3.99E-79 | 1.02E-74 | Ep-C7 |
| IDS | 0.505 | 0.752 | 0.534 | 1.54E-77 | 3.95E-73 | Ep-C7 |
| WNT7B | 0.501 | 0.677 | 0.436 | 2.47E-77 | 6.33E-73 | Ep-C7 |
| TM4SF19 | 0.538 | 0.388 | 0.161 | 3.11E-77 | 7.98E-73 | Ep-C7 |
| VSIR | 0.518 | 0.561 | 0.326 | 2.04E-76 | 5.22E-72 | Ep-C7 |
| MMP1 | 0.834 | 0.329 | 0.116 | 2.37E-76 | 6.08E-72 | Ep-C7 |
| HLA-E | 0.527 | 0.995 | 0.975 | 4.57E-76 | 1.17E-71 | Ep-C7 |
| FST | 0.845 | 0.713 | 0.556 | 7.30E-76 | 1.87E-71 | Ep-C7 |
| RASAL2 | 0.501 | 0.532 | 0.311 | 2.35E-75 | 6.04E-71 | Ep-C7 |
| IGFL2-AS1 | 0.605 | 0.552 | 0.288 | 7.94E-75 | 2.03E-70 | Ep-C7 |
| TUBB | 0.527 | 0.995 | 0.98 | 8.87E-71 | 2.27E-66 | Ep-C7 |
| DCN | 0.73 | 0.345 | 0.137 | 9.87E-71 | 2.53E-66 | Ep-C7 |
| SLC16A3 | 0.575 | 0.774 | 0.576 | 2.52E-70 | 6.45E-66 | Ep-C7 |
| CKB | 0.748 | 0.933 | 0.855 | 2.73E-70 | 7.00E-66 | Ep-C7 |
| HERPUD1 | 0.631 | 0.852 | 0.827 | 1.09E-69 | 2.80E-65 | Ep-C7 |
| FLNB | 0.508 | 0.774 | 0.584 | 3.13E-66 | 8.02E-62 | Ep-C7 |
| CPE | 0.501 | 0.546 | 0.302 | 8.10E-66 | 2.08E-61 | Ep-C7 |
| IGFBP7 | 0.577 | 0.618 | 0.369 | 3.64E-65 | 9.33E-61 | Ep-C7 |
| RBP1 | 0.674 | 0.886 | 0.744 | 1.57E-64 | 4.01E-60 | Ep-C7 |
| DCBLD2 | 0.52 | 0.546 | 0.363 | 4.31E-63 | 1.10E-58 | Ep-C7 |
| IFI44 | 0.505 | 0.629 | 0.403 | 3.73E-62 | 9.56E-58 | Ep-C7 |
| CITED4 | 0.686 | 0.708 | 0.562 | 4.68E-61 | 1.20E-56 | Ep-C7 |
| TUBA1B | 0.609 | 0.989 | 0.962 | 1.10E-60 | 2.81E-56 | Ep-C7 |
| TNFSF10 | 0.71 | 0.882 | 0.848 | 6.14E-60 | 1.57E-55 | Ep-C7 |
| ACKR3 | 0.57 | 0.713 | 0.498 | 7.65E-60 | 1.96E-55 | Ep-C7 |
| TM4SF1 | 0.61 | 0.982 | 0.944 | 4.09E-59 | 1.05E-54 | Ep-C7 |
| COL6A2 | 0.99 | 0.347 | 0.182 | 2.96E-58 | 7.58E-54 | Ep-C7 |
| KRT7 | 0.717 | 0.26 | 0.249 | 1.00E-57 | 2.56E-53 | Ep-C7 |
| BIK | 0.52 | 0.624 | 0.419 | 3.50E-57 | 8.97E-53 | Ep-C7 |
| MT1X | 0.597 | 0.959 | 0.967 | 2.03E-56 | 5.20E-52 | Ep-C7 |
| CDH2 | 0.507 | 0.182 | 0.063 | 1.47E-54 | 3.76E-50 | Ep-C7 |
| MMP10 | 1.162 | 0.166 | 0.043 | 8.17E-53 | 2.09E-48 | Ep-C7 |
| ODC1 | 0.604 | 0.928 | 0.823 | 2.95E-51 | 7.56E-47 | Ep-C7 |
| FGFBP1 | 0.882 | 0.808 | 0.711 | 3.34E-50 | 8.56E-46 | Ep-C7 |
| C1QTNF12 | 0.512 | 0.707 | 0.576 | 1.30E-46 | 3.33E-42 | Ep-C7 |
| FOXM1 | 0.502 | 0.436 | 0.251 | 7.01E-46 | 1.80E-41 | Ep-C7 |
| F3 | 0.578 | 0.451 | 0.264 | 1.57E-45 | 4.02E-41 | Ep-C7 |
| SELENOP | 0.575 | 0.829 | 0.758 | 9.75E-42 | 2.50E-37 | Ep-C7 |
| CCND1 | 0.888 | 0.967 | 0.924 | 1.88E-38 | 4.81E-34 | Ep-C7 |
| COL6A1 | 0.716 | 0.328 | 0.206 | 6.61E-37 | 1.69E-32 | Ep-C7 |
| SHKBP1 | 0.536 | 0.633 | 0.489 | 1.84E-35 | 4.71E-31 | Ep-C7 |
| AMTN | 0.888 | 0.122 | 0.051 | 2.19E-20 | 5.61E-16 | Ep-C7 |
| FGF19 | 0.896 | 0.215 | 0.137 | 3.24E-17 | 8.32E-13 | Ep-C7 |
| SPRR3 | 6.993 | 0.764 | 0.251 | 0 | 0 | Ep-C8 |
| SPRR2E | 4.924 | 0.753 | 0.199 | 0 | 0 | Ep-C8 |
| SPRR2A | 4.827 | 0.908 | 0.389 | 0 | 0 | Ep-C8 |
| SPRR2D | 4.551 | 0.827 | 0.257 | 0 | 0 | Ep-C8 |
| SPRR1B | 4.463 | 0.914 | 0.332 | 0 | 0 | Ep-C8 |
| PI3 | 4.249 | 0.694 | 0.256 | 0 | 0 | Ep-C8 |
| RHCG | 3.944 | 0.9 | 0.246 | 0 | 0 | Ep-C8 |
| KRT13 | 3.757 | 0.957 | 0.704 | 0 | 0 | Ep-C8 |
| CNFN | 3.734 | 0.668 | 0.407 | 0 | 0 | Ep-C8 |
| S100A7 | 3.681 | 0.905 | 0.557 | 0 | 0 | Ep-C8 |
| SLPI | 3.408 | 0.987 | 0.659 | 0 | 0 | Ep-C8 |
| LCN2 | 3.349 | 0.968 | 0.574 | 0 | 0 | Ep-C8 |
| CSTB | 3.295 | 1 | 1 | 0 | 0 | Ep-C8 |
| S100A8 | 3.137 | 0.998 | 0.857 | 0 | 0 | Ep-C8 |
| CSTA | 3.103 | 1 | 0.922 | 0 | 0 | Ep-C8 |
| KRT6C | 3.065 | 0.611 | 0.146 | 0 | 0 | Ep-C8 |
| CD24 | 3.042 | 0.965 | 0.569 | 0 | 0 | Ep-C8 |
| SERPINB3 | 3.007 | 0.888 | 0.595 | 0 | 0 | Ep-C8 |
| CLIC3 | 2.927 | 0.63 | 0.114 | 0 | 0 | Ep-C8 |
| SERPINB4 | 2.914 | 0.75 | 0.345 | 0 | 0 | Ep-C8 |
| AQP3 | 2.736 | 0.841 | 0.507 | 0 | 0 | Ep-C8 |
| IGFBP3 | 2.701 | 0.736 | 0.332 | 0 | 0 | Ep-C8 |
| SLURP2 | 2.697 | 0.382 | 0.041 | 0 | 0 | Ep-C8 |
| IVL | 2.696 | 0.565 | 0.035 | 0 | 0 | Ep-C8 |
| IL1RN | 2.684 | 0.849 | 0.206 | 0 | 0 | Ep-C8 |
| S100A9 | 2.646 | 0.999 | 0.918 | 0 | 0 | Ep-C8 |
| IL36G | 2.64 | 0.601 | 0.173 | 0 | 0 | Ep-C8 |
| SBSN | 2.411 | 0.466 | 0.067 | 0 | 0 | Ep-C8 |
| S100A12 | 2.405 | 0.627 | 0.123 | 0 | 0 | Ep-C8 |
| KRT16 | 2.312 | 0.967 | 0.843 | 0 | 0 | Ep-C8 |
| PRSS3 | 2.291 | 0.449 | 0.048 | 0 | 0 | Ep-C8 |
| MALL | 2.216 | 0.894 | 0.307 | 0 | 0 | Ep-C8 |
| LYPD3 | 2.172 | 0.968 | 0.716 | 0 | 0 | Ep-C8 |
| GJB2 | 2.171 | 0.949 | 0.687 | 0 | 0 | Ep-C8 |
| KLK12 | 2.12 | 0.419 | 0.036 | 0 | 0 | Ep-C8 |
| GLTP | 2.034 | 0.994 | 0.875 | 0 | 0 | Ep-C8 |
| TGM1 | 2.027 | 0.602 | 0.085 | 0 | 0 | Ep-C8 |
| SULT2B1 | 2.008 | 0.628 | 0.083 | 0 | 0 | Ep-C8 |
| RAB11FIP1 | 1.991 | 0.843 | 0.486 | 0 | 0 | Ep-C8 |
| GJB6 | 1.97 | 0.861 | 0.499 | 0 | 0 | Ep-C8 |
| SDC1 | 1.953 | 0.994 | 0.913 | 0 | 0 | Ep-C8 |
| MXD1 | 1.952 | 0.857 | 0.5 | 0 | 0 | Ep-C8 |
| CYSRT1 | 1.857 | 0.515 | 0.039 | 0 | 0 | Ep-C8 |
| SPRR1A | 1.856 | 0.558 | 0.03 | 0 | 0 | Ep-C8 |
| CLDN4 | 1.828 | 0.979 | 0.684 | 0 | 0 | Ep-C8 |
| SDCBP2 | 1.827 | 0.88 | 0.28 | 0 | 0 | Ep-C8 |
| C10orf99 | 1.813 | 0.691 | 0.191 | 0 | 0 | Ep-C8 |
| ANXA1 | 1.813 | 0.994 | 0.986 | 0 | 0 | Ep-C8 |
| KLK11 | 1.811 | 0.94 | 0.583 | 0 | 0 | Ep-C8 |
| SERPINB13 | 1.8 | 0.839 | 0.401 | 0 | 0 | Ep-C8 |
| FAM83A | 1.796 | 0.799 | 0.389 | 0 | 0 | Ep-C8 |
| DSC2 | 1.783 | 0.851 | 0.556 | 0 | 0 | Ep-C8 |
| PRSS22 | 1.759 | 0.788 | 0.338 | 0 | 0 | Ep-C8 |
| SERPINB1 | 1.754 | 0.973 | 0.8 | 0 | 0 | Ep-C8 |
| NECTIN4 | 1.752 | 0.948 | 0.483 | 0 | 0 | Ep-C8 |
| AC105460.1 | 1.672 | 0.757 | 0.295 | 0 | 0 | Ep-C8 |
| KLK13 | 1.656 | 0.521 | 0.119 | 0 | 0 | Ep-C8 |
| NCCRP1 | 1.651 | 0.353 | 0.017 | 0 | 0 | Ep-C8 |
| CLDN7 | 1.634 | 0.973 | 0.671 | 0 | 0 | Ep-C8 |
| RAB10 | 1.629 | 0.991 | 0.942 | 0 | 0 | Ep-C8 |
| GRHL1 | 1.597 | 0.785 | 0.272 | 0 | 0 | Ep-C8 |
| KLK8 | 1.579 | 0.898 | 0.608 | 0 | 0 | Ep-C8 |
| METRNL | 1.574 | 0.864 | 0.403 | 0 | 0 | Ep-C8 |
| SAMD9 | 1.545 | 0.955 | 0.618 | 0 | 0 | Ep-C8 |
| ELF3 | 1.545 | 0.996 | 0.882 | 0 | 0 | Ep-C8 |
| TMEM40 | 1.532 | 0.884 | 0.365 | 0 | 0 | Ep-C8 |
| CST3 | 1.531 | 0.999 | 0.969 | 0 | 0 | Ep-C8 |
| TMPRSS11D | 1.513 | 0.885 | 0.458 | 0 | 0 | Ep-C8 |
| TACSTD2 | 1.505 | 1 | 0.934 | 0 | 0 | Ep-C8 |
| SMIM22 | 1.49 | 0.848 | 0.397 | 0 | 0 | Ep-C8 |
| C4orf3 | 1.483 | 0.997 | 0.955 | 0 | 0 | Ep-C8 |
| SAT1 | 1.467 | 0.997 | 0.988 | 0 | 0 | Ep-C8 |
| UGCG | 1.466 | 0.865 | 0.522 | 0 | 0 | Ep-C8 |
| RAB25 | 1.458 | 0.983 | 0.832 | 0 | 0 | Ep-C8 |
| PRSS27 | 1.454 | 0.349 | 0.023 | 0 | 0 | Ep-C8 |
| SMIM5 | 1.443 | 0.497 | 0.027 | 0 | 0 | Ep-C8 |
| TMEM79 | 1.434 | 0.781 | 0.274 | 0 | 0 | Ep-C8 |
| PERP | 1.422 | 1 | 1 | 0 | 0 | Ep-C8 |
| TMEM45B | 1.385 | 0.63 | 0.069 | 0 | 0 | Ep-C8 |
| MAL2 | 1.372 | 0.993 | 0.902 | 0 | 0 | Ep-C8 |
| EPS8L1 | 1.329 | 0.513 | 0.115 | 0 | 0 | Ep-C8 |
| FUT3 | 1.311 | 0.528 | 0.049 | 0 | 0 | Ep-C8 |
| PPL | 1.294 | 0.729 | 0.358 | 0 | 0 | Ep-C8 |
| NDRG2 | 1.289 | 0.556 | 0.233 | 0 | 0 | Ep-C8 |
| MPZL2 | 1.283 | 0.981 | 0.787 | 0 | 0 | Ep-C8 |
| NIBAN2 | 1.251 | 0.895 | 0.613 | 0 | 0 | Ep-C8 |
| CD68 | 1.243 | 0.837 | 0.399 | 0 | 0 | Ep-C8 |
| ABLIM1 | 1.21 | 0.829 | 0.346 | 0 | 0 | Ep-C8 |
| MBOAT2 | 1.19 | 0.75 | 0.348 | 0 | 0 | Ep-C8 |
| ARHGAP27 | 1.177 | 0.64 | 0.257 | 0 | 0 | Ep-C8 |
| GRHL3 | 1.171 | 0.621 | 0.081 | 0 | 0 | Ep-C8 |
| CEACAM1 | 1.162 | 0.444 | 0.042 | 0 | 0 | Ep-C8 |
| CARHSP1 | 1.091 | 0.96 | 0.8 | 0 | 0 | Ep-C8 |
| GIPC1 | 1.069 | 0.988 | 0.868 | 0 | 0 | Ep-C8 |
| S100A11 | 1.062 | 1 | 1 | 0 | 0 | Ep-C8 |
| LIPH | 1.034 | 0.388 | 0.034 | 0 | 0 | Ep-C8 |
| RAB11A | 1.014 | 0.972 | 0.889 | 0 | 0 | Ep-C8 |
| EPHX3 | 0.994 | 0.63 | 0.148 | 0 | 0 | Ep-C8 |
| ASPG | 0.99 | 0.462 | 0.06 | 0 | 0 | Ep-C8 |
| SCEL | 0.99 | 0.377 | 0.019 | 0 | 0 | Ep-C8 |
| TTC9 | 0.986 | 0.565 | 0.121 | 0 | 0 | Ep-C8 |
| ALDH3B2 | 0.974 | 0.608 | 0.144 | 0 | 0 | Ep-C8 |
| ATP11B | 1.004 | 0.786 | 0.522 | 6.91691904177745e-323 | 1.77287551959798e-318 | Ep-C8 |
| EPHA2 | 1.263 | 0.866 | 0.502 | 2.45828982567966e-317 | 6.30084265219955e-313 | Ep-C8 |
| KRT23 | 2.171 | 0.551 | 0.144 | 8.34437850074566e-314 | 2.13874765352612e-309 | Ep-C8 |
| PRDM1 | 1.268 | 0.609 | 0.192 | 1.00390691674509e-313 | 2.57311381830933e-309 | Ep-C8 |
| FAM3D | 1.64 | 0.332 | 0.026 | 1.46456850633932e-312 | 3.75E-308 | Ep-C8 |
| TMPRSS2 | 0.987 | 0.448 | 0.068 | 3.84903949964455e-311 | 9.87E-307 | Ep-C8 |
| SPINT1 | 1.039 | 0.961 | 0.802 | 6.45756142401055e-311 | 1.66E-306 | Ep-C8 |
| MUC4 | 1.744 | 0.628 | 0.209 | 8.5145260370723e-311 | 2.18E-306 | Ep-C8 |
| JUP | 0.954 | 0.999 | 0.992 | 8.38676187088742e-309 | 2.15E-304 | Ep-C8 |
| KRT80 | 1.071 | 0.353 | 0.033 | 4.73E-307 | 1.21E-302 | Ep-C8 |
| MUC20 | 1.74 | 0.51 | 0.129 | 5.03E-307 | 1.29E-302 | Ep-C8 |
| PIM1 | 1.33 | 0.747 | 0.374 | 9.02E-305 | 2.31E-300 | Ep-C8 |
| BICDL2 | 0.936 | 0.623 | 0.217 | 3.60E-300 | 9.22E-296 | Ep-C8 |
| ECM1 | 2.129 | 0.569 | 0.234 | 1.87E-298 | 4.79E-294 | Ep-C8 |
| PLEKHM1 | 0.945 | 0.557 | 0.187 | 1.34E-294 | 3.43E-290 | Ep-C8 |
| LINC01133 | 1.359 | 0.889 | 0.476 | 8.64E-294 | 2.21E-289 | Ep-C8 |
| KRT6B | 1.887 | 0.955 | 0.795 | 6.43E-293 | 1.65E-288 | Ep-C8 |
| SDR9C7 | 0.618 | 0.252 | 0.005 | 7.01E-289 | 1.80E-284 | Ep-C8 |
| TMPRSS11A | 1.151 | 0.672 | 0.19 | 6.55E-286 | 1.68E-281 | Ep-C8 |
| HK2 | 1.554 | 0.646 | 0.27 | 9.73E-285 | 2.50E-280 | Ep-C8 |
| HEBP2 | 0.925 | 0.991 | 0.968 | 1.86E-284 | 4.78E-280 | Ep-C8 |
| DBI | 1.548 | 0.998 | 0.998 | 1.03E-282 | 2.65E-278 | Ep-C8 |
| FLG | 1.781 | 0.412 | 0.056 | 2.11E-280 | 5.42E-276 | Ep-C8 |
| S100P | 2.227 | 0.381 | 0.058 | 5.90E-279 | 1.51E-274 | Ep-C8 |
| PRSS8 | 1.087 | 0.726 | 0.327 | 3.31E-278 | 8.49E-274 | Ep-C8 |
| CYP3A5 | 0.837 | 0.455 | 0.078 | 9.23E-277 | 2.36E-272 | Ep-C8 |
| B4GALT4 | 1.308 | 0.804 | 0.519 | 4.50E-276 | 1.15E-271 | Ep-C8 |
| OAS1 | 1.46 | 0.684 | 0.368 | 6.25E-276 | 1.60E-271 | Ep-C8 |
| A2ML1 | 0.96 | 0.311 | 0.026 | 6.78E-273 | 1.74E-268 | Ep-C8 |
| TGFA | 1.004 | 0.587 | 0.207 | 1.10E-272 | 2.83E-268 | Ep-C8 |
| ANKRD22 | 0.852 | 0.47 | 0.101 | 1.07E-266 | 2.74E-262 | Ep-C8 |
| KRT6A | 1.356 | 0.998 | 1 | 4.23E-263 | 1.08E-258 | Ep-C8 |
| S100A7A | 2.006 | 0.389 | 0.055 | 6.84E-263 | 1.75E-258 | Ep-C8 |
| RAC1 | 0.651 | 1 | 1 | 1.66E-262 | 4.25E-258 | Ep-C8 |
| RAB5IF | 0.802 | 0.997 | 0.995 | 6.16E-261 | 1.58E-256 | Ep-C8 |
| CHMP5 | 1.274 | 0.955 | 0.893 | 7.49E-261 | 1.92E-256 | Ep-C8 |
| PCDH1 | 0.593 | 0.331 | 0.03 | 8.44E-260 | 2.16E-255 | Ep-C8 |
| SERPINB11 | 0.678 | 0.326 | 0.026 | 5.75E-259 | 1.47E-254 | Ep-C8 |
| C15orf48 | 2.527 | 0.389 | 0.105 | 3.65E-258 | 9.35E-254 | Ep-C8 |
| ARPC5 | 1.004 | 0.998 | 0.978 | 5.46E-258 | 1.40E-253 | Ep-C8 |
| SH3BGRL3 | 0.852 | 1 | 0.995 | 5.98E-258 | 1.53E-253 | Ep-C8 |
| CERS3 | 0.853 | 0.677 | 0.306 | 1.78E-256 | 4.55E-252 | Ep-C8 |
| GCNT3 | 0.933 | 0.343 | 0.037 | 9.35E-256 | 2.40E-251 | Ep-C8 |
| PPP1CB | 1.073 | 0.995 | 0.963 | 1.27E-254 | 3.26E-250 | Ep-C8 |
| RNF141 | 0.838 | 0.835 | 0.574 | 2.66E-253 | 6.83E-249 | Ep-C8 |
| BNIPL | 0.733 | 0.443 | 0.083 | 2.80E-250 | 7.19E-246 | Ep-C8 |
| UBAP1 | 1.118 | 0.737 | 0.398 | 2.56E-249 | 6.56E-245 | Ep-C8 |
| ZNF185 | 0.889 | 0.651 | 0.273 | 1.17E-247 | 2.99E-243 | Ep-C8 |
| DCAF12 | 1.144 | 0.729 | 0.393 | 2.17E-247 | 5.57E-243 | Ep-C8 |
| EVPL | 0.869 | 0.605 | 0.272 | 4.05E-246 | 1.04E-241 | Ep-C8 |
| DEGS2 | 1.035 | 0.523 | 0.134 | 2.09E-244 | 5.35E-240 | Ep-C8 |
| NCF2 | 0.807 | 0.436 | 0.074 | 7.85E-242 | 2.01E-237 | Ep-C8 |
| COX7A1 | 1.168 | 0.452 | 0.102 | 7.26E-241 | 1.86E-236 | Ep-C8 |
| ERBB3 | 0.881 | 0.711 | 0.363 | 5.01E-240 | 1.29E-235 | Ep-C8 |
| ID1 | 1.49 | 0.96 | 0.824 | 2.91E-239 | 7.46E-235 | Ep-C8 |
| DSG3 | 1.092 | 0.98 | 0.81 | 1.75E-238 | 4.48E-234 | Ep-C8 |
| EHF | 1.008 | 0.939 | 0.666 | 8.71E-236 | 2.23E-231 | Ep-C8 |
| C6orf132 | 0.939 | 0.797 | 0.435 | 9.60E-236 | 2.46E-231 | Ep-C8 |
| CXCL17 | 1.495 | 0.551 | 0.159 | 1.73E-234 | 4.44E-230 | Ep-C8 |
| CLCA4 | 1.181 | 0.396 | 0.068 | 7.21E-234 | 1.85E-229 | Ep-C8 |
| CYB5R1 | 1 | 0.756 | 0.448 | 3.04E-231 | 7.79E-227 | Ep-C8 |
| SPRR2F | 2.517 | 0.299 | 0.043 | 3.08E-229 | 7.89E-225 | Ep-C8 |
| PTK6 | 0.782 | 0.612 | 0.231 | 1.68E-227 | 4.30E-223 | Ep-C8 |
| LY6G6C | 0.749 | 0.358 | 0.052 | 4.48E-227 | 1.15E-222 | Ep-C8 |
| ZDHHC13 | 0.763 | 0.598 | 0.276 | 2.15E-226 | 5.50E-222 | Ep-C8 |
| SLURP1 | 1.71 | 0.224 | 0.01 | 6.23E-225 | 1.60E-220 | Ep-C8 |
| SMAGP | 0.88 | 0.681 | 0.38 | 2.42E-222 | 6.20E-218 | Ep-C8 |
| CRB3 | 0.875 | 0.768 | 0.441 | 8.16E-222 | 2.09E-217 | Ep-C8 |
| SPTSSB | 1.145 | 0.388 | 0.088 | 3.00E-221 | 7.69E-217 | Ep-C8 |
| ARPC3 | 0.625 | 1 | 0.999 | 4.46E-221 | 1.14E-216 | Ep-C8 |
| B3GNT8 | 0.729 | 0.514 | 0.149 | 2.65E-218 | 6.80E-214 | Ep-C8 |
| B3GNT5 | 0.913 | 0.895 | 0.656 | 1.68E-217 | 4.30E-213 | Ep-C8 |
| UBE2R2 | 1.056 | 0.864 | 0.777 | 3.74E-216 | 9.60E-212 | Ep-C8 |
| F3 | 1.656 | 0.578 | 0.256 | 7.24E-216 | 1.86E-211 | Ep-C8 |
| TMPRSS11E | 1.529 | 0.6 | 0.202 | 4.78E-214 | 1.23E-209 | Ep-C8 |
| TRIM16 | 0.903 | 0.811 | 0.529 | 7.84E-212 | 2.01E-207 | Ep-C8 |
| AC008397.1 | 0.901 | 0.392 | 0.077 | 8.59E-212 | 2.20E-207 | Ep-C8 |
| TTC22 | 0.711 | 0.521 | 0.19 | 1.18E-210 | 3.02E-206 | Ep-C8 |
| GNA15 | 0.929 | 0.836 | 0.564 | 3.83E-210 | 9.81E-206 | Ep-C8 |
| FAM3B | 1.114 | 0.738 | 0.341 | 3.90E-209 | 1.00E-204 | Ep-C8 |
| VAMP8 | 0.748 | 0.996 | 0.975 | 7.98E-208 | 2.05E-203 | Ep-C8 |
| MAP3K8 | 1.008 | 0.74 | 0.374 | 8.67E-208 | 2.22E-203 | Ep-C8 |
| TPM4 | 0.847 | 0.986 | 0.976 | 1.37E-207 | 3.51E-203 | Ep-C8 |
| GRN | 0.854 | 0.997 | 0.959 | 1.96E-207 | 5.02E-203 | Ep-C8 |
| CTSD | 0.937 | 0.997 | 0.977 | 2.27E-207 | 5.81E-203 | Ep-C8 |
| SPINT2 | 0.721 | 1 | 0.999 | 4.43E-206 | 1.13E-201 | Ep-C8 |
| BZW1 | 0.816 | 0.953 | 0.873 | 3.35E-203 | 8.60E-199 | Ep-C8 |
| S100A16 | 0.838 | 0.999 | 0.996 | 5.59E-203 | 1.43E-198 | Ep-C8 |
| HILPDA | 1.621 | 0.671 | 0.418 | 1.29E-198 | 3.30E-194 | Ep-C8 |
| MRPS30-DT | 0.875 | 0.444 | 0.103 | 4.13E-198 | 1.06E-193 | Ep-C8 |
| EIF6 | 0.669 | 0.995 | 0.992 | 1.21E-196 | 3.11E-192 | Ep-C8 |
| CEACAM5 | 1.674 | 0.239 | 0.031 | 3.31E-194 | 8.47E-190 | Ep-C8 |
| MUC1 | 1.131 | 0.626 | 0.277 | 3.57E-194 | 9.16E-190 | Ep-C8 |
| TMEM265 | 0.912 | 0.87 | 0.533 | 7.31E-193 | 1.87E-188 | Ep-C8 |
| PDZK1IP1 | 1.287 | 0.792 | 0.449 | 1.05E-191 | 2.70E-187 | Ep-C8 |
| DNAJC5 | 0.727 | 0.744 | 0.457 | 6.57E-191 | 1.68E-186 | Ep-C8 |
| DHRS9 | 0.97 | 0.201 | 0.012 | 8.62E-190 | 2.21E-185 | Ep-C8 |
| ATP6V1G1 | 0.648 | 0.999 | 0.998 | 1.48E-189 | 3.78E-185 | Ep-C8 |
| TMEM41A | 0.818 | 0.843 | 0.671 | 7.32E-189 | 1.88E-184 | Ep-C8 |
| CITED4 | 1.087 | 0.774 | 0.558 | 1.11E-186 | 2.85E-182 | Ep-C8 |
| DUOX2 | 0.848 | 0.278 | 0.046 | 2.01E-185 | 5.15E-181 | Ep-C8 |
| C19orf33 | 0.82 | 0.998 | 0.885 | 1.19E-183 | 3.06E-179 | Ep-C8 |
| MYH14 | 0.705 | 0.617 | 0.271 | 1.00E-182 | 2.57E-178 | Ep-C8 |
| CLTA | 1.074 | 0.989 | 0.995 | 1.07E-182 | 2.75E-178 | Ep-C8 |
| KRT24 | 0.944 | 0.172 | 0.005 | 1.79E-182 | 4.58E-178 | Ep-C8 |
| CRYBG2 | 0.614 | 0.479 | 0.138 | 2.22E-182 | 5.69E-178 | Ep-C8 |
| FABP5 | 1.268 | 0.997 | 0.997 | 4.02E-182 | 1.03E-177 | Ep-C8 |
| IL36RN | 0.724 | 0.189 | 0.009 | 8.85E-181 | 2.27E-176 | Ep-C8 |
| PHLDA2 | 0.894 | 0.985 | 0.898 | 9.51E-179 | 2.44E-174 | Ep-C8 |
| FAM214B | 0.717 | 0.469 | 0.161 | 1.91E-178 | 4.90E-174 | Ep-C8 |
| NDRG1 | 1.143 | 0.98 | 0.868 | 8.66E-178 | 2.22E-173 | Ep-C8 |
| ARPC5L | 0.701 | 0.988 | 0.934 | 8.78E-178 | 2.25E-173 | Ep-C8 |
| KLK10 | 1.24 | 0.766 | 0.441 | 1.86E-177 | 4.76E-173 | Ep-C8 |
| RHOV | 0.839 | 0.955 | 0.742 | 2.85E-177 | 7.29E-173 | Ep-C8 |
| QSOX1 | 0.824 | 0.564 | 0.272 | 3.20E-176 | 8.20E-172 | Ep-C8 |
| PSORS1C2 | 0.503 | 0.167 | 0.005 | 6.29E-176 | 1.61E-171 | Ep-C8 |
| DDAH2 | 0.853 | 0.72 | 0.383 | 2.24E-175 | 5.73E-171 | Ep-C8 |
| IKZF2 | 0.527 | 0.364 | 0.079 | 4.04E-174 | 1.04E-169 | Ep-C8 |
| PROM2 | 0.884 | 0.709 | 0.396 | 5.38E-174 | 1.38E-169 | Ep-C8 |
| SDC4 | 0.837 | 0.965 | 0.833 | 6.49E-174 | 1.66E-169 | Ep-C8 |
| PPARD | 0.687 | 0.491 | 0.205 | 1.90E-173 | 4.88E-169 | Ep-C8 |
| CDH1 | 0.77 | 0.87 | 0.642 | 1.60E-172 | 4.11E-168 | Ep-C8 |
| CIB1 | 0.708 | 0.994 | 0.984 | 4.23E-172 | 1.08E-167 | Ep-C8 |
| TRIP10 | 0.702 | 0.627 | 0.326 | 6.12E-172 | 1.57E-167 | Ep-C8 |
| SH3GLB1 | 0.68 | 0.942 | 0.834 | 1.72E-170 | 4.42E-166 | Ep-C8 |
| SLCO4A1 | 0.563 | 0.332 | 0.064 | 1.75E-170 | 4.48E-166 | Ep-C8 |
| GLUL | 1.071 | 0.985 | 0.969 | 1.96E-168 | 5.02E-164 | Ep-C8 |
| NBEAL2 | 0.617 | 0.49 | 0.174 | 1.19E-167 | 3.04E-163 | Ep-C8 |
| BCL2L1 | 0.696 | 0.604 | 0.329 | 9.06E-167 | 2.32E-162 | Ep-C8 |
| ST14 | 0.781 | 0.732 | 0.456 | 2.72E-165 | 6.98E-161 | Ep-C8 |
| NPEPPS | 0.771 | 0.759 | 0.551 | 1.27E-161 | 3.25E-157 | Ep-C8 |
| CRCT1 | 2.976 | 0.215 | 0.039 | 4.38E-161 | 1.12E-156 | Ep-C8 |
| MAP1LC3A | 0.931 | 0.681 | 0.378 | 1.06E-160 | 2.71E-156 | Ep-C8 |
| SH3YL1 | 0.76 | 0.774 | 0.441 | 1.74E-160 | 4.45E-156 | Ep-C8 |
| AL121761.1 | 1.032 | 0.322 | 0.071 | 4.60E-159 | 1.18E-154 | Ep-C8 |
| ADGRF1 | 0.948 | 0.222 | 0.022 | 4.31E-158 | 1.10E-153 | Ep-C8 |
| MAGEA4 | 0.96 | 0.648 | 0.26 | 2.92E-157 | 7.49E-153 | Ep-C8 |
| GPX3 | 0.607 | 0.307 | 0.062 | 1.95E-156 | 5.01E-152 | Ep-C8 |
| AHNAK2 | 0.983 | 0.879 | 0.606 | 4.37E-155 | 1.12E-150 | Ep-C8 |
| ALOX15B | 0.595 | 0.318 | 0.066 | 4.62E-154 | 1.18E-149 | Ep-C8 |
| ZDHHC21 | 0.554 | 0.453 | 0.166 | 1.65E-153 | 4.23E-149 | Ep-C8 |
| DUOXA2 | 0.864 | 0.178 | 0.012 | 5.71E-153 | 1.46E-148 | Ep-C8 |
| TMPRSS4 | 0.834 | 0.907 | 0.68 | 7.39E-153 | 1.90E-148 | Ep-C8 |
| KLK9 | 0.637 | 0.321 | 0.075 | 7.53E-153 | 1.93E-148 | Ep-C8 |
| KRT10 | 1.148 | 0.998 | 0.997 | 2.55E-152 | 6.52E-148 | Ep-C8 |
| CEACAM6 | 1.342 | 0.262 | 0.057 | 4.50E-152 | 1.15E-147 | Ep-C8 |
| ADAM15 | 0.654 | 0.793 | 0.529 | 5.64E-148 | 1.45E-143 | Ep-C8 |
| TMPRSS13 | 0.54 | 0.454 | 0.156 | 5.85E-148 | 1.50E-143 | Ep-C8 |
| HMOX1 | 1.109 | 0.432 | 0.131 | 2.30E-147 | 5.88E-143 | Ep-C8 |
| MAP3K13 | 0.698 | 0.986 | 0.965 | 2.16E-146 | 5.54E-142 | Ep-C8 |
| SLC12A6 | 0.58 | 0.504 | 0.191 | 2.39E-146 | 6.13E-142 | Ep-C8 |
| SLAMF9 | 0.55 | 0.223 | 0.024 | 3.16E-146 | 8.10E-142 | Ep-C8 |
| RAET1G | 0.536 | 0.395 | 0.1 | 8.52E-146 | 2.18E-141 | Ep-C8 |
| SFN | 0.907 | 0.994 | 0.999 | 2.33E-145 | 5.97E-141 | Ep-C8 |
| CDKN1A | 0.87 | 0.895 | 0.692 | 6.89E-145 | 1.77E-140 | Ep-C8 |
| GGT6 | 0.51 | 0.351 | 0.089 | 1.00E-144 | 2.56E-140 | Ep-C8 |
| SLC20A2 | 0.807 | 0.75 | 0.523 | 4.57E-144 | 1.17E-139 | Ep-C8 |
| CYP4B1 | 0.814 | 0.16 | 0.012 | 1.71E-143 | 4.38E-139 | Ep-C8 |
| MPZL3 | 0.519 | 0.392 | 0.13 | 2.43E-143 | 6.24E-139 | Ep-C8 |
| RBM47 | 0.671 | 0.721 | 0.441 | 1.49E-142 | 3.82E-138 | Ep-C8 |
| CXXC5 | 0.591 | 0.449 | 0.185 | 1.96E-142 | 5.03E-138 | Ep-C8 |
| GTF2A2 | 0.664 | 0.96 | 0.922 | 4.88E-142 | 1.25E-137 | Ep-C8 |
| CPEB4 | 0.598 | 0.491 | 0.214 | 1.78E-141 | 4.55E-137 | Ep-C8 |
| RAB7A | 0.572 | 0.998 | 0.973 | 2.47E-141 | 6.34E-137 | Ep-C8 |
| B4GALT5 | 0.761 | 0.604 | 0.407 | 6.50E-141 | 1.67E-136 | Ep-C8 |
| RALB | 0.648 | 0.673 | 0.406 | 7.56E-141 | 1.94E-136 | Ep-C8 |
| EPHB3 | 0.789 | 0.578 | 0.315 | 8.49E-141 | 2.18E-136 | Ep-C8 |
| ELF3-AS1 | 0.53 | 0.405 | 0.119 | 1.78E-140 | 4.55E-136 | Ep-C8 |
| CLDND1 | 0.783 | 0.909 | 0.796 | 3.41E-140 | 8.75E-136 | Ep-C8 |
| KRTDAP | 2.633 | 0.258 | 0.053 | 4.13E-140 | 1.06E-135 | Ep-C8 |
| ZBTB7B | 0.603 | 0.557 | 0.265 | 6.14E-140 | 1.57E-135 | Ep-C8 |
| ERO1A | 1.105 | 0.852 | 0.683 | 4.54E-139 | 1.16E-134 | Ep-C8 |
| CAMK1G | 0.503 | 0.49 | 0.192 | 6.86E-139 | 1.76E-134 | Ep-C8 |
| DUOX1 | 0.599 | 0.523 | 0.23 | 1.15E-138 | 2.94E-134 | Ep-C8 |
| RNF149 | 0.647 | 0.774 | 0.559 | 2.31E-138 | 5.92E-134 | Ep-C8 |
| ATP2A2 | 0.622 | 0.938 | 0.836 | 2.88E-138 | 7.39E-134 | Ep-C8 |
| RNF39 | 0.649 | 0.267 | 0.08 | 4.62E-138 | 1.18E-133 | Ep-C8 |
| ARHGAP32 | 0.573 | 0.575 | 0.256 | 1.20E-137 | 3.08E-133 | Ep-C8 |
| SEMA3F | 0.563 | 0.497 | 0.2 | 1.82E-136 | 4.66E-132 | Ep-C8 |
| PPDPF | 0.781 | 1 | 0.999 | 2.20E-136 | 5.65E-132 | Ep-C8 |
| CHST2 | 0.9 | 0.533 | 0.226 | 2.29E-136 | 5.86E-132 | Ep-C8 |
| FAM25A | 0.937 | 0.187 | 0.026 | 3.93E-136 | 1.01E-131 | Ep-C8 |
| CALM1 | 0.623 | 1 | 0.999 | 4.36E-136 | 1.12E-131 | Ep-C8 |
| RABIF | 0.61 | 0.612 | 0.352 | 1.82E-135 | 4.65E-131 | Ep-C8 |
| PITX1 | 0.809 | 0.986 | 0.946 | 3.50E-135 | 8.96E-131 | Ep-C8 |
| SPINK5 | 1.072 | 0.256 | 0.098 | 6.34E-135 | 1.62E-130 | Ep-C8 |
| KDM7A-DT | 0.644 | 0.574 | 0.277 | 7.84E-135 | 2.01E-130 | Ep-C8 |
| TECR | 0.641 | 0.994 | 0.982 | 1.79E-134 | 4.59E-130 | Ep-C8 |
| GFOD2 | 0.538 | 0.5 | 0.248 | 1.95E-134 | 4.99E-130 | Ep-C8 |
| NDUFA4L2 | 1.203 | 0.759 | 0.485 | 1.01E-133 | 2.58E-129 | Ep-C8 |
| KLF4 | 0.785 | 0.958 | 0.807 | 1.17E-133 | 3.00E-129 | Ep-C8 |
| EMP1 | 1.14 | 0.805 | 0.58 | 2.36E-133 | 6.05E-129 | Ep-C8 |
| FUT2 | 0.534 | 0.499 | 0.192 | 2.68E-133 | 6.86E-129 | Ep-C8 |
| LYPD2 | 2.161 | 0.139 | 0.01 | 7.78E-133 | 1.99E-128 | Ep-C8 |
| C5orf66-AS1 | 0.722 | 0.66 | 0.368 | 3.93E-132 | 1.01E-127 | Ep-C8 |
| S100A14 | 0.957 | 0.986 | 0.947 | 6.34E-132 | 1.63E-127 | Ep-C8 |
| PGM2 | 0.594 | 0.708 | 0.483 | 1.58E-131 | 4.04E-127 | Ep-C8 |
| RAB31 | 0.729 | 0.551 | 0.341 | 4.02E-131 | 1.03E-126 | Ep-C8 |
| SNX18 | 0.662 | 0.539 | 0.272 | 1.30E-130 | 3.34E-126 | Ep-C8 |
| WDR72 | 0.672 | 0.841 | 0.699 | 2.41E-130 | 6.17E-126 | Ep-C8 |
| CEL | 1.748 | 0.39 | 0.155 | 2.45E-130 | 6.27E-126 | Ep-C8 |
| GID8 | 0.578 | 0.864 | 0.777 | 7.54E-130 | 1.93E-125 | Ep-C8 |
| GPR160 | 0.752 | 0.477 | 0.172 | 9.97E-130 | 2.55E-125 | Ep-C8 |
| AC023157.3 | 1.128 | 0.497 | 0.31 | 2.67E-128 | 6.84E-124 | Ep-C8 |
| MIEN1 | 0.632 | 0.954 | 0.897 | 1.05E-125 | 2.69E-121 | Ep-C8 |
| TM7SF2 | 0.833 | 0.595 | 0.345 | 2.20E-125 | 5.64E-121 | Ep-C8 |
| APOL1 | 0.837 | 0.751 | 0.47 | 2.67E-124 | 6.84E-120 | Ep-C8 |
| HDHD3 | 0.596 | 0.558 | 0.282 | 2.71E-124 | 6.94E-120 | Ep-C8 |
| TXN | 0.823 | 0.999 | 1 | 3.72E-123 | 9.53E-119 | Ep-C8 |
| CEP170B | 0.758 | 0.708 | 0.41 | 3.82E-123 | 9.79E-119 | Ep-C8 |
| PPP1R11 | 0.596 | 0.737 | 0.471 | 1.36E-122 | 3.49E-118 | Ep-C8 |
| ZNF385A | 0.6 | 0.746 | 0.509 | 1.45E-122 | 3.71E-118 | Ep-C8 |
| ZMYND8 | 0.65 | 0.696 | 0.43 | 2.14E-122 | 5.49E-118 | Ep-C8 |
| BTBD6 | 0.749 | 0.806 | 0.665 | 7.82E-122 | 2.00E-117 | Ep-C8 |
| CNN3 | 0.718 | 0.54 | 0.333 | 1.14E-121 | 2.92E-117 | Ep-C8 |
| SRD5A3 | 0.827 | 0.585 | 0.365 | 1.42E-121 | 3.65E-117 | Ep-C8 |
| GNG5 | 0.526 | 0.996 | 0.989 | 4.29E-121 | 1.10E-116 | Ep-C8 |
| LMO7 | 0.954 | 0.372 | 0.246 | 2.24E-120 | 5.74E-116 | Ep-C8 |
| MFSD1 | 0.576 | 0.764 | 0.553 | 4.46E-120 | 1.14E-115 | Ep-C8 |
| ABI1 | 0.598 | 0.789 | 0.561 | 4.87E-120 | 1.25E-115 | Ep-C8 |
| PHLDB3 | 0.505 | 0.439 | 0.194 | 6.46E-120 | 1.66E-115 | Ep-C8 |
| SDR16C5 | 0.716 | 0.678 | 0.438 | 1.35E-119 | 3.45E-115 | Ep-C8 |
| LYN | 0.663 | 0.607 | 0.306 | 2.71E-119 | 6.96E-115 | Ep-C8 |
| ARRDC1 | 0.585 | 0.676 | 0.433 | 8.54E-119 | 2.19E-114 | Ep-C8 |
| DUOXA1 | 0.529 | 0.533 | 0.241 | 9.35E-119 | 2.40E-114 | Ep-C8 |
| IL1A | 0.791 | 0.285 | 0.076 | 1.20E-118 | 3.08E-114 | Ep-C8 |
| SCAMP2 | 0.563 | 0.655 | 0.449 | 1.66E-118 | 4.25E-114 | Ep-C8 |
| FAM162A | 0.818 | 0.973 | 0.945 | 4.77E-118 | 1.22E-113 | Ep-C8 |
| KLK7 | 0.675 | 0.193 | 0.043 | 8.28E-118 | 2.12E-113 | Ep-C8 |
| AMN | 0.635 | 0.704 | 0.435 | 1.07E-117 | 2.74E-113 | Ep-C8 |
| RORA | 0.663 | 0.671 | 0.371 | 4.07E-117 | 1.04E-112 | Ep-C8 |
| RAPGEFL1 | 0.597 | 0.697 | 0.395 | 1.37E-116 | 3.51E-112 | Ep-C8 |
| NOTCH3 | 0.632 | 0.684 | 0.378 | 1.77E-116 | 4.54E-112 | Ep-C8 |
| KRT4 | 1.136 | 0.174 | 0.025 | 1.32E-115 | 3.39E-111 | Ep-C8 |
| ATP6V1F | 0.531 | 0.995 | 0.954 | 2.16E-115 | 5.55E-111 | Ep-C8 |
| B3GALT4 | 0.557 | 0.532 | 0.276 | 3.17E-115 | 8.12E-111 | Ep-C8 |
| TOLLIP | 0.535 | 0.779 | 0.595 | 4.35E-115 | 1.12E-110 | Ep-C8 |
| ATP6V0C | 0.516 | 0.998 | 0.993 | 1.43E-114 | 3.65E-110 | Ep-C8 |
| TINF2 | 0.577 | 0.61 | 0.384 | 1.72E-114 | 4.40E-110 | Ep-C8 |
| TSPAN1 | 1.112 | 0.587 | 0.357 | 4.51E-114 | 1.16E-109 | Ep-C8 |
| KRT7 | 1.642 | 0.451 | 0.237 | 6.85E-114 | 1.75E-109 | Ep-C8 |
| ERMP1 | 0.535 | 0.439 | 0.2 | 1.25E-113 | 3.20E-109 | Ep-C8 |
| S100A6 | 0.936 | 1 | 1 | 2.73E-113 | 7.01E-109 | Ep-C8 |
| ANGPTL4 | 0.723 | 0.553 | 0.244 | 3.44E-113 | 8.82E-109 | Ep-C8 |
| SQOR | 0.616 | 0.81 | 0.578 | 2.94E-112 | 7.54E-108 | Ep-C8 |
| MGLL | 0.62 | 0.456 | 0.176 | 3.26E-112 | 8.36E-108 | Ep-C8 |
| UNC5B-AS1 | 0.906 | 0.419 | 0.18 | 3.98E-112 | 1.02E-107 | Ep-C8 |
| ABHD17C | 0.609 | 0.679 | 0.447 | 1.33E-111 | 3.40E-107 | Ep-C8 |
| SORT1 | 0.522 | 0.448 | 0.199 | 2.38E-111 | 6.10E-107 | Ep-C8 |
| TMEM184A | 0.571 | 0.457 | 0.187 | 5.92E-111 | 1.52E-106 | Ep-C8 |
| TMEM165 | 0.546 | 0.939 | 0.848 | 2.78E-110 | 7.12E-106 | Ep-C8 |
| ADM | 0.917 | 0.896 | 0.731 | 4.07E-109 | 1.04E-104 | Ep-C8 |
| TICAM1 | 0.554 | 0.5 | 0.259 | 1.28E-108 | 3.28E-104 | Ep-C8 |
| SERP1 | 0.526 | 0.999 | 0.998 | 1.15E-106 | 2.95E-102 | Ep-C8 |
| EPS8L2 | 0.579 | 0.752 | 0.51 | 1.58E-106 | 4.04E-102 | Ep-C8 |
| ZNF750 | 0.58 | 0.763 | 0.446 | 4.08E-106 | 1.05E-101 | Ep-C8 |
| CAMK2N1 | 0.778 | 0.503 | 0.238 | 6.46E-106 | 1.66E-101 | Ep-C8 |
| CRABP2 | 1.034 | 0.792 | 0.575 | 7.69E-106 | 1.97E-101 | Ep-C8 |
| DYNLL1 | 0.586 | 0.999 | 0.999 | 9.56E-106 | 2.45E-101 | Ep-C8 |
| TMBIM1 | 0.599 | 0.883 | 0.711 | 1.54E-105 | 3.95E-101 | Ep-C8 |
| TMEM159 | 0.585 | 0.81 | 0.6 | 1.77E-105 | 4.52E-101 | Ep-C8 |
| CDC42SE1 | 0.614 | 0.866 | 0.724 | 2.43E-105 | 6.22E-101 | Ep-C8 |
| NAGK | 0.554 | 0.664 | 0.395 | 2.58E-105 | 6.62E-101 | Ep-C8 |
| COBLL1 | 0.575 | 0.611 | 0.331 | 5.22E-105 | 1.34E-100 | Ep-C8 |
| RIT1 | 0.6 | 0.761 | 0.539 | 5.82E-105 | 1.49E-100 | Ep-C8 |
| UBE2G1 | 0.598 | 0.757 | 0.618 | 6.84E-105 | 1.75E-100 | Ep-C8 |
| VPS4B | 0.529 | 0.75 | 0.577 | 8.35E-105 | 2.14E-100 | Ep-C8 |
| CHP1 | 0.56 | 0.754 | 0.604 | 8.72E-105 | 2.23E-100 | Ep-C8 |
| MGST1 | 0.681 | 0.956 | 0.805 | 8.82E-105 | 2.26E-100 | Ep-C8 |
| H19 | 1.911 | 0.318 | 0.116 | 2.49E-104 | 6.38E-100 | Ep-C8 |
| CMPK1 | 0.542 | 0.925 | 0.798 | 3.00E-104 | 7.69E-100 | Ep-C8 |
| SERINC2 | 0.528 | 0.899 | 0.645 | 5.05E-104 | 1.29E-99 | Ep-C8 |
| CLCA2 | 0.741 | 0.902 | 0.733 | 1.43E-103 | 3.65E-99 | Ep-C8 |
| SERPINB2 | 0.734 | 0.203 | 0.039 | 1.62E-103 | 4.14E-99 | Ep-C8 |
| PPIC | 0.66 | 0.912 | 0.774 | 1.65E-103 | 4.22E-99 | Ep-C8 |
| CDCP1 | 0.632 | 0.682 | 0.451 | 2.44E-103 | 6.25E-99 | Ep-C8 |
| C12orf29 | 0.529 | 0.587 | 0.366 | 5.97E-103 | 1.53E-98 | Ep-C8 |
| B4GALT1 | 0.768 | 0.792 | 0.579 | 7.21E-103 | 1.85E-98 | Ep-C8 |
| NCK1 | 0.553 | 0.913 | 0.814 | 1.78E-102 | 4.56E-98 | Ep-C8 |
| SLC44A2 | 0.528 | 0.554 | 0.278 | 8.41E-102 | 2.16E-97 | Ep-C8 |
| GALNT3 | 0.552 | 0.682 | 0.434 | 1.01E-101 | 2.59E-97 | Ep-C8 |
| PTGR1 | 0.739 | 0.941 | 0.776 | 1.15E-101 | 2.95E-97 | Ep-C8 |
| AKT1 | 0.646 | 0.883 | 0.775 | 6.69E-101 | 1.71E-96 | Ep-C8 |
| CALML5 | 1.555 | 0.529 | 0.291 | 1.10E-100 | 2.82E-96 | Ep-C8 |
| ANXA3 | 0.753 | 0.491 | 0.221 | 4.30E-100 | 1.10E-95 | Ep-C8 |
| SLC6A8 | 0.837 | 0.825 | 0.69 | 2.08E-99 | 5.34E-95 | Ep-C8 |
| CCNDBP1 | 0.595 | 0.642 | 0.461 | 5.45E-99 | 1.40E-94 | Ep-C8 |
| SERTAD2 | 0.625 | 0.738 | 0.562 | 1.51E-98 | 3.88E-94 | Ep-C8 |
| SOWAHC | 0.638 | 0.756 | 0.536 | 1.67E-98 | 4.27E-94 | Ep-C8 |
| S100A10 | 0.678 | 0.998 | 0.998 | 1.67E-98 | 4.27E-94 | Ep-C8 |
| GPR87 | 0.587 | 0.815 | 0.539 | 7.94E-98 | 2.03E-93 | Ep-C8 |
| PLIN3 | 0.577 | 0.874 | 0.763 | 1.48E-97 | 3.79E-93 | Ep-C8 |
| HAS3 | 0.787 | 0.767 | 0.555 | 2.34E-97 | 5.99E-93 | Ep-C8 |
| GPCPD1 | 0.591 | 0.569 | 0.317 | 2.56E-97 | 6.57E-93 | Ep-C8 |
| TMOD3 | 0.532 | 0.858 | 0.707 | 3.17E-97 | 8.13E-93 | Ep-C8 |
| C9orf16 | 0.57 | 0.997 | 0.989 | 6.03E-97 | 1.55E-92 | Ep-C8 |
| ANXA11 | 0.559 | 0.934 | 0.888 | 7.53E-97 | 1.93E-92 | Ep-C8 |
| REEP4 | 0.586 | 0.75 | 0.531 | 3.79E-96 | 9.71E-92 | Ep-C8 |
| KCNK6 | 0.587 | 0.681 | 0.411 | 5.63E-96 | 1.44E-91 | Ep-C8 |
| TJP2 | 0.534 | 0.555 | 0.304 | 1.28E-95 | 3.27E-91 | Ep-C8 |
| MYO10 | 0.694 | 0.814 | 0.618 | 2.34E-95 | 5.99E-91 | Ep-C8 |
| UPK3BL1 | 1.209 | 0.662 | 0.548 | 7.40E-95 | 1.90E-90 | Ep-C8 |
| ARF6 | 0.539 | 0.92 | 0.852 | 9.32E-94 | 2.39E-89 | Ep-C8 |
| YPEL3 | 0.598 | 0.72 | 0.545 | 1.35E-93 | 3.45E-89 | Ep-C8 |
| LAPTM4A | 0.592 | 0.982 | 0.973 | 1.82E-93 | 4.66E-89 | Ep-C8 |
| AFDN | 0.55 | 0.644 | 0.443 | 2.75E-93 | 7.05E-89 | Ep-C8 |
| PTBP3 | 0.529 | 0.935 | 0.855 | 4.76E-92 | 1.22E-87 | Ep-C8 |
| UPP1 | 0.961 | 0.814 | 0.681 | 6.16E-92 | 1.58E-87 | Ep-C8 |
| BHLHE40 | 0.625 | 0.874 | 0.725 | 7.82E-92 | 2.00E-87 | Ep-C8 |
| ZNF117 | 0.505 | 0.281 | 0.084 | 1.13E-91 | 2.89E-87 | Ep-C8 |
| AKIRIN2 | 0.548 | 0.783 | 0.585 | 1.55E-91 | 3.97E-87 | Ep-C8 |
| GCHFR | 0.728 | 0.687 | 0.564 | 1.99E-91 | 5.10E-87 | Ep-C8 |
| ISG20 | 0.729 | 0.578 | 0.314 | 2.96E-91 | 7.59E-87 | Ep-C8 |
| RIOK3 | 0.529 | 0.927 | 0.851 | 1.16E-90 | 2.98E-86 | Ep-C8 |
| RNF11 | 0.511 | 0.665 | 0.44 | 2.22E-90 | 5.68E-86 | Ep-C8 |
| CDKN2B | 0.632 | 0.759 | 0.566 | 2.93E-90 | 7.50E-86 | Ep-C8 |
| AL445524.1 | 0.632 | 0.566 | 0.331 | 9.46E-90 | 2.42E-85 | Ep-C8 |
| TPRG1 | 0.932 | 0.459 | 0.244 | 1.66E-89 | 4.26E-85 | Ep-C8 |
| DENND2C | 0.558 | 0.737 | 0.469 | 2.10E-89 | 5.37E-85 | Ep-C8 |
| KRT31 | 0.747 | 0.422 | 0.172 | 3.44E-89 | 8.81E-85 | Ep-C8 |
| LRRFIP2 | 0.511 | 0.767 | 0.593 | 3.67E-89 | 9.41E-85 | Ep-C8 |
| DUSP5 | 0.697 | 0.503 | 0.276 | 6.37E-89 | 1.63E-84 | Ep-C8 |
| EDN2 | 1.358 | 0.34 | 0.123 | 4.42E-88 | 1.13E-83 | Ep-C8 |
| ERBB2 | 0.507 | 0.593 | 0.375 | 5.81E-88 | 1.49E-83 | Ep-C8 |
| GALE | 0.536 | 0.612 | 0.375 | 2.19E-87 | 5.62E-83 | Ep-C8 |
| TJP3 | 1.046 | 0.152 | 0.023 | 4.45E-87 | 1.14E-82 | Ep-C8 |
| HMGCS1 | 0.856 | 0.915 | 0.867 | 6.55E-87 | 1.68E-82 | Ep-C8 |
| DYNLT3 | 0.56 | 0.798 | 0.646 | 1.49E-86 | 3.81E-82 | Ep-C8 |
| KRT19 | 0.808 | 0.976 | 0.926 | 1.95E-86 | 4.99E-82 | Ep-C8 |
| MRPS30 | 0.705 | 0.828 | 0.744 | 2.93E-86 | 7.50E-82 | Ep-C8 |
| CFLAR | 0.541 | 0.726 | 0.549 | 4.28E-84 | 1.10E-79 | Ep-C8 |
| ELOVL1 | 0.559 | 0.74 | 0.603 | 1.22E-83 | 3.12E-79 | Ep-C8 |
| ATP1B1 | 0.769 | 0.988 | 0.975 | 1.75E-83 | 4.49E-79 | Ep-C8 |
| HS3ST1 | 0.516 | 0.352 | 0.127 | 5.35E-83 | 1.37E-78 | Ep-C8 |
| ULBP2 | 0.592 | 0.677 | 0.481 | 3.63E-81 | 9.29E-77 | Ep-C8 |
| FAM110C | 0.592 | 0.556 | 0.336 | 5.89E-79 | 1.51E-74 | Ep-C8 |
| HOXB2 | 0.55 | 0.605 | 0.378 | 1.33E-78 | 3.41E-74 | Ep-C8 |
| CD59 | 0.566 | 0.967 | 0.926 | 3.53E-78 | 9.05E-74 | Ep-C8 |
| MACC1 | 0.522 | 0.196 | 0.054 | 5.89E-78 | 1.51E-73 | Ep-C8 |
| BCL10 | 0.502 | 0.784 | 0.588 | 2.62E-77 | 6.72E-73 | Ep-C8 |
| CCDC80 | 0.886 | 0.351 | 0.146 | 2.94E-77 | 7.52E-73 | Ep-C8 |
| TMEM267 | 0.584 | 0.633 | 0.364 | 5.03E-77 | 1.29E-72 | Ep-C8 |
| CTSS | 0.6 | 0.28 | 0.093 | 6.13E-77 | 1.57E-72 | Ep-C8 |
| LGMN | 0.577 | 0.66 | 0.496 | 1.22E-76 | 3.12E-72 | Ep-C8 |
| RESF1 | 0.675 | 0.538 | 0.438 | 2.96E-75 | 7.58E-71 | Ep-C8 |
| TMEM154 | 0.571 | 0.606 | 0.371 | 3.85E-75 | 9.87E-71 | Ep-C8 |
| C1orf56 | 0.649 | 0.543 | 0.362 | 5.54E-75 | 1.42E-70 | Ep-C8 |
| SGK1 | 0.642 | 0.958 | 0.891 | 6.45E-75 | 1.65E-70 | Ep-C8 |
| ALDH1A3 | 0.718 | 0.323 | 0.163 | 7.98E-74 | 2.04E-69 | Ep-C8 |
| DCTN3 | 0.607 | 0.938 | 0.917 | 1.28E-73 | 3.27E-69 | Ep-C8 |
| PPP2R5C | 0.597 | 0.804 | 0.711 | 1.49E-73 | 3.81E-69 | Ep-C8 |
| ZFP36 | 0.681 | 0.955 | 0.865 | 2.17E-73 | 5.56E-69 | Ep-C8 |
| MYO6 | 0.533 | 0.627 | 0.429 | 5.35E-73 | 1.37E-68 | Ep-C8 |
| FLNB | 0.563 | 0.73 | 0.587 | 3.38E-71 | 8.66E-67 | Ep-C8 |
| ASPRV1 | 0.657 | 0.107 | 0.017 | 1.92E-70 | 4.92E-66 | Ep-C8 |
| IDH1 | 0.501 | 0.592 | 0.549 | 2.65E-70 | 6.80E-66 | Ep-C8 |
| TRNP1 | 0.675 | 0.292 | 0.186 | 9.60E-70 | 2.46E-65 | Ep-C8 |
| DEFB1 | 0.708 | 0.616 | 0.356 | 2.93E-69 | 7.51E-65 | Ep-C8 |
| GPRC5A | 0.723 | 0.499 | 0.297 | 4.49E-69 | 1.15E-64 | Ep-C8 |
| CRYAB | 0.88 | 0.654 | 0.432 | 8.47E-69 | 2.17E-64 | Ep-C8 |
| SERPINB5 | 0.532 | 0.956 | 0.865 | 2.25E-68 | 5.77E-64 | Ep-C8 |
| DSP | 0.588 | 0.992 | 0.976 | 7.84E-68 | 2.01E-63 | Ep-C8 |
| PAIP1 | 0.516 | 0.88 | 0.852 | 1.74E-67 | 4.47E-63 | Ep-C8 |
| RNASE1 | 0.663 | 0.236 | 0.085 | 2.41E-67 | 6.19E-63 | Ep-C8 |
| UBE2H | 0.518 | 0.842 | 0.757 | 8.37E-66 | 2.14E-61 | Ep-C8 |
| MYEOV | 0.734 | 0.223 | 0.111 | 3.30E-64 | 8.47E-60 | Ep-C8 |
| ARL4D | 0.612 | 0.746 | 0.554 | 3.71E-63 | 9.50E-59 | Ep-C8 |
| MIDN | 0.517 | 0.929 | 0.85 | 4.12E-63 | 1.06E-58 | Ep-C8 |
| HEPHL1 | 0.545 | 0.105 | 0.022 | 1.94E-62 | 4.98E-58 | Ep-C8 |
| ACKR3 | 0.762 | 0.65 | 0.502 | 1.70E-61 | 4.35E-57 | Ep-C8 |
| LGALS3 | 0.608 | 0.999 | 0.964 | 3.44E-61 | 8.83E-57 | Ep-C8 |
| STC2 | 0.516 | 0.344 | 0.163 | 5.16E-59 | 1.32E-54 | Ep-C8 |
| IGFL1 | 1.055 | 0.218 | 0.066 | 8.84E-59 | 2.26E-54 | Ep-C8 |
| CCL20 | 1.589 | 0.567 | 0.545 | 9.20E-59 | 2.36E-54 | Ep-C8 |
| OVOL1 | 0.524 | 0.554 | 0.33 | 2.56E-58 | 6.56E-54 | Ep-C8 |
| MAL | 0.95 | 0.111 | 0.085 | 3.79E-58 | 9.71E-54 | Ep-C8 |
| CCDC107 | 0.523 | 0.612 | 0.444 | 5.31E-57 | 1.36E-52 | Ep-C8 |
| APOBEC3A | 1.164 | 0.2 | 0.068 | 7.22E-56 | 1.85E-51 | Ep-C8 |
| MSLN | 0.711 | 0.127 | 0.034 | 6.31E-54 | 1.62E-49 | Ep-C8 |
| MKNK2 | 0.532 | 0.842 | 0.732 | 1.98E-52 | 5.08E-48 | Ep-C8 |
| KRT17 | 1.436 | 0.962 | 0.937 | 9.87E-51 | 2.53E-46 | Ep-C8 |
| GSTA1 | 1.916 | 0.413 | 0.246 | 2.86E-49 | 7.33E-45 | Ep-C8 |
| THBD | 0.548 | 0.504 | 0.293 | 1.12E-48 | 2.88E-44 | Ep-C8 |
| BAG1 | 0.502 | 0.924 | 0.892 | 2.92E-47 | 7.49E-43 | Ep-C8 |
| CXCL1 | 0.568 | 0.216 | 0.361 | 2.81E-46 | 7.19E-42 | Ep-C8 |
| TXNRD1 | 0.532 | 0.842 | 0.747 | 1.34E-45 | 3.44E-41 | Ep-C8 |
| SPRR2G | 1.42 | 0.102 | 0.024 | 1.64E-43 | 4.21E-39 | Ep-C8 |
| HCAR3 | 0.516 | 0.424 | 0.266 | 2.71E-43 | 6.94E-39 | Ep-C8 |
| PLAC8 | 0.818 | 0.244 | 0.156 | 8.68E-42 | 2.23E-37 | Ep-C8 |
| RDH10 | 0.665 | 0.521 | 0.407 | 1.07E-41 | 2.73E-37 | Ep-C8 |
| ISG15 | 0.713 | 0.877 | 0.81 | 3.67E-41 | 9.40E-37 | Ep-C8 |
| ALDH3A1 | 0.88 | 0.84 | 0.776 | 6.44E-41 | 1.65E-36 | Ep-C8 |
| GPX2 | 0.628 | 0.917 | 0.85 | 8.09E-41 | 2.07E-36 | Ep-C8 |
| NEAT1 | 0.57 | 0.998 | 0.999 | 6.70E-39 | 1.72E-34 | Ep-C8 |
| LY6D | 0.936 | 0.889 | 0.828 | 3.61E-38 | 9.26E-34 | Ep-C8 |
| PLAUR | 0.597 | 0.643 | 0.49 | 1.51E-36 | 3.87E-32 | Ep-C8 |
| PLAT | 0.723 | 0.668 | 0.615 | 3.55E-34 | 9.10E-30 | Ep-C8 |
| CXCL8 | 0.992 | 0.352 | 0.359 | 2.17E-32 | 5.56E-28 | Ep-C8 |
| IL1B | 0.922 | 0.295 | 0.173 | 3.32E-31 | 8.51E-27 | Ep-C8 |
| CA9 | 0.593 | 0.302 | 0.168 | 2.14E-30 | 5.48E-26 | Ep-C8 |
| DMKN | 0.522 | 0.747 | 0.703 | 7.13E-25 | 1.83E-20 | Ep-C8 |
| HOPX | 0.918 | 0.521 | 0.455 | 1.54E-21 | 3.95E-17 | Ep-C8 |
| PLAU | 0.507 | 0.742 | 0.683 | 3.64E-19 | 9.34E-15 | Ep-C8 |
| IGLC2 | 0.921 | 0.687 | 0.64 | 9.34E-19 | 2.39E-14 | Ep-C8 |
| FABP4 | 4.589 | 0.675 | 0.212 | 0 | 0 | Ep-C9 |
| S100A7 | 2.635 | 0.898 | 0.56 | 0 | 0 | Ep-C9 |
| KRT16 | 2.345 | 0.987 | 0.843 | 0 | 0 | Ep-C9 |
| S100A8 | 2.052 | 0.999 | 0.858 | 0 | 0 | Ep-C9 |
| RBP1 | 1.965 | 0.949 | 0.742 | 0 | 0 | Ep-C9 |
| KRT6B | 1.893 | 0.981 | 0.795 | 0 | 0 | Ep-C9 |
| S100A9 | 1.889 | 1 | 0.919 | 0 | 0 | Ep-C9 |
| FABP5 | 1.753 | 1 | 0.997 | 0 | 0 | Ep-C9 |
| GJB2 | 1.751 | 0.954 | 0.688 | 0 | 0 | Ep-C9 |
| PDZK1IP1 | 1.648 | 0.942 | 0.443 | 0 | 0 | Ep-C9 |
| BPGM | 1.6 | 0.763 | 0.356 | 1.09172108659533e-313 | 2.79819031705249e-309 | Ep-C9 |
| CYB5R1 | 1.065 | 0.811 | 0.446 | 1.87E-267 | 4.79E-263 | Ep-C9 |
| IL36G | 1.522 | 0.648 | 0.173 | 2.91E-258 | 7.45E-254 | Ep-C9 |
| TMEM132A | 0.987 | 0.814 | 0.511 | 8.78E-251 | 2.25E-246 | Ep-C9 |
| TUBA4A | 1.158 | 0.946 | 0.79 | 1.04E-244 | 2.67E-240 | Ep-C9 |
| PLAU | 1.289 | 0.923 | 0.673 | 6.54E-232 | 1.68E-227 | Ep-C9 |
| SFN | 1.026 | 1 | 0.998 | 3.18E-230 | 8.16E-226 | Ep-C9 |
| RHCG | 1.301 | 0.737 | 0.26 | 6.61E-230 | 1.69E-225 | Ep-C9 |
| ALDH2 | 1.143 | 0.873 | 0.641 | 7.52E-222 | 1.93E-217 | Ep-C9 |
| LGALS7B | 1.197 | 0.867 | 0.546 | 6.82E-219 | 1.75E-214 | Ep-C9 |
| TYMP | 1.018 | 0.997 | 0.949 | 9.89E-208 | 2.53E-203 | Ep-C9 |
| KRT14 | 1.325 | 0.988 | 0.827 | 3.64E-206 | 9.32E-202 | Ep-C9 |
| AMTN | 2.35 | 0.334 | 0.039 | 1.46E-205 | 3.75E-201 | Ep-C9 |
| CAPG | 0.842 | 0.969 | 0.816 | 1.99E-199 | 5.10E-195 | Ep-C9 |
| SERPINB1 | 1.209 | 0.961 | 0.802 | 3.86E-190 | 9.89E-186 | Ep-C9 |
| IL1RN | 1.181 | 0.648 | 0.222 | 3.07E-189 | 7.87E-185 | Ep-C9 |
| SAT1 | 1.218 | 0.999 | 0.988 | 6.76E-186 | 1.73E-181 | Ep-C9 |
| KRT6A | 1.032 | 1 | 0.999 | 1.91E-183 | 4.90E-179 | Ep-C9 |
| SERPINB4 | 1.835 | 0.724 | 0.349 | 9.69E-182 | 2.48E-177 | Ep-C9 |
| VAMP8 | 0.659 | 0.996 | 0.975 | 2.18E-178 | 5.59E-174 | Ep-C9 |
| LY6D | 1.556 | 0.955 | 0.825 | 6.34E-177 | 1.63E-172 | Ep-C9 |
| HCAR3 | 0.981 | 0.639 | 0.254 | 4.07E-176 | 1.04E-171 | Ep-C9 |
| THBD | 1.062 | 0.687 | 0.284 | 1.38E-174 | 3.53E-170 | Ep-C9 |
| SDCBP2 | 0.869 | 0.716 | 0.293 | 4.58E-173 | 1.17E-168 | Ep-C9 |
| CXCL14 | 1.637 | 0.855 | 0.619 | 1.51E-171 | 3.87E-167 | Ep-C9 |
| CTSB | 1.086 | 0.99 | 0.96 | 7.78E-170 | 1.99E-165 | Ep-C9 |
| DNAJB6 | 0.753 | 0.988 | 0.964 | 1.16E-167 | 2.98E-163 | Ep-C9 |
| DSG3 | 0.773 | 0.989 | 0.811 | 1.64E-166 | 4.21E-162 | Ep-C9 |
| C10orf99 | 1.381 | 0.58 | 0.201 | 7.58E-164 | 1.94E-159 | Ep-C9 |
| STRA6 | 0.524 | 0.307 | 0.044 | 1.95E-162 | 4.99E-158 | Ep-C9 |
| S100A2 | 1.205 | 1 | 0.997 | 1.17E-160 | 3.00E-156 | Ep-C9 |
| SERPINB3 | 1.382 | 0.862 | 0.598 | 2.97E-160 | 7.62E-156 | Ep-C9 |
| HCAR2 | 0.925 | 0.764 | 0.415 | 3.88E-159 | 9.96E-155 | Ep-C9 |
| SERPINA3 | 1.178 | 0.271 | 0.036 | 9.41E-159 | 2.41E-154 | Ep-C9 |
| ID1 | 0.975 | 0.979 | 0.823 | 2.93E-158 | 7.51E-154 | Ep-C9 |
| TUBB2A | 0.893 | 0.935 | 0.706 | 2.73E-157 | 6.99E-153 | Ep-C9 |
| IMPA2 | 0.725 | 0.966 | 0.857 | 4.10E-157 | 1.05E-152 | Ep-C9 |
| RAB38 | 0.743 | 0.888 | 0.663 | 4.49E-157 | 1.15E-152 | Ep-C9 |
| KRT17 | 1.336 | 0.989 | 0.935 | 2.03E-156 | 5.20E-152 | Ep-C9 |
| CD24 | 0.9 | 0.893 | 0.575 | 2.25E-156 | 5.76E-152 | Ep-C9 |
| GJB6 | 1.141 | 0.786 | 0.506 | 1.89E-154 | 4.85E-150 | Ep-C9 |
| FERMT1 | 0.971 | 0.844 | 0.612 | 1.48E-152 | 3.80E-148 | Ep-C9 |
| S100A6 | 0.889 | 1 | 1 | 4.67E-151 | 1.20E-146 | Ep-C9 |
| HES2 | 0.801 | 0.704 | 0.355 | 2.54E-150 | 6.50E-146 | Ep-C9 |
| PHLDA2 | 0.829 | 0.99 | 0.898 | 1.27E-149 | 3.25E-145 | Ep-C9 |
| CSTB | 0.61 | 1 | 1 | 7.49E-149 | 1.92E-144 | Ep-C9 |
| MMP7 | 2.129 | 0.527 | 0.189 | 8.51E-149 | 2.18E-144 | Ep-C9 |
| CDKN1A | 0.775 | 0.921 | 0.692 | 7.33E-145 | 1.88E-140 | Ep-C9 |
| PLAUR | 1.015 | 0.81 | 0.481 | 2.40E-144 | 6.16E-140 | Ep-C9 |
| KYNU | 0.94 | 0.706 | 0.353 | 1.79E-143 | 4.60E-139 | Ep-C9 |
| RGCC | 1.039 | 0.659 | 0.37 | 7.91E-142 | 2.03E-137 | Ep-C9 |
| KRT6C | 1.854 | 0.493 | 0.156 | 2.21E-141 | 5.67E-137 | Ep-C9 |
| SAA1 | 1.401 | 0.683 | 0.363 | 2.63E-141 | 6.75E-137 | Ep-C9 |
| CDH3 | 0.828 | 0.844 | 0.584 | 5.60E-141 | 1.43E-136 | Ep-C9 |
| IFNGR1 | 0.697 | 0.937 | 0.77 | 8.15E-140 | 2.09E-135 | Ep-C9 |
| PRDM1 | 0.691 | 0.56 | 0.197 | 1.11E-139 | 2.85E-135 | Ep-C9 |
| CAV2 | 0.773 | 0.915 | 0.726 | 5.31E-139 | 1.36E-134 | Ep-C9 |
| INHBA | 1.674 | 0.489 | 0.199 | 2.84E-137 | 7.28E-133 | Ep-C9 |
| PERP | 0.629 | 1 | 1 | 8.09E-136 | 2.07E-131 | Ep-C9 |
| UPP1 | 1.048 | 0.866 | 0.678 | 4.54E-135 | 1.16E-130 | Ep-C9 |
| APOBEC3A | 1.371 | 0.31 | 0.063 | 6.25E-135 | 1.60E-130 | Ep-C9 |
| HAS3 | 0.845 | 0.848 | 0.552 | 6.66E-134 | 1.71E-129 | Ep-C9 |
| SERPINB5 | 0.65 | 0.978 | 0.865 | 3.25E-133 | 8.32E-129 | Ep-C9 |
| CRABP2 | 1.028 | 0.858 | 0.572 | 4.71E-132 | 1.21E-127 | Ep-C9 |
| GRN | 0.656 | 0.989 | 0.96 | 5.03E-132 | 1.29E-127 | Ep-C9 |
| TMEM45A | 1.052 | 0.566 | 0.256 | 1.15E-131 | 2.95E-127 | Ep-C9 |
| SPRR2D | 0.717 | 0.637 | 0.272 | 1.77E-131 | 4.54E-127 | Ep-C9 |
| MAP7D1 | 0.716 | 0.851 | 0.602 | 4.44E-131 | 1.14E-126 | Ep-C9 |
| SPRR1B | 1.034 | 0.706 | 0.348 | 1.61E-130 | 4.13E-126 | Ep-C9 |
| SERPINB13 | 0.766 | 0.778 | 0.408 | 1.86E-130 | 4.76E-126 | Ep-C9 |
| PALMD | 0.832 | 0.571 | 0.258 | 1.19E-129 | 3.05E-125 | Ep-C9 |
| TMEM79 | 0.606 | 0.659 | 0.285 | 1.36E-129 | 3.48E-125 | Ep-C9 |
| RTN4 | 0.609 | 0.998 | 0.996 | 2.88E-129 | 7.37E-125 | Ep-C9 |
| OVOL1 | 0.826 | 0.654 | 0.326 | 6.17E-126 | 1.58E-121 | Ep-C9 |
| DSP | 0.768 | 0.997 | 0.976 | 1.78E-125 | 4.55E-121 | Ep-C9 |
| PRNP | 0.639 | 0.969 | 0.876 | 6.38E-125 | 1.64E-120 | Ep-C9 |
| ARPC1B | 0.719 | 0.981 | 0.959 | 6.33E-124 | 1.62E-119 | Ep-C9 |
| TACSTD2 | 0.721 | 0.993 | 0.935 | 2.75E-122 | 7.05E-118 | Ep-C9 |
| S100A10 | 0.652 | 1 | 0.998 | 9.05E-122 | 2.32E-117 | Ep-C9 |
| CD82 | 0.69 | 0.87 | 0.652 | 1.72E-119 | 4.40E-115 | Ep-C9 |
| CDC42EP1 | 0.675 | 0.707 | 0.421 | 2.60E-118 | 6.67E-114 | Ep-C9 |
| NDRG1 | 0.615 | 0.993 | 0.868 | 2.97E-117 | 7.62E-113 | Ep-C9 |
| GIPC1 | 0.554 | 0.967 | 0.87 | 5.12E-117 | 1.31E-112 | Ep-C9 |
| JUP | 0.551 | 1 | 0.992 | 2.53E-116 | 6.49E-112 | Ep-C9 |
| OAF | 0.589 | 0.529 | 0.252 | 9.31E-114 | 2.39E-109 | Ep-C9 |
| MXD1 | 0.704 | 0.788 | 0.506 | 1.83E-113 | 4.68E-109 | Ep-C9 |
| CITED4 | 0.802 | 0.827 | 0.556 | 9.18E-113 | 2.35E-108 | Ep-C9 |
| SQOR | 0.637 | 0.834 | 0.579 | 2.65E-112 | 6.79E-108 | Ep-C9 |
| TMSB10 | 0.508 | 1 | 1 | 5.56E-112 | 1.42E-107 | Ep-C9 |
| TMEM265 | 0.697 | 0.83 | 0.538 | 1.53E-111 | 3.93E-107 | Ep-C9 |
| ALDH1A3 | 0.738 | 0.431 | 0.158 | 3.51E-111 | 9.00E-107 | Ep-C9 |
| KLF7 | 0.588 | 0.663 | 0.326 | 7.76E-111 | 1.99E-106 | Ep-C9 |
| CD59 | 0.687 | 0.984 | 0.926 | 7.86E-111 | 2.01E-106 | Ep-C9 |
| SOD2 | 0.913 | 0.987 | 0.946 | 9.29E-111 | 2.38E-106 | Ep-C9 |
| GM2A | 0.619 | 0.776 | 0.496 | 8.28E-110 | 2.12E-105 | Ep-C9 |
| CLCA2 | 0.576 | 0.956 | 0.731 | 2.31E-108 | 5.92E-104 | Ep-C9 |
| GNA15 | 0.548 | 0.852 | 0.565 | 9.71E-107 | 2.49E-102 | Ep-C9 |
| CTSD | 0.542 | 0.997 | 0.978 | 4.92E-104 | 1.26E-99 | Ep-C9 |
| HOMER3 | 0.669 | 0.785 | 0.576 | 9.98E-104 | 2.56E-99 | Ep-C9 |
| MARCKS | 0.633 | 0.994 | 0.978 | 1.50E-103 | 3.85E-99 | Ep-C9 |
| LYPD3 | 0.694 | 0.932 | 0.72 | 1.98E-103 | 5.08E-99 | Ep-C9 |
| TMPRSS11D | 0.646 | 0.782 | 0.467 | 6.91E-103 | 1.77E-98 | Ep-C9 |
| PRSS8 | 0.523 | 0.665 | 0.334 | 8.30E-103 | 2.13E-98 | Ep-C9 |
| RIN2 | 0.622 | 0.668 | 0.386 | 1.45E-100 | 3.73E-96 | Ep-C9 |
| TFAP2A | 0.605 | 0.785 | 0.524 | 2.20E-99 | 5.64E-95 | Ep-C9 |
| EPAS1 | 0.542 | 0.658 | 0.339 | 7.04E-99 | 1.80E-94 | Ep-C9 |
| IL1B | 1.119 | 0.429 | 0.166 | 9.46E-98 | 2.42E-93 | Ep-C9 |
| AKR1B10 | 0.859 | 0.899 | 0.685 | 9.94E-98 | 2.55E-93 | Ep-C9 |
| FRMD4B | 0.622 | 0.645 | 0.372 | 1.94E-97 | 4.97E-93 | Ep-C9 |
| EIF1AY | 0.544 | 0.399 | 0.129 | 2.21E-97 | 5.66E-93 | Ep-C9 |
| WNT4 | 0.694 | 0.54 | 0.245 | 1.30E-95 | 3.33E-91 | Ep-C9 |
| KLK8 | 0.636 | 0.859 | 0.612 | 2.72E-95 | 6.96E-91 | Ep-C9 |
| BMP2 | 0.683 | 0.568 | 0.278 | 9.41E-94 | 2.41E-89 | Ep-C9 |
| SH3BGRL3 | 0.517 | 0.999 | 0.995 | 4.83E-93 | 1.24E-88 | Ep-C9 |
| TNFRSF12A | 0.644 | 0.9 | 0.693 | 2.04E-92 | 5.23E-88 | Ep-C9 |
| S100A14 | 0.756 | 0.989 | 0.947 | 6.32E-92 | 1.62E-87 | Ep-C9 |
| TINCR | 0.509 | 0.613 | 0.304 | 1.28E-91 | 3.28E-87 | Ep-C9 |
| MMP28 | 0.775 | 0.547 | 0.282 | 1.29E-91 | 3.31E-87 | Ep-C9 |
| KLC3 | 0.544 | 0.753 | 0.461 | 9.45E-91 | 2.42E-86 | Ep-C9 |
| CD68 | 0.629 | 0.699 | 0.41 | 3.33E-90 | 8.53E-86 | Ep-C9 |
| SGPP2 | 0.529 | 0.718 | 0.427 | 2.50E-89 | 6.41E-85 | Ep-C9 |
| DSC3 | 0.556 | 0.973 | 0.885 | 7.13E-89 | 1.83E-84 | Ep-C9 |
| ACKR3 | 0.82 | 0.733 | 0.498 | 1.17E-87 | 2.99E-83 | Ep-C9 |
| STK17A | 0.56 | 0.873 | 0.683 | 6.14E-87 | 1.57E-82 | Ep-C9 |
| MSN | 0.591 | 0.902 | 0.756 | 7.65E-87 | 1.96E-82 | Ep-C9 |
| GJA1 | 0.669 | 0.863 | 0.61 | 1.55E-86 | 3.98E-82 | Ep-C9 |
| CYB561A3 | 0.582 | 0.607 | 0.399 | 8.15E-85 | 2.09E-80 | Ep-C9 |
| PXDC1 | 0.561 | 0.688 | 0.397 | 4.28E-84 | 1.10E-79 | Ep-C9 |
| S100A7A | 0.964 | 0.266 | 0.065 | 4.68E-84 | 1.20E-79 | Ep-C9 |
| IGSF3 | 0.518 | 0.75 | 0.479 | 2.47E-83 | 6.33E-79 | Ep-C9 |
| ITGA6 | 0.665 | 0.949 | 0.831 | 1.15E-82 | 2.94E-78 | Ep-C9 |
| MMP1 | 2.012 | 0.227 | 0.124 | 3.41E-82 | 8.73E-78 | Ep-C9 |
| MYO1B | 0.571 | 0.729 | 0.478 | 4.48E-82 | 1.15E-77 | Ep-C9 |
| FLNB | 0.554 | 0.809 | 0.583 | 8.80E-82 | 2.25E-77 | Ep-C9 |
| SPRR2A | 0.655 | 0.662 | 0.407 | 1.51E-80 | 3.88E-76 | Ep-C9 |
| KCTD11 | 0.515 | 0.463 | 0.22 | 6.79E-79 | 1.74E-74 | Ep-C9 |
| FAM83A | 0.567 | 0.69 | 0.398 | 9.30E-79 | 2.38E-74 | Ep-C9 |
| GADD45A | 0.74 | 0.837 | 0.68 | 1.19E-78 | 3.04E-74 | Ep-C9 |
| SBSN | 0.646 | 0.289 | 0.08 | 1.78E-78 | 4.56E-74 | Ep-C9 |
| RAI14 | 0.556 | 0.515 | 0.266 | 2.71E-78 | 6.94E-74 | Ep-C9 |
| PLK3 | 0.593 | 0.683 | 0.418 | 8.54E-78 | 2.19E-73 | Ep-C9 |
| DENND2C | 0.523 | 0.741 | 0.471 | 7.24E-77 | 1.85E-72 | Ep-C9 |
| PON2 | 0.508 | 0.779 | 0.577 | 7.97E-75 | 2.04E-70 | Ep-C9 |
| JAG1 | 0.643 | 0.9 | 0.846 | 1.03E-74 | 2.64E-70 | Ep-C9 |
| DUSP14 | 0.574 | 0.775 | 0.548 | 1.32E-74 | 3.37E-70 | Ep-C9 |
| NRIP1 | 0.605 | 0.745 | 0.533 | 2.51E-74 | 6.44E-70 | Ep-C9 |
| MAFB | 0.623 | 0.822 | 0.617 | 3.51E-74 | 8.99E-70 | Ep-C9 |
| JARID2 | 0.501 | 0.7 | 0.47 | 3.54E-74 | 9.07E-70 | Ep-C9 |
| DSC2 | 0.639 | 0.801 | 0.56 | 8.49E-74 | 2.18E-69 | Ep-C9 |
| IGFL1 | 1.033 | 0.251 | 0.066 | 1.07E-73 | 2.74E-69 | Ep-C9 |
| TNFAIP8 | 0.552 | 0.799 | 0.631 | 4.58E-73 | 1.17E-68 | Ep-C9 |
| SDC1 | 0.542 | 0.987 | 0.914 | 2.10E-72 | 5.39E-68 | Ep-C9 |
| EFNB1 | 0.516 | 0.801 | 0.611 | 8.09E-71 | 2.07E-66 | Ep-C9 |
| ZNF750 | 0.676 | 0.697 | 0.452 | 9.86E-69 | 2.53E-64 | Ep-C9 |
| CTSC | 0.681 | 0.936 | 0.88 | 1.94E-68 | 4.97E-64 | Ep-C9 |
| TNS4 | 0.503 | 0.829 | 0.615 | 7.09E-68 | 1.82E-63 | Ep-C9 |
| CSRP2 | 0.743 | 0.838 | 0.764 | 1.30E-67 | 3.34E-63 | Ep-C9 |
| LIMA1 | 0.505 | 0.905 | 0.754 | 3.96E-67 | 1.01E-62 | Ep-C9 |
| ANXA3 | 0.543 | 0.472 | 0.224 | 1.23E-66 | 3.16E-62 | Ep-C9 |
| HBEGF | 0.545 | 0.782 | 0.554 | 2.08E-65 | 5.34E-61 | Ep-C9 |
| MGST1 | 0.503 | 0.951 | 0.806 | 9.34E-63 | 2.39E-58 | Ep-C9 |
| TINAGL1 | 0.635 | 0.798 | 0.614 | 1.25E-62 | 3.19E-58 | Ep-C9 |
| CRYAB | 0.597 | 0.675 | 0.433 | 1.52E-57 | 3.89E-53 | Ep-C9 |
| SERPINE1 | 0.743 | 0.488 | 0.262 | 1.94E-54 | 4.97E-50 | Ep-C9 |
| CDA | 0.551 | 0.38 | 0.173 | 2.49E-54 | 6.38E-50 | Ep-C9 |
| ATP1B3 | 0.559 | 1 | 0.998 | 1.09E-52 | 2.79E-48 | Ep-C9 |
| SLPI | 0.918 | 0.771 | 0.674 | 1.60E-50 | 4.10E-46 | Ep-C9 |
| CAPNS2 | 0.51 | 0.586 | 0.355 | 3.53E-50 | 9.05E-46 | Ep-C9 |
| ID2 | 0.591 | 0.533 | 0.325 | 2.18E-47 | 5.58E-43 | Ep-C9 |
| ANKRD33B | 0.557 | 0.322 | 0.22 | 2.21E-47 | 5.66E-43 | Ep-C9 |
| CLDN4 | 0.646 | 0.79 | 0.697 | 6.34E-47 | 1.62E-42 | Ep-C9 |
| TUBB3 | 0.567 | 0.593 | 0.386 | 4.37E-46 | 1.12E-41 | Ep-C9 |
| KLF6 | 0.516 | 0.859 | 0.778 | 1.19E-44 | 3.04E-40 | Ep-C9 |
| ANXA1 | 0.61 | 0.991 | 0.986 | 1.71E-44 | 4.40E-40 | Ep-C9 |
| FMO2 | 0.534 | 0.21 | 0.071 | 5.24E-43 | 1.34E-38 | Ep-C9 |
| DAPL1 | 0.547 | 0.872 | 0.762 | 3.23E-38 | 8.27E-34 | Ep-C9 |
| KRT75 | 0.537 | 0.149 | 0.054 | 3.79E-37 | 9.72E-33 | Ep-C9 |
| PI3 | 0.945 | 0.426 | 0.275 | 1.52E-34 | 3.89E-30 | Ep-C9 |
| MYO10 | 0.537 | 0.743 | 0.623 | 8.80E-30 | 2.25E-25 | Ep-C9 |
| ODC1 | 0.598 | 0.812 | 0.831 | 3.21E-28 | 8.22E-24 | Ep-C9 |
| APOE | 0.528 | 0.402 | 0.267 | 1.54E-25 | 3.95E-21 | Ep-C9 |
| MMP10 | 1.199 | 0.101 | 0.047 | 2.57E-21 | 6.58E-17 | Ep-C9 |
| MMP13 | 0.631 | 0.175 | 0.087 | 2.33E-20 | 5.96E-16 | Ep-C9 |
| IGFBP3 | 0.786 | 0.475 | 0.35 | 1.06E-18 | 2.73E-14 | Ep-C9 |
| HLA-DRB1 | 3.659 | 0.423 | 0.172 | 0 | 0 | Ep-C10 |
| CD74 | 2.9 | 0.519 | 0.231 | 0 | 0 | Ep-C10 |
| NUPR1 | 1.935 | 0.941 | 0.678 | 0 | 0 | Ep-C10 |
| IFIT3 | 2.173 | 0.555 | 0.191 | 8.75484324430689e-321 | 2.2439538719483e-316 | Ep-C10 |
| HLA-DRA | 2.834 | 0.497 | 0.257 | 2.83E-286 | 7.25E-282 | Ep-C10 |
| HLA-DPA1 | 2.36 | 0.326 | 0.087 | 2.56E-274 | 6.55E-270 | Ep-C10 |
| HLA-F | 1.278 | 0.684 | 0.381 | 3.63E-269 | 9.30E-265 | Ep-C10 |
| HLA-DPB1 | 1.643 | 0.313 | 0.072 | 5.36E-269 | 1.37E-264 | Ep-C10 |
| RTP4 | 1.178 | 0.636 | 0.286 | 2.33E-255 | 5.97E-251 | Ep-C10 |
| OASL | 2.52 | 0.442 | 0.127 | 1.69E-254 | 4.32E-250 | Ep-C10 |
| HLA-DQB1 | 1.48 | 0.351 | 0.103 | 5.24E-236 | 1.34E-231 | Ep-C10 |
| IFIT1 | 1.785 | 0.478 | 0.206 | 4.76E-223 | 1.22E-218 | Ep-C10 |
| HLA-DQA1 | 2.229 | 0.244 | 0.033 | 1.29E-218 | 3.31E-214 | Ep-C10 |
| C1S | 1.275 | 0.657 | 0.339 | 1.13E-213 | 2.90E-209 | Ep-C10 |
| RSAD2 | 1.583 | 0.361 | 0.052 | 3.22E-213 | 8.26E-209 | Ep-C10 |
| IL32 | 1.791 | 0.555 | 0.239 | 1.01E-211 | 2.59E-207 | Ep-C10 |
| IFIT2 | 2.401 | 0.343 | 0.07 | 6.56E-210 | 1.68E-205 | Ep-C10 |
| HLA-C | 1.099 | 1 | 0.992 | 6.72E-195 | 1.72E-190 | Ep-C10 |
| PLPP5 | 1.421 | 0.544 | 0.478 | 8.63E-194 | 2.21E-189 | Ep-C10 |
| HLA-A | 1.275 | 0.999 | 0.997 | 2.17E-193 | 5.57E-189 | Ep-C10 |
| TNFSF10 | 1.472 | 0.938 | 0.845 | 5.62E-192 | 1.44E-187 | Ep-C10 |
| WARS | 1.427 | 0.529 | 0.426 | 3.88E-190 | 9.94E-186 | Ep-C10 |
| BST2 | 1.608 | 0.711 | 0.406 | 1.58E-187 | 4.05E-183 | Ep-C10 |
| IFIH1 | 0.879 | 0.523 | 0.241 | 8.91E-187 | 2.28E-182 | Ep-C10 |
| UBD | 0.904 | 0.225 | 0.014 | 2.93E-180 | 7.51E-176 | Ep-C10 |
| GBP4 | 0.845 | 0.455 | 0.124 | 8.51E-178 | 2.18E-173 | Ep-C10 |
| XAF1 | 0.995 | 0.624 | 0.373 | 1.76E-177 | 4.51E-173 | Ep-C10 |
| IFI44L | 1.235 | 0.411 | 0.151 | 6.31E-177 | 1.62E-172 | Ep-C10 |
| TAP1 | 0.985 | 0.83 | 0.661 | 2.18E-173 | 5.59E-169 | Ep-C10 |
| IDO1 | 1.448 | 0.319 | 0.053 | 2.75E-173 | 7.04E-169 | Ep-C10 |
| IFI44 | 1.033 | 0.598 | 0.407 | 3.53E-173 | 9.05E-169 | Ep-C10 |
| PARP14 | 0.967 | 0.736 | 0.591 | 8.93E-173 | 2.29E-168 | Ep-C10 |
| TACC1 | 1.119 | 0.503 | 0.403 | 4.95E-169 | 1.27E-164 | Ep-C10 |
| ISG20 | 1.316 | 0.54 | 0.318 | 1.29E-168 | 3.32E-164 | Ep-C10 |
| HLA-DMA | 0.946 | 0.351 | 0.137 | 7.32E-168 | 1.88E-163 | Ep-C10 |
| DDX58 | 0.862 | 0.436 | 0.183 | 5.09E-164 | 1.30E-159 | Ep-C10 |
| PLAAT4 | 1.12 | 0.672 | 0.348 | 4.86E-161 | 1.24E-156 | Ep-C10 |
| IKBKB | 0.68 | 0.407 | 0.275 | 1.93E-155 | 4.95E-151 | Ep-C10 |
| MX1 | 1.154 | 0.76 | 0.566 | 1.47E-154 | 3.76E-150 | Ep-C10 |
| CMPK2 | 0.718 | 0.338 | 0.083 | 4.44E-154 | 1.14E-149 | Ep-C10 |
| HLA-E | 0.812 | 0.998 | 0.975 | 7.97E-153 | 2.04E-148 | Ep-C10 |
| HLA-DMB | 0.637 | 0.241 | 0.036 | 7.17E-152 | 1.84E-147 | Ep-C10 |
| HERC5 | 0.983 | 0.32 | 0.165 | 1.01E-144 | 2.60E-140 | Ep-C10 |
| TRIM22 | 0.792 | 0.447 | 0.187 | 5.95E-144 | 1.53E-139 | Ep-C10 |
| C1R | 0.918 | 0.731 | 0.455 | 1.11E-143 | 2.85E-139 | Ep-C10 |
| HLA-B | 1.149 | 1 | 0.98 | 2.38E-136 | 6.10E-132 | Ep-C10 |
| PSMB9 | 0.95 | 0.878 | 0.826 | 9.54E-136 | 2.45E-131 | Ep-C10 |
| LIME1 | 0.884 | 0.496 | 0.279 | 1.12E-134 | 2.87E-130 | Ep-C10 |
| OAS3 | 0.809 | 0.522 | 0.317 | 2.16E-134 | 5.54E-130 | Ep-C10 |
| LAMP3 | 0.93 | 0.346 | 0.171 | 9.84E-134 | 2.52E-129 | Ep-C10 |
| CTSS | 0.788 | 0.316 | 0.093 | 1.61E-128 | 4.13E-124 | Ep-C10 |
| GOLGA7 | 0.623 | 0.652 | 0.567 | 4.59E-124 | 1.18E-119 | Ep-C10 |
| EPSTI1 | 0.733 | 0.37 | 0.137 | 4.29E-123 | 1.10E-118 | Ep-C10 |
| SAA1 | 1.588 | 0.645 | 0.366 | 6.48E-123 | 1.66E-118 | Ep-C10 |
| SRD5A1 | 0.673 | 0.55 | 0.368 | 1.21E-122 | 3.10E-118 | Ep-C10 |
| FNTA | 0.792 | 0.863 | 0.811 | 4.70E-121 | 1.20E-116 | Ep-C10 |
| TAPBP | 0.677 | 0.947 | 0.845 | 1.05E-120 | 2.69E-116 | Ep-C10 |
| PLSCR1 | 0.893 | 0.843 | 0.77 | 1.18E-119 | 3.02E-115 | Ep-C10 |
| ALDH2 | 0.897 | 0.811 | 0.645 | 4.04E-118 | 1.03E-113 | Ep-C10 |
| SP100 | 0.664 | 0.762 | 0.619 | 5.88E-118 | 1.51E-113 | Ep-C10 |
| CXCL10 | 2.415 | 0.206 | 0.036 | 3.18E-117 | 8.16E-113 | Ep-C10 |
| ISG15 | 1.857 | 0.838 | 0.812 | 1.93E-116 | 4.95E-112 | Ep-C10 |
| HGSNAT | 0.607 | 0.497 | 0.34 | 3.99E-116 | 1.02E-111 | Ep-C10 |
| OAS2 | 0.701 | 0.523 | 0.28 | 1.14E-115 | 2.93E-111 | Ep-C10 |
| NFIC | 0.85 | 0.735 | 0.69 | 9.90E-113 | 2.54E-108 | Ep-C10 |
| TLE5 | 0.868 | 0.966 | 0.945 | 1.57E-112 | 4.01E-108 | Ep-C10 |
| GPNMB | 1.002 | 0.943 | 0.838 | 3.49E-111 | 8.95E-107 | Ep-C10 |
| LRG1 | 0.672 | 0.294 | 0.119 | 6.11E-111 | 1.57E-106 | Ep-C10 |
| PPP1R12B | 0.576 | 0.329 | 0.149 | 1.08E-109 | 2.76E-105 | Ep-C10 |
| SYNPO2 | 0.714 | 0.22 | 0.049 | 6.92E-109 | 1.77E-104 | Ep-C10 |
| PARP9 | 0.725 | 0.65 | 0.528 | 9.02E-107 | 2.31E-102 | Ep-C10 |
| OPTN | 0.681 | 0.721 | 0.523 | 1.24E-106 | 3.19E-102 | Ep-C10 |
| ZC3HAV1 | 0.788 | 0.588 | 0.409 | 5.14E-106 | 1.32E-101 | Ep-C10 |
| HCP5 | 0.633 | 0.542 | 0.375 | 1.61E-104 | 4.13E-100 | Ep-C10 |
| MED29 | 0.624 | 0.529 | 0.474 | 3.16E-104 | 8.11E-100 | Ep-C10 |
| DHRS3 | 0.741 | 0.819 | 0.701 | 6.97E-104 | 1.79E-99 | Ep-C10 |
| IRF1 | 0.801 | 0.912 | 0.761 | 2.17E-103 | 5.57E-99 | Ep-C10 |
| TM2D2 | 0.629 | 0.637 | 0.585 | 3.61E-103 | 9.25E-99 | Ep-C10 |
| DDX60 | 0.861 | 0.46 | 0.24 | 1.18E-101 | 3.03E-97 | Ep-C10 |
| FOXQ1 | 1.018 | 0.614 | 0.424 | 2.19E-101 | 5.62E-97 | Ep-C10 |
| GBP1 | 1.017 | 0.648 | 0.406 | 2.80E-101 | 7.17E-97 | Ep-C10 |
| UBE2L6 | 0.692 | 0.81 | 0.691 | 5.58E-101 | 1.43E-96 | Ep-C10 |
| PNRC1 | 0.672 | 0.969 | 0.92 | 3.36E-100 | 8.62E-96 | Ep-C10 |
| RNF213 | 0.654 | 0.747 | 0.605 | 7.56E-99 | 1.94E-94 | Ep-C10 |
| MIR99AHG | 0.882 | 0.467 | 0.228 | 8.74E-99 | 2.24E-94 | Ep-C10 |
| PMAIP1 | 1.058 | 0.922 | 0.81 | 4.85E-98 | 1.24E-93 | Ep-C10 |
| SOD2 | 1.036 | 0.983 | 0.947 | 1.79E-96 | 4.58E-92 | Ep-C10 |
| LGALS9 | 0.549 | 0.338 | 0.188 | 8.54E-95 | 2.19E-90 | Ep-C10 |
| NSD3 | 0.78 | 0.865 | 0.822 | 1.42E-94 | 3.63E-90 | Ep-C10 |
| SAMD4B | 0.685 | 0.688 | 0.606 | 1.52E-94 | 3.90E-90 | Ep-C10 |
| B2M | 0.638 | 1 | 1 | 1.34E-92 | 3.44E-88 | Ep-C10 |
| GALNT11 | 0.579 | 0.492 | 0.353 | 1.35E-91 | 3.46E-87 | Ep-C10 |
| HMG20B | 0.668 | 0.749 | 0.72 | 1.98E-90 | 5.07E-86 | Ep-C10 |
| KYNU | 0.8 | 0.631 | 0.359 | 2.70E-89 | 6.92E-85 | Ep-C10 |
| GBP2 | 0.747 | 0.671 | 0.394 | 3.04E-89 | 7.78E-85 | Ep-C10 |
| MMP7 | 1.561 | 0.424 | 0.196 | 3.12E-89 | 8.00E-85 | Ep-C10 |
| STAT1 | 0.706 | 0.706 | 0.638 | 4.77E-89 | 1.22E-84 | Ep-C10 |
| ZNFX1 | 0.528 | 0.521 | 0.317 | 1.13E-87 | 2.91E-83 | Ep-C10 |
| PYCARD | 0.666 | 0.944 | 0.867 | 1.31E-87 | 3.35E-83 | Ep-C10 |
| IFI35 | 0.667 | 0.631 | 0.474 | 1.02E-85 | 2.61E-81 | Ep-C10 |
| CXCL16 | 0.554 | 0.557 | 0.415 | 4.87E-84 | 1.25E-79 | Ep-C10 |
| CFLAR | 0.589 | 0.712 | 0.552 | 1.67E-83 | 4.28E-79 | Ep-C10 |
| MX2 | 0.621 | 0.406 | 0.24 | 3.84E-82 | 9.83E-78 | Ep-C10 |
| PCDH7 | 0.603 | 0.441 | 0.2 | 2.33E-81 | 5.97E-77 | Ep-C10 |
| STAP2 | 0.567 | 0.727 | 0.596 | 2.82E-81 | 7.22E-77 | Ep-C10 |
| SCPEP1 | 0.586 | 0.953 | 0.911 | 3.91E-81 | 1.00E-76 | Ep-C10 |
| SUPT5H | 0.598 | 0.618 | 0.557 | 1.44E-80 | 3.70E-76 | Ep-C10 |
| TYMP | 0.709 | 0.995 | 0.95 | 1.80E-80 | 4.61E-76 | Ep-C10 |
| SPATS2L | 0.622 | 0.787 | 0.661 | 1.90E-80 | 4.87E-76 | Ep-C10 |
| SAMD9L | 0.544 | 0.347 | 0.143 | 4.92E-80 | 1.26E-75 | Ep-C10 |
| POLR2J3 | 0.669 | 0.686 | 0.605 | 4.09E-78 | 1.05E-73 | Ep-C10 |
| PTPRZ1 | 0.602 | 0.679 | 0.486 | 1.14E-77 | 2.93E-73 | Ep-C10 |
| SMIM19 | 0.714 | 0.799 | 0.742 | 4.76E-76 | 1.22E-71 | Ep-C10 |
| PSMB10 | 0.586 | 0.788 | 0.645 | 6.21E-75 | 1.59E-70 | Ep-C10 |
| CEBPB | 0.707 | 0.988 | 0.934 | 4.52E-74 | 1.16E-69 | Ep-C10 |
| IRF7 | 0.544 | 0.515 | 0.351 | 8.13E-74 | 2.08E-69 | Ep-C10 |
| PHLDA3 | 0.602 | 0.936 | 0.793 | 1.36E-72 | 3.49E-68 | Ep-C10 |
| DTX2 | 0.526 | 0.603 | 0.426 | 2.32E-70 | 5.96E-66 | Ep-C10 |
| ARL6IP5 | 0.533 | 0.79 | 0.657 | 5.22E-69 | 1.34E-64 | Ep-C10 |
| LSM1 | 0.529 | 0.805 | 0.814 | 6.55E-69 | 1.68E-64 | Ep-C10 |
| IFITM3 | 0.684 | 0.998 | 0.98 | 8.60E-69 | 2.21E-64 | Ep-C10 |
| RND3 | 0.763 | 0.923 | 0.801 | 1.65E-68 | 4.24E-64 | Ep-C10 |
| MIR22HG | 0.562 | 0.596 | 0.378 | 2.72E-66 | 6.96E-62 | Ep-C10 |
| SLFN5 | 0.64 | 0.627 | 0.446 | 1.94E-65 | 4.97E-61 | Ep-C10 |
| MIR205HG | 1.012 | 0.998 | 0.99 | 4.91E-65 | 1.26E-60 | Ep-C10 |
| SOX4 | 0.749 | 0.919 | 0.834 | 5.09E-65 | 1.30E-60 | Ep-C10 |
| MALAT1 | 0.747 | 1 | 1 | 7.54E-65 | 1.93E-60 | Ep-C10 |
| CH25H | 0.659 | 0.175 | 0.042 | 6.50E-63 | 1.66E-58 | Ep-C10 |
| EVA1C | 0.517 | 0.425 | 0.232 | 1.24E-62 | 3.17E-58 | Ep-C10 |
| FMO2 | 1.028 | 0.223 | 0.071 | 5.86E-62 | 1.50E-57 | Ep-C10 |
| NEAT1 | 0.653 | 1 | 0.999 | 1.01E-61 | 2.59E-57 | Ep-C10 |
| AIM2 | 0.909 | 0.408 | 0.229 | 3.67E-61 | 9.40E-57 | Ep-C10 |
| SCO2 | 0.501 | 0.813 | 0.67 | 5.60E-61 | 1.44E-56 | Ep-C10 |
| SNHG5 | 0.544 | 0.93 | 0.844 | 5.12E-59 | 1.31E-54 | Ep-C10 |
| N4BP1 | 0.514 | 0.862 | 0.751 | 6.18E-59 | 1.58E-54 | Ep-C10 |
| BTG2 | 0.686 | 0.897 | 0.795 | 9.32E-59 | 2.39E-54 | Ep-C10 |
| OAS1 | 0.71 | 0.558 | 0.379 | 5.05E-58 | 1.30E-53 | Ep-C10 |
| MT-CO3 | 0.569 | 1 | 1 | 2.01E-57 | 5.15E-53 | Ep-C10 |
| TACSTD2 | 0.804 | 0.967 | 0.936 | 5.27E-57 | 1.35E-52 | Ep-C10 |
| EIF2AK2 | 0.503 | 0.8 | 0.725 | 1.08E-56 | 2.76E-52 | Ep-C10 |
| ELF3 | 0.788 | 0.923 | 0.887 | 2.35E-56 | 6.02E-52 | Ep-C10 |
| CX3CL1 | 0.517 | 0.371 | 0.214 | 7.98E-56 | 2.04E-51 | Ep-C10 |
| PLAUR | 0.83 | 0.609 | 0.493 | 1.27E-55 | 3.24E-51 | Ep-C10 |
| TOB1 | 0.522 | 0.859 | 0.735 | 1.27E-55 | 3.25E-51 | Ep-C10 |
| ZFP36L2 | 0.615 | 0.83 | 0.711 | 9.74E-55 | 2.50E-50 | Ep-C10 |
| CFB | 0.566 | 0.394 | 0.196 | 3.69E-54 | 9.45E-50 | Ep-C10 |
| STAT2 | 0.501 | 0.539 | 0.38 | 7.23E-54 | 1.85E-49 | Ep-C10 |
| ADH5 | 0.598 | 0.96 | 0.943 | 5.95E-53 | 1.53E-48 | Ep-C10 |
| TXNIP | 0.782 | 0.713 | 0.547 | 9.66E-53 | 2.48E-48 | Ep-C10 |
| MT-CYB | 0.503 | 1 | 1 | 2.09E-52 | 5.35E-48 | Ep-C10 |
| BTG1 | 0.601 | 0.99 | 0.985 | 7.57E-52 | 1.94E-47 | Ep-C10 |
| CLDN1 | 0.658 | 0.844 | 0.7 | 4.45E-51 | 1.14E-46 | Ep-C10 |
| ADRB2 | 0.561 | 0.476 | 0.28 | 2.98E-50 | 7.63E-46 | Ep-C10 |
| TAX1BP3 | 0.674 | 0.892 | 0.825 | 4.43E-50 | 1.14E-45 | Ep-C10 |
| TCIM | 0.992 | 0.265 | 0.142 | 1.24E-49 | 3.19E-45 | Ep-C10 |
| NCOA7 | 0.61 | 0.903 | 0.806 | 9.84E-49 | 2.52E-44 | Ep-C10 |
| LY6E | 0.585 | 0.996 | 0.987 | 2.44E-48 | 6.26E-44 | Ep-C10 |
| DDIT3 | 0.638 | 0.804 | 0.721 | 5.73E-47 | 1.47E-42 | Ep-C10 |
| GADD45B | 0.773 | 0.782 | 0.693 | 3.36E-46 | 8.61E-42 | Ep-C10 |
| SPAG9 | 0.606 | 0.813 | 0.752 | 1.62E-45 | 4.15E-41 | Ep-C10 |
| ADH1C | 1.402 | 0.244 | 0.148 | 1.32E-44 | 3.39E-40 | Ep-C10 |
| TNFAIP3 | 0.571 | 0.825 | 0.7 | 1.74E-44 | 4.47E-40 | Ep-C10 |
| ID2 | 0.649 | 0.495 | 0.328 | 2.70E-44 | 6.92E-40 | Ep-C10 |
| SAMD9 | 0.634 | 0.784 | 0.631 | 3.51E-43 | 9.00E-39 | Ep-C10 |
| MARCKSL1 | 0.505 | 0.942 | 0.856 | 1.96E-41 | 5.04E-37 | Ep-C10 |
| ATF3 | 0.556 | 0.854 | 0.76 | 2.42E-41 | 6.20E-37 | Ep-C10 |
| TSC22D1 | 0.542 | 0.881 | 0.782 | 7.69E-41 | 1.97E-36 | Ep-C10 |
| BIRC3 | 0.504 | 0.327 | 0.187 | 5.23E-39 | 1.34E-34 | Ep-C10 |
| RAB11FIP1 | 0.757 | 0.555 | 0.505 | 1.33E-37 | 3.42E-33 | Ep-C10 |
| ZFP36 | 0.632 | 0.935 | 0.867 | 8.14E-37 | 2.09E-32 | Ep-C10 |
| ZNF750 | 0.553 | 0.488 | 0.464 | 4.81E-36 | 1.23E-31 | Ep-C10 |
| IFI6 | 0.932 | 0.756 | 0.732 | 1.09E-35 | 2.78E-31 | Ep-C10 |
| KLF6 | 0.604 | 0.825 | 0.78 | 2.95E-34 | 7.55E-30 | Ep-C10 |
| IL1R2 | 0.877 | 0.357 | 0.213 | 9.89E-32 | 2.53E-27 | Ep-C10 |
| CCDC80 | 0.543 | 0.277 | 0.152 | 3.62E-31 | 9.27E-27 | Ep-C10 |
| GADD45A | 0.716 | 0.761 | 0.685 | 2.67E-30 | 6.85E-26 | Ep-C10 |
| PTGS2 | 0.533 | 0.21 | 0.122 | 9.30E-30 | 2.38E-25 | Ep-C10 |
| C11orf96 | 0.815 | 0.261 | 0.17 | 3.70E-28 | 9.48E-24 | Ep-C10 |
| KRT15 | 0.807 | 0.903 | 0.862 | 3.28E-26 | 8.40E-22 | Ep-C10 |
| HES1 | 0.539 | 0.902 | 0.862 | 1.81E-15 | 4.63E-11 | Ep-C10 |
| EIF4EBP1 | 0.523 | 0.885 | 0.902 | 3.24E-10 | 8.31E-06 | Ep-C10 |
| FKBP4 | 1.642 | 0.932 | 0.785 | 0 | 0 | Ep-C11 |
| PHB2 | 1.201 | 0.998 | 0.959 | 5.53353523342196e-322 | 1.41830041567838e-317 | Ep-C11 |
| LDHB | 1.357 | 1 | 0.978 | 1.64646175897076e-316 | 4.22004613441796e-312 | Ep-C11 |
| NDUFA9 | 1.165 | 0.943 | 0.784 | 7.09818017307072e-310 | 1.82E-305 | Ep-C11 |
| COX6B1 | 1.215 | 1 | 0.999 | 2.19E-298 | 5.61E-294 | Ep-C11 |
| POLR2I | 1.329 | 0.974 | 0.91 | 4.01E-286 | 1.03E-281 | Ep-C11 |
| EIF3K | 0.91 | 1 | 0.999 | 4.72E-286 | 1.21E-281 | Ep-C11 |
| STRAP | 1.11 | 0.983 | 0.925 | 3.04E-279 | 7.80E-275 | Ep-C11 |
| FGF19 | 2.875 | 0.57 | 0.119 | 9.37E-278 | 2.40E-273 | Ep-C11 |
| EMG1 | 1.119 | 0.915 | 0.737 | 7.17E-268 | 1.84E-263 | Ep-C11 |
| MRPL51 | 1.034 | 1 | 0.987 | 3.74E-256 | 9.57E-252 | Ep-C11 |
| RBM42 | 1.212 | 0.857 | 0.65 | 3.71E-247 | 9.51E-243 | Ep-C11 |
| MRPS12 | 1.029 | 0.984 | 0.932 | 6.60E-246 | 1.69E-241 | Ep-C11 |
| MAGEB2 | 1.077 | 0.504 | 0.086 | 7.36E-236 | 1.89E-231 | Ep-C11 |
| SHKBP1 | 1.199 | 0.775 | 0.483 | 5.51E-235 | 1.41E-230 | Ep-C11 |
| COX8C | 1.315 | 0.526 | 0.094 | 9.24E-233 | 2.37E-228 | Ep-C11 |
| M6PR | 0.962 | 0.888 | 0.713 | 1.59E-224 | 4.08E-220 | Ep-C11 |
| TPI1 | 0.895 | 1 | 1 | 6.23E-220 | 1.60E-215 | Ep-C11 |
| MRPL12 | 0.866 | 0.989 | 0.931 | 1.90E-218 | 4.88E-214 | Ep-C11 |
| TBCB | 1.056 | 0.94 | 0.864 | 4.67E-217 | 1.20E-212 | Ep-C11 |
| FGF3 | 1.19 | 0.42 | 0.058 | 4.36E-215 | 1.12E-210 | Ep-C11 |
| TIMM50 | 1.039 | 0.844 | 0.594 | 6.16E-215 | 1.58E-210 | Ep-C11 |
| UBA2 | 1.046 | 0.927 | 0.805 | 6.53E-215 | 1.67E-210 | Ep-C11 |
| SAP25 | 1.152 | 0.534 | 0.125 | 2.31E-214 | 5.92E-210 | Ep-C11 |
| XRCC6 | 0.833 | 0.992 | 0.962 | 2.87E-213 | 7.35E-209 | Ep-C11 |
| MEST | 1.191 | 0.673 | 0.336 | 3.30E-213 | 8.45E-209 | Ep-C11 |
| KRT18 | 1.384 | 0.995 | 0.978 | 7.13E-213 | 1.83E-208 | Ep-C11 |
| SOHLH2 | 0.823 | 0.455 | 0.074 | 1.33E-212 | 3.41E-208 | Ep-C11 |
| PDCD5 | 0.978 | 0.998 | 0.971 | 1.42E-212 | 3.64E-208 | Ep-C11 |
| AC010198.2 | 0.926 | 0.477 | 0.083 | 4.55E-210 | 1.17E-205 | Ep-C11 |
| MCM5 | 1.048 | 0.771 | 0.385 | 3.59E-208 | 9.21E-204 | Ep-C11 |
| LINC00665 | 0.999 | 0.63 | 0.275 | 9.45E-208 | 2.42E-203 | Ep-C11 |
| MRPL21 | 1.502 | 0.985 | 0.92 | 4.63E-203 | 1.19E-198 | Ep-C11 |
| ELOB | 0.661 | 1 | 1 | 4.89E-201 | 1.25E-196 | Ep-C11 |
| COPS7A | 0.922 | 0.815 | 0.549 | 1.00E-199 | 2.58E-195 | Ep-C11 |
| GTSF1 | 0.879 | 0.479 | 0.09 | 2.19E-199 | 5.62E-195 | Ep-C11 |
| AC009509.1 | 0.692 | 0.428 | 0.067 | 3.72E-198 | 9.54E-194 | Ep-C11 |
| AP003555.1 | 0.965 | 0.477 | 0.095 | 6.52E-196 | 1.67E-191 | Ep-C11 |
| FBL | 0.861 | 0.995 | 0.925 | 1.59E-195 | 4.08E-191 | Ep-C11 |
| HSP90AA1 | 0.994 | 1 | 1 | 1.67E-194 | 4.29E-190 | Ep-C11 |
| UQCRFS1 | 0.809 | 0.989 | 0.961 | 6.39E-194 | 1.64E-189 | Ep-C11 |
| HEBP1 | 0.883 | 0.801 | 0.588 | 6.70E-193 | 1.72E-188 | Ep-C11 |
| PSMD8 | 0.789 | 1 | 0.995 | 2.88E-192 | 7.38E-188 | Ep-C11 |
| ATP5F1A | 0.777 | 0.991 | 0.944 | 7.04E-191 | 1.81E-186 | Ep-C11 |
| WBP11 | 0.924 | 0.813 | 0.575 | 3.08E-190 | 7.88E-186 | Ep-C11 |
| CCND1 | 1.469 | 0.979 | 0.924 | 4.53E-190 | 1.16E-185 | Ep-C11 |
| RRP7A | 0.859 | 0.84 | 0.607 | 4.23E-189 | 1.08E-184 | Ep-C11 |
| TOMM22 | 0.727 | 0.993 | 0.951 | 1.63E-188 | 4.18E-184 | Ep-C11 |
| DHCR7 | 1.147 | 0.844 | 0.599 | 2.17E-188 | 5.57E-184 | Ep-C11 |
| CYC1 | 0.756 | 0.997 | 0.978 | 6.22E-187 | 1.59E-182 | Ep-C11 |
| SFRP2 | 1.424 | 0.386 | 0.06 | 4.46E-184 | 1.14E-179 | Ep-C11 |
| HNRNPUL1 | 0.939 | 0.891 | 0.745 | 2.84E-181 | 7.27E-177 | Ep-C11 |
| CDH2 | 0.74 | 0.377 | 0.054 | 2.36E-180 | 6.05E-176 | Ep-C11 |
| TUFM | 0.67 | 0.999 | 0.984 | 2.55E-180 | 6.52E-176 | Ep-C11 |
| MRPL28 | 0.743 | 0.963 | 0.848 | 1.87E-178 | 4.80E-174 | Ep-C11 |
| DCTPP1 | 0.779 | 0.953 | 0.796 | 2.27E-178 | 5.82E-174 | Ep-C11 |
| BOP1 | 0.866 | 0.841 | 0.559 | 7.43E-178 | 1.90E-173 | Ep-C11 |
| NDUFB10 | 0.693 | 0.998 | 0.995 | 8.85E-178 | 2.27E-173 | Ep-C11 |
| HSPD1 | 0.777 | 1 | 0.988 | 2.12E-177 | 5.43E-173 | Ep-C11 |
| SERPINH1 | 1.093 | 0.887 | 0.579 | 3.54E-177 | 9.08E-173 | Ep-C11 |
| KDM5A | 0.937 | 0.829 | 0.619 | 6.27E-177 | 1.61E-172 | Ep-C11 |
| TMEM147 | 0.89 | 0.937 | 0.847 | 2.20E-174 | 5.63E-170 | Ep-C11 |
| UQCR10 | 0.668 | 1 | 0.996 | 1.34E-172 | 3.43E-168 | Ep-C11 |
| LINC02582 | 0.75 | 0.414 | 0.074 | 3.90E-172 | 9.99E-168 | Ep-C11 |
| TOMM40 | 0.776 | 0.951 | 0.828 | 5.92E-171 | 1.52E-166 | Ep-C11 |
| AC092490.1 | 0.744 | 0.416 | 0.076 | 5.12E-170 | 1.31E-165 | Ep-C11 |
| PEG10 | 0.933 | 0.507 | 0.136 | 1.14E-169 | 2.92E-165 | Ep-C11 |
| GAPDH | 0.754 | 1 | 1 | 4.90E-168 | 1.26E-163 | Ep-C11 |
| COX6C | 0.589 | 1 | 1 | 1.01E-166 | 2.60E-162 | Ep-C11 |
| H2AFJ | 1.11 | 0.98 | 0.939 | 1.92E-166 | 4.92E-162 | Ep-C11 |
| PSENEN | 0.853 | 0.798 | 0.612 | 4.89E-166 | 1.25E-161 | Ep-C11 |
| MLF2 | 0.738 | 0.993 | 0.971 | 4.10E-165 | 1.05E-160 | Ep-C11 |
| PHLDA1 | 1.332 | 0.798 | 0.567 | 5.25E-164 | 1.35E-159 | Ep-C11 |
| MRPS35 | 0.787 | 0.896 | 0.727 | 1.06E-162 | 2.72E-158 | Ep-C11 |
| USP5 | 0.844 | 0.72 | 0.412 | 8.07E-162 | 2.07E-157 | Ep-C11 |
| IGFLR1 | 0.824 | 0.523 | 0.169 | 2.07E-161 | 5.30E-157 | Ep-C11 |
| LY6E | 0.805 | 1 | 0.987 | 1.21E-160 | 3.10E-156 | Ep-C11 |
| SNU13 | 0.639 | 0.998 | 0.987 | 4.54E-160 | 1.16E-155 | Ep-C11 |
| C8orf88 | 0.614 | 0.433 | 0.091 | 6.23E-160 | 1.60E-155 | Ep-C11 |
| FHL1 | 0.875 | 0.407 | 0.082 | 2.19E-159 | 5.62E-155 | Ep-C11 |
| CHCHD10 | 0.759 | 0.996 | 0.97 | 1.13E-158 | 2.89E-154 | Ep-C11 |
| DNM1L | 0.822 | 0.851 | 0.628 | 2.01E-157 | 5.14E-153 | Ep-C11 |
| HSPE1 | 0.746 | 1 | 0.994 | 2.56E-157 | 6.56E-153 | Ep-C11 |
| LINC00839 | 0.793 | 0.582 | 0.23 | 3.17E-156 | 8.12E-152 | Ep-C11 |
| TNFRSF6B | 1.038 | 0.569 | 0.197 | 3.55E-155 | 9.09E-151 | Ep-C11 |
| ANO1 | 1.082 | 0.865 | 0.608 | 1.51E-154 | 3.87E-150 | Ep-C11 |
| EEF2KMT | 0.677 | 0.569 | 0.238 | 5.70E-152 | 1.46E-147 | Ep-C11 |
| TRIB2 | 0.841 | 0.552 | 0.219 | 7.68E-151 | 1.97E-146 | Ep-C11 |
| NDUFAF8 | 0.647 | 0.991 | 0.943 | 2.27E-150 | 5.82E-146 | Ep-C11 |
| LTBR | 0.797 | 0.911 | 0.799 | 2.56E-150 | 6.57E-146 | Ep-C11 |
| POLR2E | 0.665 | 0.965 | 0.887 | 4.32E-150 | 1.11E-145 | Ep-C11 |
| ATP5ME | 0.73 | 0.993 | 0.965 | 4.91E-150 | 1.26E-145 | Ep-C11 |
| LSM14A | 0.779 | 0.859 | 0.732 | 7.51E-150 | 1.93E-145 | Ep-C11 |
| DYRK4 | 0.739 | 0.585 | 0.28 | 6.20E-149 | 1.59E-144 | Ep-C11 |
| COL6A1 | 0.999 | 0.571 | 0.194 | 9.05E-149 | 2.32E-144 | Ep-C11 |
| C1QBP | 0.693 | 0.999 | 0.985 | 9.49E-148 | 2.43E-143 | Ep-C11 |
| LPCAT3 | 0.753 | 0.794 | 0.547 | 2.74E-147 | 7.03E-143 | Ep-C11 |
| MT-ND6 | 1.036 | 0.828 | 0.57 | 3.42E-147 | 8.77E-143 | Ep-C11 |
| TEAD4 | 0.69 | 0.574 | 0.227 | 7.01E-147 | 1.80E-142 | Ep-C11 |
| NAP1L1 | 0.64 | 0.999 | 0.98 | 7.76E-146 | 1.99E-141 | Ep-C11 |
| GADD45GIP1 | 0.634 | 0.994 | 0.976 | 2.90E-145 | 7.44E-141 | Ep-C11 |
| TIMM13 | 0.715 | 0.986 | 0.951 | 4.71E-145 | 1.21E-140 | Ep-C11 |
| MRPS34 | 0.671 | 0.997 | 0.972 | 7.86E-145 | 2.01E-140 | Ep-C11 |
| GALNT1 | 0.936 | 0.894 | 0.695 | 8.62E-145 | 2.21E-140 | Ep-C11 |
| EIF4A1 | 0.561 | 1 | 1 | 9.25E-144 | 2.37E-139 | Ep-C11 |
| EXOSC5 | 0.761 | 0.831 | 0.601 | 1.12E-143 | 2.87E-139 | Ep-C11 |
| CAPNS1 | 0.774 | 0.954 | 0.919 | 1.05E-142 | 2.68E-138 | Ep-C11 |
| PRMT1 | 0.657 | 0.991 | 0.955 | 2.78E-141 | 7.13E-137 | Ep-C11 |
| IGHMBP2 | 0.917 | 0.639 | 0.312 | 6.26E-141 | 1.60E-136 | Ep-C11 |
| ETV5 | 0.701 | 0.622 | 0.241 | 3.44E-140 | 8.81E-136 | Ep-C11 |
| SMIM10L1 | 0.72 | 0.835 | 0.548 | 1.23E-139 | 3.14E-135 | Ep-C11 |
| TPCN2 | 0.774 | 0.549 | 0.213 | 1.87E-139 | 4.79E-135 | Ep-C11 |
| LAPTM4B | 0.696 | 0.993 | 0.95 | 8.01E-139 | 2.05E-134 | Ep-C11 |
| MAGOHB | 0.733 | 0.859 | 0.597 | 4.37E-138 | 1.12E-133 | Ep-C11 |
| GCSH | 0.709 | 0.945 | 0.821 | 1.18E-137 | 3.03E-133 | Ep-C11 |
| RANBP1 | 0.659 | 0.996 | 0.983 | 3.08E-137 | 7.89E-133 | Ep-C11 |
| PXDN | 0.766 | 0.476 | 0.137 | 2.04E-136 | 5.24E-132 | Ep-C11 |
| SLIRP | 0.712 | 0.989 | 0.94 | 9.70E-136 | 2.49E-131 | Ep-C11 |
| FGF4 | 0.569 | 0.261 | 0.031 | 3.51E-135 | 8.99E-131 | Ep-C11 |
| PUF60 | 0.611 | 0.992 | 0.957 | 9.86E-135 | 2.53E-130 | Ep-C11 |
| LTO1 | 0.816 | 0.824 | 0.509 | 2.22E-134 | 5.69E-130 | Ep-C11 |
| MAEL | 0.51 | 0.321 | 0.054 | 2.68E-134 | 6.86E-130 | Ep-C11 |
| ARHGAP5-AS1 | 0.682 | 0.503 | 0.2 | 1.11E-133 | 2.84E-129 | Ep-C11 |
| VIM | 1.069 | 0.803 | 0.505 | 1.82E-133 | 4.67E-129 | Ep-C11 |
| CCDC88A | 0.786 | 0.683 | 0.327 | 5.66E-133 | 1.45E-128 | Ep-C11 |
| PARP1 | 0.687 | 0.934 | 0.791 | 1.13E-132 | 2.90E-128 | Ep-C11 |
| CLDN10 | 1.038 | 0.598 | 0.236 | 1.68E-132 | 4.31E-128 | Ep-C11 |
| MRPL52 | 0.622 | 0.986 | 0.904 | 2.13E-132 | 5.46E-128 | Ep-C11 |
| SERPINE2 | 0.983 | 0.85 | 0.567 | 1.50E-131 | 3.84E-127 | Ep-C11 |
| BCAT1 | 0.72 | 0.478 | 0.144 | 1.91E-131 | 4.89E-127 | Ep-C11 |
| AHSA1 | 0.717 | 0.92 | 0.766 | 1.37E-130 | 3.50E-126 | Ep-C11 |
| GNG4 | 0.734 | 0.429 | 0.12 | 3.96E-130 | 1.01E-125 | Ep-C11 |
| COL6A2 | 0.888 | 0.526 | 0.174 | 5.32E-130 | 1.36E-125 | Ep-C11 |
| PLEKHA5 | 0.815 | 0.741 | 0.54 | 2.85E-129 | 7.31E-125 | Ep-C11 |
| UQCRC1 | 0.584 | 0.985 | 0.922 | 3.06E-129 | 7.85E-125 | Ep-C11 |
| PSMB6 | 0.6 | 0.998 | 0.987 | 4.86E-129 | 1.25E-124 | Ep-C11 |
| RPS16 | 0.555 | 1 | 1 | 8.95E-129 | 2.29E-124 | Ep-C11 |
| FGFR1OP2 | 0.697 | 0.853 | 0.641 | 1.53E-127 | 3.93E-123 | Ep-C11 |
| ZNF22 | 0.763 | 0.721 | 0.471 | 1.87E-127 | 4.79E-123 | Ep-C11 |
| ZNF146 | 0.728 | 0.669 | 0.411 | 3.38E-127 | 8.66E-123 | Ep-C11 |
| HNRNPF | 0.624 | 0.998 | 0.992 | 9.54E-127 | 2.44E-122 | Ep-C11 |
| SARS2 | 0.667 | 0.611 | 0.292 | 2.54E-126 | 6.52E-122 | Ep-C11 |
| BMS1 | 0.692 | 0.725 | 0.467 | 9.30E-125 | 2.38E-120 | Ep-C11 |
| GINS2 | 0.802 | 0.671 | 0.326 | 1.40E-124 | 3.58E-120 | Ep-C11 |
| LGALS1 | 0.804 | 0.89 | 0.6 | 1.62E-124 | 4.15E-120 | Ep-C11 |
| BORCS5 | 0.702 | 0.656 | 0.359 | 2.05E-124 | 5.26E-120 | Ep-C11 |
| METTL26 | 0.606 | 0.991 | 0.939 | 3.92E-124 | 1.00E-119 | Ep-C11 |
| ATP5F1B | 0.532 | 0.999 | 0.998 | 4.63E-124 | 1.19E-119 | Ep-C11 |
| NDUFA6 | 0.571 | 0.988 | 0.948 | 4.72E-124 | 1.21E-119 | Ep-C11 |
| LTBP4 | 0.858 | 0.746 | 0.482 | 5.27E-124 | 1.35E-119 | Ep-C11 |
| SDHAF1 | 0.668 | 0.649 | 0.404 | 2.99E-123 | 7.67E-119 | Ep-C11 |
| RIMKLB | 0.674 | 0.491 | 0.197 | 3.04E-123 | 7.80E-119 | Ep-C11 |
| COX7B | 0.564 | 1 | 0.996 | 5.17E-123 | 1.33E-118 | Ep-C11 |
| NFKBID | 0.869 | 0.533 | 0.237 | 1.33E-122 | 3.41E-118 | Ep-C11 |
| FH | 0.643 | 0.823 | 0.596 | 1.43E-122 | 3.65E-118 | Ep-C11 |
| PTX3 | 0.606 | 0.271 | 0.04 | 2.98E-122 | 7.65E-118 | Ep-C11 |
| LSM4 | 0.594 | 0.994 | 0.946 | 3.76E-122 | 9.65E-118 | Ep-C11 |
| POLR2L | 0.567 | 0.999 | 0.982 | 4.28E-121 | 1.10E-116 | Ep-C11 |
| HNRNPA2B1 | 0.552 | 1 | 0.998 | 2.23E-120 | 5.72E-116 | Ep-C11 |
| DKK1 | 1.327 | 0.547 | 0.215 | 6.23E-120 | 1.60E-115 | Ep-C11 |
| PDCD2L | 0.627 | 0.55 | 0.243 | 7.07E-120 | 1.81E-115 | Ep-C11 |
| CTNNAL1 | 0.689 | 0.686 | 0.316 | 1.19E-119 | 3.04E-115 | Ep-C11 |
| GSDMD | 0.623 | 0.902 | 0.739 | 1.87E-118 | 4.79E-114 | Ep-C11 |
| BHLHE41 | 0.745 | 0.553 | 0.219 | 2.11E-118 | 5.41E-114 | Ep-C11 |
| LRP6 | 0.614 | 0.569 | 0.247 | 2.51E-118 | 6.43E-114 | Ep-C11 |
| NDUFB7 | 0.522 | 1 | 0.989 | 3.15E-118 | 8.08E-114 | Ep-C11 |
| H2AFY2 | 0.642 | 0.724 | 0.431 | 3.77E-118 | 9.67E-114 | Ep-C11 |
| CAVIN3 | 0.765 | 0.764 | 0.411 | 1.84E-117 | 4.72E-113 | Ep-C11 |
| PGM2L1 | 0.864 | 0.582 | 0.277 | 2.85E-117 | 7.31E-113 | Ep-C11 |
| ETV4 | 0.586 | 0.6 | 0.249 | 6.37E-117 | 1.63E-112 | Ep-C11 |
| HMGB1 | 0.604 | 1 | 0.999 | 1.08E-116 | 2.78E-112 | Ep-C11 |
| SAMM50 | 0.592 | 0.811 | 0.598 | 3.01E-116 | 7.72E-112 | Ep-C11 |
| NPTX2 | 0.5 | 0.291 | 0.051 | 3.36E-116 | 8.60E-112 | Ep-C11 |
| PSMC4 | 0.671 | 0.955 | 0.885 | 5.26E-116 | 1.35E-111 | Ep-C11 |
| MRPS7 | 0.525 | 0.989 | 0.938 | 1.89E-115 | 4.84E-111 | Ep-C11 |
| LRRCC1 | 0.677 | 0.644 | 0.326 | 9.29E-115 | 2.38E-110 | Ep-C11 |
| CCT7 | 0.614 | 0.968 | 0.914 | 1.13E-114 | 2.90E-110 | Ep-C11 |
| PAQR4 | 0.653 | 0.567 | 0.259 | 1.21E-114 | 3.10E-110 | Ep-C11 |
| RPS19BP1 | 0.537 | 0.986 | 0.946 | 1.41E-114 | 3.62E-110 | Ep-C11 |
| MAZ | 0.581 | 0.956 | 0.853 | 2.01E-114 | 5.15E-110 | Ep-C11 |
| NAA38 | 0.575 | 0.982 | 0.934 | 7.57E-114 | 1.94E-109 | Ep-C11 |
| CAPN2 | 0.679 | 0.911 | 0.795 | 9.73E-114 | 2.50E-109 | Ep-C11 |
| ATP5MPL | 0.562 | 0.999 | 0.992 | 1.07E-112 | 2.75E-108 | Ep-C11 |
| SSPN | 0.685 | 0.596 | 0.266 | 1.60E-112 | 4.10E-108 | Ep-C11 |
| NEDD8 | 0.523 | 0.998 | 0.993 | 3.28E-112 | 8.41E-108 | Ep-C11 |
| SNRPD1 | 0.585 | 1 | 0.978 | 3.30E-112 | 8.45E-108 | Ep-C11 |
| TYSND1 | 0.635 | 0.664 | 0.381 | 3.58E-112 | 9.18E-108 | Ep-C11 |
| ADSL | 0.586 | 0.839 | 0.598 | 4.09E-112 | 1.05E-107 | Ep-C11 |
| CENPM | 0.678 | 0.7 | 0.343 | 6.86E-112 | 1.76E-107 | Ep-C11 |
| GPAA1 | 0.587 | 0.928 | 0.771 | 1.17E-111 | 3.00E-107 | Ep-C11 |
| MT-ND3 | 0.714 | 0.999 | 1 | 2.88E-111 | 7.37E-107 | Ep-C11 |
| ADIPOR2 | 0.639 | 0.73 | 0.449 | 5.91E-111 | 1.52E-106 | Ep-C11 |
| MAF1 | 0.59 | 0.935 | 0.866 | 7.33E-111 | 1.88E-106 | Ep-C11 |
| HNRNPU | 0.601 | 0.999 | 0.984 | 9.24E-111 | 2.37E-106 | Ep-C11 |
| TSTA3 | 0.59 | 0.951 | 0.832 | 2.05E-110 | 5.25E-106 | Ep-C11 |
| CHCHD7 | 0.599 | 0.897 | 0.739 | 1.47E-109 | 3.76E-105 | Ep-C11 |
| MCM4 | 0.722 | 0.762 | 0.482 | 2.76E-109 | 7.07E-105 | Ep-C11 |
| NUBP2 | 0.563 | 0.895 | 0.734 | 3.71E-109 | 9.51E-105 | Ep-C11 |
| TMEM88 | 0.57 | 0.341 | 0.099 | 6.98E-109 | 1.79E-104 | Ep-C11 |
| IFITM3 | 0.688 | 0.997 | 0.98 | 2.43E-108 | 6.22E-104 | Ep-C11 |
| CDT1 | 0.687 | 0.602 | 0.285 | 1.59E-107 | 4.08E-103 | Ep-C11 |
| ILF3-DT | 0.672 | 0.661 | 0.392 | 2.10E-107 | 5.38E-103 | Ep-C11 |
| YARS2 | 0.607 | 0.731 | 0.447 | 2.69E-107 | 6.89E-103 | Ep-C11 |
| YIF1B | 0.619 | 0.713 | 0.44 | 2.74E-107 | 7.02E-103 | Ep-C11 |
| PGP | 0.679 | 0.853 | 0.628 | 3.12E-107 | 7.99E-103 | Ep-C11 |
| TIMM23 | 0.608 | 0.641 | 0.39 | 2.30E-106 | 5.90E-102 | Ep-C11 |
| ERGIC2 | 0.619 | 0.908 | 0.788 | 4.82E-106 | 1.24E-101 | Ep-C11 |
| C19orf54 | 0.525 | 0.421 | 0.144 | 6.00E-106 | 1.54E-101 | Ep-C11 |
| NOVA1 | 0.509 | 0.329 | 0.084 | 8.02E-106 | 2.06E-101 | Ep-C11 |
| LINC00392 | 0.757 | 0.469 | 0.185 | 1.23E-105 | 3.15E-101 | Ep-C11 |
| ARF1 | 0.56 | 0.995 | 0.982 | 1.80E-105 | 4.60E-101 | Ep-C11 |
| SLC2A4RG | 0.578 | 0.947 | 0.8 | 1.92E-105 | 4.92E-101 | Ep-C11 |
| RTCB | 0.584 | 0.869 | 0.723 | 2.15E-105 | 5.52E-101 | Ep-C11 |
| TNFRSF1A | 0.647 | 0.924 | 0.849 | 2.38E-105 | 6.09E-101 | Ep-C11 |
| MRPL38 | 0.546 | 0.924 | 0.809 | 2.49E-105 | 6.38E-101 | Ep-C11 |
| RECQL | 0.673 | 0.703 | 0.426 | 4.38E-105 | 1.12E-100 | Ep-C11 |
| NCOA4 | 0.641 | 0.837 | 0.667 | 9.27E-105 | 2.38E-100 | Ep-C11 |
| DNMT1 | 0.677 | 0.83 | 0.569 | 2.40E-104 | 6.15E-100 | Ep-C11 |
| PYCR3 | 0.582 | 0.605 | 0.302 | 4.04E-104 | 1.04E-99 | Ep-C11 |
| JMJD8 | 0.601 | 0.759 | 0.519 | 4.20E-104 | 1.08E-99 | Ep-C11 |
| ZNF585A | 0.567 | 0.456 | 0.19 | 7.73E-104 | 1.98E-99 | Ep-C11 |
| SUPT16H | 0.614 | 0.871 | 0.667 | 1.86E-103 | 4.76E-99 | Ep-C11 |
| MAGEA3 | 0.687 | 0.658 | 0.386 | 2.90E-103 | 7.42E-99 | Ep-C11 |
| MAGEA6 | 0.662 | 0.703 | 0.4 | 3.00E-103 | 7.69E-99 | Ep-C11 |
| HSPA6 | 1.185 | 0.527 | 0.214 | 1.11E-102 | 2.85E-98 | Ep-C11 |
| SLC25A11 | 0.543 | 0.918 | 0.768 | 1.30E-102 | 3.34E-98 | Ep-C11 |
| PRDX4 | 0.561 | 0.977 | 0.906 | 1.58E-102 | 4.05E-98 | Ep-C11 |
| CISD1 | 0.617 | 0.898 | 0.785 | 1.75E-101 | 4.49E-97 | Ep-C11 |
| USF2 | 0.622 | 0.797 | 0.603 | 3.80E-101 | 9.73E-97 | Ep-C11 |
| C19orf53 | 0.504 | 0.998 | 0.989 | 5.16E-100 | 1.32E-95 | Ep-C11 |
| NASP | 0.555 | 0.984 | 0.869 | 6.29E-100 | 1.61E-95 | Ep-C11 |
| MRPL4 | 0.501 | 0.978 | 0.894 | 1.16E-99 | 2.97E-95 | Ep-C11 |
| EGLN2 | 0.645 | 0.712 | 0.486 | 5.34E-99 | 1.37E-94 | Ep-C11 |
| SSRP1 | 0.542 | 0.906 | 0.742 | 1.45E-98 | 3.72E-94 | Ep-C11 |
| ITPRID2 | 0.755 | 0.831 | 0.679 | 1.55E-98 | 3.98E-94 | Ep-C11 |
| PTGES2 | 0.543 | 0.893 | 0.719 | 1.66E-98 | 4.25E-94 | Ep-C11 |
| SLC12A2 | 0.702 | 0.579 | 0.286 | 3.39E-98 | 8.70E-94 | Ep-C11 |
| PSME2 | 0.534 | 0.994 | 0.953 | 1.11E-97 | 2.86E-93 | Ep-C11 |
| SNRPA | 0.558 | 0.86 | 0.632 | 1.16E-97 | 2.97E-93 | Ep-C11 |
| PAICS | 0.545 | 0.888 | 0.711 | 2.55E-97 | 6.53E-93 | Ep-C11 |
| DNAJC15 | 0.591 | 0.869 | 0.711 | 3.26E-97 | 8.36E-93 | Ep-C11 |
| TK1 | 0.563 | 0.854 | 0.542 | 3.60E-97 | 9.22E-93 | Ep-C11 |
| RNF187 | 0.567 | 0.919 | 0.78 | 7.33E-97 | 1.88E-92 | Ep-C11 |
| TULP3 | 0.579 | 0.541 | 0.285 | 1.55E-96 | 3.97E-92 | Ep-C11 |
| STUB1 | 0.532 | 0.979 | 0.956 | 4.04E-96 | 1.04E-91 | Ep-C11 |
| ERH | 0.503 | 0.996 | 0.981 | 5.98E-96 | 1.53E-91 | Ep-C11 |
| SNRNP25 | 0.557 | 0.864 | 0.639 | 6.35E-96 | 1.63E-91 | Ep-C11 |
| HEY1 | 0.734 | 0.686 | 0.368 | 2.65E-95 | 6.79E-91 | Ep-C11 |
| KRR1 | 0.599 | 0.898 | 0.8 | 3.29E-95 | 8.44E-91 | Ep-C11 |
| RALY | 0.517 | 0.965 | 0.914 | 5.26E-95 | 1.35E-90 | Ep-C11 |
| COQ8B | 0.548 | 0.546 | 0.289 | 5.57E-95 | 1.43E-90 | Ep-C11 |
| CKAP4 | 0.634 | 0.844 | 0.681 | 1.48E-94 | 3.79E-90 | Ep-C11 |
| ETNK1 | 0.621 | 0.784 | 0.6 | 1.63E-94 | 4.18E-90 | Ep-C11 |
| MPDU1 | 0.551 | 0.731 | 0.452 | 2.83E-94 | 7.26E-90 | Ep-C11 |
| FOXM1 | 0.55 | 0.568 | 0.246 | 3.13E-94 | 8.02E-90 | Ep-C11 |
| PSME1 | 0.509 | 0.996 | 0.972 | 3.35E-94 | 8.58E-90 | Ep-C11 |
| RPN2 | 0.516 | 0.992 | 0.968 | 6.65E-94 | 1.70E-89 | Ep-C11 |
| VPS26A | 0.548 | 0.892 | 0.794 | 8.56E-94 | 2.19E-89 | Ep-C11 |
| CSTF2T | 0.524 | 0.501 | 0.241 | 1.03E-93 | 2.64E-89 | Ep-C11 |
| ECH1 | 0.532 | 0.978 | 0.929 | 2.70E-93 | 6.91E-89 | Ep-C11 |
| RHNO1 | 0.584 | 0.625 | 0.326 | 3.21E-93 | 8.24E-89 | Ep-C11 |
| ATOX1 | 0.549 | 0.916 | 0.785 | 1.29E-92 | 3.30E-88 | Ep-C11 |
| ARHGDIB | 0.605 | 0.439 | 0.184 | 1.88E-92 | 4.83E-88 | Ep-C11 |
| PSMB5 | 0.509 | 0.993 | 0.967 | 2.95E-92 | 7.55E-88 | Ep-C11 |
| TSR3 | 0.507 | 0.879 | 0.758 | 3.19E-92 | 8.16E-88 | Ep-C11 |
| MCM7 | 0.644 | 0.832 | 0.589 | 3.31E-92 | 8.49E-88 | Ep-C11 |
| FXYD5 | 0.734 | 0.804 | 0.595 | 4.69E-92 | 1.20E-87 | Ep-C11 |
| TRAP1 | 0.541 | 0.869 | 0.657 | 6.35E-92 | 1.63E-87 | Ep-C11 |
| MT-ND2 | 0.628 | 1 | 1 | 8.14E-92 | 2.09E-87 | Ep-C11 |
| NPW | 0.667 | 0.81 | 0.514 | 1.47E-91 | 3.76E-87 | Ep-C11 |
| IARS2 | 0.56 | 0.818 | 0.591 | 1.60E-91 | 4.09E-87 | Ep-C11 |
| POLR3K | 0.578 | 0.758 | 0.506 | 3.13E-91 | 8.02E-87 | Ep-C11 |
| EID2 | 0.554 | 0.631 | 0.378 | 1.85E-90 | 4.74E-86 | Ep-C11 |
| GPS1 | 0.551 | 0.797 | 0.574 | 2.06E-90 | 5.29E-86 | Ep-C11 |
| MAP9 | 0.542 | 0.508 | 0.225 | 2.26E-90 | 5.79E-86 | Ep-C11 |
| HSP90B1 | 0.526 | 0.997 | 0.992 | 2.82E-90 | 7.22E-86 | Ep-C11 |
| SPSB2 | 0.531 | 0.536 | 0.255 | 3.27E-90 | 8.39E-86 | Ep-C11 |
| RAB30-DT | 0.601 | 0.677 | 0.446 | 6.92E-90 | 1.77E-85 | Ep-C11 |
| PDHA1 | 0.508 | 0.877 | 0.703 | 9.46E-90 | 2.43E-85 | Ep-C11 |
| SPR | 0.548 | 0.631 | 0.396 | 2.29E-89 | 5.87E-85 | Ep-C11 |
| HMGA2 | 0.519 | 0.471 | 0.183 | 3.39E-89 | 8.70E-85 | Ep-C11 |
| ACSL4 | 0.67 | 0.562 | 0.313 | 3.47E-89 | 8.89E-85 | Ep-C11 |
| FADD | 0.631 | 0.875 | 0.67 | 8.15E-89 | 2.09E-84 | Ep-C11 |
| PRMT5 | 0.517 | 0.607 | 0.357 | 1.22E-88 | 3.12E-84 | Ep-C11 |
| APEX1 | 0.525 | 0.968 | 0.895 | 1.28E-88 | 3.28E-84 | Ep-C11 |
| COL4A2 | 0.535 | 0.666 | 0.343 | 1.66E-88 | 4.25E-84 | Ep-C11 |
| AC092821.3 | 0.522 | 0.486 | 0.217 | 1.96E-88 | 5.03E-84 | Ep-C11 |
| KRT8 | 0.877 | 0.918 | 0.848 | 2.57E-88 | 6.58E-84 | Ep-C11 |
| GTF2E2 | 0.538 | 0.766 | 0.53 | 1.11E-87 | 2.84E-83 | Ep-C11 |
| NOP2 | 0.526 | 0.554 | 0.279 | 1.54E-87 | 3.94E-83 | Ep-C11 |
| FKBP11 | 0.559 | 0.723 | 0.455 | 2.74E-87 | 7.01E-83 | Ep-C11 |
| DRG1 | 0.515 | 0.757 | 0.521 | 4.24E-87 | 1.09E-82 | Ep-C11 |
| U2AF1L4 | 0.514 | 0.497 | 0.221 | 7.12E-87 | 1.83E-82 | Ep-C11 |
| CYB5R3 | 0.521 | 0.933 | 0.809 | 2.14E-86 | 5.48E-82 | Ep-C11 |
| RASSF8 | 0.517 | 0.456 | 0.178 | 3.03E-86 | 7.76E-82 | Ep-C11 |
| AGR2 | 1.022 | 0.545 | 0.288 | 3.95E-86 | 1.01E-81 | Ep-C11 |
| RAD51AP1 | 0.689 | 0.553 | 0.259 | 9.41E-86 | 2.41E-81 | Ep-C11 |
| MTHFD1 | 0.524 | 0.613 | 0.327 | 1.49E-85 | 3.82E-81 | Ep-C11 |
| LMNB2 | 0.581 | 0.726 | 0.443 | 9.11E-85 | 2.33E-80 | Ep-C11 |
| ARL4C | 0.765 | 0.794 | 0.593 | 3.58E-84 | 9.16E-80 | Ep-C11 |
| UQCC3 | 0.534 | 0.917 | 0.8 | 4.40E-84 | 1.13E-79 | Ep-C11 |
| METRN | 0.557 | 0.835 | 0.64 | 4.62E-84 | 1.18E-79 | Ep-C11 |
| NR2F2 | 0.521 | 0.446 | 0.183 | 1.44E-83 | 3.70E-79 | Ep-C11 |
| HAUS5 | 0.504 | 0.481 | 0.21 | 2.70E-83 | 6.92E-79 | Ep-C11 |
| NFKBIB | 0.551 | 0.697 | 0.482 | 3.37E-83 | 8.63E-79 | Ep-C11 |
| C1orf35 | 0.531 | 0.858 | 0.672 | 5.73E-83 | 1.47E-78 | Ep-C11 |
| C20orf27 | 0.515 | 0.877 | 0.685 | 6.67E-83 | 1.71E-78 | Ep-C11 |
| RUVBL2 | 0.501 | 0.858 | 0.649 | 4.84E-82 | 1.24E-77 | Ep-C11 |
| RCN1 | 0.508 | 0.883 | 0.722 | 8.39E-82 | 2.15E-77 | Ep-C11 |
| MCM3 | 0.6 | 0.741 | 0.458 | 1.26E-81 | 3.22E-77 | Ep-C11 |
| FXYD3 | 0.747 | 1 | 1 | 2.17E-81 | 5.56E-77 | Ep-C11 |
| LAS1L | 0.516 | 0.58 | 0.334 | 2.35E-81 | 6.03E-77 | Ep-C11 |
| CNN2 | 0.509 | 0.921 | 0.767 | 2.46E-81 | 6.30E-77 | Ep-C11 |
| IMPAD1 | 0.519 | 0.817 | 0.62 | 2.87E-81 | 7.37E-77 | Ep-C11 |
| CCN3 | 0.563 | 0.445 | 0.174 | 5.34E-81 | 1.37E-76 | Ep-C11 |
| NCL | 0.539 | 0.996 | 0.979 | 1.14E-80 | 2.91E-76 | Ep-C11 |
| DDX49 | 0.513 | 0.782 | 0.574 | 1.25E-80 | 3.19E-76 | Ep-C11 |
| TSPAN9 | 0.547 | 0.582 | 0.344 | 2.23E-80 | 5.71E-76 | Ep-C11 |
| CD320 | 0.551 | 0.792 | 0.545 | 2.39E-79 | 6.12E-75 | Ep-C11 |
| PEPD | 0.575 | 0.778 | 0.585 | 3.38E-79 | 8.67E-75 | Ep-C11 |
| PRPS2 | 0.509 | 0.696 | 0.454 | 3.46E-79 | 8.88E-75 | Ep-C11 |
| STC1 | 0.591 | 0.216 | 0.041 | 4.06E-79 | 1.04E-74 | Ep-C11 |
| FAM217B | 0.607 | 0.592 | 0.369 | 9.17E-79 | 2.35E-74 | Ep-C11 |
| DTYMK | 0.518 | 0.845 | 0.593 | 1.33E-78 | 3.40E-74 | Ep-C11 |
| NFIB | 0.603 | 0.689 | 0.419 | 1.45E-78 | 3.72E-74 | Ep-C11 |
| SAR1A | 0.52 | 0.907 | 0.801 | 2.36E-78 | 6.05E-74 | Ep-C11 |
| CMAS | 0.502 | 0.842 | 0.721 | 6.77E-78 | 1.73E-73 | Ep-C11 |
| TFAM | 0.514 | 0.771 | 0.541 | 8.03E-78 | 2.06E-73 | Ep-C11 |
| DPYSL3 | 0.594 | 0.574 | 0.299 | 1.09E-77 | 2.80E-73 | Ep-C11 |
| PRSS21 | 0.601 | 0.752 | 0.485 | 1.71E-77 | 4.38E-73 | Ep-C11 |
| CXCL1 | 0.804 | 0.624 | 0.337 | 2.21E-77 | 5.66E-73 | Ep-C11 |
| IFITM1 | 0.616 | 0.753 | 0.484 | 2.93E-76 | 7.51E-72 | Ep-C11 |
| KRT7 | 0.571 | 0.515 | 0.235 | 7.20E-76 | 1.85E-71 | Ep-C11 |
| KMT2B | 0.533 | 0.612 | 0.383 | 1.14E-75 | 2.91E-71 | Ep-C11 |
| ATN1 | 0.554 | 0.702 | 0.465 | 1.84E-75 | 4.70E-71 | Ep-C11 |
| PLRG1 | 0.58 | 0.709 | 0.553 | 4.70E-75 | 1.21E-70 | Ep-C11 |
| MT-ND4L | 0.542 | 0.985 | 0.96 | 2.04E-74 | 5.23E-70 | Ep-C11 |
| DDX21 | 0.523 | 0.971 | 0.914 | 4.73E-74 | 1.21E-69 | Ep-C11 |
| KNOP1 | 0.503 | 0.715 | 0.498 | 1.39E-73 | 3.57E-69 | Ep-C11 |
| ALOX15 | 0.574 | 0.238 | 0.058 | 1.41E-73 | 3.60E-69 | Ep-C11 |
| ISYNA1 | 0.543 | 0.553 | 0.293 | 2.53E-73 | 6.48E-69 | Ep-C11 |
| MT-ND4 | 0.522 | 1 | 1 | 4.12E-73 | 1.06E-68 | Ep-C11 |
| HS3ST1 | 0.581 | 0.328 | 0.13 | 4.65E-73 | 1.19E-68 | Ep-C11 |
| ZC3H13 | 0.519 | 0.777 | 0.623 | 5.97E-73 | 1.53E-68 | Ep-C11 |
| NME4 | 0.531 | 0.873 | 0.735 | 7.11E-73 | 1.82E-68 | Ep-C11 |
| TXNDC17 | 0.515 | 1 | 0.995 | 9.02E-72 | 2.31E-67 | Ep-C11 |
| WNK1 | 0.558 | 0.87 | 0.744 | 1.16E-71 | 2.97E-67 | Ep-C11 |
| MAGEA1 | 0.515 | 0.53 | 0.273 | 4.30E-71 | 1.10E-66 | Ep-C11 |
| MCM6 | 0.511 | 0.559 | 0.304 | 1.41E-70 | 3.61E-66 | Ep-C11 |
| GOLT1B | 0.52 | 0.728 | 0.48 | 2.57E-70 | 6.59E-66 | Ep-C11 |
| ZWINT | 0.588 | 0.673 | 0.391 | 7.86E-70 | 2.01E-65 | Ep-C11 |
| CDC45 | 0.506 | 0.467 | 0.208 | 1.70E-69 | 4.36E-65 | Ep-C11 |
| PRKDC | 0.503 | 0.971 | 0.912 | 1.99E-69 | 5.10E-65 | Ep-C11 |
| COL3A1 | 0.543 | 0.443 | 0.189 | 2.01E-69 | 5.15E-65 | Ep-C11 |
| MSX1 | 0.512 | 0.465 | 0.212 | 1.34E-68 | 3.45E-64 | Ep-C11 |
| DUSP6 | 0.647 | 0.66 | 0.4 | 3.99E-68 | 1.02E-63 | Ep-C11 |
| SIGIRR | 0.557 | 0.796 | 0.583 | 4.93E-68 | 1.26E-63 | Ep-C11 |
| JMJD1C | 0.516 | 0.726 | 0.533 | 1.56E-67 | 4.01E-63 | Ep-C11 |
| PLBD1 | 0.537 | 0.624 | 0.455 | 1.77E-67 | 4.53E-63 | Ep-C11 |
| CXCL3 | 0.665 | 0.482 | 0.225 | 9.26E-67 | 2.37E-62 | Ep-C11 |
| MT-ND1 | 0.506 | 1 | 1 | 1.37E-66 | 3.50E-62 | Ep-C11 |
| HAS2 | 0.535 | 0.24 | 0.064 | 5.43E-66 | 1.39E-61 | Ep-C11 |
| MT-ATP6 | 0.527 | 1 | 1 | 5.81E-66 | 1.49E-61 | Ep-C11 |
| TPBG | 0.656 | 0.825 | 0.679 | 9.07E-66 | 2.33E-61 | Ep-C11 |
| BCKDHA | 0.512 | 0.704 | 0.502 | 1.25E-65 | 3.22E-61 | Ep-C11 |
| NAPRT | 0.527 | 0.911 | 0.799 | 3.16E-63 | 8.11E-59 | Ep-C11 |
| GPI | 0.516 | 0.951 | 0.869 | 6.08E-63 | 1.56E-58 | Ep-C11 |
| PAK1 | 0.549 | 0.771 | 0.546 | 3.65E-62 | 9.36E-58 | Ep-C11 |
| KLK10 | 0.836 | 0.626 | 0.452 | 1.06E-61 | 2.72E-57 | Ep-C11 |
| HSPH1 | 0.529 | 0.939 | 0.852 | 1.37E-61 | 3.50E-57 | Ep-C11 |
| UNG | 0.507 | 0.578 | 0.365 | 4.64E-61 | 1.19E-56 | Ep-C11 |
| CEBPG | 0.512 | 0.783 | 0.654 | 1.49E-60 | 3.83E-56 | Ep-C11 |
| IGFBP4 | 0.556 | 0.857 | 0.695 | 1.72E-60 | 4.40E-56 | Ep-C11 |
| GLIPR1 | 0.659 | 0.453 | 0.242 | 6.59E-60 | 1.69E-55 | Ep-C11 |
| PTHLH | 0.522 | 0.832 | 0.603 | 2.49E-58 | 6.37E-54 | Ep-C11 |
| CLEC2B | 0.518 | 0.794 | 0.555 | 9.60E-58 | 2.46E-53 | Ep-C11 |
| HIST1H1C | 0.853 | 0.777 | 0.668 | 4.02E-57 | 1.03E-52 | Ep-C11 |
| TMSB4X | 0.561 | 1 | 0.999 | 1.06E-56 | 2.71E-52 | Ep-C11 |
| EPS8 | 0.531 | 0.6 | 0.369 | 1.49E-54 | 3.83E-50 | Ep-C11 |
| FOXP1 | 0.525 | 0.631 | 0.43 | 2.94E-51 | 7.54E-47 | Ep-C11 |
| ODC1 | 0.536 | 0.93 | 0.825 | 1.44E-47 | 3.70E-43 | Ep-C11 |
| ARID5B | 0.529 | 0.904 | 0.819 | 1.64E-43 | 4.21E-39 | Ep-C11 |
| KRT6B | 0.51 | 0.852 | 0.803 | 1.66E-35 | 4.25E-31 | Ep-C11 |
| ZNF322 | 3.036 | 0.816 | 0.331 | 6.15249138609773e-316 | 1.57694506717071e-311 | Ep-C12 |
| SS18 | 1.76 | 0.949 | 0.524 | 4.75E-281 | 1.22E-276 | Ep-C12 |
| AOAH | 1.978 | 0.812 | 0.055 | 9.67E-232 | 2.48E-227 | Ep-C12 |
| RBBP8 | 1.732 | 0.996 | 0.624 | 9.76E-209 | 2.50E-204 | Ep-C12 |
| KCTD1 | 1.468 | 0.897 | 0.428 | 7.82E-189 | 2.00E-184 | Ep-C12 |
| NUDT14 | 1.757 | 0.996 | 0.769 | 2.45E-183 | 6.28E-179 | Ep-C12 |
| HEY1 | 2.458 | 0.88 | 0.379 | 2.67E-183 | 6.84E-179 | Ep-C12 |
| TAF4B | 1.442 | 0.799 | 0.214 | 7.14E-183 | 1.83E-178 | Ep-C12 |
| CAMK1G | 2.272 | 0.983 | 0.2 | 1.81E-167 | 4.64E-163 | Ep-C12 |
| SIVA1 | 1.826 | 1 | 0.963 | 1.07E-142 | 2.75E-138 | Ep-C12 |
| DNAJA1 | 1.791 | 1 | 0.945 | 4.90E-142 | 1.26E-137 | Ep-C12 |
| MAGEA4 | 1.946 | 0.949 | 0.276 | 5.64E-139 | 1.44E-134 | Ep-C12 |
| PAIP1 | 1.645 | 0.996 | 0.852 | 1.06E-138 | 2.72E-134 | Ep-C12 |
| CTAG2 | 1.67 | 0.949 | 0.207 | 4.20E-135 | 1.08E-130 | Ep-C12 |
| SGK1 | 1.722 | 0.996 | 0.894 | 7.63E-134 | 1.95E-129 | Ep-C12 |
| ZNF131 | 1.579 | 0.996 | 0.72 | 5.39E-133 | 1.38E-128 | Ep-C12 |
| UBAP2 | 1.431 | 0.953 | 0.567 | 2.75E-129 | 7.04E-125 | Ep-C12 |
| PLAT | 1.927 | 0.979 | 0.613 | 9.99E-128 | 2.56E-123 | Ep-C12 |
| CRABP1 | 1.627 | 0.868 | 0.162 | 7.01E-126 | 1.80E-121 | Ep-C12 |
| HMGCS1 | 1.915 | 1 | 0.869 | 2.08E-122 | 5.33E-118 | Ep-C12 |
| SNHG15 | 1.261 | 0.97 | 0.703 | 1.60E-116 | 4.10E-112 | Ep-C12 |
| ADSSL1 | 1.403 | 0.868 | 0.318 | 9.44E-116 | 2.42E-111 | Ep-C12 |
| C1QTNF12 | 1.442 | 0.974 | 0.58 | 2.48E-114 | 6.36E-110 | Ep-C12 |
| TFF2 | 1.287 | 0.791 | 0.146 | 2.27E-113 | 5.81E-109 | Ep-C12 |
| DUSP2 | 2.084 | 0.803 | 0.425 | 1.07E-111 | 2.75E-107 | Ep-C12 |
| BTBD6 | 1.324 | 0.987 | 0.67 | 6.03E-110 | 1.54E-105 | Ep-C12 |
| MRPS30 | 1.496 | 0.991 | 0.746 | 7.24E-109 | 1.86E-104 | Ep-C12 |
| BEX4 | 1.279 | 0.91 | 0.406 | 4.92E-108 | 1.26E-103 | Ep-C12 |
| NUDT2 | 1.258 | 0.957 | 0.59 | 9.16E-108 | 2.35E-103 | Ep-C12 |
| IGFBP4 | 1.425 | 1 | 0.699 | 2.21E-107 | 5.67E-103 | Ep-C12 |
| FTL | 1.552 | 1 | 1 | 1.28E-106 | 3.28E-102 | Ep-C12 |
| AKT1 | 1.286 | 0.991 | 0.779 | 2.87E-106 | 7.37E-102 | Ep-C12 |
| ARPC5 | 1.206 | 0.996 | 0.979 | 9.82E-106 | 2.52E-101 | Ep-C12 |
| KLHL42 | 1.195 | 0.893 | 0.436 | 4.41E-105 | 1.13E-100 | Ep-C12 |
| ZNF667-AS1 | 1.063 | 0.654 | 0.095 | 8.27E-105 | 2.12E-100 | Ep-C12 |
| CRIP1 | 1.599 | 0.991 | 0.76 | 1.75E-104 | 4.48E-100 | Ep-C12 |
| GSTO1 | 1.27 | 0.996 | 0.939 | 4.96E-104 | 1.27E-99 | Ep-C12 |
| FAM133A | 0.827 | 0.483 | 0.029 | 1.41E-101 | 3.61E-97 | Ep-C12 |
| ETNK1 | 1.156 | 0.919 | 0.606 | 2.62E-100 | 6.72E-96 | Ep-C12 |
| TOMM6 | 0.986 | 1 | 0.993 | 6.94E-100 | 1.78E-95 | Ep-C12 |
| ATP2B1 | 1.259 | 0.966 | 0.773 | 1.72E-97 | 4.41E-93 | Ep-C12 |
| DEDD2 | 1.11 | 0.88 | 0.465 | 2.58E-97 | 6.62E-93 | Ep-C12 |
| HSPA8 | 1.295 | 1 | 0.973 | 4.61E-97 | 1.18E-92 | Ep-C12 |
| BRD2 | 1.092 | 1 | 0.909 | 9.63E-97 | 2.47E-92 | Ep-C12 |
| TMEM14B | 1.116 | 0.987 | 0.848 | 1.61E-96 | 4.13E-92 | Ep-C12 |
| MAP1LC3A | 1.337 | 0.927 | 0.39 | 3.36E-95 | 8.60E-91 | Ep-C12 |
| NDUFAF4 | 1.093 | 0.863 | 0.5 | 7.55E-95 | 1.94E-90 | Ep-C12 |
| CT69 | 1.429 | 0.479 | 0.052 | 1.50E-94 | 3.85E-90 | Ep-C12 |
| HSPA1A | 1.628 | 1 | 0.962 | 1.56E-94 | 3.99E-90 | Ep-C12 |
| UCHL1 | 1.505 | 0.88 | 0.297 | 6.05E-93 | 1.55E-88 | Ep-C12 |
| TGIF1 | 1.239 | 0.962 | 0.732 | 1.33E-92 | 3.42E-88 | Ep-C12 |
| TM7SF3 | 1.065 | 0.915 | 0.593 | 3.60E-92 | 9.23E-88 | Ep-C12 |
| ARL4D | 1.387 | 0.936 | 0.561 | 3.72E-92 | 9.53E-88 | Ep-C12 |
| CLDN20 | 0.89 | 0.444 | 0.041 | 1.29E-91 | 3.30E-87 | Ep-C12 |
| MRPL50 | 1.002 | 0.932 | 0.714 | 1.61E-91 | 4.13E-87 | Ep-C12 |
| COX7A2 | 0.868 | 1 | 0.999 | 3.12E-89 | 7.99E-85 | Ep-C12 |
| SNHG5 | 1.031 | 0.991 | 0.847 | 7.71E-88 | 1.98E-83 | Ep-C12 |
| RPL27 | 0.795 | 1 | 1 | 1.04E-87 | 2.66E-83 | Ep-C12 |
| RPL22L1 | 1.11 | 1 | 0.989 | 4.18E-87 | 1.07E-82 | Ep-C12 |
| DNAJC19 | 0.98 | 1 | 0.957 | 4.62E-87 | 1.18E-82 | Ep-C12 |
| CCN1 | 1.411 | 0.786 | 0.255 | 7.07E-87 | 1.81E-82 | Ep-C12 |
| UBE2R2 | 1.094 | 0.983 | 0.78 | 2.85E-86 | 7.29E-82 | Ep-C12 |
| NDUFB2 | 0.994 | 1 | 0.981 | 1.05E-85 | 2.69E-81 | Ep-C12 |
| HSPA1B | 1.412 | 1 | 0.822 | 1.87E-85 | 4.80E-81 | Ep-C12 |
| TRMT10C | 0.99 | 0.962 | 0.798 | 8.01E-85 | 2.05E-80 | Ep-C12 |
| RSRC2 | 0.928 | 0.996 | 0.908 | 3.28E-84 | 8.40E-80 | Ep-C12 |
| DNAJB4 | 1.372 | 0.803 | 0.439 | 6.99E-84 | 1.79E-79 | Ep-C12 |
| SPSB1 | 0.965 | 0.786 | 0.393 | 3.63E-83 | 9.29E-79 | Ep-C12 |
| GOLT1B | 1.045 | 0.889 | 0.488 | 3.63E-83 | 9.30E-79 | Ep-C12 |
| PTHLH | 1.79 | 0.97 | 0.611 | 1.10E-81 | 2.82E-77 | Ep-C12 |
| SNRPN | 1.091 | 0.812 | 0.332 | 4.18E-81 | 1.07E-76 | Ep-C12 |
| AKR1C3 | 1.415 | 1 | 0.863 | 1.38E-80 | 3.53E-76 | Ep-C12 |
| EIF1 | 0.761 | 1 | 1 | 1.41E-80 | 3.62E-76 | Ep-C12 |
| NIT2 | 0.996 | 0.979 | 0.873 | 6.36E-79 | 1.63E-74 | Ep-C12 |
| MAPK13 | 0.97 | 0.949 | 0.749 | 2.16E-78 | 5.54E-74 | Ep-C12 |
| JAG2 | 1.254 | 0.88 | 0.441 | 2.39E-78 | 6.11E-74 | Ep-C12 |
| C11orf1 | 0.922 | 0.842 | 0.523 | 1.57E-77 | 4.01E-73 | Ep-C12 |
| RSRP1 | 1.067 | 0.944 | 0.725 | 2.35E-77 | 6.02E-73 | Ep-C12 |
| TAF1D | 0.936 | 0.991 | 0.909 | 3.33E-77 | 8.53E-73 | Ep-C12 |
| ACTR6 | 0.873 | 0.816 | 0.526 | 7.47E-77 | 1.91E-72 | Ep-C12 |
| TMEM14C | 0.919 | 1 | 0.889 | 1.59E-76 | 4.08E-72 | Ep-C12 |
| HAS3 | 1.29 | 0.923 | 0.564 | 1.67E-76 | 4.28E-72 | Ep-C12 |
| IFRD1 | 1.133 | 0.91 | 0.637 | 2.28E-76 | 5.83E-72 | Ep-C12 |
| SINHCAF | 0.929 | 0.979 | 0.832 | 5.16E-76 | 1.32E-71 | Ep-C12 |
| KRAS | 0.919 | 0.987 | 0.816 | 6.55E-76 | 1.68E-71 | Ep-C12 |
| SRSF10 | 0.85 | 0.974 | 0.87 | 4.62E-75 | 1.18E-70 | Ep-C12 |
| MED21 | 0.965 | 0.953 | 0.703 | 7.58E-75 | 1.94E-70 | Ep-C12 |
| AKR1C1 | 1.402 | 0.991 | 0.774 | 8.68E-75 | 2.23E-70 | Ep-C12 |
| SRP14 | 0.704 | 1 | 1 | 1.31E-74 | 3.37E-70 | Ep-C12 |
| BICD2 | 0.977 | 0.915 | 0.714 | 1.50E-74 | 3.85E-70 | Ep-C12 |
| SNRPB2 | 0.852 | 0.974 | 0.863 | 5.11E-74 | 1.31E-69 | Ep-C12 |
| NEDD9 | 1.313 | 0.769 | 0.323 | 7.78E-74 | 2.00E-69 | Ep-C12 |
| MTHFS | 0.9 | 0.838 | 0.472 | 3.31E-73 | 8.47E-69 | Ep-C12 |
| GNG10 | 0.84 | 0.996 | 0.926 | 4.74E-73 | 1.22E-68 | Ep-C12 |
| JAKMIP2 | 0.861 | 0.491 | 0.063 | 1.09E-72 | 2.80E-68 | Ep-C12 |
| ZFAS1 | 0.848 | 1 | 0.999 | 3.05E-72 | 7.82E-68 | Ep-C12 |
| LDHB | 1.064 | 1 | 0.979 | 3.77E-72 | 9.65E-68 | Ep-C12 |
| PODXL2 | 1.041 | 0.889 | 0.393 | 1.77E-71 | 4.55E-67 | Ep-C12 |
| STMN1 | 1.145 | 0.996 | 0.878 | 1.80E-71 | 4.62E-67 | Ep-C12 |
| MRPS35 | 0.92 | 0.966 | 0.733 | 2.11E-71 | 5.40E-67 | Ep-C12 |
| KRBOX1 | 1.001 | 0.722 | 0.214 | 2.31E-71 | 5.92E-67 | Ep-C12 |
| SLC4A11 | 1.142 | 0.679 | 0.21 | 3.57E-71 | 9.14E-67 | Ep-C12 |
| NAV1 | 1.025 | 0.769 | 0.373 | 4.97E-71 | 1.27E-66 | Ep-C12 |
| CACNA2D3 | 0.828 | 0.5 | 0.127 | 6.23E-71 | 1.60E-66 | Ep-C12 |
| MARCKSL1 | 1.013 | 0.996 | 0.859 | 1.33E-70 | 3.41E-66 | Ep-C12 |
| TP63 | 0.962 | 0.996 | 0.895 | 1.68E-70 | 4.31E-66 | Ep-C12 |
| ISCA1 | 0.865 | 0.915 | 0.69 | 2.42E-70 | 6.20E-66 | Ep-C12 |
| RHOB | 1.129 | 0.991 | 0.788 | 4.09E-70 | 1.05E-65 | Ep-C12 |
| SRSF7 | 0.876 | 1 | 0.981 | 7.82E-70 | 2.00E-65 | Ep-C12 |
| TSTD1 | 0.914 | 0.987 | 0.904 | 4.45E-69 | 1.14E-64 | Ep-C12 |
| CUTA | 0.885 | 0.987 | 0.937 | 4.85E-69 | 1.24E-64 | Ep-C12 |
| ARL6IP6 | 0.95 | 0.868 | 0.513 | 6.12E-69 | 1.57E-64 | Ep-C12 |
| EHMT1 | 0.934 | 0.769 | 0.467 | 4.23E-68 | 1.08E-63 | Ep-C12 |
| PHYHD1 | 0.888 | 0.679 | 0.227 | 4.77E-68 | 1.22E-63 | Ep-C12 |
| NDUFA4L2 | 1.399 | 0.953 | 0.497 | 5.56E-68 | 1.42E-63 | Ep-C12 |
| FGFR2 | 1.047 | 0.726 | 0.296 | 1.03E-66 | 2.64E-62 | Ep-C12 |
| DCTN3 | 0.92 | 1 | 0.918 | 1.34E-66 | 3.43E-62 | Ep-C12 |
| SRSF3 | 0.788 | 1 | 0.997 | 2.23E-66 | 5.72E-62 | Ep-C12 |
| AL078590.2 | 0.781 | 0.282 | 0.016 | 2.50E-66 | 6.41E-62 | Ep-C12 |
| PRPF38B | 0.79 | 0.97 | 0.793 | 7.15E-66 | 1.83E-61 | Ep-C12 |
| GSTM3 | 1.273 | 0.991 | 0.822 | 1.95E-65 | 5.00E-61 | Ep-C12 |
| WDR33 | 0.826 | 0.953 | 0.75 | 2.59E-65 | 6.64E-61 | Ep-C12 |
| TMEM267 | 1.006 | 0.889 | 0.374 | 3.02E-65 | 7.74E-61 | Ep-C12 |
| SNHG12 | 0.986 | 0.795 | 0.465 | 7.04E-65 | 1.80E-60 | Ep-C12 |
| FST | 1.244 | 0.906 | 0.561 | 1.16E-64 | 2.98E-60 | Ep-C12 |
| TOMM7 | 0.685 | 1 | 0.999 | 1.96E-64 | 5.01E-60 | Ep-C12 |
| FAM120AOS | 0.822 | 0.859 | 0.66 | 2.16E-64 | 5.53E-60 | Ep-C12 |
| IDI1 | 1.053 | 0.919 | 0.716 | 3.07E-64 | 7.87E-60 | Ep-C12 |
| HAPLN2 | 0.651 | 0.491 | 0.073 | 4.61E-64 | 1.18E-59 | Ep-C12 |
| SNRPE | 0.757 | 1 | 0.988 | 6.02E-64 | 1.54E-59 | Ep-C12 |
| EIF4A2 | 0.859 | 1 | 0.996 | 6.43E-64 | 1.65E-59 | Ep-C12 |
| RPP25L | 0.937 | 0.85 | 0.525 | 7.16E-64 | 1.84E-59 | Ep-C12 |
| RIC3 | 0.881 | 0.641 | 0.155 | 2.13E-63 | 5.46E-59 | Ep-C12 |
| CEP170B | 0.999 | 0.88 | 0.422 | 9.08E-63 | 2.33E-58 | Ep-C12 |
| TOM1L1 | 0.829 | 0.791 | 0.437 | 3.06E-62 | 7.83E-58 | Ep-C12 |
| PPIA | 0.715 | 1 | 1 | 2.73E-61 | 7.01E-57 | Ep-C12 |
| BAG1 | 0.945 | 0.991 | 0.893 | 3.07E-61 | 7.88E-57 | Ep-C12 |
| TSEN34 | 0.782 | 0.936 | 0.756 | 3.64E-61 | 9.32E-57 | Ep-C12 |
| RND3 | 1.185 | 0.983 | 0.805 | 5.81E-61 | 1.49E-56 | Ep-C12 |
| ERP29 | 0.807 | 1 | 0.967 | 1.46E-60 | 3.74E-56 | Ep-C12 |
| SIGMAR1 | 1.01 | 0.897 | 0.571 | 1.75E-60 | 4.50E-56 | Ep-C12 |
| AHSA1 | 0.86 | 0.97 | 0.772 | 2.89E-60 | 7.41E-56 | Ep-C12 |
| CBR1 | 1.164 | 0.996 | 0.939 | 3.80E-60 | 9.73E-56 | Ep-C12 |
| FOS | 1.335 | 0.996 | 0.95 | 6.75E-60 | 1.73E-55 | Ep-C12 |
| AC007996.1 | 0.628 | 0.423 | 0.073 | 1.25E-59 | 3.20E-55 | Ep-C12 |
| TUBA1A | 1.25 | 0.825 | 0.423 | 1.85E-59 | 4.75E-55 | Ep-C12 |
| LAMTOR5 | 0.723 | 1 | 0.971 | 2.37E-59 | 6.08E-55 | Ep-C12 |
| GMPR | 0.636 | 0.427 | 0.062 | 4.45E-59 | 1.14E-54 | Ep-C12 |
| INSIG1 | 1.095 | 0.782 | 0.461 | 4.46E-59 | 1.14E-54 | Ep-C12 |
| SEZ6L2 | 0.929 | 0.735 | 0.262 | 5.66E-59 | 1.45E-54 | Ep-C12 |
| HSP90AA1 | 0.914 | 1 | 1 | 7.06E-59 | 1.81E-54 | Ep-C12 |
| CDCA4 | 0.914 | 0.974 | 0.608 | 8.00E-59 | 2.05E-54 | Ep-C12 |
| FXYD6 | 0.819 | 0.611 | 0.17 | 8.98E-59 | 2.30E-54 | Ep-C12 |
| KRT8 | 1.142 | 1 | 0.85 | 1.42E-58 | 3.64E-54 | Ep-C12 |
| RPL35A | 0.581 | 1 | 1 | 3.32E-58 | 8.50E-54 | Ep-C12 |
| ZFAND2A | 1.277 | 0.761 | 0.609 | 6.23E-58 | 1.60E-53 | Ep-C12 |
| SRSF6 | 0.795 | 0.897 | 0.639 | 1.52E-57 | 3.91E-53 | Ep-C12 |
| MRPS28 | 0.772 | 0.94 | 0.809 | 2.54E-57 | 6.50E-53 | Ep-C12 |
| ERGIC2 | 0.821 | 0.979 | 0.792 | 3.13E-57 | 8.02E-53 | Ep-C12 |
| UBAP1 | 0.947 | 0.846 | 0.413 | 3.28E-57 | 8.40E-53 | Ep-C12 |
| IGFBP2 | 1.278 | 1 | 0.92 | 4.17E-57 | 1.07E-52 | Ep-C12 |
| AC105460.1 | 0.922 | 0.829 | 0.317 | 6.04E-57 | 1.55E-52 | Ep-C12 |
| UNC50 | 0.803 | 0.868 | 0.557 | 6.70E-57 | 1.72E-52 | Ep-C12 |
| MYH11 | 1.077 | 0.47 | 0.079 | 6.73E-57 | 1.73E-52 | Ep-C12 |
| DGKA | 0.831 | 0.756 | 0.422 | 6.87E-57 | 1.76E-52 | Ep-C12 |
| ETFRF1 | 0.789 | 0.795 | 0.461 | 8.02E-57 | 2.06E-52 | Ep-C12 |
| DDIT3 | 1.04 | 0.962 | 0.723 | 8.03E-57 | 2.06E-52 | Ep-C12 |
| RNF168 | 0.963 | 0.957 | 0.821 | 1.27E-56 | 3.25E-52 | Ep-C12 |
| NGRN | 0.744 | 0.966 | 0.838 | 1.44E-56 | 3.68E-52 | Ep-C12 |
| BCAS2 | 0.78 | 0.957 | 0.809 | 2.10E-56 | 5.38E-52 | Ep-C12 |
| LYRM4 | 0.822 | 0.868 | 0.573 | 2.54E-56 | 6.52E-52 | Ep-C12 |
| AC023157.3 | 1.058 | 0.769 | 0.316 | 3.35E-56 | 8.58E-52 | Ep-C12 |
| HINT1 | 0.628 | 1 | 0.999 | 5.04E-56 | 1.29E-51 | Ep-C12 |
| DNPH1 | 0.794 | 0.979 | 0.851 | 1.47E-55 | 3.78E-51 | Ep-C12 |
| ZNRD1 | 0.788 | 0.902 | 0.643 | 2.85E-55 | 7.32E-51 | Ep-C12 |
| SPX | 0.528 | 0.338 | 0.039 | 9.41E-55 | 2.41E-50 | Ep-C12 |
| ABT1 | 0.784 | 0.778 | 0.497 | 1.30E-54 | 3.34E-50 | Ep-C12 |
| LINC01578 | 0.805 | 1 | 0.95 | 2.03E-54 | 5.21E-50 | Ep-C12 |
| NME3 | 0.74 | 0.979 | 0.867 | 2.88E-54 | 7.37E-50 | Ep-C12 |
| ACYP2 | 0.884 | 0.799 | 0.393 | 3.00E-54 | 7.69E-50 | Ep-C12 |
| EGR2 | 1.022 | 0.62 | 0.266 | 3.00E-54 | 7.70E-50 | Ep-C12 |
| ATP6V1G1 | 0.708 | 1 | 0.998 | 3.27E-54 | 8.39E-50 | Ep-C12 |
| ENY2 | 0.719 | 1 | 0.983 | 4.86E-54 | 1.25E-49 | Ep-C12 |
| EPC1 | 0.759 | 0.722 | 0.432 | 9.80E-54 | 2.51E-49 | Ep-C12 |
| TSPYL1 | 0.767 | 0.829 | 0.599 | 1.38E-53 | 3.53E-49 | Ep-C12 |
| AOPEP | 0.874 | 0.996 | 0.951 | 3.28E-53 | 8.42E-49 | Ep-C12 |
| GSN | 0.965 | 0.966 | 0.845 | 3.49E-53 | 8.94E-49 | Ep-C12 |
| SRSF2 | 0.723 | 1 | 0.988 | 4.82E-53 | 1.24E-48 | Ep-C12 |
| POP5 | 0.749 | 0.919 | 0.683 | 7.05E-53 | 1.81E-48 | Ep-C12 |
| CAMK2N1 | 0.911 | 0.709 | 0.249 | 9.58E-53 | 2.46E-48 | Ep-C12 |
| SOX2 | 1.2 | 0.944 | 0.738 | 1.34E-52 | 3.42E-48 | Ep-C12 |
| WNK2 | 0.864 | 0.654 | 0.218 | 1.92E-52 | 4.91E-48 | Ep-C12 |
| MTCH1 | 0.686 | 1 | 0.968 | 2.62E-52 | 6.71E-48 | Ep-C12 |
| ADRA1D | 0.579 | 0.222 | 0.012 | 3.80E-52 | 9.74E-48 | Ep-C12 |
| TRA2B | 0.751 | 1 | 0.973 | 3.91E-52 | 1.00E-47 | Ep-C12 |
| HAGLROS | 0.876 | 0.671 | 0.237 | 4.31E-52 | 1.10E-47 | Ep-C12 |
| TRMT112 | 0.678 | 1 | 0.994 | 5.51E-52 | 1.41E-47 | Ep-C12 |
| CHMP5 | 0.767 | 0.996 | 0.895 | 7.26E-52 | 1.86E-47 | Ep-C12 |
| LSM12 | 0.711 | 0.91 | 0.71 | 9.64E-52 | 2.47E-47 | Ep-C12 |
| TMEM158 | 1.157 | 0.812 | 0.416 | 1.33E-51 | 3.40E-47 | Ep-C12 |
| TMEM116 | 0.822 | 0.517 | 0.168 | 1.59E-51 | 4.07E-47 | Ep-C12 |
| TXN | 0.801 | 1 | 1 | 1.75E-51 | 4.49E-47 | Ep-C12 |
| IFI27L2 | 0.712 | 0.991 | 0.931 | 2.00E-51 | 5.13E-47 | Ep-C12 |
| EIF5 | 0.707 | 1 | 0.994 | 2.13E-51 | 5.45E-47 | Ep-C12 |
| AL590483.2 | 0.621 | 0.432 | 0.073 | 2.28E-51 | 5.85E-47 | Ep-C12 |
| TMEM87A | 0.791 | 0.902 | 0.706 | 2.81E-51 | 7.21E-47 | Ep-C12 |
| PRXL2C | 0.788 | 0.739 | 0.379 | 3.02E-51 | 7.74E-47 | Ep-C12 |
| AC025171.1 | 0.808 | 0.632 | 0.191 | 3.05E-51 | 7.83E-47 | Ep-C12 |
| DCAF12 | 0.889 | 0.829 | 0.409 | 4.26E-51 | 1.09E-46 | Ep-C12 |
| ERG28 | 0.768 | 0.936 | 0.774 | 7.02E-51 | 1.80E-46 | Ep-C12 |
| PRKRA | 0.72 | 0.868 | 0.621 | 1.11E-50 | 2.84E-46 | Ep-C12 |
| AKIRIN1 | 0.747 | 0.872 | 0.661 | 1.63E-50 | 4.18E-46 | Ep-C12 |
| SON | 0.74 | 0.996 | 0.95 | 2.09E-50 | 5.36E-46 | Ep-C12 |
| MRPS21 | 0.742 | 0.991 | 0.917 | 2.28E-50 | 5.84E-46 | Ep-C12 |
| NDUFB4 | 0.68 | 1 | 0.997 | 2.76E-50 | 7.07E-46 | Ep-C12 |
| AL365181.4 | 0.52 | 0.376 | 0.052 | 3.02E-50 | 7.73E-46 | Ep-C12 |
| FKBP10 | 0.788 | 0.594 | 0.163 | 1.07E-49 | 2.75E-45 | Ep-C12 |
| CCDC91 | 0.711 | 0.957 | 0.657 | 1.18E-49 | 3.02E-45 | Ep-C12 |
| OSER1 | 0.768 | 0.902 | 0.701 | 1.39E-49 | 3.55E-45 | Ep-C12 |
| SLCO1B3 | 1.003 | 0.735 | 0.322 | 1.81E-49 | 4.65E-45 | Ep-C12 |
| SOX4 | 0.882 | 0.996 | 0.836 | 2.26E-49 | 5.80E-45 | Ep-C12 |
| BTG1 | 0.946 | 1 | 0.985 | 2.91E-49 | 7.47E-45 | Ep-C12 |
| RPL24 | 0.607 | 1 | 1 | 3.24E-49 | 8.32E-45 | Ep-C12 |
| AKR1C2 | 0.928 | 0.991 | 0.838 | 8.52E-49 | 2.18E-44 | Ep-C12 |
| MRPL42 | 0.691 | 0.953 | 0.772 | 9.31E-49 | 2.39E-44 | Ep-C12 |
| CDK4 | 0.764 | 0.962 | 0.804 | 1.32E-48 | 3.38E-44 | Ep-C12 |
| NUPR2 | 0.724 | 0.59 | 0.17 | 1.45E-48 | 3.71E-44 | Ep-C12 |
| LYRM2 | 0.736 | 0.833 | 0.602 | 1.49E-48 | 3.81E-44 | Ep-C12 |
| ARPP19 | 0.696 | 0.97 | 0.773 | 1.88E-48 | 4.82E-44 | Ep-C12 |
| COX20 | 0.712 | 0.983 | 0.904 | 1.95E-48 | 4.99E-44 | Ep-C12 |
| SNRPF | 0.72 | 1 | 0.975 | 2.30E-48 | 5.89E-44 | Ep-C12 |
| CALM1 | 0.676 | 1 | 0.999 | 5.66E-48 | 1.45E-43 | Ep-C12 |
| SLC25A33 | 0.77 | 0.795 | 0.525 | 5.84E-48 | 1.50E-43 | Ep-C12 |
| TXNDC5 | 0.737 | 0.816 | 0.554 | 7.58E-48 | 1.94E-43 | Ep-C12 |
| ULBP2 | 0.883 | 0.765 | 0.49 | 9.09E-48 | 2.33E-43 | Ep-C12 |
| CMAS | 0.732 | 0.936 | 0.724 | 1.06E-47 | 2.72E-43 | Ep-C12 |
| PAK1 | 1.024 | 0.829 | 0.555 | 1.12E-47 | 2.88E-43 | Ep-C12 |
| BEX2 | 0.855 | 0.863 | 0.527 | 1.92E-47 | 4.91E-43 | Ep-C12 |
| PPIG | 0.669 | 0.991 | 0.942 | 2.18E-47 | 5.59E-43 | Ep-C12 |
| SNRPB | 0.666 | 1 | 0.997 | 2.45E-47 | 6.28E-43 | Ep-C12 |
| ADM | 0.982 | 0.962 | 0.738 | 2.67E-47 | 6.84E-43 | Ep-C12 |
| DUSP6 | 1.059 | 0.774 | 0.409 | 2.69E-47 | 6.88E-43 | Ep-C12 |
| OSGIN1 | 1.006 | 0.718 | 0.305 | 3.24E-47 | 8.31E-43 | Ep-C12 |
| SEC61B | 0.62 | 1 | 0.997 | 3.61E-47 | 9.25E-43 | Ep-C12 |
| MTX2 | 0.706 | 0.936 | 0.728 | 4.07E-47 | 1.04E-42 | Ep-C12 |
| TRA2A | 0.766 | 0.897 | 0.671 | 4.43E-47 | 1.14E-42 | Ep-C12 |
| CDH11 | 0.858 | 0.564 | 0.16 | 4.46E-47 | 1.14E-42 | Ep-C12 |
| YARS2 | 0.774 | 0.774 | 0.458 | 5.65E-47 | 1.45E-42 | Ep-C12 |
| PCSK1N | 0.754 | 0.449 | 0.087 | 7.57E-47 | 1.94E-42 | Ep-C12 |
| ETV4 | 0.779 | 0.675 | 0.262 | 8.67E-47 | 2.22E-42 | Ep-C12 |
| MYO10 | 0.825 | 0.944 | 0.626 | 9.43E-47 | 2.42E-42 | Ep-C12 |
| NOP10 | 0.671 | 1 | 0.96 | 1.36E-46 | 3.48E-42 | Ep-C12 |
| ZNF749 | 0.728 | 0.714 | 0.339 | 1.56E-46 | 3.99E-42 | Ep-C12 |
| NR4A1 | 0.98 | 0.897 | 0.623 | 1.64E-46 | 4.21E-42 | Ep-C12 |
| THYN1 | 0.732 | 0.726 | 0.452 | 8.11E-46 | 2.08E-41 | Ep-C12 |
| NPPC | 1.152 | 0.444 | 0.114 | 9.81E-46 | 2.51E-41 | Ep-C12 |
| BCAM | 0.979 | 0.769 | 0.48 | 1.56E-45 | 4.00E-41 | Ep-C12 |
| MRPS26 | 0.687 | 0.983 | 0.894 | 1.63E-45 | 4.19E-41 | Ep-C12 |
| CCT2 | 0.673 | 0.996 | 0.943 | 1.69E-45 | 4.34E-41 | Ep-C12 |
| CKB | 0.811 | 0.996 | 0.858 | 1.83E-45 | 4.69E-41 | Ep-C12 |
| HMCES | 0.721 | 0.885 | 0.658 | 3.59E-45 | 9.20E-41 | Ep-C12 |
| PAK1IP1 | 0.732 | 0.791 | 0.493 | 3.93E-45 | 1.01E-40 | Ep-C12 |
| FKBP9 | 0.838 | 0.833 | 0.487 | 4.13E-45 | 1.06E-40 | Ep-C12 |
| GTF2B | 0.708 | 0.816 | 0.634 | 4.76E-45 | 1.22E-40 | Ep-C12 |
| SOD1 | 0.643 | 1 | 0.995 | 6.84E-45 | 1.75E-40 | Ep-C12 |
| LINC02562 | 0.768 | 0.684 | 0.39 | 7.15E-45 | 1.83E-40 | Ep-C12 |
| TRIM28 | 0.712 | 0.97 | 0.811 | 7.55E-45 | 1.93E-40 | Ep-C12 |
| KRT10 | 0.589 | 0.996 | 0.997 | 9.15E-45 | 2.35E-40 | Ep-C12 |
| PAPOLA | 0.66 | 1 | 0.961 | 9.82E-45 | 2.52E-40 | Ep-C12 |
| MSRB2 | 0.713 | 0.726 | 0.501 | 1.28E-44 | 3.29E-40 | Ep-C12 |
| PRDX6 | 0.641 | 1 | 0.996 | 2.27E-44 | 5.81E-40 | Ep-C12 |
| TMEM98 | 0.784 | 0.769 | 0.378 | 3.08E-44 | 7.90E-40 | Ep-C12 |
| CHORDC1 | 0.746 | 0.825 | 0.606 | 3.18E-44 | 8.15E-40 | Ep-C12 |
| FAM241B | 0.687 | 0.624 | 0.311 | 3.87E-44 | 9.92E-40 | Ep-C12 |
| AMD1 | 0.717 | 0.962 | 0.855 | 6.55E-44 | 1.68E-39 | Ep-C12 |
| BYSL | 0.661 | 0.641 | 0.362 | 6.66E-44 | 1.71E-39 | Ep-C12 |
| MGLL | 0.827 | 0.573 | 0.189 | 7.29E-44 | 1.87E-39 | Ep-C12 |
| UBC | 0.701 | 1 | 1 | 1.40E-43 | 3.58E-39 | Ep-C12 |
| UBE2N | 0.653 | 0.957 | 0.881 | 1.71E-43 | 4.38E-39 | Ep-C12 |
| PPT1 | 0.764 | 0.893 | 0.641 | 2.24E-43 | 5.75E-39 | Ep-C12 |
| NNT | 0.79 | 0.812 | 0.414 | 2.47E-43 | 6.34E-39 | Ep-C12 |
| DNAH14 | 0.673 | 0.59 | 0.217 | 2.89E-43 | 7.40E-39 | Ep-C12 |
| SLC16A8 | 0.653 | 0.521 | 0.149 | 2.97E-43 | 7.62E-39 | Ep-C12 |
| HDAC2 | 0.669 | 0.902 | 0.778 | 3.37E-43 | 8.64E-39 | Ep-C12 |
| SNX3 | 0.598 | 1 | 0.979 | 3.37E-43 | 8.65E-39 | Ep-C12 |
| PRKAG2-AS1 | 0.65 | 0.543 | 0.162 | 3.99E-43 | 1.02E-38 | Ep-C12 |
| DUSP14 | 0.828 | 0.833 | 0.557 | 4.26E-43 | 1.09E-38 | Ep-C12 |
| RAB7A | 0.644 | 0.996 | 0.974 | 4.48E-43 | 1.15E-38 | Ep-C12 |
| TBPL1 | 0.699 | 0.748 | 0.484 | 5.39E-43 | 1.38E-38 | Ep-C12 |
| FXR1 | 0.625 | 1 | 0.949 | 5.90E-43 | 1.51E-38 | Ep-C12 |
| HEXIM1 | 0.792 | 0.786 | 0.46 | 6.16E-43 | 1.58E-38 | Ep-C12 |
| POLR2H | 0.69 | 0.996 | 0.96 | 7.03E-43 | 1.80E-38 | Ep-C12 |
| EPB41L4A-AS1 | 0.74 | 0.855 | 0.585 | 7.92E-43 | 2.03E-38 | Ep-C12 |
| PTS | 0.69 | 0.923 | 0.771 | 8.46E-43 | 2.17E-38 | Ep-C12 |
| EGR1 | 1.065 | 0.94 | 0.808 | 8.94E-43 | 2.29E-38 | Ep-C12 |
| LSM2 | 0.64 | 0.987 | 0.904 | 9.84E-43 | 2.52E-38 | Ep-C12 |
| SERF2 | 0.54 | 1 | 1 | 1.02E-42 | 2.61E-38 | Ep-C12 |
| YRDC | 0.682 | 0.748 | 0.502 | 2.48E-42 | 6.36E-38 | Ep-C12 |
| SDHD | 0.647 | 0.996 | 0.901 | 3.05E-42 | 7.81E-38 | Ep-C12 |
| SMIM26 | 0.633 | 0.983 | 0.917 | 3.48E-42 | 8.93E-38 | Ep-C12 |
| LINC02298 | 0.717 | 0.573 | 0.193 | 3.50E-42 | 8.98E-38 | Ep-C12 |
| IFT43 | 0.675 | 0.808 | 0.557 | 4.69E-42 | 1.20E-37 | Ep-C12 |
| CACYBP | 0.741 | 0.991 | 0.933 | 5.70E-42 | 1.46E-37 | Ep-C12 |
| COX7A1 | 0.629 | 0.487 | 0.119 | 7.45E-42 | 1.91E-37 | Ep-C12 |
| STMP1 | 0.684 | 0.987 | 0.881 | 9.47E-42 | 2.43E-37 | Ep-C12 |
| SELENOT | 0.645 | 0.996 | 0.957 | 9.53E-42 | 2.44E-37 | Ep-C12 |
| ZNHIT3 | 0.643 | 0.91 | 0.742 | 1.48E-41 | 3.78E-37 | Ep-C12 |
| TMEM35B | 0.715 | 0.791 | 0.52 | 1.88E-41 | 4.81E-37 | Ep-C12 |
| A1BG | 0.768 | 0.628 | 0.231 | 2.18E-41 | 5.59E-37 | Ep-C12 |
| CYSTM1 | 0.737 | 0.966 | 0.801 | 2.35E-41 | 6.02E-37 | Ep-C12 |
| HACD3 | 0.698 | 0.94 | 0.79 | 2.63E-41 | 6.75E-37 | Ep-C12 |
| DDOST | 0.653 | 0.962 | 0.903 | 2.68E-41 | 6.87E-37 | Ep-C12 |
| AC087612.1 | 0.548 | 0.47 | 0.115 | 2.86E-41 | 7.33E-37 | Ep-C12 |
| COA5 | 0.704 | 0.846 | 0.578 | 3.22E-41 | 8.26E-37 | Ep-C12 |
| FLOT1 | 0.665 | 0.923 | 0.764 | 3.74E-41 | 9.58E-37 | Ep-C12 |
| TIMM8B | 0.674 | 0.987 | 0.932 | 3.83E-41 | 9.81E-37 | Ep-C12 |
| NFE2L2 | 0.813 | 1 | 0.983 | 3.86E-41 | 9.90E-37 | Ep-C12 |
| CYB5A | 0.832 | 0.94 | 0.799 | 4.14E-41 | 1.06E-36 | Ep-C12 |
| SSB | 0.658 | 0.996 | 0.953 | 4.19E-41 | 1.08E-36 | Ep-C12 |
| AL162231.1 | 0.565 | 0.427 | 0.104 | 4.30E-41 | 1.10E-36 | Ep-C12 |
| H1FX | 0.696 | 1 | 0.991 | 4.93E-41 | 1.26E-36 | Ep-C12 |
| ATP5MC3 | 0.646 | 1 | 0.999 | 5.40E-41 | 1.38E-36 | Ep-C12 |
| NDUFS5 | 0.596 | 1 | 0.998 | 5.41E-41 | 1.39E-36 | Ep-C12 |
| ATG101 | 0.666 | 0.897 | 0.727 | 8.03E-41 | 2.06E-36 | Ep-C12 |
| ZSCAN31 | 0.653 | 0.436 | 0.129 | 8.05E-41 | 2.06E-36 | Ep-C12 |
| NXT1 | 0.673 | 0.936 | 0.704 | 9.23E-41 | 2.37E-36 | Ep-C12 |
| MBOAT7 | 0.662 | 0.722 | 0.419 | 9.25E-41 | 2.37E-36 | Ep-C12 |
| AC019080.1 | 0.879 | 0.735 | 0.323 | 1.03E-40 | 2.65E-36 | Ep-C12 |
| SLC27A5 | 0.748 | 0.701 | 0.316 | 1.54E-40 | 3.95E-36 | Ep-C12 |
| UBE2E3 | 0.678 | 0.974 | 0.874 | 2.62E-40 | 6.72E-36 | Ep-C12 |
| H3F3B | 0.512 | 1 | 1 | 2.72E-40 | 6.97E-36 | Ep-C12 |
| ATP5F1C | 0.599 | 0.996 | 0.981 | 3.00E-40 | 7.68E-36 | Ep-C12 |
| TNFRSF18 | 0.742 | 0.94 | 0.784 | 3.17E-40 | 8.13E-36 | Ep-C12 |
| MZT2A | 0.581 | 1 | 0.985 | 5.53E-40 | 1.42E-35 | Ep-C12 |
| MMADHC | 0.614 | 0.97 | 0.81 | 6.21E-40 | 1.59E-35 | Ep-C12 |
| HSPA6 | 1.553 | 0.615 | 0.225 | 6.25E-40 | 1.60E-35 | Ep-C12 |
| UBXN4 | 0.61 | 1 | 0.978 | 7.91E-40 | 2.03E-35 | Ep-C12 |
| MDK | 0.77 | 0.987 | 0.888 | 8.44E-40 | 2.16E-35 | Ep-C12 |
| TSHZ2 | 0.804 | 0.846 | 0.668 | 1.03E-39 | 2.65E-35 | Ep-C12 |
| ANAPC13 | 0.695 | 0.838 | 0.611 | 1.14E-39 | 2.91E-35 | Ep-C12 |
| DPP7 | 0.657 | 0.962 | 0.838 | 1.69E-39 | 4.33E-35 | Ep-C12 |
| HSP90AB1 | 0.593 | 1 | 1 | 2.25E-39 | 5.76E-35 | Ep-C12 |
| MYC | 0.874 | 0.987 | 0.841 | 2.39E-39 | 6.13E-35 | Ep-C12 |
| TM2D3 | 0.647 | 0.739 | 0.485 | 2.48E-39 | 6.35E-35 | Ep-C12 |
| LRRC8A | 0.732 | 0.932 | 0.697 | 2.85E-39 | 7.31E-35 | Ep-C12 |
| ZNF428 | 0.728 | 0.812 | 0.545 | 3.14E-39 | 8.05E-35 | Ep-C12 |
| CLDND1 | 0.718 | 0.966 | 0.801 | 3.45E-39 | 8.85E-35 | Ep-C12 |
| UBB | 0.649 | 1 | 0.999 | 5.08E-39 | 1.30E-34 | Ep-C12 |
| PSMG4 | 0.658 | 0.791 | 0.513 | 5.44E-39 | 1.39E-34 | Ep-C12 |
| ZBTB42 | 0.655 | 0.603 | 0.214 | 6.53E-39 | 1.67E-34 | Ep-C12 |
| NOL7 | 0.629 | 0.974 | 0.878 | 6.75E-39 | 1.73E-34 | Ep-C12 |
| SVIP | 0.785 | 0.692 | 0.336 | 8.14E-39 | 2.09E-34 | Ep-C12 |
| HINT2 | 0.66 | 0.966 | 0.724 | 8.76E-39 | 2.25E-34 | Ep-C12 |
| MICOS10 | 0.578 | 1 | 0.988 | 1.20E-38 | 3.06E-34 | Ep-C12 |
| PPIF | 0.736 | 0.932 | 0.772 | 1.28E-38 | 3.28E-34 | Ep-C12 |
| IP6K2 | 0.644 | 0.876 | 0.669 | 1.38E-38 | 3.53E-34 | Ep-C12 |
| C1orf52 | 0.632 | 0.782 | 0.551 | 2.06E-38 | 5.28E-34 | Ep-C12 |
| PCNA | 0.783 | 0.953 | 0.777 | 2.06E-38 | 5.28E-34 | Ep-C12 |
| CFAP20 | 0.618 | 0.731 | 0.594 | 2.83E-38 | 7.24E-34 | Ep-C12 |
| CAMTA1 | 0.607 | 0.974 | 0.913 | 3.42E-38 | 8.77E-34 | Ep-C12 |
| APOE | 1.068 | 0.615 | 0.27 | 3.60E-38 | 9.24E-34 | Ep-C12 |
| MCUR1 | 0.682 | 0.885 | 0.626 | 3.89E-38 | 9.96E-34 | Ep-C12 |
| ZSCAN18 | 0.631 | 0.615 | 0.245 | 5.08E-38 | 1.30E-33 | Ep-C12 |
| LDLR | 0.785 | 0.919 | 0.758 | 5.42E-38 | 1.39E-33 | Ep-C12 |
| JUN | 0.851 | 0.996 | 0.96 | 5.83E-38 | 1.49E-33 | Ep-C12 |
| SERP1 | 0.603 | 1 | 0.998 | 5.88E-38 | 1.51E-33 | Ep-C12 |
| COX5B | 0.558 | 1 | 1 | 6.79E-38 | 1.74E-33 | Ep-C12 |
| NME1 | 0.651 | 0.991 | 0.959 | 7.94E-38 | 2.03E-33 | Ep-C12 |
| SNHG21 | 0.629 | 0.517 | 0.192 | 9.24E-38 | 2.37E-33 | Ep-C12 |
| TFRC | 0.736 | 0.944 | 0.774 | 9.92E-38 | 2.54E-33 | Ep-C12 |
| RPF2 | 0.631 | 0.808 | 0.584 | 1.09E-37 | 2.78E-33 | Ep-C12 |
| SIGIRR | 0.76 | 0.863 | 0.591 | 1.31E-37 | 3.35E-33 | Ep-C12 |
| TBCC | 0.681 | 0.756 | 0.44 | 1.51E-37 | 3.88E-33 | Ep-C12 |
| SNHG8 | 0.682 | 0.979 | 0.918 | 1.55E-37 | 3.97E-33 | Ep-C12 |
| MTLN | 0.677 | 0.906 | 0.712 | 1.57E-37 | 4.02E-33 | Ep-C12 |
| NDUFC1 | 0.589 | 0.983 | 0.954 | 2.71E-37 | 6.94E-33 | Ep-C12 |
| PINLYP | 0.654 | 0.543 | 0.184 | 2.76E-37 | 7.06E-33 | Ep-C12 |
| RPP25 | 0.722 | 0.726 | 0.393 | 3.51E-37 | 8.99E-33 | Ep-C12 |
| CLEC2L | 0.66 | 0.568 | 0.197 | 3.83E-37 | 9.81E-33 | Ep-C12 |
| NME4 | 0.683 | 0.949 | 0.739 | 7.16E-37 | 1.84E-32 | Ep-C12 |
| CDC26 | 0.633 | 0.91 | 0.73 | 8.70E-37 | 2.23E-32 | Ep-C12 |
| ZNF226 | 0.578 | 0.577 | 0.244 | 1.49E-36 | 3.83E-32 | Ep-C12 |
| SLC25A25 | 0.816 | 0.701 | 0.379 | 2.17E-36 | 5.57E-32 | Ep-C12 |
| RAN | 0.572 | 1 | 0.999 | 2.29E-36 | 5.86E-32 | Ep-C12 |
| CA12 | 0.734 | 0.953 | 0.753 | 2.47E-36 | 6.34E-32 | Ep-C12 |
| TMEM14A | 0.652 | 0.974 | 0.87 | 2.64E-36 | 6.76E-32 | Ep-C12 |
| ARG2 | 0.72 | 0.568 | 0.283 | 3.43E-36 | 8.78E-32 | Ep-C12 |
| GRASP | 0.588 | 0.453 | 0.123 | 3.72E-36 | 9.54E-32 | Ep-C12 |
| KRT18 | 0.675 | 1 | 0.979 | 3.84E-36 | 9.84E-32 | Ep-C12 |
| SBSPON | 0.508 | 0.402 | 0.089 | 5.95E-36 | 1.53E-31 | Ep-C12 |
| OST4 | 0.548 | 1 | 0.996 | 5.97E-36 | 1.53E-31 | Ep-C12 |
| PNISR | 0.665 | 0.953 | 0.851 | 6.07E-36 | 1.56E-31 | Ep-C12 |
| MRPL18 | 0.629 | 0.962 | 0.926 | 8.59E-36 | 2.20E-31 | Ep-C12 |
| VPS25 | 0.608 | 0.97 | 0.824 | 9.16E-36 | 2.35E-31 | Ep-C12 |
| COX7C | 0.518 | 1 | 0.999 | 1.07E-35 | 2.74E-31 | Ep-C12 |
| RHEB | 0.618 | 1 | 0.96 | 1.23E-35 | 3.15E-31 | Ep-C12 |
| CD164 | 0.621 | 0.987 | 0.901 | 1.35E-35 | 3.47E-31 | Ep-C12 |
| RBM39 | 0.564 | 1 | 0.991 | 1.47E-35 | 3.77E-31 | Ep-C12 |
| RAC3 | 0.726 | 0.731 | 0.446 | 1.50E-35 | 3.84E-31 | Ep-C12 |
| TMA7 | 0.523 | 1 | 0.997 | 2.20E-35 | 5.63E-31 | Ep-C12 |
| ETV5 | 0.713 | 0.637 | 0.256 | 2.23E-35 | 5.70E-31 | Ep-C12 |
| PAIP2 | 0.584 | 0.936 | 0.845 | 2.33E-35 | 5.98E-31 | Ep-C12 |
| CASC15 | 0.553 | 0.363 | 0.077 | 2.44E-35 | 6.25E-31 | Ep-C12 |
| NNT-AS1 | 0.814 | 0.722 | 0.377 | 2.62E-35 | 6.71E-31 | Ep-C12 |
| EID1 | 0.647 | 0.987 | 0.913 | 3.26E-35 | 8.35E-31 | Ep-C12 |
| CCDC59 | 0.578 | 0.885 | 0.723 | 4.19E-35 | 1.07E-30 | Ep-C12 |
| TMBIM6 | 0.514 | 1 | 0.999 | 4.20E-35 | 1.08E-30 | Ep-C12 |
| ELOVL5 | 0.659 | 0.739 | 0.493 | 5.72E-35 | 1.47E-30 | Ep-C12 |
| SF3B6 | 0.534 | 1 | 0.996 | 6.53E-35 | 1.67E-30 | Ep-C12 |
| HNRNPA3 | 0.581 | 1 | 0.998 | 7.42E-35 | 1.90E-30 | Ep-C12 |
| ADH5 | 0.603 | 0.996 | 0.943 | 8.37E-35 | 2.15E-30 | Ep-C12 |
| FAM229B | 0.59 | 0.526 | 0.221 | 9.88E-35 | 2.53E-30 | Ep-C12 |
| TOMM5 | 0.608 | 1 | 0.967 | 1.01E-34 | 2.58E-30 | Ep-C12 |
| SYNCRIP | 0.579 | 0.983 | 0.937 | 1.14E-34 | 2.91E-30 | Ep-C12 |
| AC024588.1 | 0.509 | 0.415 | 0.099 | 1.49E-34 | 3.81E-30 | Ep-C12 |
| SF3B5 | 0.545 | 1 | 0.982 | 1.79E-34 | 4.58E-30 | Ep-C12 |
| PDRG1 | 0.582 | 0.679 | 0.482 | 1.82E-34 | 4.67E-30 | Ep-C12 |
| DGUOK | 0.586 | 0.983 | 0.896 | 1.95E-34 | 4.99E-30 | Ep-C12 |
| TNFSF9 | 0.789 | 0.632 | 0.275 | 2.37E-34 | 6.07E-30 | Ep-C12 |
| SWI5 | 0.624 | 0.893 | 0.699 | 3.40E-34 | 8.71E-30 | Ep-C12 |
| CLTA | 0.51 | 1 | 0.995 | 3.60E-34 | 9.23E-30 | Ep-C12 |
| BEX1 | 0.52 | 0.265 | 0.04 | 3.92E-34 | 1.00E-29 | Ep-C12 |
| MORF4L1 | 0.552 | 0.996 | 0.976 | 4.46E-34 | 1.14E-29 | Ep-C12 |
| SHC1 | 0.705 | 0.829 | 0.578 | 4.63E-34 | 1.19E-29 | Ep-C12 |
| NTRK2 | 0.711 | 0.987 | 0.838 | 4.90E-34 | 1.26E-29 | Ep-C12 |
| SF1 | 0.591 | 0.987 | 0.908 | 5.60E-34 | 1.43E-29 | Ep-C12 |
| HDDC2 | 0.573 | 0.936 | 0.813 | 6.20E-34 | 1.59E-29 | Ep-C12 |
| DHX9 | 0.591 | 0.91 | 0.728 | 6.99E-34 | 1.79E-29 | Ep-C12 |
| RSL24D1 | 0.566 | 0.974 | 0.914 | 7.75E-34 | 1.99E-29 | Ep-C12 |
| DYNLL1 | 0.59 | 1 | 0.999 | 9.14E-34 | 2.34E-29 | Ep-C12 |
| MTA1 | 0.631 | 0.782 | 0.499 | 9.97E-34 | 2.56E-29 | Ep-C12 |
| VSNL1 | 0.81 | 0.906 | 0.719 | 1.10E-33 | 2.81E-29 | Ep-C12 |
| SCCPDH | 0.658 | 0.803 | 0.485 | 1.38E-33 | 3.53E-29 | Ep-C12 |
| GSTM4 | 0.674 | 0.697 | 0.317 | 1.47E-33 | 3.76E-29 | Ep-C12 |
| HNRNPH1 | 0.737 | 0.962 | 0.895 | 1.57E-33 | 4.02E-29 | Ep-C12 |
| BX284668.5 | 0.637 | 0.624 | 0.34 | 1.77E-33 | 4.53E-29 | Ep-C12 |
| DDX5 | 0.582 | 1 | 0.998 | 2.02E-33 | 5.18E-29 | Ep-C12 |
| GPX2 | 0.646 | 1 | 0.853 | 2.21E-33 | 5.66E-29 | Ep-C12 |
| DLX5 | 0.709 | 0.509 | 0.186 | 2.28E-33 | 5.85E-29 | Ep-C12 |
| MLF1 | 0.647 | 0.983 | 0.942 | 2.61E-33 | 6.69E-29 | Ep-C12 |
| KLHL23 | 0.648 | 0.56 | 0.24 | 3.42E-33 | 8.76E-29 | Ep-C12 |
| FGFR1OP2 | 0.628 | 0.91 | 0.648 | 3.65E-33 | 9.35E-29 | Ep-C12 |
| FUS | 0.595 | 0.996 | 0.982 | 4.89E-33 | 1.25E-28 | Ep-C12 |
| SLC25A36 | 0.632 | 0.974 | 0.87 | 5.44E-33 | 1.40E-28 | Ep-C12 |
| SNHG32 | 0.694 | 0.876 | 0.659 | 6.02E-33 | 1.54E-28 | Ep-C12 |
| NOP56 | 0.625 | 0.962 | 0.873 | 6.11E-33 | 1.57E-28 | Ep-C12 |
| ACOT13 | 0.648 | 0.829 | 0.546 | 6.26E-33 | 1.61E-28 | Ep-C12 |
| ALDH3A1 | 1.036 | 0.932 | 0.778 | 7.80E-33 | 2.00E-28 | Ep-C12 |
| MIEN1 | 0.567 | 0.983 | 0.899 | 8.93E-33 | 2.29E-28 | Ep-C12 |
| GTF2H4 | 0.568 | 0.654 | 0.358 | 9.24E-33 | 2.37E-28 | Ep-C12 |
| MRPL47 | 0.552 | 1 | 0.972 | 1.21E-32 | 3.11E-28 | Ep-C12 |
| THAP9-AS1 | 0.652 | 0.735 | 0.468 | 1.21E-32 | 3.11E-28 | Ep-C12 |
| C20orf194 | 0.585 | 0.47 | 0.195 | 1.24E-32 | 3.17E-28 | Ep-C12 |
| AIG1 | 0.596 | 0.91 | 0.715 | 1.37E-32 | 3.51E-28 | Ep-C12 |
| CKS2 | 0.714 | 0.966 | 0.782 | 1.52E-32 | 3.90E-28 | Ep-C12 |
| PRMT1 | 0.562 | 0.996 | 0.956 | 2.50E-32 | 6.40E-28 | Ep-C12 |
| NEK11 | 0.528 | 0.479 | 0.176 | 3.20E-32 | 8.20E-28 | Ep-C12 |
| FAAP20 | 0.555 | 0.932 | 0.823 | 3.39E-32 | 8.69E-28 | Ep-C12 |
| B4GALT4 | 0.887 | 0.791 | 0.533 | 3.75E-32 | 9.61E-28 | Ep-C12 |
| MZT2B | 0.507 | 1 | 0.996 | 4.47E-32 | 1.14E-27 | Ep-C12 |
| TM2D2 | 0.624 | 0.816 | 0.585 | 4.54E-32 | 1.16E-27 | Ep-C12 |
| GTF3C6 | 0.568 | 0.987 | 0.89 | 6.97E-32 | 1.79E-27 | Ep-C12 |
| MRPL3 | 0.564 | 0.996 | 0.952 | 6.99E-32 | 1.79E-27 | Ep-C12 |
| IER5L | 0.792 | 0.842 | 0.65 | 8.75E-32 | 2.24E-27 | Ep-C12 |
| CDK2AP1 | 0.577 | 0.996 | 0.948 | 9.30E-32 | 2.38E-27 | Ep-C12 |
| SMU1 | 0.62 | 0.846 | 0.549 | 9.35E-32 | 2.40E-27 | Ep-C12 |
| SELENOW | 0.56 | 1 | 0.995 | 1.03E-31 | 2.64E-27 | Ep-C12 |
| RPL7L1 | 0.592 | 0.966 | 0.87 | 1.07E-31 | 2.74E-27 | Ep-C12 |
| NSG1 | 0.688 | 0.756 | 0.432 | 1.08E-31 | 2.76E-27 | Ep-C12 |
| SRSF9 | 0.506 | 1 | 0.995 | 1.14E-31 | 2.92E-27 | Ep-C12 |
| MRAP2 | 0.574 | 0.487 | 0.179 | 1.28E-31 | 3.28E-27 | Ep-C12 |
| EEF1E1 | 0.582 | 0.863 | 0.675 | 1.31E-31 | 3.35E-27 | Ep-C12 |
| PPP1R14A | 0.556 | 0.551 | 0.196 | 1.32E-31 | 3.38E-27 | Ep-C12 |
| ATRAID | 0.584 | 0.996 | 0.968 | 1.56E-31 | 4.01E-27 | Ep-C12 |
| GGPS1 | 0.611 | 0.697 | 0.437 | 1.64E-31 | 4.21E-27 | Ep-C12 |
| RASD1 | 0.795 | 0.658 | 0.309 | 1.82E-31 | 4.67E-27 | Ep-C12 |
| RNASET2 | 0.587 | 0.88 | 0.738 | 2.33E-31 | 5.97E-27 | Ep-C12 |
| 9-Mar | 0.608 | 0.628 | 0.361 | 2.34E-31 | 5.99E-27 | Ep-C12 |
| CDC42EP3 | 0.703 | 0.607 | 0.291 | 2.51E-31 | 6.42E-27 | Ep-C12 |
| GPANK1 | 0.586 | 0.658 | 0.338 | 2.71E-31 | 6.94E-27 | Ep-C12 |
| SMIM13 | 0.639 | 0.726 | 0.42 | 2.95E-31 | 7.57E-27 | Ep-C12 |
| RPP21 | 0.549 | 0.97 | 0.896 | 3.00E-31 | 7.68E-27 | Ep-C12 |
| FOXE1 | 0.713 | 0.769 | 0.493 | 3.28E-31 | 8.41E-27 | Ep-C12 |
| MESP1 | 0.582 | 0.53 | 0.193 | 3.35E-31 | 8.57E-27 | Ep-C12 |
| CD9 | 0.528 | 1 | 1 | 4.26E-31 | 1.09E-26 | Ep-C12 |
| SRSF8 | 0.622 | 0.718 | 0.462 | 4.86E-31 | 1.25E-26 | Ep-C12 |
| PFDN4 | 0.557 | 0.953 | 0.877 | 4.94E-31 | 1.27E-26 | Ep-C12 |
| ARL5A | 0.546 | 0.919 | 0.827 | 5.67E-31 | 1.45E-26 | Ep-C12 |
| DIPK2A | 0.657 | 0.667 | 0.386 | 6.47E-31 | 1.66E-26 | Ep-C12 |
| AKIP1 | 0.651 | 0.654 | 0.433 | 6.57E-31 | 1.68E-26 | Ep-C12 |
| RAB4A | 0.548 | 0.966 | 0.896 | 8.15E-31 | 2.09E-26 | Ep-C12 |
| BCL11B | 0.675 | 0.688 | 0.371 | 8.16E-31 | 2.09E-26 | Ep-C12 |
| HAT1 | 0.595 | 0.902 | 0.71 | 9.25E-31 | 2.37E-26 | Ep-C12 |
| SNCA | 0.628 | 0.62 | 0.255 | 9.97E-31 | 2.56E-26 | Ep-C12 |
| AKR7A2 | 0.568 | 0.923 | 0.783 | 1.02E-30 | 2.61E-26 | Ep-C12 |
| TMEM99 | 0.625 | 0.795 | 0.526 | 1.11E-30 | 2.86E-26 | Ep-C12 |
| CLDN7 | 0.502 | 0.966 | 0.686 | 1.12E-30 | 2.87E-26 | Ep-C12 |
| PUM1 | 0.598 | 0.897 | 0.766 | 1.15E-30 | 2.94E-26 | Ep-C12 |
| ATP5MF | 0.508 | 1 | 0.997 | 1.19E-30 | 3.05E-26 | Ep-C12 |
| COX17 | 0.61 | 0.983 | 0.888 | 1.42E-30 | 3.65E-26 | Ep-C12 |
| HTATIP2 | 0.6 | 0.953 | 0.891 | 1.78E-30 | 4.55E-26 | Ep-C12 |
| BDH2 | 0.569 | 0.645 | 0.339 | 1.82E-30 | 4.68E-26 | Ep-C12 |
| HES4 | 0.691 | 0.919 | 0.658 | 2.24E-30 | 5.75E-26 | Ep-C12 |
| PTBP2 | 0.505 | 0.474 | 0.184 | 2.43E-30 | 6.22E-26 | Ep-C12 |
| CNPY2 | 0.559 | 0.987 | 0.877 | 2.47E-30 | 6.34E-26 | Ep-C12 |
| LGMN | 0.612 | 0.786 | 0.503 | 3.14E-30 | 8.05E-26 | Ep-C12 |
| GLO1 | 0.564 | 0.957 | 0.85 | 3.54E-30 | 9.06E-26 | Ep-C12 |
| BRF1 | 0.554 | 0.551 | 0.273 | 3.58E-30 | 9.17E-26 | Ep-C12 |
| PEBP1 | 0.544 | 1 | 0.98 | 4.05E-30 | 1.04E-25 | Ep-C12 |
| STK38L | 0.826 | 0.679 | 0.419 | 4.15E-30 | 1.06E-25 | Ep-C12 |
| TMED10 | 0.555 | 0.996 | 0.972 | 5.35E-30 | 1.37E-25 | Ep-C12 |
| MAPRE1 | 0.564 | 0.94 | 0.801 | 6.17E-30 | 1.58E-25 | Ep-C12 |
| EXOSC3 | 0.605 | 0.821 | 0.565 | 6.76E-30 | 1.73E-25 | Ep-C12 |
| TCTEX1D2 | 0.634 | 0.936 | 0.722 | 7.16E-30 | 1.84E-25 | Ep-C12 |
| SLIRP | 0.54 | 1 | 0.941 | 7.49E-30 | 1.92E-25 | Ep-C12 |
| ARF5 | 0.512 | 0.987 | 0.884 | 8.59E-30 | 2.20E-25 | Ep-C12 |
| AEBP2 | 0.618 | 0.718 | 0.431 | 9.90E-30 | 2.54E-25 | Ep-C12 |
| ATP6V0C | 0.53 | 1 | 0.993 | 1.08E-29 | 2.77E-25 | Ep-C12 |
| RBM17 | 0.55 | 0.962 | 0.817 | 1.70E-29 | 4.36E-25 | Ep-C12 |
| DTWD1 | 0.57 | 0.675 | 0.376 | 1.70E-29 | 4.37E-25 | Ep-C12 |
| ATG5 | 0.559 | 0.791 | 0.617 | 1.74E-29 | 4.46E-25 | Ep-C12 |
| NEFM | 0.565 | 0.329 | 0.074 | 2.25E-29 | 5.77E-25 | Ep-C12 |
| PPP1R10 | 0.62 | 0.829 | 0.557 | 2.75E-29 | 7.04E-25 | Ep-C12 |
| SCN9A | 0.613 | 0.44 | 0.139 | 3.30E-29 | 8.46E-25 | Ep-C12 |
| SPRY2 | 0.661 | 0.479 | 0.185 | 4.08E-29 | 1.05E-24 | Ep-C12 |
| ATP5MC1 | 0.577 | 1 | 0.988 | 4.15E-29 | 1.06E-24 | Ep-C12 |
| MCL1 | 0.603 | 0.983 | 0.949 | 5.10E-29 | 1.31E-24 | Ep-C12 |
| DNAJB1 | 0.658 | 0.996 | 0.95 | 5.64E-29 | 1.45E-24 | Ep-C12 |
| NCBP2 | 0.556 | 0.979 | 0.932 | 5.77E-29 | 1.48E-24 | Ep-C12 |
| C19orf48 | 0.608 | 0.889 | 0.654 | 5.96E-29 | 1.53E-24 | Ep-C12 |
| POLB | 0.555 | 0.641 | 0.326 | 6.35E-29 | 1.63E-24 | Ep-C12 |
| TTC32 | 0.559 | 0.62 | 0.305 | 6.38E-29 | 1.64E-24 | Ep-C12 |
| NOS1AP | 0.643 | 0.513 | 0.211 | 7.43E-29 | 1.91E-24 | Ep-C12 |
| HMGA1 | 0.533 | 1 | 0.992 | 7.55E-29 | 1.94E-24 | Ep-C12 |
| PRPF4B | 0.563 | 0.859 | 0.677 | 8.04E-29 | 2.06E-24 | Ep-C12 |
| PPP1R2 | 0.519 | 0.991 | 0.93 | 8.14E-29 | 2.09E-24 | Ep-C12 |
| TIMMDC1 | 0.549 | 0.962 | 0.9 | 8.31E-29 | 2.13E-24 | Ep-C12 |
| SSBP1 | 0.526 | 0.996 | 0.971 | 9.75E-29 | 2.50E-24 | Ep-C12 |
| DUT | 0.631 | 0.97 | 0.884 | 1.26E-28 | 3.23E-24 | Ep-C12 |
| C18orf32 | 0.563 | 0.902 | 0.75 | 1.52E-28 | 3.89E-24 | Ep-C12 |
| RCN2 | 0.542 | 0.991 | 0.93 | 1.71E-28 | 4.39E-24 | Ep-C12 |
| RHBDL1 | 0.574 | 0.53 | 0.219 | 1.81E-28 | 4.63E-24 | Ep-C12 |
| LIMA1 | 0.686 | 0.936 | 0.76 | 1.90E-28 | 4.88E-24 | Ep-C12 |
| ATP6V1F | 0.515 | 1 | 0.956 | 1.92E-28 | 4.92E-24 | Ep-C12 |
| ABHD3 | 0.603 | 0.637 | 0.381 | 2.08E-28 | 5.34E-24 | Ep-C12 |
| RABIF | 0.581 | 0.658 | 0.364 | 2.08E-28 | 5.34E-24 | Ep-C12 |
| PARD3 | 0.64 | 0.714 | 0.504 | 2.10E-28 | 5.38E-24 | Ep-C12 |
| SMCO4 | 0.639 | 0.637 | 0.324 | 2.34E-28 | 5.99E-24 | Ep-C12 |
| RIOK3 | 0.56 | 0.953 | 0.854 | 2.56E-28 | 6.56E-24 | Ep-C12 |
| AKIRIN2 | 0.592 | 0.825 | 0.594 | 2.97E-28 | 7.61E-24 | Ep-C12 |
| PIH1D1 | 0.552 | 0.893 | 0.664 | 3.17E-28 | 8.13E-24 | Ep-C12 |
| PHB | 0.53 | 0.996 | 0.971 | 3.45E-28 | 8.84E-24 | Ep-C12 |
| CBX1 | 0.586 | 0.889 | 0.716 | 3.70E-28 | 9.49E-24 | Ep-C12 |
| PLCXD2 | 0.665 | 0.56 | 0.232 | 3.71E-28 | 9.50E-24 | Ep-C12 |
| PHF20 | 0.526 | 0.795 | 0.626 | 4.23E-28 | 1.08E-23 | Ep-C12 |
| NPDC1 | 0.664 | 0.607 | 0.284 | 5.22E-28 | 1.34E-23 | Ep-C12 |
| HMGN2 | 0.631 | 1 | 0.968 | 5.35E-28 | 1.37E-23 | Ep-C12 |
| TACC1 | 0.549 | 0.748 | 0.404 | 5.46E-28 | 1.40E-23 | Ep-C12 |
| SOCS1 | 0.744 | 0.769 | 0.471 | 6.64E-28 | 1.70E-23 | Ep-C12 |
| PCNP | 0.522 | 0.953 | 0.875 | 6.92E-28 | 1.77E-23 | Ep-C12 |
| HOXB2 | 0.643 | 0.688 | 0.389 | 8.37E-28 | 2.14E-23 | Ep-C12 |
| VDAC3 | 0.521 | 0.983 | 0.898 | 9.37E-28 | 2.40E-23 | Ep-C12 |
| MRPL17 | 0.568 | 0.902 | 0.763 | 1.06E-27 | 2.72E-23 | Ep-C12 |
| NRARP | 0.691 | 1 | 0.923 | 1.11E-27 | 2.86E-23 | Ep-C12 |
| CCNL1 | 0.608 | 1 | 0.985 | 1.23E-27 | 3.16E-23 | Ep-C12 |
| MOCS2 | 0.601 | 0.803 | 0.518 | 1.28E-27 | 3.29E-23 | Ep-C12 |
| NOL6 | 0.514 | 0.534 | 0.213 | 1.59E-27 | 4.07E-23 | Ep-C12 |
| ATP2B1-AS1 | 0.574 | 0.444 | 0.181 | 1.68E-27 | 4.30E-23 | Ep-C12 |
| PTGER4 | 0.613 | 0.513 | 0.221 | 2.01E-27 | 5.16E-23 | Ep-C12 |
| IRS2 | 0.698 | 0.744 | 0.458 | 2.06E-27 | 5.28E-23 | Ep-C12 |
| KANSL1 | 0.557 | 0.799 | 0.624 | 2.45E-27 | 6.29E-23 | Ep-C12 |
| FBXO2 | 0.585 | 0.692 | 0.341 | 2.49E-27 | 6.39E-23 | Ep-C12 |
| MBNL1-AS1 | 0.634 | 0.393 | 0.221 | 3.02E-27 | 7.75E-23 | Ep-C12 |
| SF3B4 | 0.554 | 0.863 | 0.658 | 3.33E-27 | 8.53E-23 | Ep-C12 |
| PYROXD1 | 0.534 | 0.615 | 0.305 | 3.36E-27 | 8.60E-23 | Ep-C12 |
| SMS | 0.732 | 0.927 | 0.824 | 3.91E-27 | 1.00E-22 | Ep-C12 |
| COMMD2 | 0.525 | 0.949 | 0.814 | 4.23E-27 | 1.08E-22 | Ep-C12 |
| PSMB3 | 0.518 | 1 | 0.992 | 9.21E-27 | 2.36E-22 | Ep-C12 |
| DEK | 0.525 | 1 | 0.966 | 1.06E-26 | 2.71E-22 | Ep-C12 |
| TBC1D2 | 0.581 | 0.688 | 0.426 | 1.20E-26 | 3.08E-22 | Ep-C12 |
| TP53I13 | 0.543 | 0.761 | 0.538 | 1.63E-26 | 4.18E-22 | Ep-C12 |
| LY6E | 0.574 | 1 | 0.987 | 2.15E-26 | 5.52E-22 | Ep-C12 |
| TXNRD1 | 0.661 | 0.936 | 0.751 | 2.28E-26 | 5.86E-22 | Ep-C12 |
| CRYAB | 1.139 | 0.679 | 0.443 | 2.62E-26 | 6.73E-22 | Ep-C12 |
| SS18L2 | 0.53 | 0.897 | 0.743 | 2.65E-26 | 6.79E-22 | Ep-C12 |
| TPRG1 | 0.886 | 0.534 | 0.254 | 3.27E-26 | 8.38E-22 | Ep-C12 |
| C15orf61 | 0.576 | 0.791 | 0.555 | 3.85E-26 | 9.86E-22 | Ep-C12 |
| CYBA | 0.51 | 1 | 0.975 | 4.50E-26 | 1.15E-21 | Ep-C12 |
| BCL10 | 0.641 | 0.803 | 0.597 | 4.51E-26 | 1.16E-21 | Ep-C12 |
| ALDOC | 0.577 | 0.65 | 0.364 | 5.53E-26 | 1.42E-21 | Ep-C12 |
| PIGX | 0.551 | 0.966 | 0.886 | 5.66E-26 | 1.45E-21 | Ep-C12 |
| SNHG1 | 0.562 | 0.744 | 0.569 | 6.56E-26 | 1.68E-21 | Ep-C12 |
| CREB5 | 0.648 | 0.513 | 0.21 | 6.64E-26 | 1.70E-21 | Ep-C12 |
| NIFK | 0.526 | 0.944 | 0.86 | 7.30E-26 | 1.87E-21 | Ep-C12 |
| LARP6 | 0.582 | 0.761 | 0.516 | 7.62E-26 | 1.95E-21 | Ep-C12 |
| GTPBP4 | 0.511 | 0.812 | 0.675 | 1.30E-25 | 3.33E-21 | Ep-C12 |
| HSD17B8 | 0.554 | 0.714 | 0.493 | 1.33E-25 | 3.41E-21 | Ep-C12 |
| TUBB2A | 0.687 | 0.923 | 0.717 | 1.53E-25 | 3.91E-21 | Ep-C12 |
| ACY1 | 0.511 | 0.645 | 0.35 | 1.69E-25 | 4.34E-21 | Ep-C12 |
| HDDC3 | 0.542 | 0.624 | 0.383 | 1.94E-25 | 4.96E-21 | Ep-C12 |
| ODR4 | 0.523 | 0.662 | 0.422 | 2.00E-25 | 5.12E-21 | Ep-C12 |
| TPR | 0.525 | 0.979 | 0.907 | 2.12E-25 | 5.44E-21 | Ep-C12 |
| TSPAN13 | 0.607 | 0.863 | 0.636 | 2.24E-25 | 5.73E-21 | Ep-C12 |
| CALML3 | 0.682 | 0.991 | 0.929 | 2.50E-25 | 6.41E-21 | Ep-C12 |
| BCLAF1 | 0.544 | 0.957 | 0.843 | 3.08E-25 | 7.90E-21 | Ep-C12 |
| PCBD1 | 0.543 | 0.966 | 0.85 | 4.96E-25 | 1.27E-20 | Ep-C12 |
| FZD7 | 0.559 | 0.547 | 0.252 | 5.88E-25 | 1.51E-20 | Ep-C12 |
| UCP2 | 0.52 | 0.513 | 0.201 | 6.11E-25 | 1.57E-20 | Ep-C12 |
| PLEKHA3 | 0.535 | 0.645 | 0.4 | 6.30E-25 | 1.61E-20 | Ep-C12 |
| SPINT1-AS1 | 0.563 | 0.709 | 0.433 | 6.66E-25 | 1.71E-20 | Ep-C12 |
| SNAPC5 | 0.539 | 0.684 | 0.472 | 7.39E-25 | 1.89E-20 | Ep-C12 |
| ESF1 | 0.545 | 0.833 | 0.653 | 8.34E-25 | 2.14E-20 | Ep-C12 |
| NPC2 | 0.533 | 0.991 | 0.934 | 9.62E-25 | 2.47E-20 | Ep-C12 |
| DCUN1D5 | 0.511 | 0.932 | 0.828 | 1.05E-24 | 2.69E-20 | Ep-C12 |
| TCF15 | 0.525 | 0.47 | 0.177 | 1.21E-24 | 3.09E-20 | Ep-C12 |
| OARD1 | 0.524 | 0.795 | 0.592 | 1.36E-24 | 3.48E-20 | Ep-C12 |
| DNAJB6 | 0.519 | 1 | 0.965 | 1.45E-24 | 3.72E-20 | Ep-C12 |
| PPP1R16A | 0.572 | 0.769 | 0.582 | 1.62E-24 | 4.15E-20 | Ep-C12 |
| SFPQ | 0.502 | 1 | 0.972 | 2.13E-24 | 5.45E-20 | Ep-C12 |
| TTC39C | 0.554 | 0.761 | 0.527 | 2.16E-24 | 5.54E-20 | Ep-C12 |
| EXOSC4 | 0.521 | 0.872 | 0.663 | 4.56E-24 | 1.17E-19 | Ep-C12 |
| NAT14 | 0.514 | 0.547 | 0.267 | 5.57E-24 | 1.43E-19 | Ep-C12 |
| SNHG17 | 0.515 | 0.885 | 0.765 | 7.35E-24 | 1.88E-19 | Ep-C12 |
| HSD17B1 | 0.566 | 0.594 | 0.419 | 7.89E-24 | 2.02E-19 | Ep-C12 |
| FAM136A | 0.527 | 0.893 | 0.76 | 1.06E-23 | 2.71E-19 | Ep-C12 |
| ERVK3-1 | 0.509 | 0.722 | 0.521 | 1.15E-23 | 2.94E-19 | Ep-C12 |
| BAMBI | 0.612 | 0.402 | 0.162 | 1.16E-23 | 2.97E-19 | Ep-C12 |
| JAG1 | 0.59 | 0.97 | 0.848 | 1.18E-23 | 3.02E-19 | Ep-C12 |
| TNC | 1.048 | 0.62 | 0.395 | 1.56E-23 | 4.01E-19 | Ep-C12 |
| KLF4 | 0.641 | 0.923 | 0.815 | 2.89E-23 | 7.42E-19 | Ep-C12 |
| PSMC1 | 0.502 | 0.987 | 0.951 | 2.93E-23 | 7.50E-19 | Ep-C12 |
| BAG2 | 0.533 | 0.585 | 0.281 | 3.31E-23 | 8.48E-19 | Ep-C12 |
| SERINC1 | 0.538 | 0.808 | 0.612 | 4.69E-23 | 1.20E-18 | Ep-C12 |
| RUNX1 | 0.554 | 0.748 | 0.538 | 5.83E-23 | 1.49E-18 | Ep-C12 |
| CCDC107 | 0.504 | 0.765 | 0.451 | 6.64E-23 | 1.70E-18 | Ep-C12 |
| LUC7L2 | 0.508 | 0.889 | 0.766 | 8.19E-23 | 2.10E-18 | Ep-C12 |
| FBXO22 | 0.516 | 0.744 | 0.534 | 1.83E-22 | 4.70E-18 | Ep-C12 |
| ID4 | 0.633 | 0.543 | 0.243 | 1.91E-22 | 4.88E-18 | Ep-C12 |
| RAB18 | 0.516 | 0.868 | 0.675 | 3.58E-22 | 9.18E-18 | Ep-C12 |
| IVNS1ABP | 0.523 | 0.88 | 0.678 | 4.31E-22 | 1.10E-17 | Ep-C12 |
| DEGS1 | 0.558 | 0.863 | 0.641 | 4.42E-22 | 1.13E-17 | Ep-C12 |
| MFSD2A | 0.513 | 0.564 | 0.317 | 5.28E-22 | 1.35E-17 | Ep-C12 |
| LINC01980 | 0.529 | 0.709 | 0.472 | 5.92E-22 | 1.52E-17 | Ep-C12 |
| RGS2 | 0.532 | 0.547 | 0.253 | 6.38E-22 | 1.63E-17 | Ep-C12 |
| CTNNAL1 | 0.769 | 0.59 | 0.332 | 6.47E-22 | 1.66E-17 | Ep-C12 |
| ARID1A | 0.503 | 0.756 | 0.559 | 9.31E-22 | 2.39E-17 | Ep-C12 |
| NETO2 | 0.505 | 0.585 | 0.313 | 1.18E-21 | 3.03E-17 | Ep-C12 |
| ARL5B | 0.561 | 0.654 | 0.387 | 1.27E-21 | 3.25E-17 | Ep-C12 |
| ID3 | 0.676 | 0.748 | 0.45 | 1.34E-21 | 3.44E-17 | Ep-C12 |
| PCMTD1 | 0.523 | 0.838 | 0.65 | 1.56E-21 | 4.00E-17 | Ep-C12 |
| NRP2 | 0.741 | 0.799 | 0.591 | 1.77E-21 | 4.55E-17 | Ep-C12 |
| SLC20A2 | 0.525 | 0.782 | 0.534 | 2.45E-21 | 6.29E-17 | Ep-C12 |
| ADH7 | 0.636 | 0.816 | 0.6 | 3.62E-21 | 9.27E-17 | Ep-C12 |
| PFN2 | 0.516 | 0.957 | 0.867 | 4.25E-21 | 1.09E-16 | Ep-C12 |
| ACYP1 | 0.538 | 0.709 | 0.488 | 4.53E-21 | 1.16E-16 | Ep-C12 |
| WEE1 | 0.546 | 0.791 | 0.57 | 5.10E-21 | 1.31E-16 | Ep-C12 |
| NOP16 | 0.522 | 0.675 | 0.462 | 5.48E-21 | 1.40E-16 | Ep-C12 |
| CDKN1C | 0.73 | 0.654 | 0.387 | 5.52E-21 | 1.42E-16 | Ep-C12 |
| ARID5B | 0.54 | 0.962 | 0.822 | 7.40E-21 | 1.90E-16 | Ep-C12 |
| JUND | 0.503 | 1 | 0.995 | 1.04E-20 | 2.67E-16 | Ep-C12 |
| PLEKHA5 | 0.58 | 0.786 | 0.547 | 1.10E-20 | 2.82E-16 | Ep-C12 |
| IER5 | 0.586 | 0.932 | 0.793 | 2.83E-20 | 7.26E-16 | Ep-C12 |
| CASC19 | 0.775 | 0.543 | 0.285 | 3.41E-20 | 8.75E-16 | Ep-C12 |
| ERRFI1 | 0.62 | 0.65 | 0.41 | 3.44E-20 | 8.82E-16 | Ep-C12 |
| NR4A2 | 0.547 | 0.603 | 0.326 | 5.31E-20 | 1.36E-15 | Ep-C12 |
| CITED2 | 0.549 | 0.487 | 0.253 | 6.16E-20 | 1.58E-15 | Ep-C12 |
| ZBTB38 | 0.637 | 0.701 | 0.469 | 6.28E-20 | 1.61E-15 | Ep-C12 |
| MAFB | 0.579 | 0.859 | 0.625 | 1.24E-19 | 3.17E-15 | Ep-C12 |
| GOLIM4 | 0.573 | 0.833 | 0.65 | 1.24E-19 | 3.19E-15 | Ep-C12 |
| SRXN1 | 0.583 | 0.722 | 0.513 | 3.41E-19 | 8.73E-15 | Ep-C12 |
| C2CD4B | 0.521 | 0.278 | 0.079 | 3.64E-19 | 9.32E-15 | Ep-C12 |
| MLLT11 | 0.507 | 0.457 | 0.196 | 3.97E-19 | 1.02E-14 | Ep-C12 |
| HMOX1 | 0.711 | 0.393 | 0.146 | 4.41E-19 | 1.13E-14 | Ep-C12 |
| FCGRT | 0.517 | 0.808 | 0.605 | 6.97E-19 | 1.79E-14 | Ep-C12 |
| CLUAP1 | 0.577 | 0.59 | 0.461 | 8.11E-19 | 2.08E-14 | Ep-C12 |
| DLK2 | 0.557 | 0.509 | 0.271 | 8.45E-19 | 2.17E-14 | Ep-C12 |
| CTSF | 0.505 | 0.534 | 0.272 | 8.95E-19 | 2.29E-14 | Ep-C12 |
| WNT5A | 0.554 | 0.547 | 0.288 | 9.00E-19 | 2.31E-14 | Ep-C12 |
| MXRA7 | 0.502 | 0.876 | 0.704 | 1.09E-18 | 2.80E-14 | Ep-C12 |
| DNAJA4 | 0.506 | 0.603 | 0.341 | 1.90E-18 | 4.88E-14 | Ep-C12 |
| BNIP3 | 0.692 | 0.795 | 0.534 | 3.01E-18 | 7.71E-14 | Ep-C12 |
| TNS4 | 0.51 | 0.842 | 0.625 | 3.39E-18 | 8.69E-14 | Ep-C12 |
| ING1 | 0.507 | 0.709 | 0.471 | 4.91E-18 | 1.26E-13 | Ep-C12 |
| LINC02762 | 0.572 | 0.496 | 0.25 | 5.01E-18 | 1.28E-13 | Ep-C12 |
| IER3 | 0.623 | 0.996 | 0.969 | 5.58E-18 | 1.43E-13 | Ep-C12 |
| GMNN | 0.542 | 0.684 | 0.454 | 6.48E-18 | 1.66E-13 | Ep-C12 |
| WDR54 | 0.502 | 0.671 | 0.44 | 8.30E-18 | 2.13E-13 | Ep-C12 |
| PHLDA1 | 0.539 | 0.829 | 0.576 | 9.31E-17 | 2.39E-12 | Ep-C12 |
| EGR3 | 0.609 | 0.624 | 0.388 | 1.08E-16 | 2.78E-12 | Ep-C12 |
| KLF6 | 0.629 | 0.906 | 0.781 | 3.23E-15 | 8.27E-11 | Ep-C12 |
| MSMO1 | 0.516 | 0.816 | 0.692 | 1.99E-13 | 5.11E-09 | Ep-C12 |
| C6orf15 | 0.861 | 0.107 | 0.016 | 1.68E-12 | 4.30E-08 | Ep-C12 |
| CCL26 | 0.605 | 0.252 | 0.14 | 3.58E-10 | 9.18E-06 | Ep-C12 |
| RGCC | 0.522 | 0.521 | 0.384 | 6.32E-08 | 0.001620459 | Ep-C12 |

| Supplementary Table 3 The gene signatures of tumor epithelial lineages | | | | | | | | | | | | |
| --- | --- | --- | --- | --- | --- | --- | --- | --- | --- | --- | --- | --- |
| Ep-C0 | Ep-C1 | Ep-C2 | Ep-C3 | Ep-C4 | Ep-C5 | Ep-C6 | Ep-C7 | Ep-C8 | Ep-C9 | Ep-C10 | Ep-C11 | Ep-C12 |
| NR4A1 | CCL20 | CES1 | VEGFA | UBE2C | SPON2 | HIST1H4C | LAMC2 | SPRR3 | FABP4 | HLA-DRB1 | FGF19 | ZNF322 |
| CXCL14 | LCN2 | NTS | ERO1A | CENPF | SELENOP | PCLAF | TGFBI | SPRR2E | S100A7 | CD74 | RBM42 | SS18 |
| GEM | PDZK1IP1 | AL033397.1 | PTHLH | CCNB1 | CA2 | RRM2 | P3H2 | SPRR2A | RBP1 | NUPR1 | MAGEB2 | AOAH |
| EGR3 | NUDT8 | OSGIN1 | SLC6A8 | TOP2A | LGALS7 | TYMS | CAV1 | SPRR2D | GJB2 | IFIT3 | SHKBP1 | RBBP8 |
| TXNIP | LYN | PART1 | P4HA1 | CDC20 | LUM | CDK1 | LGALS1 | SPRR1B | PDZK1IP1 | HLA-DRA | COX8C | KCTD1 |
| ID3 | LINC01133 | EPHX1 | EGLN3 | CDKN3 | COPZ2 | ZWINT | COL17A1 | PI3 | BPGM | HLA-DPA1 | FGF3 | NUDT14 |
| HOPX | MUC4 | CYP4F3 | PLOD2 | TPX2 | HSPB3 | TK1 | INHBA | RHCG | CYB5R1 | HLA-F | TIMM50 | HEY1 |
| SNRPN | EHF | TCF4 | GJA1 | ASPM | CRABP2 | CENPM | LAMB3 | KRT13 | IL36G | HLA-DPB1 | SAP25 | TAF4B |
| MIR22HG | TNFAIP2 | CYP4F11 | ENO2 | KPNA2 | IL20RB | UBE2T | PMEPA1 | CNFN | TMEM132A | RTP4 | MEST | CAMK1G |
| CDKN2A | DEFB1 | GSTM2 | NOL3 | BIRC5 | SERPINB3 | RAD51AP1 | PDPN | S100A7 | PLAU | OASL | SOHLH2 | MAGEA4 |
| CKB | CXCL17 | ABCA4 | SLC16A3 | CCNB2 | S100A4 | ATAD2 | LAMA3 | SLPI | RHCG | HLA-DQB1 | AC010198.2 | CTAG2 |
| EPCAM | ASS1 | ABCC1 | CA9 | PLK1 | IGFBP6 | MYBL2 | VIM | LCN2 | ALDH2 | IFIT1 | MCM5 | ZNF131 |
| FSCN1 | CALML5 | AP002387.2 | MME | NUSAP1 | PPP2R5A | HMGB2 | COL5A2 | KRT6C | LGALS7B | HLA-DQA1 | LINC00665 | UBAP2 |
| ITGB4 | EPB41L1 | TRAPPC6A | TMEM91 | HMMR | LGALS7B | PCNA | IGFBP6 | CD24 | AMTN | C1S | COPS7A | PLAT |
| LAPTM4B | AL445524.1 | SNHG19 | NDUFA4L2 | CKS2 | AC100801.1 | DHFR | MMP2 | SERPINB3 | IL1RN | RSAD2 | GTSF1 | CRABP1 |
| MLF1 | GAL | GSTM4 | AF165147.1 | HMGB2 | TMPRSS11E | RFC4 | EMP3 | CLIC3 | SERPINB4 | IL32 | AC009509.1 | SNHG15 |
| NTRK2 | TNIP3 | ALDH1A1 | BNIP3 | MKI67 | SERPINB13 | MCM7 | CTSV | SERPINB4 | HCAR3 | IFIT2 | AP003555.1 | ADSSL1 |
|  | FAM3B | SLC7A11 | VIM | CDK1 | EDIL3 | SPC25 | FLNA | AQP3 | THBD | BST2 | HEBP1 | C1QTNF12 |
|  | CPT1A | SNX31 | SLCO1B3 | PTTG1 | CDA | MAD2L1 | TNFRSF12A | IGFBP3 | SDCBP2 | IFIH1 | WBP11 | TFF2 |
|  | CLDN7 | DUXAP8 | AHNAK2 | CENPA | AL161431.1 | SMC2 | ANO1 | SLURP2 | CXCL14 | UBD | RRP7A | DUSP2 |
|  | ADH1C | GSTM1 | PNCK | DLGAP5 | UPK3BL1 | CDC45 | SEMA3C | IVL | C10orf99 | GBP4 | DHCR7 | BTBD6 |
|  | S100A7 | STXBP6 | LGALS1 | CDCA3 | CRISPLD1 | FEN1 | SERPINE2 | IL1RN | STRA6 | XAF1 | SFRP2 | MRPS30 |
|  | COTL1 | CEP68 | CLEC2B | NUF2 | UPK1B | GINS2 | SERINC2 | IL36G | SERPINB3 | IFI44L | CDH2 | BEX4 |
|  | PSPH | ADH7 | MIR210HG | CENPE | DSC2 | ORC6 | SERPINH1 | SBSN | HCAR2 | IDO1 | BOP1 | NUDT2 |
|  | HLA-DRA | DRAM2 | LGALS7 | ECT2 | IGFL2-AS1 | TMEM106C | PRSS23 | S100A12 | SERPINA3 | ISG20 | SERPINH1 | IGFBP4 |
|  | STEAP1 | RAB3B | IGFL2-AS1 | PRC1 | MIR99AHG | HIST1H1A | COL12A1 | PRSS3 | TUBB2A | HLA-DMA | KDM5A | AKT1 |
|  | CXCL8 | AKR1B10 | PGF | AURKA | TPPP3 | CDCA5 | CAVIN3 | MALL | RAB38 | DDX58 | LINC02582 | KLHL42 |
|  | NTS | CPT1A | AC011632.1 | KIF20B | SERPINB9 | PBK | COL4A2 | LYPD3 | CD24 | PLAAT4 | AC092490.1 | ZNF667-AS1 |
|  | BBC3 | EPS8 | P4HA2 | NCAPD2 | GJA1 | CLSPN | PTHLH | GJB2 | GJB6 | CMPK2 | PEG10 | CRIP1 |
|  | HCAR3 | PLAC8 | COL17A1 | GTSE1 | TMEM265 | TOP2A | MFAP5 | KLK12 | FERMT1 | HLA-DMB | PHLDA1 | FAM133A |
|  | CRYAB | RBP7 | CRIP2 | SMC4 | WNT4 | PKMYT1 | AMIGO2 | TGM1 | HES2 | TRIM22 | USP5 | ETNK1 |
|  | RARRES1 | TESMIN | P3H2 | TROAP | PALMD | SMC4 | COL4A1 | SULT2B1 | MMP7 | C1R | IGFLR1 | DEDD2 |
|  | AKR1B10 | BCL11A | LUM | NEK2 | DCN | DNMT1 | LPCAT2 | RAB11FIP1 | CDKN1A | LIME1 | C8orf88 | MAP1LC3A |
|  | GSTA1 | PAK1 | TMEM265 | CEP55 | FABP4 | CDC6 | MMP28 | GJB6 | PLAUR | OAS3 | FHL1 | NDUFAF4 |
|  | CFB | GSTA1 | EMP3 | CCNA2 | MAF | ASF1B | DAAM1 | MXD1 | KYNU | CTSS | DNM1L | CT69 |
|  | HLA-DMA | CALML5 | IGFBP6 | MAD2L1 | CDKN2A | MCM3 | CRIP2 | CYSRT1 | RGCC | EPSTI1 | LINC00839 | UCHL1 |
|  | TMPRSS11A | CTSF | CDKN2A | AURKB | CKB | CDT1 | ACTN1 | SPRR1A | KRT6C | SAA1 | TNFRSF6B | TGIF1 |
|  | HILPDA | TSPAN7 | CKB | KIF2C | EPCAM | RPL39L | PLEK2 | CLDN4 | SAA1 | OAS2 | ANO1 | TM7SF3 |
|  | CES1 | MBIP | EPCAM | CKAP2 | FSCN1 | NUSAP1 | ITGB6 | SDCBP2 | CDH3 | DDX60 | EEF2KMT | ARL4D |
|  | GBP1 | WNT5A | FSCN1 | KIF23 | ITGB4 | MKI67 | CAVIN1 | C10orf99 | PRDM1 | GBP1 | TRIB2 | CLDN20 |
|  | FAM83A | SPP1 | ITGB4 | KNSTRN | LAPTM4B | UBE2C | FLRT2 | KLK11 | INHBA | MIR99AHG | DYRK4 | MRPL50 |
|  | CDKN2A | ALG1L | LAPTM4B | CDCA8 | MLF1 | HELLS | ITGA3 | SERPINB13 | APOBEC3A | KYNU | COL6A1 | CCN1 |
|  | CKB | ABHD2 | MLF1 | DEPDC1 | NTRK2 | H2AFX | FSTL3 | FAM83A | HAS3 | GBP2 | LPCAT3 | UBE2R2 |
|  | EPCAM | CLUAP1 | NTRK2 | ANLN |  | BIRC5 | COL7A1 | DSC2 | CRABP2 | MMP7 | MT-ND6 | DNAJB4 |
|  | FSCN1 | UGDH |  | PBK |  | ESCO2 | PLS3 | PRSS22 | TMEM45A | ZNFX1 | TEAD4 | SPSB1 |
|  | ITGB4 | RGMA |  | SGO2 |  | FOXM1 | GALNT2 | NECTIN4 | SPRR2D | PCDH7 | EXOSC5 | GOLT1B |
|  | LAPTM4B | TM7SF2 |  | CENPW |  | MCM4 | P4HA2 | AC105460.1 | MAP7D1 | SAMD9L | IGHMBP2 | PTHLH |
|  | MLF1 | PAX9 |  | MZT1 |  | RFC2 | MSC | KLK13 | SPRR1B | MIR22HG | ETV5 | SNRPN |
|  | NTRK2 | CHL1 |  | H2AFX |  | MCM5 | CDK6 | NCCRP1 | SERPINB13 | CDKN2A | SMIM10L1 | JAG2 |
|  |  | CDKN2A |  | UBE2T |  | GMNN | CSPG4 | CLDN7 | PALMD | CKB | TPCN2 | C11orf1 |
|  |  | CKB |  | SKA2 |  | CENPK | WDR66 | GRHL1 | TMEM79 | EPCAM | MAGOHB | RSRP1 |
|  |  | EPCAM |  | TACC3 |  | AURKB | TUBB6 | KLK8 | OVOL1 | FSCN1 | PXDN | ACTR6 |
|  |  | FSCN1 |  | FOXM1 |  | CENPU | NDFIP2 | METRNL | CD82 | ITGB4 | FGF4 | HAS3 |
|  |  | ITGB4 |  | HMGB3 |  | RRM1 | FAP | SAMD9 | CDC42EP1 | LAPTM4B | LTO1 | IFRD1 |
|  |  | LAPTM4B |  | FAM83D |  | BRCA1 | FXYD5 | TMEM40 | OAF | MLF1 | MAEL | MED21 |
|  |  | MLF1 |  | ANP32E |  | DTYMK | SERPINE1 | TMPRSS11D | MXD1 | NTRK2 | ARHGAP5-AS1 | AKR1C1 |
|  |  | NTRK2 |  | KIF11 |  | CENPH | ITGAV | SMIM22 | CITED4 |  | VIM | BICD2 |
|  |  |  |  | RACGAP1 |  | TMPO | MYL9 | UGCG | SQOR |  | CCDC88A | NEDD9 |
|  |  |  |  | CKAP5 |  | FANCI | ITGA5 | PRSS27 | TMEM265 |  | CLDN10 | MTHFS |
|  |  |  |  | PIMREG |  | CENPN | SLITRK6 | SMIM5 | ALDH1A3 |  | SERPINE2 | JAKMIP2 |
|  |  |  |  | NDC80 |  | DNAJC9 | TGM2 | TMEM79 | KLF7 |  | BCAT1 | PODXL2 |
|  |  |  |  | DTYMK |  | FBXO5 | CDH3 | TMEM45B | GM2A |  | GNG4 | MRPS35 |
|  |  |  |  | KIF14 |  | MELK | LAMC1 | EPS8L1 | CLCA2 |  | COL6A2 | KRBOX1 |
|  |  |  |  | CDC25B |  | FAM111B | PDGFA | FUT3 | GNA15 |  | PLEKHA5 | SLC4A11 |
|  |  |  |  | BUB1 |  | VRK1 | MMP13 | PPL | HOMER3 |  | FGFR1OP2 | NAV1 |
|  |  |  |  | KIF4A |  | TPX2 | GALNT1 | NDRG2 | LYPD3 |  | ZNF22 | CACNA2D3 |
|  |  |  |  | TTK |  | MND1 | USB1 | NIBAN2 | TMPRSS11D |  | ZNF146 | ISCA1 |
|  |  |  |  | TNFAIP8L1 |  | GGH | KDELR3 | CD68 | PRSS8 |  | SARS2 | RHOB |
|  |  |  |  | CENPN |  | TUBG1 | CAV2 | ABLIM1 | RIN2 |  | BMS1 | ARL6IP6 |
|  |  |  |  | NCAPG |  | NDC80 | AREG | MBOAT2 | TFAP2A |  | GINS2 | EHMT1 |
|  |  |  |  | ARHGAP11A |  | ATAD5 | LOXL2 | ARHGAP27 | EPAS1 |  | LGALS1 | PHYHD1 |
|  |  |  |  | GPSM2 |  | DIAPH3 | LGALS7 | GRHL3 | IL1B |  | BORCS5 | NDUFA4L2 |
|  |  |  |  | PRR11 |  | PRC1 | IL7R | CEACAM1 | AKR1B10 |  | LTBP4 | FGFR2 |
|  |  |  |  | KIFC1 |  | CENPF | VEGFC | LIPH | FRMD4B |  | SDHAF1 | AL078590.2 |
|  |  |  |  | CKAP2L |  | CDCA3 | COL4A6 | EPHX3 | EIF1AY |  | RIMKLB | WDR33 |
|  |  |  |  | LMNB1 |  | TCF19 | TENM2 | ASPG | WNT4 |  | NFKBID | TMEM267 |
|  |  |  |  | KIF20A |  | DSCC1 | SLC38A5 | SCEL | KLK8 |  | FH | SNHG12 |
|  |  |  |  | RANGAP1 |  | NCAPG | RRAS | TTC9 | BMP2 |  | PTX3 | FST |
|  |  |  |  | CDCA2 |  | HIST1H1B | TINAGL1 | ALDH3B2 | TNFRSF12A |  | DKK1 | IDI1 |
|  |  |  |  | KIF22 |  | NUF2 | COPZ2 | ATP11B | TINCR |  | PDCD2L | HAPLN2 |
|  |  |  |  | SMC2 |  | CCNA2 | UPP1 | EPHA2 | MMP28 |  | CTNNAL1 | RPP25L |
|  |  |  |  | TMPO |  | RAD51 | ITGB5 | KRT23 | KLC3 |  | BHLHE41 | RIC3 |
|  |  |  |  | LMNB2 |  | SGO1 | CRELD2 | PRDM1 | CD68 |  | LRP6 | CEP170B |
|  |  |  |  | DBF4 |  | KIF23 | TGFBR2 | FAM3D | SGPP2 |  | H2AFY2 | TOM1L1 |
|  |  |  |  | KNL1 |  | ASPM | FHL2 | TMPRSS2 | ACKR3 |  | CAVIN3 | SIGMAR1 |
|  |  |  |  | PSRC1 |  | RFC5 | FERMT1 | MUC4 | GJA1 |  | PGM2L1 | AC007996.1 |
|  |  |  |  | TRIM59 |  | NCAPH | KIFC3 | KRT80 | CYB561A3 |  | ETV4 | TUBA1A |
|  |  |  |  | TK1 |  | ANLN | PDGFC | MUC20 | PXDC1 |  | SAMM50 | GMPR |
|  |  |  |  | SGO1 |  | TEX30 | WNT10A | PIM1 | S100A7A |  | NPTX2 | INSIG1 |
|  |  |  |  | DDX39A |  | KIF11 | LUM | BICDL2 | IGSF3 |  | LRRCC1 | SEZ6L2 |
|  |  |  |  | HJURP |  | PSMC3IP | IKBIP | ECM1 | MYO1B |  | PAQR4 | CDCA4 |
|  |  |  |  | CIP2A |  | CENPW | SPON2 | PLEKHM1 | FLNB |  | SSPN | FXYD6 |
|  |  |  |  | SAPCD2 |  | LIG1 | MFSD10 | LINC01133 | SPRR2A |  | TYSND1 | SRSF6 |
|  |  |  |  | SPC25 |  | SKA3 | MSN | SDR9C7 | KCTD11 |  | ADSL | UBAP1 |
|  |  |  |  | NCAPH |  | BRCA2 | NELL2 | TMPRSS11A | FAM83A |  | CENPM | AC105460.1 |
|  |  |  |  | MIS18BP1 |  | HJURP | CD109 | HK2 | SBSN |  | ADIPOR2 | UNC50 |
|  |  |  |  | TEDC1 |  | FANCA | BMP1 | FLG | RAI14 |  | MCM4 | MYH11 |
|  |  |  |  | ZWINT |  | HIST1H1D | NEFL | S100P | PLK3 |  | TMEM88 | DGKA |
|  |  |  |  | KIF18A |  | CHAF1A | MYO1B | PRSS8 | DENND2C |  | CDT1 | ETFRF1 |
|  |  |  |  | PHF19 |  | EXO1 | GJA1 | CYP3A5 | PON2 |  | ILF3-DT | DDIT3 |
|  |  |  |  | SPAG5 |  | WDR34 | BASP1 | B4GALT4 | DUSP14 |  | YARS2 | LYRM4 |
|  |  |  |  | EMC9 |  | KIFC1 | ARNTL2 | OAS1 | NRIP1 |  | YIF1B | AC023157.3 |
|  |  |  |  | ARHGEF39 |  | TFDP1 | ETS1 | A2ML1 | MAFB |  | PGP | ZNRD1 |
|  |  |  |  | RPL39L |  | MNS1 | MSC-AS1 | TGFA | JARID2 |  | TIMM23 | SPX |
|  |  |  |  | CCNF |  | POLD3 | PYGL | ANKRD22 | DSC2 |  | C19orf54 | ABT1 |
|  |  |  |  | PIF1 |  | MCM10 | ARL6IP5 | S100A7A | ZNF750 |  | NOVA1 | ACYP2 |
|  |  |  |  | DEPDC1B |  | GTSE1 | PDLIM7 | PCDH1 | TNS4 |  | LINC00392 | EGR2 |
|  |  |  |  | BORA |  | FANCB | TAGLN | SERPINB11 | ANXA3 |  | RECQL | EPC1 |
|  |  |  |  | GGH |  | TEDC2 | EHD2 | C15orf48 | HBEGF |  | DNMT1 | TSPYL1 |
|  |  |  |  | CIT |  | NCAPD3 | CTNNAL1 | CERS3 | CRYAB |  | PYCR3 | POP5 |
|  |  |  |  | BCL2L12 |  | USP1 | GPR87 | GCNT3 | SERPINE1 |  | JMJD8 | CAMK2N1 |
|  |  |  |  | RNF26 |  | CDKN3 | NRG1 | RNF141 | CDA |  | ZNF585A | SOX2 |
|  |  |  |  | BUB1B |  | RFC3 | PLOD3 | BNIPL | CAPNS2 |  | SUPT16H | WNK2 |
|  |  |  |  | KIF15 |  | HIST2H2AC | MAGEA10 | UBAP1 | ID2 |  | MAGEA3 | ADRA1D |
|  |  |  |  | PARPBP |  | SAE1 | LOX | ZNF185 | TUBB3 |  | MAGEA6 | HAGLROS |
|  |  |  |  | OIP5 |  | DTL | KC877982.1 | DCAF12 | CDKN2A |  | HSPA6 | LSM12 |
|  |  |  |  | POC1A |  | SLBP | AC011632.1 | EVPL | CKB |  | EGLN2 | TMEM158 |
|  |  |  |  | GAS2L3 |  | CEP55 | CALD1 | DEGS2 | EPCAM |  | SLC12A2 | TMEM116 |
|  |  |  |  | CDC25C |  | CKLF | MDFI | NCF2 | FSCN1 |  | SNRPA | AL590483.2 |
|  |  |  |  | RRM2 |  | CIP2A | TSKU | COX7A1 | ITGB4 |  | TK1 | PRXL2C |
|  |  |  |  | KIF18B |  | KIF15 | SYT8 | ERBB3 | LAPTM4B |  | TULP3 | AC025171.1 |
|  |  |  |  | LBR |  | MCM2 | TNFRSF6B | EHF | MLF1 |  | SNRNP25 | DCAF12 |
|  |  |  |  | MXD3 |  | FAM111A | HMGA2 | C6orf132 | NTRK2 |  | HEY1 | PRKRA |
|  |  |  |  | RAD51AP1 |  | TRIP13 | ANXA8 | CXCL17 |  |  | COQ8B | AKIRIN1 |
|  |  |  |  | NMU |  | KIF2C | IL20RB | CLCA4 |  |  | MPDU1 | AL365181.4 |
|  |  |  |  | SKA3 |  | DSN1 | AC100801.1 | CYB5R1 |  |  | FOXM1 | FKBP10 |
|  |  |  |  | CCDC18 |  | ECT2 | NT5E | SPRR2F |  |  | CSTF2T | CCDC91 |
|  |  |  |  | CDCA5 |  | TACC3 | TGFB1I1 | PTK6 |  |  | RHNO1 | OSER1 |
|  |  |  |  | INCENP |  | KNL1 | PLAU | LY6G6C |  |  | ARHGDIB | SLCO1B3 |
|  |  |  |  | CCDC34 |  | CDCA4 | FKBP11 | ZDHHC13 |  |  | MCM7 | NUPR2 |
|  |  |  |  | TRIP13 |  | SUPT16H | CRISPLD1 | SLURP1 |  |  | FXYD5 | LYRM2 |
|  |  |  |  | CNTRL |  | PRIM1 | GLG1 | SMAGP |  |  | TRAP1 | SLC25A33 |
|  |  |  |  | SHCBP1 |  | TEDC1 | MAP7D1 | CRB3 |  |  | NPW | TXNDC5 |
|  |  |  |  | DIAPH3 |  | SNRNP25 | IRAK1 | SPTSSB |  |  | IARS2 | ULBP2 |
|  |  |  |  | SPDL1 |  | RECQL4 | EDIL3 | B3GNT8 |  |  | POLR3K | CMAS |
|  |  |  |  | CEP70 |  | CCDC34 | COL27A1 | B3GNT5 |  |  | EID2 | PAK1 |
|  |  |  |  | G2E3 |  | LRRCC1 | HSPB3 | F3 |  |  | GPS1 | BEX2 |
|  |  |  |  | SUN2 |  | MTFR2 | GLS | TMPRSS11E |  |  | MAP9 | ADM |
|  |  |  |  | C16orf95 |  | PXMP2 | MAP4K4 | TRIM16 |  |  | SPSB2 | DUSP6 |
|  |  |  |  | HYLS1 |  | ITGB3BP | PPT1 | AC008397.1 |  |  | RAB30-DT | OSGIN1 |
|  |  |  |  | PCLAF |  | KIF20B | INO80C | TTC22 |  |  | SPR | MTX2 |
|  |  |  |  | MND1 |  | CDCA8 | PPFIBP1 | GNA15 |  |  | HMGA2 | TRA2A |
|  |  |  |  | CENPM |  | MTHFD1 | CDH13 | FAM3B |  |  | ACSL4 | CDH11 |
|  |  |  |  | PGP |  | POLA2 | GJB3 | MAP3K8 |  |  | FADD | YARS2 |
|  |  |  |  | GOT1 |  | RMI2 | DKK3 | HILPDA |  |  | PRMT5 | PCSK1N |
|  |  |  |  | SCLT1 |  | LMNB1 | EREG | MRPS30-DT |  |  | COL4A2 | ETV4 |
|  |  |  |  | MYBL2 |  | TTK | TNC | CEACAM5 |  |  | AC092821.3 | MYO10 |
|  |  |  |  | ORC6 |  | LRR1 | DSG2 | MUC1 |  |  | GTF2E2 | ZNF749 |
|  |  |  |  | NEURL1B |  | MASTL | TM9SF2 | TMEM265 |  |  | NOP2 | NR4A1 |
|  |  |  |  | MTFR2 |  | MIS18BP1 | SRPX | PDZK1IP1 |  |  | FKBP11 | THYN1 |
|  |  |  |  | CSE1L |  | PSIP1 | MAF | DNAJC5 |  |  | DRG1 | NPPC |
|  |  |  |  | NUP37 |  | E2F8 | HOMER3 | CITED4 |  |  | U2AF1L4 | BCAM |
|  |  |  |  | MELK |  | C21orf58 | MARVELD1 | DUOX2 |  |  | RASSF8 | HMCES |
|  |  |  |  | NUDCD2 |  | E2F1 | SULF2 | MYH14 |  |  | AGR2 | PAK1IP1 |
|  |  |  |  | REEP4 |  | SPC24 | POPDC3 | CRYBG2 |  |  | RAD51AP1 | FKBP9 |
|  |  |  |  | MAGOHB |  | CKAP2L | GPX8 | FAM214B |  |  | MTHFD1 | LINC02562 |
|  |  |  |  | RUVBL2 |  | CCNE2 | MAP4 | KLK10 |  |  | LMNB2 | MSRB2 |
|  |  |  |  | CENPK |  | RBBP7 | TGFB1 | RHOV |  |  | ARL4C | TMEM98 |
|  |  |  |  | ODF2 |  | RACGAP1 | ELOVL1 | QSOX1 |  |  | NR2F2 | CHORDC1 |
|  |  |  |  | FANCI |  | TIMELESS | HTRA1 | DDAH2 |  |  | HAUS5 | FAM241B |
|  |  |  |  | VRK1 |  | NCAPG2 | P3H1 | IKZF2 |  |  | NFKBIB | BYSL |
|  |  |  |  | PAK4 |  | SCCPDH | ECM1 | PROM2 |  |  | RUVBL2 | MGLL |
|  |  |  |  | RHNO1 |  | E2F7 | SPATS2L | PPARD |  |  | MCM3 | PPT1 |
|  |  |  |  | TTF2 |  | RAD51C | EFEMP1 | CDH1 |  |  | LAS1L | NNT |
|  |  |  |  | BRD8 |  | CSE1L | FRMD6 | TRIP10 |  |  | CCN3 | DNAH14 |
|  |  |  |  | LYAR |  | UBR7 | PORCN | SLCO4A1 |  |  | DDX49 | SLC16A8 |
|  |  |  |  | DCAF7 |  | IGFLR1 | EFNB2 | NBEAL2 |  |  | TSPAN9 | PRKAG2-AS1 |
|  |  |  |  | SAP30 |  | SAC3D1 | MAGEA3 | BCL2L1 |  |  | CD320 | DUSP14 |
|  |  |  |  | CENPH |  | KIF22 | CD70 | ST14 |  |  | PRPS2 | TBPL1 |
|  |  |  |  | MIS18A |  | POLD1 | UBE2Q2 | NPEPPS |  |  | FAM217B | HEXIM1 |
|  |  |  |  | RCCD1 |  | SHCBP1 | ZBED2 | MAP1LC3A |  |  | DTYMK | EPB41L4A-AS1 |
|  |  |  |  | STIP1 |  | DDX11 | IRS1 | SH3YL1 |  |  | NFIB | YRDC |
|  |  |  |  | HIST1H2BJ |  | GINS1 | PLXNB2 | AL121761.1 |  |  | TFAM | LINC02298 |
|  |  |  |  | DLEU2 |  | MIS18A | FNDC3B | MAGEA4 |  |  | DPYSL3 | IFT43 |
|  |  |  |  | GRK6 |  | POLR3K | REEP3 | GPX3 |  |  | PRSS21 | COX7A1 |
|  |  |  |  | HIST1H3G |  | HAT1 | PRR16 | AHNAK2 |  |  | CXCL1 | TMEM35B |
|  |  |  |  | RCC1 |  | POC1A | MYO1C | ALOX15B |  |  | IFITM1 | A1BG |
|  |  |  |  | PRPSAP1 |  | BCL2L12 | TPM1 | ZDHHC21 |  |  | KRT7 | AC087612.1 |
|  |  |  |  | PPIH |  | CMC2 | PLXNA1 | TMPRSS4 |  |  | KMT2B | COA5 |
|  |  |  |  | NDE1 |  | CKS2 | DCBLD1 | KLK9 |  |  | ATN1 | AL162231.1 |
|  |  |  |  | CCDC88A |  | WDR76 | RAB32 | CEACAM6 |  |  | KNOP1 | ZSCAN31 |
|  |  |  |  | FOPNL |  | PRIM2 | SEC13 | ADAM15 |  |  | ISYNA1 | NXT1 |
|  |  |  |  | NUDT15 |  | SASS6 | PXN | TMPRSS13 |  |  | MAGEA1 | MBOAT7 |
|  |  |  |  | NSD2 |  | SMC1A | THAP12 | HMOX1 |  |  | MCM6 | AC019080.1 |
|  |  |  |  | SS18 |  | UHRF1 | IGF2BP2 | SLC12A6 |  |  | GOLT1B | SLC27A5 |
|  |  |  |  | DESI2 |  | RECQL | CD276 | RAET1G |  |  | ZWINT | HSPA6 |
|  |  |  |  | CDKN1B |  | CHEK1 | CDA | CDKN1A |  |  | CDC45 | ANAPC13 |
|  |  |  |  | SPTBN1 |  | MAGOHB | ARSJ | GGT6 |  |  | COL3A1 | TM2D3 |
|  |  |  |  | WBP11 |  | MCM8 | DKK1 | SLC20A2 |  |  | MSX1 | LRRC8A |
|  |  |  |  | ETV5 |  | HIRIP3 | COL1A1 | MPZL3 |  |  | DUSP6 | ZNF428 |
|  |  |  |  | IKBIP |  | PAICS | LOXL4 | RBM47 |  |  | SIGIRR | PSMG4 |
|  |  |  |  | FBXO5 |  | TYMSOS | MAGEA11 | CXXC5 |  |  | CXCL3 | ZBTB42 |
|  |  |  |  | CBX5 |  | RHNO1 | LINC01807 | CPEB4 |  |  | BCKDHA | SVIP |
|  |  |  |  | AIMP2 |  | PTN | CA2 | RALB |  |  | PAK1 | HINT2 |
|  |  |  |  | COPS7A |  | TMEM97 | TPBG | EPHB3 |  |  | UNG | IP6K2 |
|  |  |  |  | USP5 |  | CD320 | NT5DC2 | ELF3-AS1 |  |  | GLIPR1 | C1orf52 |
|  |  |  |  | SLC12A2 |  | WDHD1 | TRNAU1AP | KRTDAP |  |  | PTHLH | APOE |
|  |  |  |  | CTNNAL1 |  | CHTF18 | KCNK1 | ZBTB7B |  |  | CLEC2B | MCUR1 |
|  |  |  |  | MEST |  | PGP | FEZ1 | CAMK1G |  |  | EPS8 | ZSCAN18 |
|  |  |  |  | CDKN2A |  | NCAPD2 | FHL3 | DUOX1 |  |  | FOXP1 | SNHG21 |
|  |  |  |  | CKB |  | SKP2 | IL1RAP | RNF149 |  |  | CDKN2A | RPF2 |
|  |  |  |  | EPCAM |  | SSRP1 | LMF2 | ARHGAP32 |  |  | CKB | SIGIRR |
|  |  |  |  | FSCN1 |  | GPN3 | PTK7 | SEMA3F |  |  | EPCAM | TBCC |
|  |  |  |  | ITGB4 |  | TONSL | NCLN | CHST2 |  |  | FSCN1 | PINLYP |
|  |  |  |  | LAPTM4B |  | CDCA7L | INTS1 | RABIF |  |  | ITGB4 | RPP25 |
|  |  |  |  | MLF1 |  | ZWILCH | ERO1A | KDM7A-DT |  |  | LAPTM4B | CLEC2L |
|  |  |  |  | NTRK2 |  | C19orf48 | MAD1L1 | GFOD2 |  |  | MLF1 | NME4 |
|  |  |  |  |  |  | CENPS | AC004556.3 | NDUFA4L2 |  |  | NTRK2 | ZNF226 |
|  |  |  |  |  |  | RNASEH2B | MFSD12 | EMP1 |  |  |  | SLC25A25 |
|  |  |  |  |  |  | CEP78 | TTYH3 | FUT2 |  |  |  | ARG2 |
|  |  |  |  |  |  | TTF2 | KLF7 | C5orf66-AS1 |  |  |  | GRASP |
|  |  |  |  |  |  | TOPBP1 | MED15 | PGM2 |  |  |  | SBSPON |
|  |  |  |  |  |  | DDX39A | PLOD2 | RAB31 |  |  |  | RAC3 |
|  |  |  |  |  |  | BARD1 | SPHK1 | SNX18 |  |  |  | ETV5 |
|  |  |  |  |  |  | HADH | THBS1 | CEL |  |  |  | CASC15 |
|  |  |  |  |  |  | LRRC45 | ACOT9 | GPR160 |  |  |  | NNT-AS1 |
|  |  |  |  |  |  | RPA2 | AIM2 | TM7SF2 |  |  |  | ELOVL5 |
|  |  |  |  |  |  | CBX5 | AC245041.2 | APOL1 |  |  |  | FAM229B |
|  |  |  |  |  |  | PHGDH | CYTOR | HDHD3 |  |  |  | AC024588.1 |
|  |  |  |  |  |  | HIST1H1E | BCAM | CEP170B |  |  |  | TNFSF9 |
|  |  |  |  |  |  | SHMT1 | GNA12 | PPP1R11 |  |  |  | BEX1 |
|  |  |  |  |  |  | CENPQ | GLIPR1 | ZNF385A |  |  |  | SHC1 |
|  |  |  |  |  |  | EZH2 | SLC7A8 | ZMYND8 |  |  |  | MTA1 |
|  |  |  |  |  |  | UNG | FAM50A | CNN3 |  |  |  | SCCPDH |
|  |  |  |  |  |  | KPNA2 | CFH | SRD5A3 |  |  |  | GSTM4 |
|  |  |  |  |  |  | CMSS1 | TUSC3 | MFSD1 |  |  |  | BX284668.5 |
|  |  |  |  |  |  | ACAT2 | SULF1 | ABI1 |  |  |  | DLX5 |
|  |  |  |  |  |  | HAUS1 | SORL1 | PHLDB3 |  |  |  | KLHL23 |
|  |  |  |  |  |  | FANCG | FN1 | SDR16C5 |  |  |  | FGFR1OP2 |
|  |  |  |  |  |  | LMNB2 | FADD | LYN |  |  |  | SNHG32 |
|  |  |  |  |  |  | CDK2 | MFAP2 | ARRDC1 |  |  |  | ACOT13 |
|  |  |  |  |  |  | YEATS4 | NPTN | DUOXA1 |  |  |  | GTF2H4 |
|  |  |  |  |  |  | BRI3BP | EVA1B | IL1A |  |  |  | THAP9-AS1 |
|  |  |  |  |  |  | OXCT1 | SNX8 | SCAMP2 |  |  |  | C20orf194 |
|  |  |  |  |  |  | HAUS5 | NRIP1 | AMN |  |  |  | NEK11 |
|  |  |  |  |  |  | NCAPH2 | AGRN | RORA |  |  |  | B4GALT4 |
|  |  |  |  |  |  | PAQR4 | DSE | RAPGEFL1 |  |  |  | TM2D2 |
|  |  |  |  |  |  | RFWD3 | AC103702.2 | NOTCH3 |  |  |  | SMU1 |
|  |  |  |  |  |  | MRPL37 | ANKLE2 | B3GALT4 |  |  |  | NSG1 |
|  |  |  |  |  |  | TCOF1 | STON2 | TINF2 |  |  |  | MRAP2 |
|  |  |  |  |  |  | HIST1H3G | ERAP2 | TSPAN1 |  |  |  | PPP1R14A |
|  |  |  |  |  |  | EXOSC8 | PDLIM2 | KRT7 |  |  |  | GGPS1 |
|  |  |  |  |  |  | MCM6 | COL4A5 | ERMP1 |  |  |  | RASD1 |
|  |  |  |  |  |  | ACYP1 | AP003555.1 | ANGPTL4 |  |  |  | 9-Mar |
|  |  |  |  |  |  | NUDT15 | IGHG3 | SQOR |  |  |  | CDC42EP3 |
|  |  |  |  |  |  | TRAP1 | BCAP29 | MGLL |  |  |  | GPANK1 |
|  |  |  |  |  |  | POLE3 | ADGRG1 | UNC5B-AS1 |  |  |  | SMIM13 |
|  |  |  |  |  |  | C5orf34 | GOLIM4 | ABHD17C |  |  |  | FOXE1 |
|  |  |  |  |  |  | SAP30 | RECQL | SORT1 |  |  |  | MESP1 |
|  |  |  |  |  |  | FN3KRP | PTGFRN | TMEM184A |  |  |  | SRSF8 |
|  |  |  |  |  |  | NSD2 | MYLK | TICAM1 |  |  |  | DIPK2A |
|  |  |  |  |  |  | DBF4 | MAGEA6 | EPS8L2 |  |  |  | AKIP1 |
|  |  |  |  |  |  | TMEM107 | INAVA | ZNF750 |  |  |  | BCL11B |
|  |  |  |  |  |  | SHMT2 | C1GALT1 | CAMK2N1 |  |  |  | SNCA |
|  |  |  |  |  |  | NETO2 | PDE7A | CRABP2 |  |  |  | TMEM99 |
|  |  |  |  |  |  | HIST1H2BJ | GJB5 | TMEM159 |  |  |  | CLDN7 |
|  |  |  |  |  |  | CENPV | SLC39A6 | NAGK |  |  |  | BDH2 |
|  |  |  |  |  |  | PRPS2 | AHNAK2 | COBLL1 |  |  |  | HES4 |
|  |  |  |  |  |  | RRP7A | FRMD4B | RIT1 |  |  |  | PTBP2 |
|  |  |  |  |  |  | RBBP8 | ELL2 | H19 |  |  |  | LGMN |
|  |  |  |  |  |  | SNRPA | MT1M | SERINC2 |  |  |  | BRF1 |
|  |  |  |  |  |  | PEG10 | SUN2 | CDCP1 |  |  |  | STK38L |
|  |  |  |  |  |  | MSH2 | CTHRC1 | C12orf29 |  |  |  | EXOSC3 |
|  |  |  |  |  |  | TOR3A | TK1 | B4GALT1 |  |  |  | TCTEX1D2 |
|  |  |  |  |  |  | CTNNAL1 | CDCP1 | SLC44A2 |  |  |  | AEBP2 |
|  |  |  |  |  |  | TMEM38B | IDS | GALNT3 |  |  |  | DTWD1 |
|  |  |  |  |  |  | ARL6IP6 | WNT7B | CALML5 |  |  |  | NEFM |
|  |  |  |  |  |  | PNKP | TM4SF19 | ANXA3 |  |  |  | PPP1R10 |
|  |  |  |  |  |  | MTHFD2 | VSIR | SOWAHC |  |  |  | SCN9A |
|  |  |  |  |  |  | TIMM50 | MMP1 | GPR87 |  |  |  | SPRY2 |
|  |  |  |  |  |  | PAK1 | RASAL2 | HAS3 |  |  |  | C19orf48 |
|  |  |  |  |  |  | FH | IGFL2-AS1 | GPCPD1 |  |  |  | POLB |
|  |  |  |  |  |  | PSAT1 | DCN | REEP4 |  |  |  | TTC32 |
|  |  |  |  |  |  | SVIP | CPE | KCNK6 |  |  |  | NOS1AP |
|  |  |  |  |  |  | GNG4 | IGFBP7 | TJP2 |  |  |  | RHBDL1 |
|  |  |  |  |  |  | WBP11 | IFI44 | AFDN |  |  |  | ABHD3 |
|  |  |  |  |  |  | USP5 | ACKR3 | ISG20 |  |  |  | RABIF |
|  |  |  |  |  |  | MAGEA1 | BIK | RNF11 |  |  |  | PARD3 |
|  |  |  |  |  |  | EXOSC5 | CDKN2A | AL445524.1 |  |  |  | SMCO4 |
|  |  |  |  |  |  | STXBP6 | CKB | TPRG1 |  |  |  | AKIRIN2 |
|  |  |  |  |  |  | MEST | EPCAM | DENND2C |  |  |  | PIH1D1 |
|  |  |  |  |  |  | EPS8 | FSCN1 | KRT31 |  |  |  | PLCXD2 |
|  |  |  |  |  |  | NFKBID | ITGB4 | DUSP5 |  |  |  | NPDC1 |
|  |  |  |  |  |  | UCHL1 | LAPTM4B | EDN2 |  |  |  | TACC1 |
|  |  |  |  |  |  | SIGMAR1 | MLF1 | ERBB2 |  |  |  | SOCS1 |
|  |  |  |  |  |  | HEY1 | NTRK2 | GALE |  |  |  | HOXB2 |
|  |  |  |  |  |  | CDKN2A |  | HS3ST1 |  |  |  | MOCS2 |
|  |  |  |  |  |  | CKB |  | FAM110C |  |  |  | NOL6 |
|  |  |  |  |  |  | EPCAM |  | HOXB2 |  |  |  | ATP2B1-AS1 |
|  |  |  |  |  |  | FSCN1 |  | CCDC80 |  |  |  | PTGER4 |
|  |  |  |  |  |  | ITGB4 |  | TMEM267 |  |  |  | IRS2 |
|  |  |  |  |  |  | LAPTM4B |  | TMEM154 |  |  |  | FBXO2 |
|  |  |  |  |  |  | MLF1 |  | DEFB1 |  |  |  | SF3B4 |
|  |  |  |  |  |  | NTRK2 |  | GPRC5A |  |  |  | PYROXD1 |
|  |  |  |  |  |  |  |  | CRYAB |  |  |  | TBC1D2 |
|  |  |  |  |  |  |  |  | OVOL1 |  |  |  | TP53I13 |
|  |  |  |  |  |  |  |  | THBD |  |  |  | CRYAB |
|  |  |  |  |  |  |  |  | CDKN2A |  |  |  | TPRG1 |
|  |  |  |  |  |  |  |  | CKB |  |  |  | C15orf61 |
|  |  |  |  |  |  |  |  | EPCAM |  |  |  | BCL10 |
|  |  |  |  |  |  |  |  | FSCN1 |  |  |  | ALDOC |
|  |  |  |  |  |  |  |  | ITGB4 |  |  |  | CREB5 |
|  |  |  |  |  |  |  |  | LAPTM4B |  |  |  | LARP6 |
|  |  |  |  |  |  |  |  | MLF1 |  |  |  | HSD17B8 |
|  |  |  |  |  |  |  |  | NTRK2 |  |  |  | TUBB2A |
|  |  |  |  |  |  |  |  |  |  |  |  | ACY1 |
|  |  |  |  |  |  |  |  |  |  |  |  | HDDC3 |
|  |  |  |  |  |  |  |  |  |  |  |  | ODR4 |
|  |  |  |  |  |  |  |  |  |  |  |  | TSPAN13 |
|  |  |  |  |  |  |  |  |  |  |  |  | FZD7 |
|  |  |  |  |  |  |  |  |  |  |  |  | UCP2 |
|  |  |  |  |  |  |  |  |  |  |  |  | PLEKHA3 |
|  |  |  |  |  |  |  |  |  |  |  |  | SPINT1-AS1 |
|  |  |  |  |  |  |  |  |  |  |  |  | SNAPC5 |
|  |  |  |  |  |  |  |  |  |  |  |  | TCF15 |
|  |  |  |  |  |  |  |  |  |  |  |  | OARD1 |
|  |  |  |  |  |  |  |  |  |  |  |  | TTC39C |
|  |  |  |  |  |  |  |  |  |  |  |  | EXOSC4 |
|  |  |  |  |  |  |  |  |  |  |  |  | NAT14 |
|  |  |  |  |  |  |  |  |  |  |  |  | ERVK3-1 |
|  |  |  |  |  |  |  |  |  |  |  |  | BAMBI |
|  |  |  |  |  |  |  |  |  |  |  |  | TNC |
|  |  |  |  |  |  |  |  |  |  |  |  | BAG2 |
|  |  |  |  |  |  |  |  |  |  |  |  | RUNX1 |
|  |  |  |  |  |  |  |  |  |  |  |  | CCDC107 |
|  |  |  |  |  |  |  |  |  |  |  |  | FBXO22 |
|  |  |  |  |  |  |  |  |  |  |  |  | ID4 |
|  |  |  |  |  |  |  |  |  |  |  |  | IVNS1ABP |
|  |  |  |  |  |  |  |  |  |  |  |  | DEGS1 |
|  |  |  |  |  |  |  |  |  |  |  |  | MFSD2A |
|  |  |  |  |  |  |  |  |  |  |  |  | LINC01980 |
|  |  |  |  |  |  |  |  |  |  |  |  | RGS2 |
|  |  |  |  |  |  |  |  |  |  |  |  | CTNNAL1 |
|  |  |  |  |  |  |  |  |  |  |  |  | NETO2 |
|  |  |  |  |  |  |  |  |  |  |  |  | ARL5B |
|  |  |  |  |  |  |  |  |  |  |  |  | ID3 |
|  |  |  |  |  |  |  |  |  |  |  |  | NRP2 |
|  |  |  |  |  |  |  |  |  |  |  |  | SLC20A2 |
|  |  |  |  |  |  |  |  |  |  |  |  | ADH7 |
|  |  |  |  |  |  |  |  |  |  |  |  | ACYP1 |
|  |  |  |  |  |  |  |  |  |  |  |  | WEE1 |
|  |  |  |  |  |  |  |  |  |  |  |  | NOP16 |
|  |  |  |  |  |  |  |  |  |  |  |  | CDKN1C |
|  |  |  |  |  |  |  |  |  |  |  |  | PLEKHA5 |
|  |  |  |  |  |  |  |  |  |  |  |  | CASC19 |
|  |  |  |  |  |  |  |  |  |  |  |  | ERRFI1 |
|  |  |  |  |  |  |  |  |  |  |  |  | NR4A2 |
|  |  |  |  |  |  |  |  |  |  |  |  | CITED2 |
|  |  |  |  |  |  |  |  |  |  |  |  | ZBTB38 |
|  |  |  |  |  |  |  |  |  |  |  |  | MAFB |
|  |  |  |  |  |  |  |  |  |  |  |  | SRXN1 |
|  |  |  |  |  |  |  |  |  |  |  |  | MLLT11 |
|  |  |  |  |  |  |  |  |  |  |  |  | HMOX1 |
|  |  |  |  |  |  |  |  |  |  |  |  | FCGRT |
|  |  |  |  |  |  |  |  |  |  |  |  | DLK2 |
|  |  |  |  |  |  |  |  |  |  |  |  | CTSF |
|  |  |  |  |  |  |  |  |  |  |  |  | WNT5A |
|  |  |  |  |  |  |  |  |  |  |  |  | DNAJA4 |
|  |  |  |  |  |  |  |  |  |  |  |  | BNIP3 |
|  |  |  |  |  |  |  |  |  |  |  |  | TNS4 |
|  |  |  |  |  |  |  |  |  |  |  |  | ING1 |
|  |  |  |  |  |  |  |  |  |  |  |  | LINC02762 |
|  |  |  |  |  |  |  |  |  |  |  |  | GMNN |
|  |  |  |  |  |  |  |  |  |  |  |  | WDR54 |
|  |  |  |  |  |  |  |  |  |  |  |  | PHLDA1 |
|  |  |  |  |  |  |  |  |  |  |  |  | EGR3 |
|  |  |  |  |  |  |  |  |  |  |  |  | CDKN2A |
|  |  |  |  |  |  |  |  |  |  |  |  | CKB |
|  |  |  |  |  |  |  |  |  |  |  |  | EPCAM |
|  |  |  |  |  |  |  |  |  |  |  |  | FSCN1 |
|  |  |  |  |  |  |  |  |  |  |  |  | ITGB4 |
|  |  |  |  |  |  |  |  |  |  |  |  | LAPTM4B |
|  |  |  |  |  |  |  |  |  |  |  |  | MLF1 |
|  |  |  |  |  |  |  |  |  |  |  |  | NTRK2 |
